# Computational mechanistic investigation of the kinetic resolution of α-methyl-phenylacetaldehyde by norcoclaurine synthase

**DOI:** 10.1038/s42004-024-01146-x

**Published:** 2024-03-27

**Authors:** Shiqing Zhang, Chenghua Zhang, Aijing Guo, Baoyan Liu, Hao Su, Xiang Sheng

**Affiliations:** 1 Tianjin Institute of Industrial Biotechnology, Chinese Academy of Sciences, Tianjin, 300308 PR China; 2 National Center of Technology Innovation for Synthetic Biology, National Engineering Research Center of Industrial Enzymes and Key Laboratory of Engineering Biology for Low-Carbon Manufacturing, Tianjin, 300308 PR China; 3 School of Pharmacy, North Sichuan Medical College, Nanchong, 637100 PR China; 4 University of Chinese Academy of Sciences, Beijing, 100049 PR China

**Keywords:** Reaction mechanisms, Computational chemistry, Biocatalysis

## Abstract

Norcoclaurine synthase from *Thalictrum flavum* (*Tf*NCS) demonstrated high stereospecificity and yield in catalyzing the Pictet-Spengler reaction of dopamine with chiral aldehydes, achieving kinetic resolution of aldehydes. However, the mechanism and the factors contributing to the stereoselectivity remain unclear. Herein, by using quantum chemical calculations, the mechanisms of *Tf*NCS-catalyzed reactions of dopamine with both enantiomers of α-methyl-phenylacetaldehyde are studied. The calculations reveal a mechanism mirroring the reaction of natural substrates, for which the deprotonation of the C5−H of the cyclized intermediate is rate-limiting. The calculated overall barriers are 20.1 kcal mol^-1^ and 21.6 kcal mol^-1^ for the reactions of (*R*)- and (*S*)-α-methyl-phenylacetaldehyde, respectively. The M97 and L72 residues are proposed to be the key residues contributing to the stereospecificity. The obtained detailed information is helpful for designing new variants of *Tf*NCS with extended substrate scope, and also advancing our understanding of *Tf*NCS reactions for potential applications.

## Abstract (web summary)

Norcoclaurine synthase from *Thalictrum flavum* (*Tf*NCS) has been demonstrated to display high stereospecificity and yield in catalyzing the Pictet-Spengler reaction of dopamine with chiral aldehydes, however, the mechanism and factors related to this high stereospecificity remain unclear. Here, the authors conduct quantum chemical calculations and reveal the rate-limiting step and differential energy barriers for the reactions of two enantiomers of α-methylphenylacetaldehyde, as well as key residues related to stereospecificity.

## Introduction

The benzylisoquinoline alkaloids (BIAs) are groups of natural products found in plants^1^. Many BIAs have been extensively studied for their pharmacological activities, with examples including berberine, glaucine, morphine, and noscapine, which display antimicrobial, anti-inflammatory, and analgesic properties, respectively^2–5^. Furthermore, compounds like (*R*)-coclaurine and (*S*)-norcoclaurine have demonstrated potent anti-HIV activities^6^. The core structure of BIAs is commonly synthesized by a well-established organic chemistry reaction, namely the Pictet-Spengler (PS) reaction, which involves the condensation of an amine with an aldehyde or ketone. The corresponding enzymes are named as Pictet-Spenglerases (PSases)^7^.

**Fig. 1:**
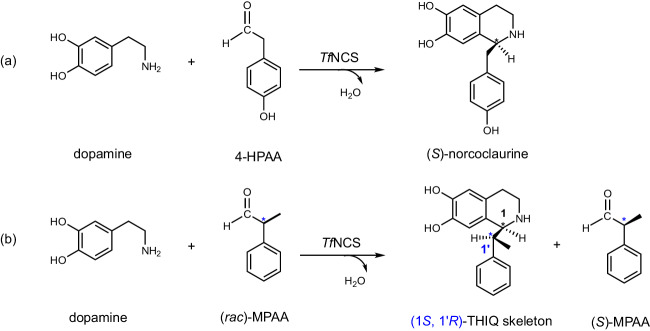
Enzymatic Pictet–Spengler reaction catalyzed by the norcoclaurine synthase from *Thalictrum flavum* (*Tf*NCS). The reaction schemes between the natural amine substrate dopamine and (**a**) the natural aldehyde substrate 4-hydroxyphenylacetaldehyde (4-HPAA) or (**b**) the non-natural aldehyde substrate α-methyl-phenylacetaldehyde (MPAA).

Norcoclaurine synthases (NCSs) are a subset of PSases that catalyze the PS condensation between dopamine and 4-hydroxyphenylacetaldehyde (4-HPAA), leading to the formation of norcoclaurine, which contains a tetrahydroisoquinoline (THIQ) skeleton. This condensation reaction is the key step in the biosynthesis of BIAs^8–11^, making NCSs have received great attention in the designed biosynthetic route of novel alkaloids^12–17^. The NCS from *Thalictrum flavum* (*Tf*NCS) stereospecifically catalyzes the PS reaction between dopamine and 4-HPAA to generate (*S*)-norcoclaurine (Fig. 1a)^8,18,19^. This enzyme exhibits a broad substrate spectrum for aldehydes. For instance, it has high activity in the conversion of phenylacetaldehydes substituted with diverse groups^20,21^. Recently, the enzyme has been shown to accept also various ketone compounds, including phenylacetone, cyclohexanone, and some of their derivatives^22^. For the amine substrate, it has been demonstrated that in addition to the natural dopamine substrate, O-hydroxyphenylethylamine and 1-methoxy-2-hydroxy-phenylethylamine can also be recognized^23^. The broad substrate spectrum makes *Tf*NCS to be a promising biocatalyst in producing non-natural alkaloids.

The PS condensation between amine and achiral aldehydes produces compounds bearing one chiral center. While chiral aldehyde substrates are used, products possessing two chiral centers can be generated. A recent study showed that *Tf*NCS accepts chiral α-substituted aldehydes to produce THIQs with two chiral centers with high conversions^24^. As *Tf*NCS displays (*S*)-enantioselectivity at C1 position, only the (1*S*, 1*’R*)- and (1*S*, 1*’S*)-diastereoisomers can be formed when the aldehyde substrates are (*R*)- and (*S*)-enantiomers, respectively. Interestingly, the enzyme converts the (*R*)-enantiomer more preferentially to (*S*)-enantiomer, thus leading to the kinetic resolution of the chiral aldehyde racemate^24^. Taking α-methyl-phenylacetaldehyde (MPAA) as an example (Fig. 1b), (1S, 1*’R*)-diastereoisomer was experimentally measured to be the major product with diastereoisomeric ratio (d. r.) of 94:6^24^. To further extend the application of *Tf*NCS in the kinetic resolution of chiral aldehydes, it is important to understand the factors controlling the stereoselectivity. To this end, the reaction mechanism should be first revealed.

Many efforts have been devoted to reveal the reaction mechanism of the natural reaction of *Tf*NCS. On the basis of the available crystal structures, the two substrates can be proposed to bind to the active site in different sequence, giving rise to two potential binding modes: the “HPAA-first”^25,26^ and “dopamine-first” binding modes^27,28^. Recent experimental and computational studies proved that the two binding modes are accessible at the stage of the enzymes-substrates complex, but only the “dopamine-first” mechanism is catalytically relevant^24,29^. The reaction mechanism of *Tf*NCS was demonstrated to follow the general mechanism of PS reaction, involving the sequential C–N bond formation, dehydration, cyclization and the final deprotonation of C–H group of the cyclized intermediate. Nevertheless, the detailed reaction mechanisms and the factors controlling the stereospecificity toward the enantiomers of the chiral α-methyl-phenylacetaldehyde remain elusive.

In this work, the quantum chemical calculations using an active site cluster model of the enzyme (Supplementary Fig. 1) were employed to explore the reaction mechanisms of *Tf*NCS-catalyzed condensation between dopamine and the (*R*)- and (*S*)-enantiomers of α-methyl-phenylacetaldehyde and to reveal the factors governing the stereospecificity. Detailed mechanisms are proposed, and the potential sources contributing to the kinetic resolution of α-methyl-phenylacetaldehyde are discussed.

## Results and discussion

### Reaction mechanism

**Fig. 2:**
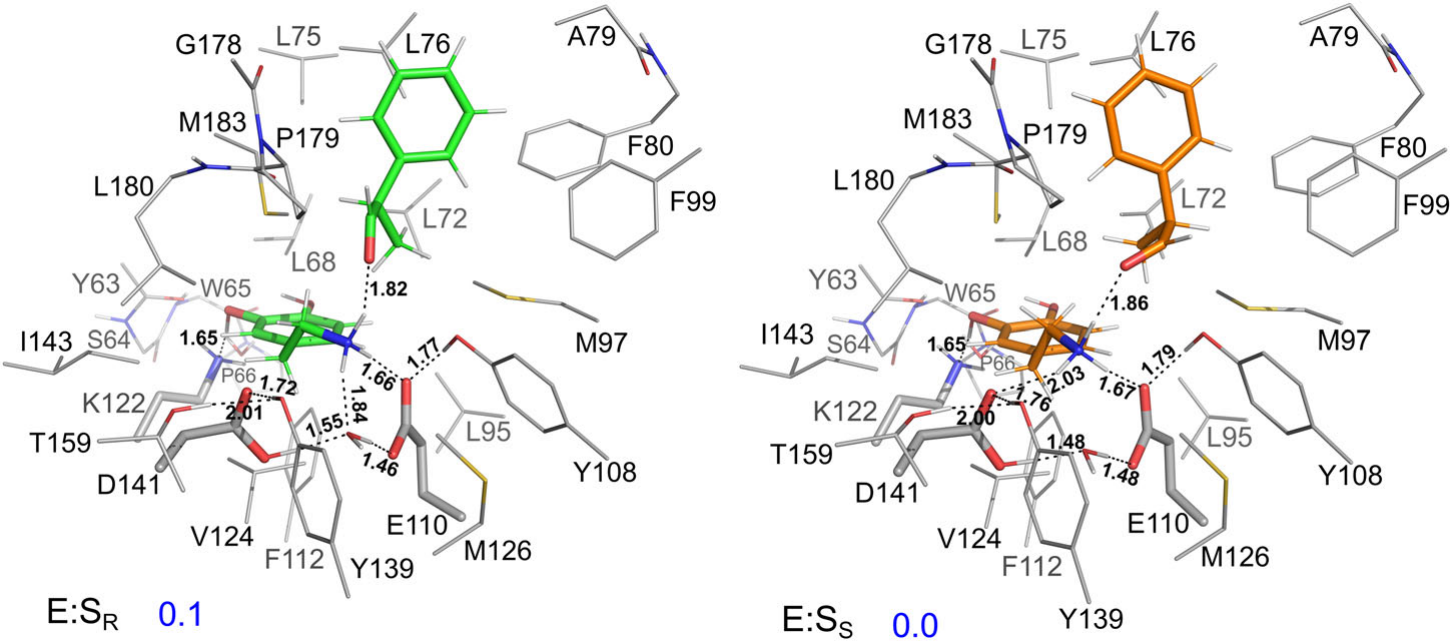
The optimized structures of the lowest-energy enzyme-substrates complexes: *R*-model (E:S_R_) and *S*-model (E:S_S_). The substrates are shown in green and orange stick in E:S_R_ and E:S_S_, respectively. The energies relative to E:S_S_ are given in kcal mol^−1^ and selected distances are given in Å. For clarity, most of the hydrogen atoms are omitted and the constraints made on the model during geometry optimization are indicated in Supplementary Fig. 1.

As discussed above, previous study suggested that the “dopamine-first” binding mode is the catalytically active mode for the *Tf*NCS reaction^29^. Consequently, in the present work, we exclusively considered this binding mode. For both the *R*- and *S*-models, we optimized the geometries of a number of enzyme–substrate complexes with different conformations of the substrates and active site residues, and evaluated the corresponding energies. The structures of the lowest-energy *R*- and *S*-models, denoted as E:S_R_ and E:S_S_, respectively, are presented in Fig. 2, and other structures are shown in Supplementary Figs. 2 and 3. The calculations reveal that E:S_R_ and E:S_S_ have very similar energies, with E:S_S_ being only 0.1 kcal mol^−1^ lower than that of E:S_R_. In both *R*- and *S*-models, the D141 is in the protonated state, while the E110 is deprotonated.

**Fig. 3:**
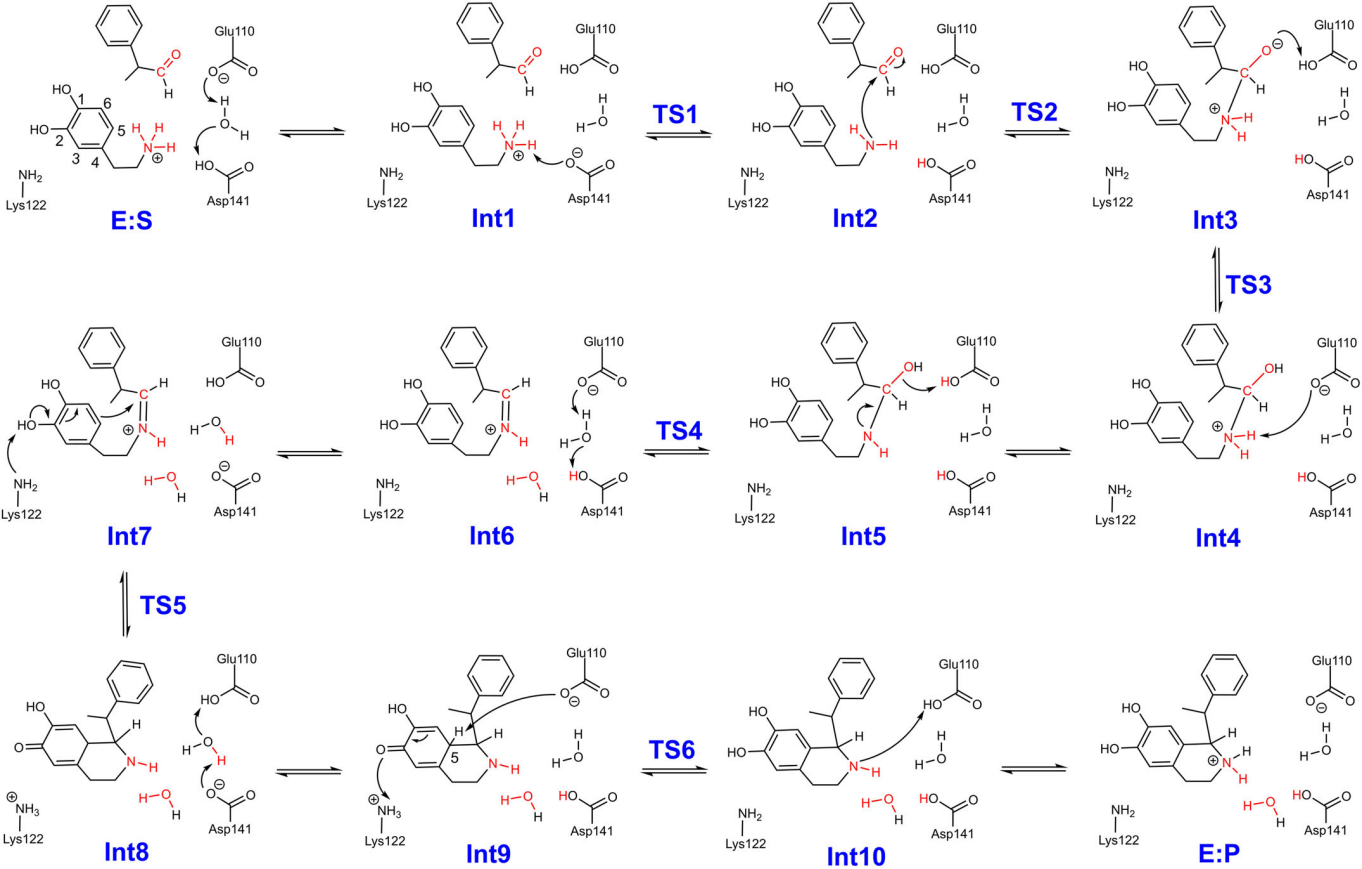
Detailed reaction mechanism suggested on the basis of the current calculations. The reaction between dopamine and α-methyl-phenylacetaldehyde is proposed to follow the “dopamine-first” mechanism, involving the sequential C–N bond formation, dehydration, cyclization and the final deprotonation of C–H group of the cyclized intermediate. A number of proton transfer events occur between the substrates and active site residues throughout the reaction process.

**Fig. 4:**
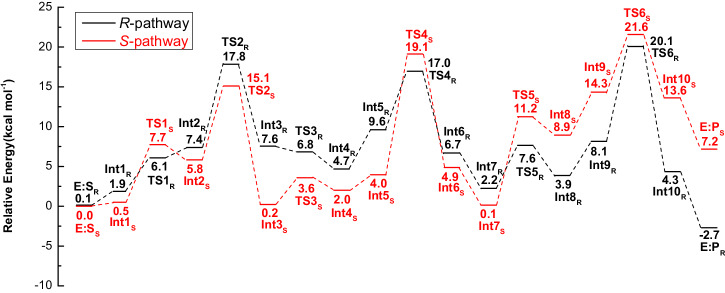
Calculated energy profiles for the *Tf*NCS-catalyzed PS reaction between dopamine and α-methyl-phenylacetaldehyde. Reaction pathways with (*R*)- and (*S*)-MPAA, named as *R*-pathway and *S*-pathway, are shown in black and red, respectively. The energy of E:S_S_ is set as the zero of the energy profiles.

**Fig. 5:**
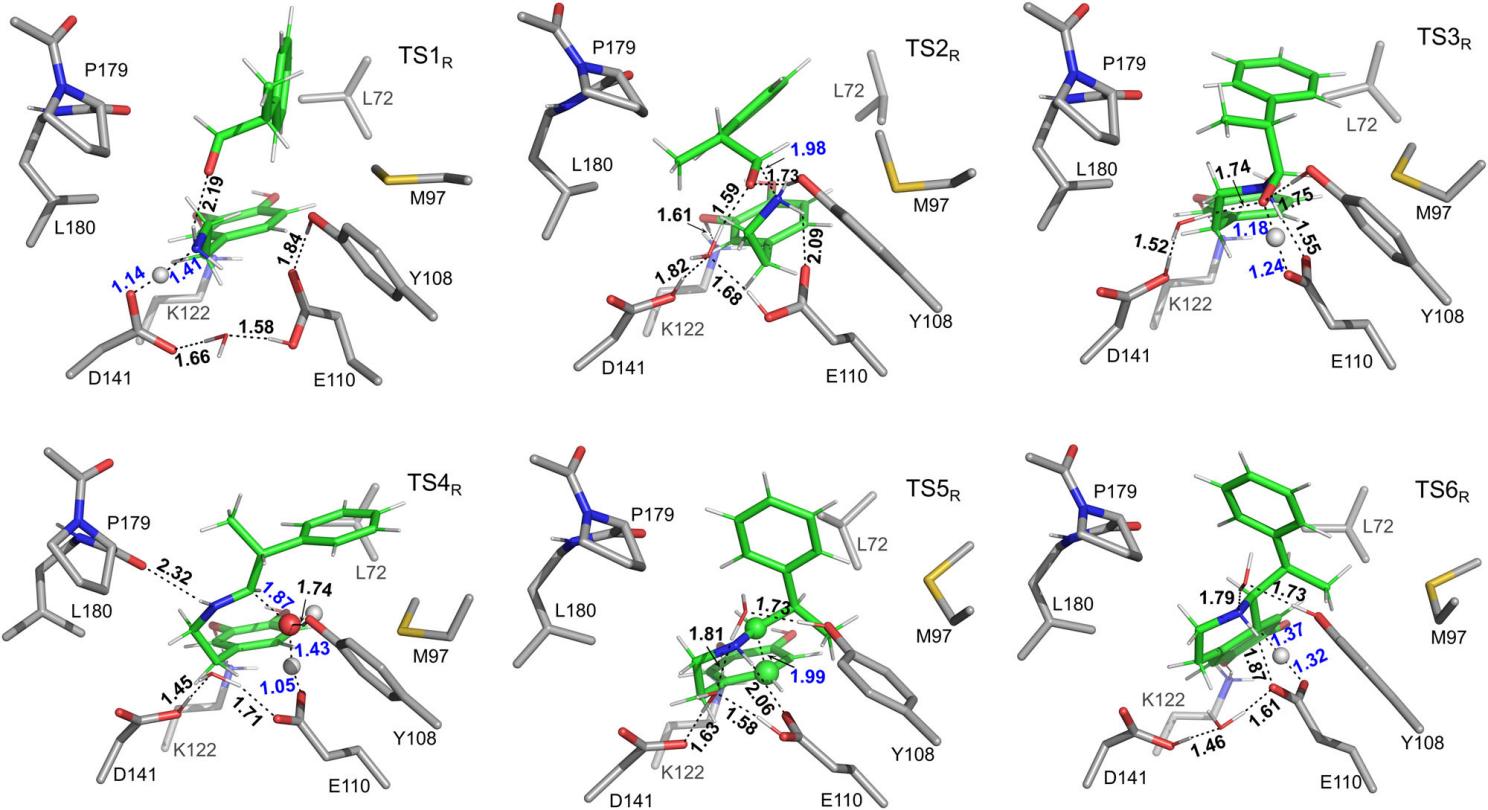
The optimized structures of the transition states involved in the *Tf*NCS-catalyzed PS reaction between dopamine and (*R*)-MPAA. Substrates are depicted as green sticks. Selected distances are given in Å. For clarity, only a small part of the model is shown. Structures with the full model are given in Supplementary Fig. 4.

The reaction mechanisms of the *Tf*NCS-catalyzed condensation of dopamine with the enantiomers of MPAA were then investigated. The calculations revealed that the reaction for *R*- and *S*-models follow the same mechanism (Fig. 3). Given (*R*)-enantiomer is preferential by the enzyme, only the pathway for the PS reaction between dopamine and (*R*)-MPAA (*R*-pathway) is here discussed in detail and compared to the reaction of the natural substrates. The entire catalytic cycle can be divided into four key processes: the nucleophilic attack of dopamine at the aldehyde forming the C–N bond, the dehydration step resulting in the generation of the iminium intermediate, cyclization of the iminium intermediate, and finally, the deprotonation of the cyclized intermediate leading to the generation of the THIQ product. The calculated energy profiles for the catalytic cycle are shown in Fig. 4, in which the energy of E:S_S_ is set as the zero of the energy profiles, and the optimized structures of corresponding species are shown in Fig. 5 and Supplementary Figs. 4 and 5. The cartesian coordinates of the optimized structures of all the species involved in the lowest-energy pathways and their corresponding energies are shown in Supplementary Data 1.

As illustrated in Fig. 3, the reaction starts with the deprotonation of D141 residue by E110 via a water bridge (E:S_R_ → Int1_R_), raising the energy by 1.9 kcal mol^−1^. The deprotonated D141 then extracts a proton from the amino group of the dopamine (Int1_R_ → [TS1_R_] → Int2_R_) with a barrier of 6.1 kcal mol^−1^ relative to E:S_S_, which is very similar to the corresponding step in the natural reaction (5.0 kcal mol^−1^)^29^. The subsequent nucleophilic attack of dopamine at the carbonyl group of (*R*)-MPAA leads to the formation of a tetrahedral intermediate (Int2_R_ → [TS2_R_] → Int3_R_). This step was calculated to have an energy barrier of 17.8 kcal mol^−1^ relative to E:S_S_ (Fig. 4), significantly higher than the equivalent step in the reaction of the natural substrates (8.0 kcal mol^−1^ from the previous study^29^). Structural comparison of the transition states (TSs) showed that the higher barrier for C–N bond formation here could be attributed to the steric hindrance between the α-methyl group of the (*R*)-MPAA substrate and P179, which is not present in the reaction of the natural substrates.

Then, two successive low-barrier proton transfer processes take place, from E110 to the alkoxide (Int3_R_ → [TS3_R_] → Int4_R_) and from ammonium ion to the E110 (Int4_R_ → Int5_R_). Owing to the presence of the steric effect between the P179 residue and the moiety of (*R*)-MPAA, the energies of the intermediates Int4_R_ and Int5_R_ are higher than E:S_S_. This contrasts with the reaction of natural substrates, where the energies of the two corresponding intermediates are lower than their enzyme-substrate complexes. The following dehydration step (Int5_R_ → [TS4_R_] → Int6_R_) was calculated to be 17.0 kcal mol^−1^ relative to E:S_S_, and the resulting iminium intermediate (Int6_R_) is 6.7 kcal mol^-1^ higher than E:S_S_. The overlap of Int6_R_ with the co-crystallized structure of *Tf*NCS with an (*R*)-iminium mimic (PDB code: 6RP3) displays very high similarity (Supplementary Fig. 6), which indicates that our model could give a good representation to the real enzymatic process.

Following the formation of Int6_R_, a proton transfer from D141 to E110 takes place, reducing the energy of the system by 4.5 kcal mol^−1^ (Int6_R_ → Int7_R_). Next, proton abstraction by K122 initiates the cyclization of the iminium intermediate (TS5_R_) with an energy barrier of 5.4 kcal mol^−1^ relative to Int7_R_, and the calculated energy of the cyclized intermediate Int8_R_ is 1.7 kcal mol^−1^ higher than Int7_R_. To form the THIQ skeleton, the C5–H must be deprotonated by a general base group. The most plausible residue is E110 in the vicinity. However, in Int7_R_, this residue is in the protonated form. Therefore, a proton transfer from E110 to D141 takes place (Int8_R_ → Int9_R_) prior to the deprotonation of C5–H group. This proton transfer was calculated to be endothermic by 4.2 kcal mol^−1^. After the proton transfer, the deprotonation of the C5–H of the cyclized intermediate by E110 occurs with a calculated barrier of 20.1 kcal mol^−1^ relative to E:S_S_ (Int9_R_ → [TS6_R_] → Int10_R_). Finally, the proton transfer from E110 to the amine group of the newly-formed six-membered ring generates the final product and restores the enzyme for the next catalytic cycle (Int10_R_ → E:P_R_).

The obtained mechanism and the calculated energy profile for the *Tf*NCS-catalyzed condensation of dopamine and (*R*)-MPAA are generally consistent with the reaction of the natural substrates in the previous study but obvious differences are observed^29^. In particular, the deprotonation of the C5–H group of the cyclized intermediate is rate-limiting in both cases. However, the barrier for the overall pathway of the reaction of (*R*)-MPAA (20.1 kcal mol^−1^) is 1.3 kcal mol^−1^ higher than that of the natural reaction. This is a result of the steric hindrance between the methyl substituent of the substrate and the M97 residue in the reaction of (*R*)-MPAA. In the reaction of 4-HPAA, this effect does not exist, allowing the substrate to adjust the conformation more freely in the active site.

The kinetic data is not available for the considered reaction here but accessible for the reactions of structurally similar aldehyde substrates^30^. The measured *k*_cat_ values for two other non-natural substrates, benzaldehyde and 4-biphenylaldehyde, are 0.237 s^−1^ and 0.001 s^−1^, respectively, which can be converted to barriers of ~19 kcal mol^−1^ and 23 kcal mol^−1^. The calculated barriers for the reactions of (*R*)-MPAA (20.1 kcal mol^−1^) and (*S*)-MPAA (21.6 kcal mol^−1^, see next section) are thus reasonable, as they are in good agreement with the experimentally derived barriers of the two comparable substrates.

### Enantioselectivity

**Fig. 6:**
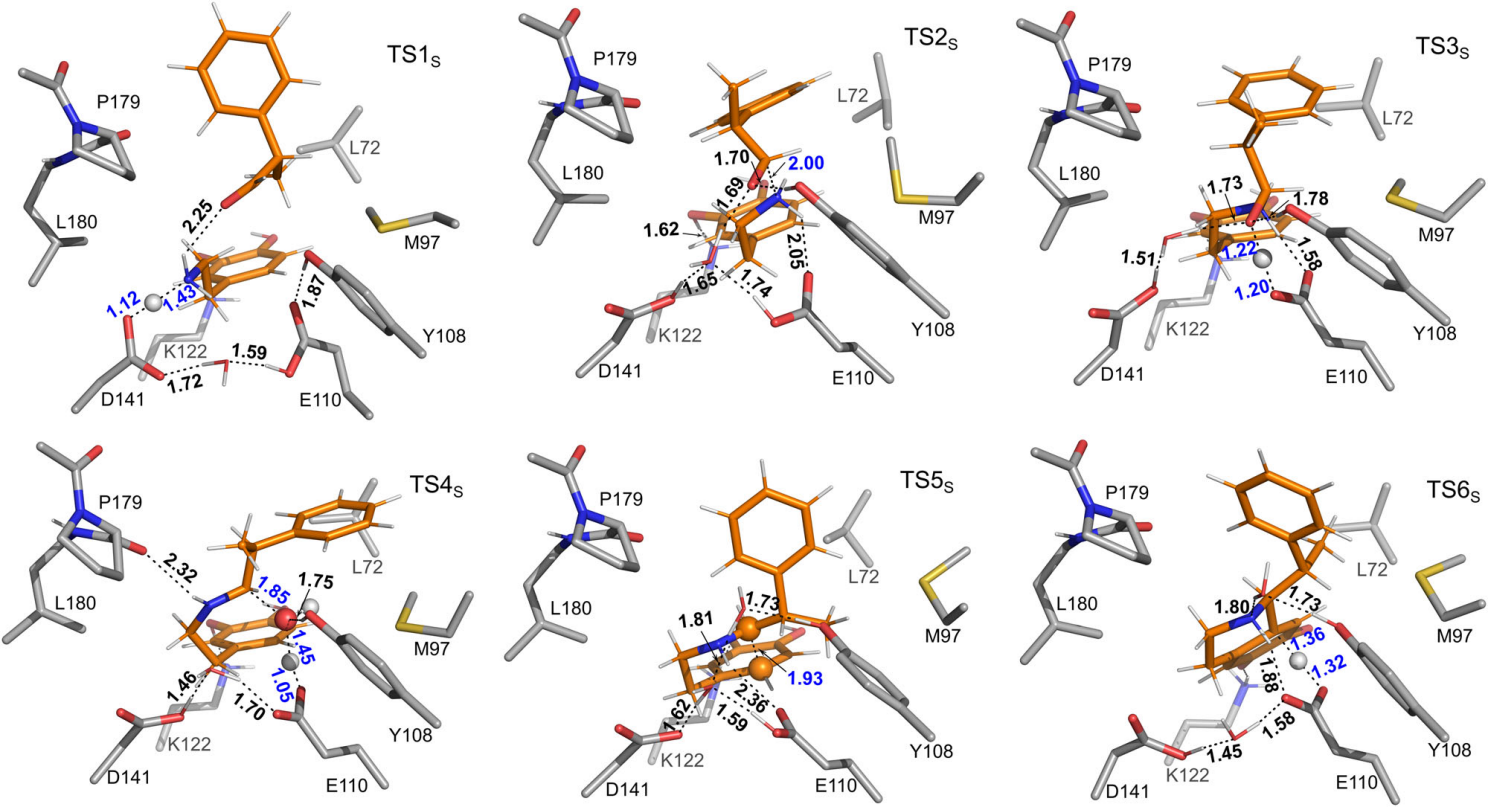
The optimized structures of the transition states involved in the *Tf*NCS-catalyzed PS reaction between dopamine and (*S*)-MPAA. Substrates are depicted as orange sticks. Selected distances are given in Å. For clarity, only a small part of the model is shown. Structures with the full model are given in Supplementary Fig. 7.

The *Tf*NCS-catalyzed condensation of dopamine and MPAA racemate exhibits high enantiospecificity in favor of the conversion of (*R*)-enantiomer, leading to the formation of (1*S*, 1’*R*)-product as the major product. To decipher the origins of the enantiospecificity, the pathway for the reaction of (*S*)-enantiomer, leading to the formation of (1*S*, 1’*S*)-product, was also studied (*S*-pathway). The calculated profile for the lowest-energy *S*-pathway is shown in Fig. 4, and the optimized structures of corresponding species are shown in Fig. 6 and Supplementary Figs. 7 and 8.

As shown in Fig. 4, in *S*-pathway, the proton transfer from the amino group of dopamine to the D141 is also easy with an energy barrier of 7.7 kcal mol^−1^ (TS1_S_ relative to E:S_S_). For the formation of C–N bond, the calculated energy barrier in the *S*-pathway (TS2_S_) is 2.7 kcal mol^−1^ lower than that of TS2_R_. By scrutinizing the optimized structures of TS2_R_ and TS2_S_ (Figs. 5 and 6), it was found that this difference can be ascribed to the fact that the P179 residue introduces steric interaction with the methyl group of the (*R*)-MPAA in TS2_R_ but not in TS2_S_. Due to this steric effect, the calculated energies of the species from Int3 to Int5 in the *R*-pathway (Int3_R_ to Int5_R_) are consistently higher than those in the *S*-pathway (Int3_S_ to Int5_S_).

The following dehydration step of *S*-pathway has a barrier of 19.1 kcal mol^−1^ (TS4_S_ relative to E:S_S_, Fig. 4), which 2.1 kcal mol^−1^ higher than the corresponding TS of *R*-pathway (TS4_R_). By analyzing the optimized structures, it can be identified that in TS4_R_ the –OH moiety of the substrate adjusts its hydrogen bonding interactions with the surrounding environment to accommodate the dehydration process and to form the *E*-configuration iminium (Supplementary Fig. 4). Additionally, the methyl group of the (*R*)-MPAA rotates away from P179 thus eliminating the previously mentioned steric interaction in TS4_R_. This trend remains in the following cyclization and deprotonation steps. The same as the *R*-pathway, the deprotonation of C5–H of the cyclized intermediate (TS6_S_) is calculated to be rate-limiting of the overall reaction for the *S*-pathway. The calculated barrier is 21.6 kcal mol^-1^ relative to E:S_S_, which is 1.5 kcal mol^−1^ higher than that of the *R*-pathway. According to the calculated energy profiles for the two pathways, this step is also the selectivity-determining step. The calculated difference between the barriers of *S*- and *R*-pathways is in good agreement with the experimental reports, 1.6 kcal mol^−1^ converted from the d. r. with a value of 94:6^24^.

By scrutinizing the geometries of the transition states of the rate-limiting steps of the two different pathways, the reasons for the obtained energy difference can be identified and the kinetic resolution of MPAA racemate can be rationalized. As shown in Supplementary Fig. 9, the methyl substituent of the substrate engages in steric interaction with M97 and L72 in TS6_R_ and TS6_S_, respectively, thus raising the corresponding energy barrier for the C5–H deprotonation step compared to the corresponding step in the natural reaction. Obviously, the hindrance with L72 in TS6_S_ is stronger than that with M97 in TS6_R_, as *R*-pathway corresponds to a lower barrier by 1.5 kcal mol^−1^. This trend is more obvious in the following steps, which are 9.3 and 9.9 kcal mol^−1^ in favor of the Int10_R_ and E:P_R_ compared to Int10_S_ and E:P_S,_ respectively. This is in fact consistent with the experimental mutagenesis analysis, for which the replacement of M97 by a smaller valine residue resulted in higher (*R*)-enantiospecificity^24^.

## Conclusions

In the present study, the detailed mechanisms of the *Tf*NCS-catalyzed PS reactions of dopamine with both enantiomers of α-methyl-phenylacetaldehyde (MPAA) and the factors contributing to the kinetic resolution of MPAA racemate are studied by using quantum chemical calculations. The calculations reveal that the reaction mechanisms are consistent with that of the natural substrates proposed in the previous study. The deprotonation of C5−H of the cyclized intermediates is the rate-limiting step for the reaction of both enantiomers of MPAA, with calculated barriers are 20.1 and 21.6 kcal mol^−1^ for (*R*)- and (*S*)-MPAA, respectively.

The current calculations perfectly reproduce the experimentally measured diastereoisomeric ratio (d. r.) value of the *Tf*NCS-catalyzed kinetic resolution of MPAA racemate (1.5 kcal mol^-1^ from the calculations vs. 1.6 kcal mol^−1^ converted from the experimental data). By scrutinizing the optimized geometries of selectivity-determining transition states, it is revealed that the steric effects between the α-methyl group of the aldehyde substrate and the L72 and M97 residues are the key factors contributing to the kinetic resolution of MPAA racemate.

We believe that the detailed information about the mechanisms and the factors contributing to the enantiospecificity of MPAA racemate will be helpful to rationally design variants of *Tf*NCS with improved reactivity and selectivity for a wider scope of substrates to generate new tetrahydroisoquinoline skeletons. Indeed, in a recent Quantum Mechanics/Molecular Mechanics (QM/MM) study on the NCS-catalyzed reaction of the natural substrates, mutants with enhanced rate were identified through the rational transition-state macrodipole stabilization method^31^. The insights provided by the current study can also be extended to other NCS enzymes and other members of the Pictet–Spenglerase family.

From the technical point of view, the present study demonstrates again that the quantum chemical cluster approach is powerful in examining enzyme properties with small energy differences. The high accuracy in energy calculations using this methodology can be attributed, in part, to the effective cancellation of systematic errors between the transition states of very high similarity.

## Method

### Technical details

The quantum chemical cluster approach was chosen for investigating the reaction mechanism and exploring the factors controlling the stereospecificity of *Tf*NCS toward the non-natural aldehyde substrate α-methyl-phenylacetaldehyde. This method has been proven to be powerful in studying the mechanism of enzymatic reactions^11,32–35^, and elucidating the origins of selectivities^29,36–38^. All calculations in this work were conducted using Gaussian16 C.01 package with the B3LYP-D3(BJ) density functional method^39–43^. Geometry optimizations were performed with 6-31G (d,p) basis set for all atoms. Based on the optimized structures, at the same level of theory of geometry optimization, the single-point solvation energies using solvation model based on density (SMD) with *ɛ* = 4.0^44^ and frequency calculations were performed to estimate the effects of enzyme environment and to obtain zero-point energies (ZPE), respectively. The transition states were validated by the imaginary normal modes of vibrations. For more accurate electronic energies, the single-point energy calculations were performed with the larger basis set 6-311 + G(2d,2p). The presented energies in this work are the electronic energies at B3LYP-D3(BJ)/6-311 + G(2d,2p) level corrected for the solvation effect and the zero-point energy correction. The absolute energies and energy corrections of the species involved in the reaction pathways are summarized in Supplementary Table 1.

### Active site model

Based on the recently solved crystal structure of *Tf*NCS in complex with an intermediate analog (PDB code: 5NON)^45^, the cluster model of the active site was designed to investigate the reaction mechanism and the stereospecificity of *Tf*NCS toward the (*R*/*S*)-α-methyl-phenylacetaldehyde. We constructed two models for the enzyme-substrates complexes, which consist of dopamine and (*R*)-α-methyl-phenylacetaldehyde (called *R*-model) or (*S*)-α-methyl-phenylacetaldehyde (called *S*-model). The intermediate analog in the crystal structure was manually replaced by the corresponding substrate. These models include residues proposed to be general acid/base groups in the reaction (E110, K122, and D141), key residues controlling selectivity based on experimental findings (L76, A79, F80, M97, Y108), and other residues constituting the active site (Y63, S64, W65, P66, L68, L72, L75, L95, F99, F112, V124, M126, Y139, I143, T159, G178, P179, L180, M183), along with two crystallographic waters. Amino acids were truncated, and hydrogen atoms were added to maintain saturation of the truncated carbon atoms. To avoid unrealistic movements, the truncated carbons were held fixed in their crystallographic positions during the geometry optimizations (see Supplementary Fig. 1 for the fixed atoms). The coordinate locking procedure gave rise to several small imaginary frequencies, typically within 50i cm^−1^. These frequencies do not contribute significantly to the ZPE and can thus be safely disregarded. The final active sites of both models consist of 396 atoms and the overall charge is 0.

The protonation states of the ionizable residues within the active site model are determined based on the previous experimental and computational studies on the NCS-catalyzed reactions^28,29^. Specifically, the established role of K122 in deprotonating the substrate’s phenolic hydroxyl group emphasizes the necessity for this residue to be in its neutral form^28,29^. K122 is thus modeled in its neutral form in the present study. The previous theoretical study showed that the pathway in which both E110 and D141 are ionized has very high energies^29^. Therefore, one of them is neutral and the other one is ionized in the reaction. The current calculation results on the non-natural substrates showed that the enzyme-substrates complex with D141 in the protonated state while E110 in the deprotonated state has the lowest energy (Fig. 2). Furthermore, as reported in the previous study^28^, the predicted p*K*_a_ values of K122 is much lower than the typical value, while the p*K*_a_ values of D141 and E110 are rather higher than usual, indicating that the p*K*_a_ of these residues are greatly influenced by the active site microenvironment and the energetic penalties associated with switching the protonation states of these residues are relatively modest.

### Supplementary information

SUPPLEMENTARY INFORMATION

Description of Additional Supplementary Files

Supplementary Data 1

## Data Availability

The authors declare that the coordinates of the optimized structures of all the species involved in the lowest-energy pathways and their corresponding energies are available within the paper and its Supplementary Data 1. The coordinates and energies of other considered structures are available from the corresponding authors upon reasonable request. The following calculation results are provided in the Supplementary Materials: optimized structures for the enzyme-substrates complexes; optimized structures of the intermediates and transition states; superposition of (*R*)-iminium ion intermediate (Int6_R_) with the co-crystallized structure; schematic illustration of the selectivity-determining transition states; absolute energies and energy corrections.
