# Supplementary material for: Computational mechanistic investigation of the kinetic resolution of α-methyl-phenylacetaldehyde by norcoclaurine synthase
Source: Commun Chem. 2024 Mar 27;7:64. doi: 10.1038/s42004-024-01146-x (PMC10973476; doi:10.1038/s42004-024-01146-x)
Supplement: Supplementary file 1 — SUPPLEMENTARY INFORMATION [file 42004_2024_1146_MOESM1_ESM.pdf]

SUPPLEMENTARY INFORMATION

# Theoretical mechanistic investigation of the kinetic resolution of α-methyl-phenylacetaldehyde by norcoclaurine synthase

Shiqing Zhang[a,b], Chenghua Zhang[a,c], Aijing Guo[a,d], Baoyan Liu[a,b], Hao Su[a,b,d*], Xiang Sheng[a,b,d*]

[a]Tianjin Institute of Industrial Biotechnology, Chinese Academy of Sciences, Tianjin 300308, P. R. China

[b]National Center of Technology Innovation for Synthetic Biology, National Engineering Research Center of Industrial Enzymes and Key Laboratory of Engineering Biology for Low-Carbon Manufacturing, Tianjin 300308, P.R. China

[c]School of Pharmacy, North Sichuan Medical College, Nanchong 637100, P. R. China

[d]University of Chinese Academy of Sciences, Beijing 100049, China

Corresponding authors:

Hao Su: suhao@tib.cas.cn

Xiang Sheng: shengx@tib.cas.cn

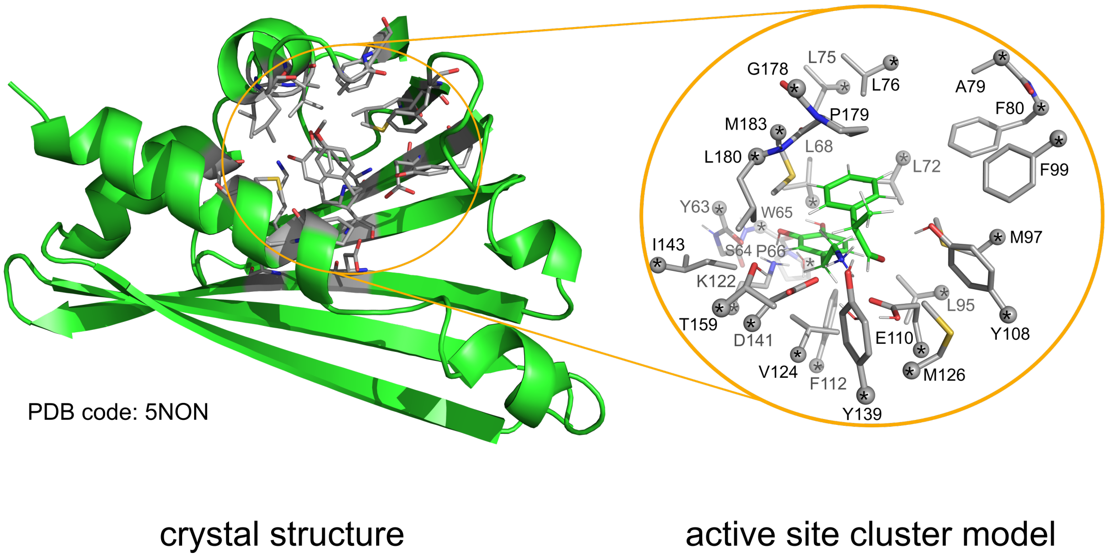


Supplementary Figure 1. The designed active site cluster model on the basis of the crystal structure of *Tf*NCS in complex with an intermediate analogue (PDB code: 5NON). The intermediate analogue in the crystal structure was replaced by the substrates manually in the construction of the cluster model. Atoms fixed during geometry optimization are marked with stars.

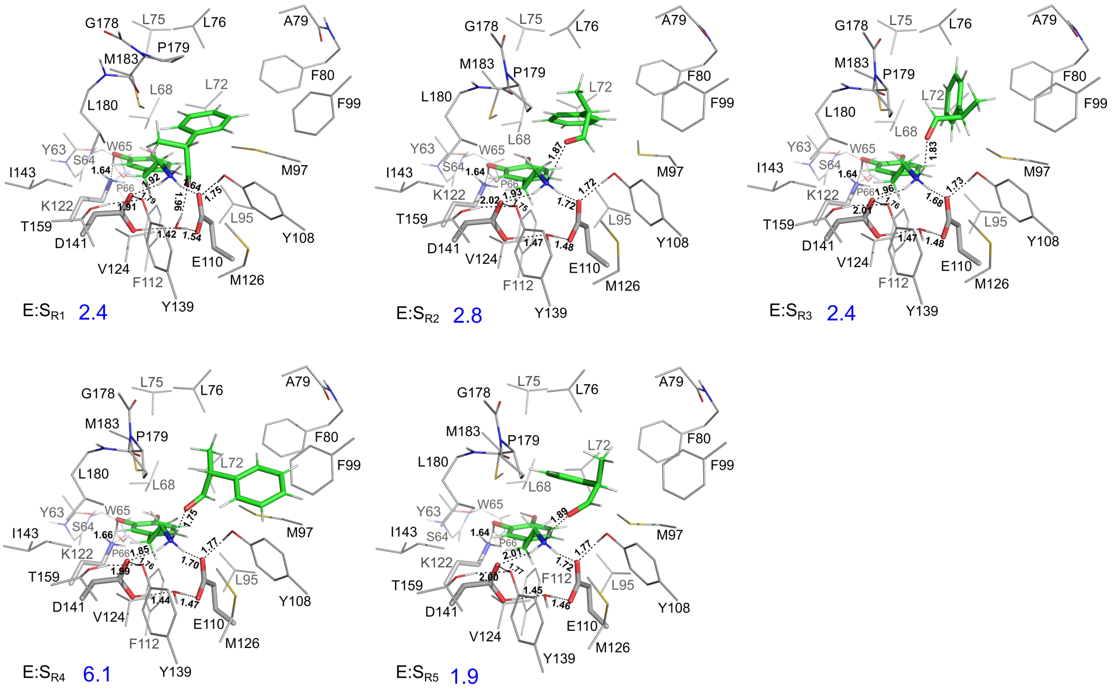


Supplementary Figure 2. Optimized structures of the enzyme-substrates complexes in the *R*-model. The substrates dopamine and (*R*)-MPAA are shown in green stick. The energies relative to that of **E:S$_S$** are shown in kcal/mol. The distances are given in Å. Structures were optimized at the level of B3LYP-D3BJ/6-31g(d,p).

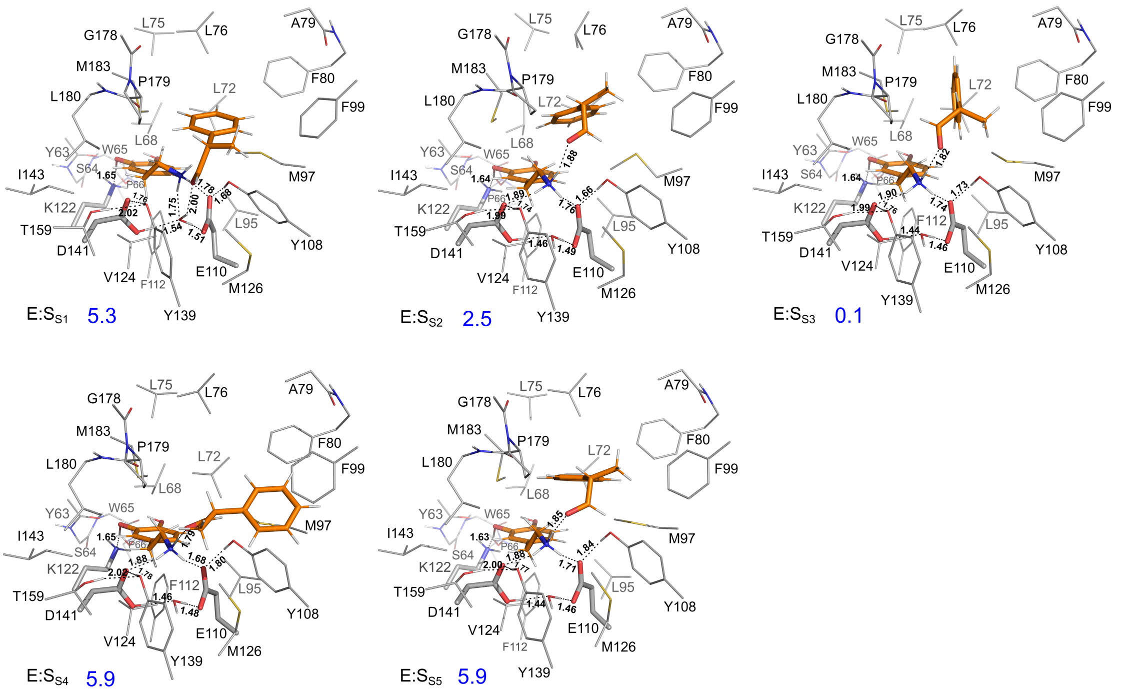


Supplementary Figure 3. Optimized structures of the enzyme-substrates complexes in the *S*-model. The substrates dopamine and (*S*)-MPAA are shown in orange stick. The energies relative to that of **E:S$_S$** are shown in kcal/mol. The distances are given in Å. Structures were optimized at the level of B3LYP-D3BJ/6-31g(d,p).

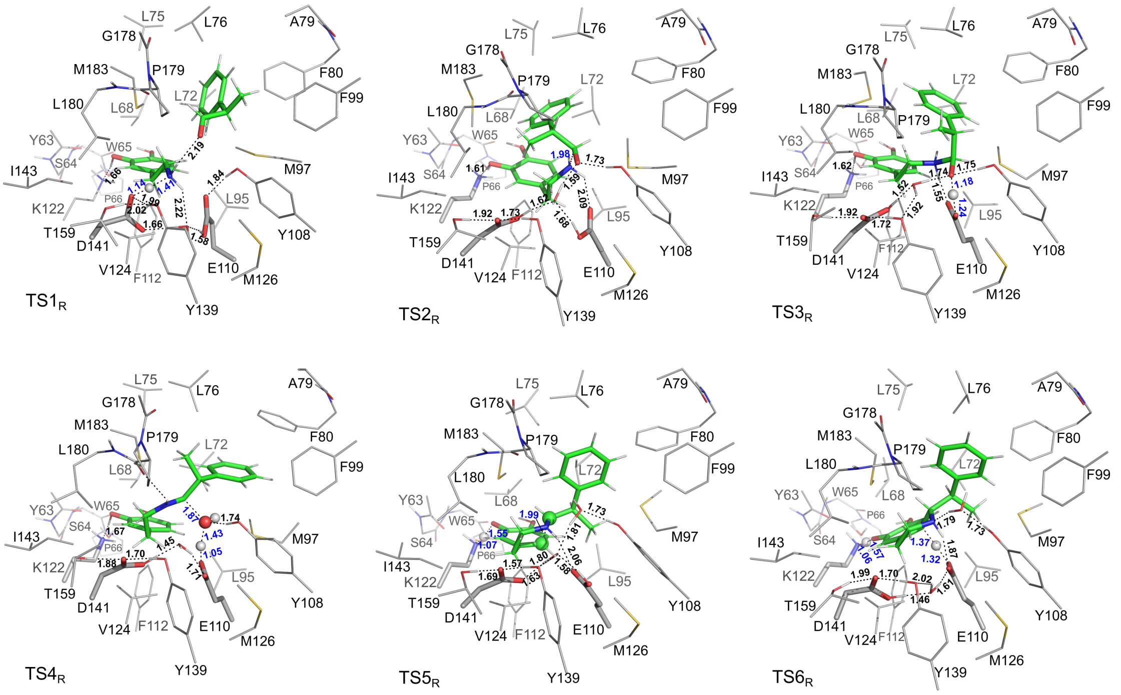


Supplementary Figure 4. Optimized structures of the transition states involved in the *R*-pathway. Substrates are depicted as green sticks. Key atoms involved in the bond formation or cleavage are shown as ball models. Dashed lines represent hydrogen bonds. Key distances are highlighted in blue, while other H-bond distances are shown in black. The distances are given in Å. Structures were optimized at the level of B3LYP-D3BJ/6-31g(d,p).

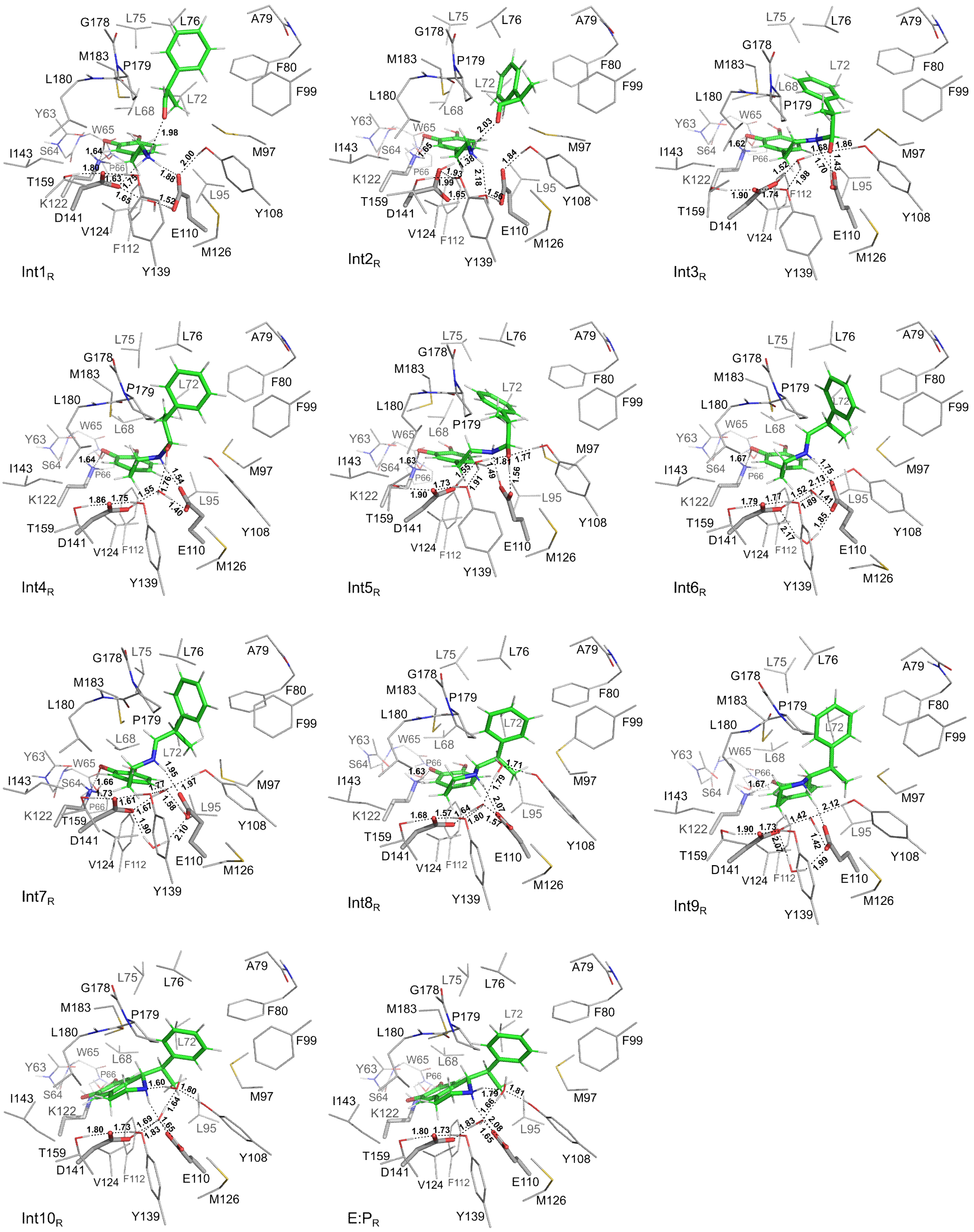


Supplementary Figure 5. Optimized structures of the intermediates and product involved in the *R*-pathway. Substrates are depicted as green sticks. Dashed lines represent hydrogen bonds. Distances are given in Å. Structures were optimized at the level of B3LYP-D3BJ/6-31g(d,p).

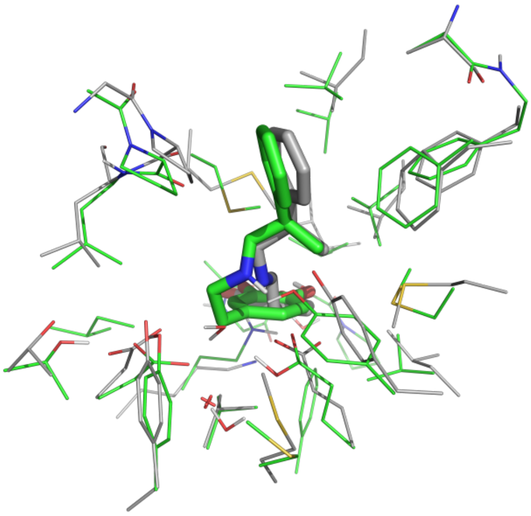

Supplementary Figure 6. The superposition of the optimized structure of the (*R*)-iminium intermediate (**Int6$_R$**, green) with the co-crystallized structure of *Tf*NCS with the (*R*)-iminium mimic (PDB ID: 6RP3, gray). The substrates are shown in sticks.

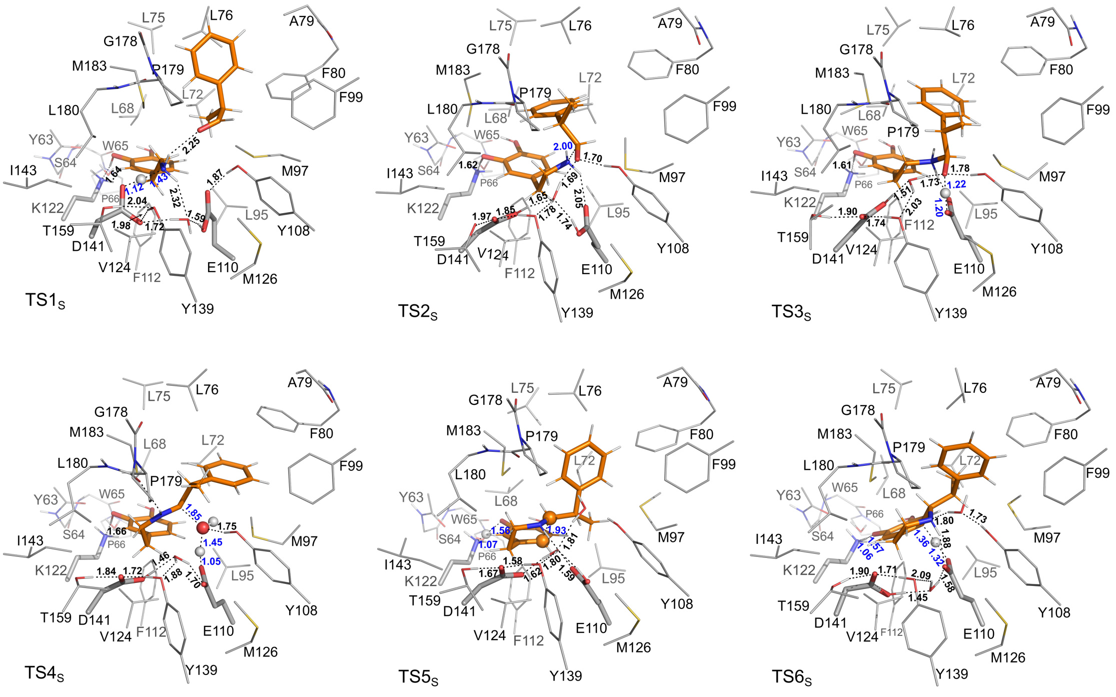


Supplementary Figure 7. Optimized structures of the transition states involved in the *S*-pathway. Substrates are depicted as orange sticks. Key atoms involved in the bond formation or cleavage are shown as ball models. Dashed lines represent hydrogen bonds. Key distances are highlighted in blue, while other H-bond distances are shown in black. Distances are given in Å. Structures were optimized at the level of B3LYP-D3BJ/6-31g(d,p).

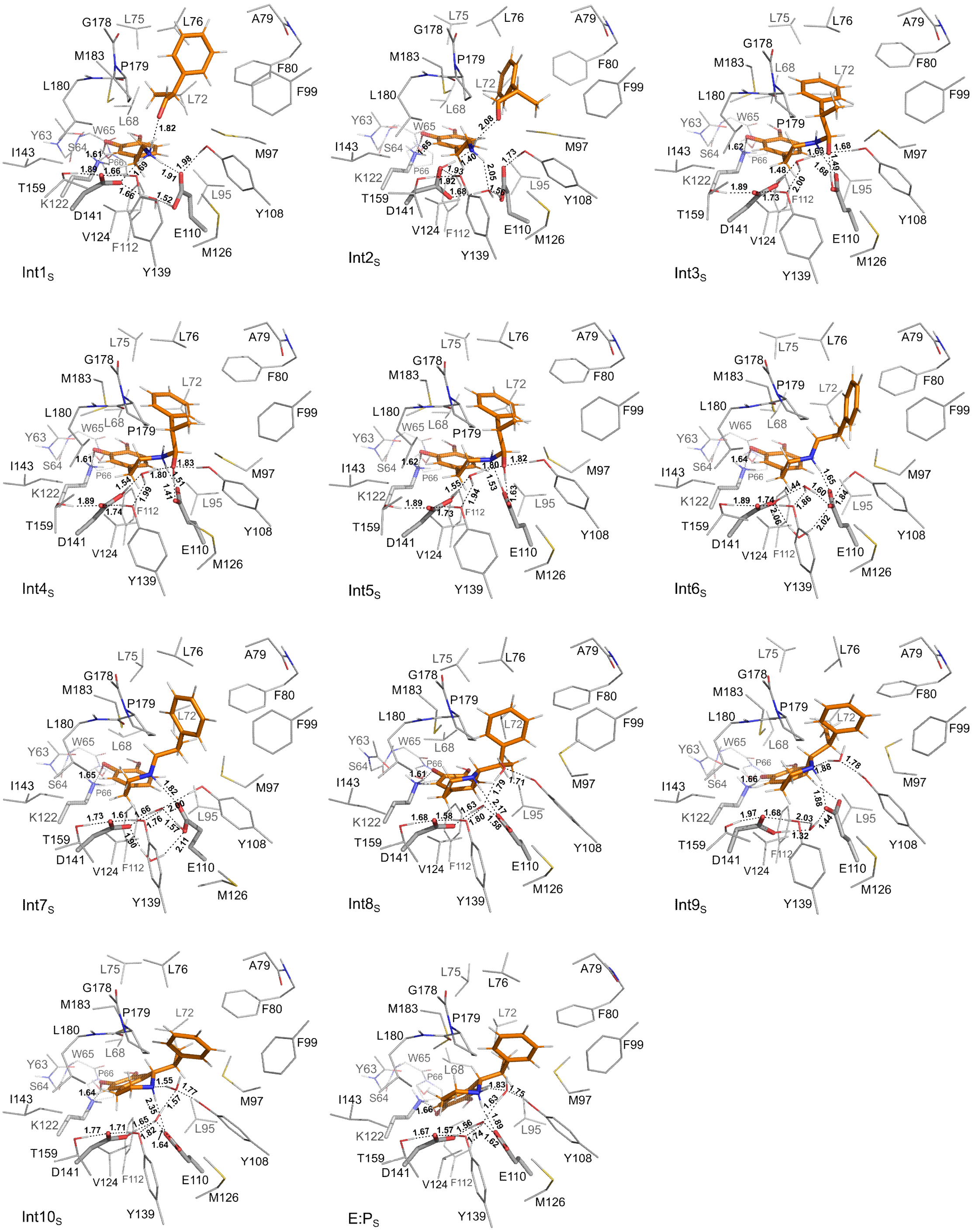


Supplementary Figure 8. Optimized structures of the intermediates and product involved in the *S*-pathway. Substrates are depicted as orange sticks. Dashed lines represent hydrogen bonds. Distances are given in Å. Structures were optimized at the level of B3LYP-D3BJ/6-31g(d,p).

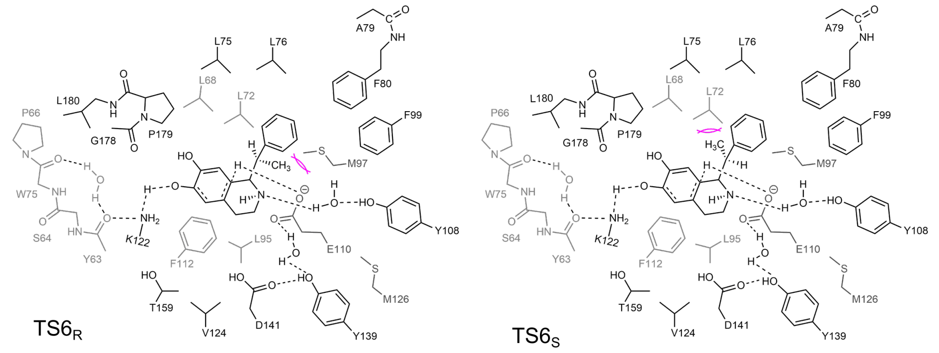

Supplementary Figure 9. Schematic illustration of the transition states for the selectivity-determining deprotonation of C5−H resulting in the formation of (*R*)-product (**TS6$_{\mathbf{R}}$**) and (*S*)-product (**TS6$_{\mathbf{S}}$**) on the base of the optimized structures.

Supplementary Table 1. Absolute energies and energy corrections.

| | $E_{6\text{-}31G\,(d,\,p)}$ [a] (au) | E-SMD$_{6\text{-}31G\,(d,\,p)}$ [b] (au) | $E_{6\text{-}311+G(2d,\,2p)}$ [c] (au) | ZPE$_{6\text{-}31G\,(d,\,p)}$ [d] (au) | $E_{total}$ [e] (au) | ΔE [f] (kcal/mol) |
|---|---|---|---|---|---|---|
| E:S$_R$ | -8047.25005 | -8047.263079 | -8049.2157 | 3.518187 | -8045.71051 | +0.1 |
| Int1$_R$ | -8047.24062 | -8047.25558 | -8049.2111 | 3.518292 | -8045.70774 | +1.9 |
| TS1$_R$ | -8047.22911 | -8047.246378 | -8049.1971 | 3.51338 | -8045.70101 | +6.1 |
| Int2$_R$ | -8047.23174 | -8047.247629 | -8049.1981 | 3.514977 | -8045.69897 | +7.4 |
| TS2$_R$ | -8047.2192 | -8047.236214 | -8049.1852 | 3.519944 | -8045.68229 | +17.8 |
| Int3$_R$ | -8047.24456 | -8047.257992 | -8049.2074 | 3.522149 | -8045.69868 | +7.6 |
| TS3$_R$ | -8047.24000 | -8047.254953 | -8049.2025 | 3.517592 | -8045.69984 | +6.8 |
| Int4$_R$ | -8047.24603 | -8047.261019 | -8049.2099 | 3.521645 | -8045.70323 | +4.7 |
| Int5$_R$ | -8047.23861 | -8047.253021 | -8049.2018 | 3.520831 | -8045.69543 | +9.6 |
| TS4$_R$ | -8047.22085 | -8047.234939 | -8049.1859 | 3.516345 | -8045.68368 | +17.0 |
| Int6$_R$ | -8047.23227 | -8047.248726 | -8049.2009 | 3.517309 | -8045.70006 | +6.7 |
| Int7$_R$ | -8047.24373 | -8047.259595 | -8049.2109 | 3.519621 | -8045.70714 | +2.2 |
| TS5$_R$ | -8047.23649 | -8047.256606 | -8049.2007 | 3.522227 | -8045.69855 | +7.6 |
| Int8$_R$ | -8047.24151 | -8047.264468 | -8049.2053 | 3.523739 | -8045.70456 | +3.9 |
| Int9$_R$ | -8047.21738 | -8047.250184 | -8049.1839 | 3.518955 | -8045.69773 | +8.1 |
| TS6$_R$ | -8047.21242 | -8047.231028 | -8049.1763 | 3.516163 | -8045.67875 | +20.1 |
| Int10$_R$ | -8047.25227 | -8047.265592 | -8049.2149 | 3.524432 | -8045.70379 | +4.3 |
| E:P$_R$ | -8047.26236 | -8047.278265 | -8049.2251 | 3.525969 | -8045.715 | -2.7 |
| E:S$_S$ | -8047.24961 | -8047.262896 | -8049.2147 | 3.517222 | -8045.71072 | 0.0 |
| Int1$_S$ | -8047.240251 | -8047.255194 | -8049.212502 | 3.517473 | -8045.709972 | +0.5 |
| TS1$_S$ | -8047.23232 | -8047.244989 | -8049.1997 | 3.514023 | -8045.69839 | +7.7 |
| Int2$_S$ | -8047.23685 | -8047.250125 | -8049.2026 | 3.514387 | -8045.70146 | +5.8 |
| TS2$_S$ | -8047.21741 | -8047.232585 | -8049.188 | 3.516534 | -8045.68665 | +15.1 |
| Int3$_S$ | -8047.25322 | -8047.26762 | -8049.2172 | 3.521268 | -8045.71037 | +0.2 |
| TS3$_S$ | -8047.24311 | -8047.257555 | -8049.2077 | 3.517123 | -8045.70503 | +3.6 |
| Int4$_S$ | -8047.24643 | -8047.262791 | -8049.2118 | 3.520639 | -8045.70752 | +2.0 |
| Int5$_S$ | -8047.24668 | -8047.26029 | -8049.2115 | 3.520706 | -8045.7044 | +4.0 |
| TS4$_S$ | -8047.22115 | -8047.234487 | -8049.1837 | 3.516831 | -8045.68022 | +19.1 |
| Int6$_S$ | -8047.22991 | -8047.248653 | -8049.2011 | 3.516933 | -8045.70295 | +4.9 |

| | | | | | | |
|---|---|---|---|---|---|---|
| $Int7_S$ | -8047.24762 | -8047.262981 | -8049.2146 | 3.519479 | -8045.7105 | +0.1 |
| $TS5_S$ | -8047.2331 | -8047.253035 | -8049.1956 | 3.522727 | -8045.69282 | +11.2 |
| $Int8_S$ | -8047.23392 | -8047.25605 | -8049.1981 | 3.523719 | -8045.69649 | +8.9 |
| $Int9_S$ | -8047.21768 | -8047.243058 | -8049.1825 | 3.519988 | -8045.68787 | +14.3 |
| $TS6_S$ | -8047.20817 | -8047.227086 | -8049.1734 | 3.51597 | -8045.67632 | +21.6 |
| $Int10_S$ | -8047.23399 | -8047.246483 | -8049.1978 | 3.521221 | -8045.68904 | +13.6 |
| $E:P_S$ | -8047.25073 | -8047.261908 | -8049.2126 | 3.524453 | -8045.69927 | +7.2 |
| $E:S_{R1}$ | -8047.24706 | -8047.261482 | -8049.2103 | 3.517868 | -8045.70688 | +2.4 |
| $E:S_{R2}$ | -8047.24997 | -8047.263703 | -8049.2125 | 3.519894 | -8045.7063 | +2.8 |
| $E:S_{R3}$ | -8047.24991 | -8047.262607 | -8049.2122 | 3.518019 | -8045.70687 | +2.4 |
| $E:S_{R4}$ | -8047.2376 | -8047.251575 | -8049.2039 | 3.516868 | -8045.70101 | +6.1 |
| $E:S_{R5}$ | -8047.24698 | -8047.26137 | -8049.2109 | 3.517546 | -8045.70777 | +1.9 |
| $E:S_{S1}$ | -8047.23812 | -8047.254583 | -8049.2041 | 3.518275 | -8045.70228 | +5.3 |
| $E:S_{S2}$ | -8047.25257 | -8047.26593 | -8049.2143 | 3.52094 | -8045.70673 | +2.5 |
| $E:S_{S3}$ | -8047.24373 | -8047.26172 | -8049.2099 | 3.517261 | -8045.71064 | +0.1 |
| $E:S_{S4}$ | -8047.23188 | -8047.249123 | -8049.2002 | 3.516175 | -8045.70129 | +5.9 |
| $E:S_{S5}$ | -8047.24260 | -8047.256169 | -8049.2056 | 3.517839 | -8045.70138 | +5.9 |

[a] $\mathbf{E_{6\text{-}31G(d,p)}}$: the energies of the optimized structures at the level of B3LYP-D3(BJ)/6-31G (d, p).

[b] $\mathbf{E\text{-}SMD_{6\text{-}31G(d,p)}}$: the single-point SMD solvation energies at the level of B3LYP-D3(BJ)/6-31G (d, p).

[c] $\mathbf{E_{6\text{-}311+G(2d,2p)}}$: the single-point energies at the level of B3LYP-D3(BJ)/6-311+G(2d, 2p).

[d] $\mathbf{ZPE_{6\text{-}31G(d,p)}}$: the zero-point energies at the level of B3LYP-D3(BJ)/6-31G (d, p).

[e] $\mathbf{E_{total} = E\text{-}SMD_{6\text{-}31G(d,p)} - E_{6\text{-}31G(d,p)} + E_{6\text{-}311+G(2d,2p)} + ZPE_{6\text{-}31G\ (d,p)}}$

[f] $\Delta$ **E=($\mathbf{E_{total}}$(X) - $\mathbf{E_{total}}$(E:$\mathbf{S_S}$))**, where $\mathbf{E_{total}}$**(X)** is total energy of the involved species in the reaction pathways and $\mathbf{E_{total}}$(**E:$\mathbf{S_S}$**) is the total energy of the **E:$\mathbf{S_S}$**.
