# Supplementary material for: Computational mechanistic investigation of the kinetic resolution of α-methyl-phenylacetaldehyde by norcoclaurine synthase
Source: Commun Chem. 2024 Mar 27;7:64. doi: 10.1038/s42004-024-01146-x (PMC10973476; doi:10.1038/s42004-024-01146-x)
Supplement: Supplementary file 2 — Description of Additional Supplementary Files [file 42004_2024_1146_MOESM2_ESM.pdf]

Description of Additional Supplementary Files

File name: Supplementary Data 1

Description: Cartesian coordinates and absolute energies of the optimized structures in the lowest-energy pathways.
