# Supplementary material for: Computational mechanistic investigation of the kinetic resolution of α-methyl-phenylacetaldehyde by norcoclaurine synthase
Source: Commun Chem. 2024 Mar 27;7:64. doi: 10.1038/s42004-024-01146-x (PMC10973476; doi:10.1038/s42004-024-01146-x)
Supplement: Supplementary file 3 — Supplementary Data 1 [file 42004_2024_1146_MOESM3_ESM.zip › Supplementary Data 1.docx]

**Cartesian coordinates and absolute energies of the optimized structures in the lowest-energy pathways:**

The numbers in the second column indicate atom fixation, where -1 means that the corresponding atom was fixed in the geometry optimization while 0 denotes no constraint imposed on that particular atom. The absolute energies presented in the parentheses are the electronic energies at the level of B3LYP-D3(BJ)/ 6-311+G(2d, 2p) corrected for the solvation effect and the zero-point energy correction (in a.u.).

**E:SR** (-8045.70978)

C -1 7.280822 0.460519 1.970018

C 0 5.931930 0.651351 2.673803

C 0 6.070496 0.535869 4.195753

C 0 5.293566 1.992714 2.290942

H 0 7.163224 0.461689 0.884197

H 0 7.978571 1.266349 2.228249

H 0 5.258142 -0.149207 2.338167

H 0 6.728224 1.322252 4.587688

H 0 5.099383 0.634353 4.691115

H 0 6.498496 -0.429703 4.485879

H 0 4.293450 2.102494 2.725001

H 0 5.903548 2.829724 2.652654

H 0 5.218564 2.115643 1.205721

C -1 2.867947 -2.653793 6.140232

C 0 3.112995 -1.962083 4.791592

C 0 2.953944 -2.939143 3.621024

C 0 2.191655 -0.751444 4.603481

H 0 1.845085 -3.045176 6.196589

H 0 3.552668 -3.495855 6.288065

H 0 4.148912 -1.596967 4.784862

H 0 1.934666 -3.341454 3.592944

H 0 3.141612 -2.439112 2.665508

H 0 3.642595 -3.786878 3.706252

H 0 1.138255 -1.059088 4.611798

H 0 2.329259 -0.016878 5.403311

H 0 2.385663 -0.257608 3.646756

C -1 0.466644 0.416873 10.770601

C 0 0.895156 0.896827 9.376207

C 0 0.481105 -0.105275 8.291771

C 0 0.329961 2.288917 9.069971

H 0 -0.624160 0.323184 10.824358

H 0 0.896081 -0.564222 11.001286

H 0 1.991551 0.969551 9.371642

H 0 -0.605192 -0.233167 8.271125

H 0 0.796925 0.229654 7.298192

H 0 0.930864 -1.087581 8.468012

H 0 -0.764695 2.261001 9.040723

H 0 0.630997 3.020687 9.827835

H 0 0.679485 2.650588 8.096866

C -1 -3.085977 -4.132881 12.675565

C 0 -3.244782 -5.421289 11.853350

O 0 -4.265734 -5.629427 11.203179

C 0 -2.974350 -2.924074 11.737828

H 0 -3.977797 -4.044884 13.302590

H 0 -2.094853 -3.007095 11.091336

H 0 -3.859472 -2.864343 11.100382

H 0 -2.892724 -1.995124 12.309658

N 0 -2.192805 -6.298106 11.874802

C -1 -2.114137 -7.480170 11.012458

C 0 -0.983396 -7.381510 9.976878

C 0 -1.021788 -6.128664 9.128957

C 0 -2.069114 -5.883337 8.229642

C 0 0.007735 -5.186144 9.226506

C 0 -2.066944 -4.742298 7.426642

C 0 0.010645 -4.037844 8.433862

C 0 -1.022350 -3.818241 7.523543

H 0 -3.087406 -7.546335 10.522292

H 0 -1.040117 -8.273593 9.340201

H 0 -0.016015 -7.435213 10.491402

H 0 -2.897722 -6.581636 8.154954

H 0 -1.358300 -6.018220 12.368673

H 0 0.825142 -5.361625 9.921787

H 0 -2.876968 -4.576401 6.724910

H 0 0.822965 -3.323896 8.518859

H 0 -1.022612 -2.926535 6.906100

C -1 0.152443 -6.400880 -0.187664

C 0 1.301918 -5.453555 -0.561879

C 0 1.242408 -5.055951 -2.041380

C 0 1.305915 -4.208514 0.333146

H 0 0.207006 -6.695564 0.866479

H 0 -0.817441 -5.916448 -0.346504

H 0 2.247114 -5.990330 -0.395631

H 0 0.331773 -4.485162 -2.250839

H 0 2.091197 -4.423728 -2.319289

H 0 1.241896 -5.936371 -2.693371

H 0 0.353870 -3.671220 0.252992

H 0 1.453717 -4.473819 1.384929

H 0 2.103365 -3.514168 0.048592

C -1 -3.596721 -6.933807 3.872113

C 0 -2.293956 -6.138595 3.931037

S 0 -2.620400 -4.354727 3.640078

C 0 -0.900173 -3.750884 3.633215

H 0 -4.288281 -6.616801 4.657923

H 0 -4.098931 -6.797442 2.909587

H 0 -1.593693 -6.483227 3.161867

H 0 -1.812490 -6.251152 4.908208

H 0 -0.315919 -4.262694 2.865158

H 0 -0.925874 -2.686427 3.392667

H 0 -0.427703 -3.888764 4.609643

C -1 -8.593009 -6.748012 0.713019

C 0 -7.632713 -5.676003 1.166946

C 0 -6.351099 -5.563771 0.619857

C 0 -8.008122 -4.734005 2.136423

C 0 -5.475603 -4.547852 1.004853

C 0 -7.152129 -3.708632 2.527970

C 0 -5.881705 -3.596381 1.948247

O 0 -5.085862 -2.560542 2.327100

H 0 -8.984906 -7.325369 1.557949

H 0 -9.453292 -6.303898 0.198151

H 0 -6.022895 -6.284578 -0.125030

H 0 -8.998133 -4.793685 2.579772

H 0 -4.478921 -4.494439 0.584892

H 0 -7.454715 -2.976241 3.269056

H 0 -4.294786 -2.556241 1.745034

C -1 -3.717827 -4.902604 -2.923979

C 0 -2.563827 -3.907872 -2.796290

C 0 -2.346274 -3.397969 -1.370673

C 0 -3.420270 -2.461048 -0.831107

O 0 -4.348905 -2.107050 -1.605770

O 0 -3.274656 -2.075664 0.377503

H 0 -4.655352 -4.443287 -2.600092

H 0 -2.747576 -3.051385 -3.450908

H 0 -1.637434 -4.379762 -3.144354

H 0 -1.403940 -2.840652 -1.296865

H 0 -2.246889 -4.226237 -0.658211

C -1 2.462903 -1.588928 -6.455091

C 0 2.733006 -1.417743 -4.974614

C 0 1.690159 -1.520681 -4.043517

C 0 4.024646 -1.173173 -4.490847

C 0 1.924800 -1.381122 -2.675209

C 0 4.273004 -1.050015 -3.120953

C 0 3.221121 -1.152969 -2.206597

H 0 3.252393 -1.133417 -7.059491

H 0 2.410198 -2.650174 -6.726575

H 0 0.681219 -1.708746 -4.397664

H 0 4.847613 -1.078526 -5.195042

H 0 1.099421 -1.441810 -1.973133

H 0 5.281770 -0.846693 -2.770258

H 0 3.399022 -1.039052 -1.141431

C -1 2.557012 4.064073 -4.609265

C 0 2.999487 3.745179 -3.177216

C 0 3.629939 2.358331 -3.057450

N 0 4.104529 2.104570 -1.682322

H 0 3.409891 4.056855 -5.297135

H 0 1.834011 3.325503 -4.968158

H 0 2.150015 3.806233 -2.486958

H 0 3.723634 4.501294 -2.839558

H 0 4.444634 2.252509 -3.789335

H 0 2.890658 1.588954 -3.298070

H 0 4.861108 2.746103 -1.445903

H 0 4.518947 1.175348 -1.637279

C -1 -2.421901 0.949306 -5.153816

C 0 -1.750105 1.057398 -3.779005

C 0 -1.751089 -0.305180 -3.081246

C 0 -0.335015 1.638747 -3.878550

H 0 -1.877126 0.246860 -5.796618

H 0 -2.351345 1.743472 -3.168240

H 0 -2.770351 -0.660001 -2.907669

H 0 -1.245313 -1.054411 -3.699999

H 0 -1.231834 -0.264612 -2.119656

H 0 0.312928 0.999422 -4.489233

H 0 -0.350055 2.636470 -4.332306

H 0 0.124713 1.722517 -2.888228

C -1 -8.784044 -2.890610 -3.161080

C 0 -8.923245 -3.820011 -1.957358

S 0 -9.990012 -3.165411 -0.616845

C 0 -8.912077 -1.833109 0.005349

H 0 -9.760488 -2.668071 -3.598332

H 0 -8.321536 -1.939210 -2.885926

H 0 -7.949718 -4.067209 -1.520240

H 0 -9.392975 -4.763721 -2.251123

H 0 -8.867711 -1.004001 -0.704298

H 0 -9.356692 -1.460426 0.929834

H 0 -7.918123 -2.224016 0.236929

C -1 -11.413946 -0.217146 -4.324783

C 0 -10.549735 0.435585 -3.272453

C 0 -9.217864 0.769945 -3.526345

C 0 -11.062765 0.743164 -2.003495

C 0 -8.424396 1.413092 -2.573510

C 0 -10.288770 1.382753 -1.040610

C 0 -8.963775 1.735725 -1.324380

O 0 -8.279238 2.433850 -0.367962

H 0 -11.890276 -1.125166 -3.939532

H 0 -12.214328 0.454443 -4.656458

H 0 -8.785219 0.538851 -4.495881

H 0 -12.088451 0.475915 -1.764623

H 0 -7.408772 1.688258 -2.824537

H 0 -10.693307 1.622470 -0.063039

H 0 -7.314256 2.458593 -0.566230

C -1 -5.820121 4.559280 -3.594175

C 0 -4.653898 3.992974 -2.757687

C 0 -5.136487 2.784569 -2.001715

O 0 -5.675930 2.867860 -0.893774

O 0 -5.009101 1.644044 -2.654577

H 0 -6.670121 4.810960 -2.953944

H 0 -4.320181 4.735612 -2.029432

H 0 -3.814330 3.714142 -3.399554

C -1 -9.495879 6.948237 -2.625197

C 0 -9.673685 5.547922 -2.050109

C 0 -10.154358 4.560444 -3.113901

O 0 -8.411670 5.165223 -1.497960

H 0 -8.769257 6.928158 -3.444328

H 0 -10.425655 5.592601 -1.244224

H 0 -11.091524 4.906498 -3.563709

H 0 -9.404211 4.469002 -3.906065

H 0 -10.328997 3.567740 -2.694829

H 0 -8.501703 4.309160 -1.044712

N 0 -7.674725 5.101615 6.010916

C 0 -6.726470 4.081777 5.532773

C 0 -5.367039 4.620974 5.091141

O 0 -4.407044 3.851692 4.982748

C 0 -7.459541 3.435719 4.334508

C 0 -8.941012 3.623810 4.679201

C 0 -8.974439 5.015895 5.321577

H 0 -6.528078 3.361651 6.332419

H 0 -7.163329 2.398449 4.176145

H 0 -7.216983 3.986350 3.418661

H 0 -9.258325 2.872728 5.410604

H 0 -9.595427 3.548482 3.807676

H 0 -9.060060 5.799657 4.556800

H 0 -9.801361 5.140084 6.023362

N 0 -5.345738 5.922533 4.739090

C -1 -4.224812 6.582929 4.089000

C 0 -4.630751 7.211047 2.748725

C 0 -5.273743 6.237250 1.746960

C 0 -5.681075 6.963364 0.458546

C 0 -4.363281 5.044146 1.435644

H 0 -3.455827 5.821958 3.949171

H 0 -3.732766 7.657708 2.300795

H 0 -5.323727 8.043699 2.936422

H 0 -6.191052 5.846388 2.209704

H 0 -6.277631 7.857308 0.674571

H 0 -4.793765 7.286341 -0.101073

H 0 -6.191692 6.447513 4.909334

H 0 -6.279840 6.315949 -0.189926

H 0 -4.177841 4.425141 2.318144

H 0 -4.820456 4.417953 0.665179

H 0 -3.389839 5.387450 1.061732

C -1 -0.160284 5.153841 5.413065

C 0 -1.147734 3.990616 5.330268

S 0 -1.191873 3.144781 3.701264

C 0 0.375374 2.212588 3.761758

H 0 0.868778 4.819383 5.247291

H 0 -0.391152 5.915143 4.662358

H 0 -2.177201 4.324351 5.479770

H 0 -0.935454 3.237166 6.097171

H 0 1.244143 2.863007 3.638707

H 0 0.451126 1.665247 4.704723

H 0 0.352534 1.498350 2.939201

C -1 -0.793810 9.087927 -2.939901

C 0 -0.502991 7.582669 -2.840831

C 0 -1.407118 6.919391 -1.784809

C 0 0.986172 7.339545 -2.561774

C 0 -1.249008 5.400244 -1.669117

H 0 -0.561130 9.590080 -1.993081

H 0 -0.742001 7.126142 -3.813332

H 0 -2.452949 7.155575 -2.022799

H 0 -1.206869 7.380918 -0.807435

H 0 1.610716 7.835597 -3.312670

H 0 1.265522 7.739758 -1.579224

H 0 1.238213 6.275599 -2.570932

H 0 -0.249545 5.121971 -1.322508

H 0 -1.967757 4.987266 -0.953984

H 0 -1.415191 4.905546 -2.633093

C -1 -8.524970 6.609963 7.776926

C 0 -7.437600 5.698109 7.228804

O 0 -6.383404 5.506948 7.823889

H 0 -9.364961 6.019504 8.159358

H 0 -8.098481 7.181950 8.600021

H 0 -1.848719 9.276332 -3.165867

H 0 -0.190883 9.558715 -3.723415

H 0 2.086523 5.050140 -4.667817

H 0 3.003275 -1.955692 6.973906

H 0 0.785255 1.118159 11.549314

H 0 -1.981585 -8.378987 11.625829

H 0 -2.214818 -4.189664 13.338802

H 0 -3.391682 -8.000544 4.007931

H 0 -8.110515 -7.447660 0.024642

H 0 -8.153025 -3.353799 -3.928284

H 0 -3.541834 -5.790784 -2.304653

H 0 -3.840082 -5.238889 -3.958724

H 0 0.172655 -7.312442 -0.794334

H 0 1.509298 -1.133524 -6.739416

H 0 -2.447319 1.917994 -5.666279

H 0 -3.451894 0.593029 -5.055500

H 0 -5.496586 5.462459 -4.119186

H 0 -9.124836 7.633098 -1.857539

H 0 -10.444289 7.332382 -3.012229

H 0 -6.143415 3.829890 -4.343253

H 0 -10.826000 -0.493135 -5.204114

H 0 -8.917041 7.290324 7.015327

H 0 -3.810671 7.350291 4.754487

H 0 -0.205098 5.619221 6.404417

O 0 7.121718 0.844557 -1.739264

O 0 -5.918490 -0.227737 -1.145293

H 0 7.899218 0.436941 -1.318124

H 0 6.994685 1.656683 -1.222313

H 0 -5.396922 0.882321 -2.092765

H 0 -6.849960 -0.461688 -1.248999

C -1 5.111282 1.407009 8.853950

C 0 4.273448 2.599060 8.372715

C 0 5.136367 3.858607 8.235482

C 0 3.563237 2.276935 7.052815

H 0 4.490894 0.514093 8.987529

H 0 5.891181 1.162274 8.122660

H 0 3.502203 2.798204 9.130038

H 0 5.923841 3.707301 7.487005

H 0 4.537265 4.719068 7.918755

H 0 5.622155 4.116047 9.182800

H 0 2.919810 3.103546 6.731969

H 0 4.291849 2.092933 6.255160

H 0 2.939089 1.383522 7.146541

C -1 -7.140104 -6.831046 9.563930

C 0 -6.709223 -6.057497 8.335305

C 0 -5.912496 -4.910163 8.463965

C 0 -7.113180 -6.441033 7.049305

C 0 -5.553012 -4.161629 7.342510

C 0 -6.743672 -5.700661 5.923252

C 0 -5.962210 -4.552450 6.065323

H 0 -6.352195 -6.820211 10.321815

H 0 -8.035557 -6.383784 10.013161

H 0 -5.562291 -4.621547 9.449965

H 0 -7.726413 -7.331165 6.928706

H 0 -4.954591 -3.265374 7.468183

H 0 -7.067957 -6.009747 4.933517

H 0 -5.671934 -3.977340 5.191310

H 0 -7.382772 -7.869259 9.316864

H 0 5.602441 1.623364 9.808568

H 0 7.750874 -0.487328 2.251210

H 0 -5.329529 -1.074518 -1.292518

C -1 6.647551 5.725772 0.043034

C 0 7.102118 4.567543 -0.812983

O 0 6.828618 3.399367 -0.501153

H 0 7.056543 5.596091 1.048243

N 0 7.822229 4.865785 -1.918381

C 0 8.346183 3.856243 -2.826814

C 0 9.730632 3.306691 -2.469461

O 0 10.556160 3.075554 -3.345705

H 0 7.664748 2.998324 -2.834433

H 0 8.087734 5.826387 -2.074667

N 0 9.946565 3.070919 -1.140205

C -1 11.056574 2.194052 -0.766062

C 0 10.603233 0.763049 -0.974807

O 0 9.598020 0.356001 -0.371950

H 0 11.931575 2.476364 -1.346302

H 0 9.115734 2.966642 -0.566099

N 0 11.273512 0.000625 -1.847318

C -1 10.757114 -1.336918 -2.119974

C 0 11.775720 -1.921689 -3.105612

C 0 12.275300 -0.681634 -3.863130

C 0 12.353890 0.395382 -2.772364

H 0 11.335131 -2.678345 -3.758491

H 0 12.602805 -2.388751 -2.559370

H 0 11.541490 -0.380292 -4.618070

H 0 13.234159 -0.832806 -4.364318

H 0 13.322669 0.367458 -2.257552

H 0 12.173327 1.398322 -3.160733

H 0 9.756173 -1.261310 -2.562714

H 0 5.558540 5.686160 0.129505

H 0 6.943713 6.702231 -0.346461

H 0 8.409741 4.268036 -3.833960

H 0 11.270122 2.337575 0.295986

H 0 10.659131 -1.905074 -1.192264

N 0 -4.377821 0.282255 1.128641

C 0 -3.289337 -0.168565 9.361861

C 0 -3.201032 -1.244883 8.479139

C 0 -3.514582 1.504416 1.107477

C 0 -2.535832 1.525356 -0.081105

C 0 -3.227380 -1.024558 7.100595

C 0 -3.327070 0.273160 6.588126

C 0 -1.107667 1.116900 0.232838

C 0 -3.415629 0.540739 5.095033

C 0 -0.072391 2.044217 0.049932

C 0 1.260051 1.710325 0.293719

C 0 -4.842194 0.916250 4.759928

C 0 1.565830 0.417872 0.758012

C 0 -3.411052 1.350121 7.482188

C 0 -3.395541 1.130207 8.859334

C 0 0.550307 -0.515275 0.946609

C 0 -0.777552 -0.172009 0.677776

O 0 -5.408417 0.709528 3.698989

O 0 2.865561 0.087524 1.043884

O 0 2.262913 2.630070 0.171583

H 0 -3.105190 -2.257868 8.853398

H 0 -3.170885 -1.871506 6.424873

H 0 3.368860 0.918764 1.018697

H 0 -1.545723 -0.928772 0.794930

H 0 -2.977622 1.557956 2.054150

H 0 -2.519237 2.544538 -0.477676

H 0 -5.382084 1.430954 5.571675

H 0 -3.499168 2.359893 7.089792

H 0 -0.288147 3.054023 -0.281712

H 0 -3.465256 1.974379 9.538790

H 0 0.806323 -1.504639 1.309239

H 0 -5.128847 0.338804 0.417826

H 0 -2.940075 0.901715 -0.885736

H 0 -4.194552 2.351526 1.060537

H 0 -4.808339 0.179195 2.063533

H 0 2.928661 2.365796 -0.556981

H 0 -3.872983 -0.611200 0.878259

H 0 -3.274892 -0.340544 10.432997

H 0 -2.861836 1.472797 4.889734

C 0 -2.844598 -0.561267 4.198055

H 0 -2.654078 -0.189514 3.191242

H 0 -1.883632 -0.893660 4.597619

H 0 -3.511129 -1.424010 4.121821

**Int1R** (-8045.707743)

C -1 7.283418 0.459639 1.969536

C 0 5.928224 0.657491 2.657329

C 0 5.993697 0.295032 4.145354

C 0 5.420754 2.093793 2.470748

H 0 7.211090 0.629202 0.892929

H 0 8.035061 1.153463 2.365919

H 0 5.204367 -0.014253 2.179195

H 0 6.697281 0.947322 4.677677

H 0 5.013879 0.399865 4.622041

H 0 6.325589 -0.738973 4.289368

H 0 4.410671 2.217041 2.879482

H 0 6.071441 2.806624 2.993306

H 0 5.414715 2.387734 1.415265

C -1 2.868894 -2.654445 6.139353

C 0 2.348656 -1.345676 5.531076

C 0 2.532912 -1.335128 4.010611

C 0 0.879983 -1.111423 5.905557

H 0 2.300579 -3.511651 5.759682

H 0 3.923580 -2.815150 5.892826

H 0 2.940494 -0.520108 5.952390

H 0 1.925352 -2.122225 3.547757

H 0 2.227978 -0.382707 3.569982

H 0 3.575464 -1.510114 3.728753

H 0 0.251480 -1.911603 5.499822

H 0 0.741855 -1.095635 6.991504

H 0 0.504859 -0.164264 5.506532

C -1 0.465515 0.417498 10.771769

C 0 -0.541586 0.239715 9.624595

C 0 -1.983703 0.376231 10.124152

C 0 -0.274167 1.230474 8.484414

H 0 0.392179 1.424473 11.199190

H 0 0.277413 -0.301570 11.575956

H 0 -0.419189 -0.777388 9.224798

H 0 -2.164432 1.385749 10.513267

H 0 -2.706251 0.202050 9.321010

H 0 -2.195099 -0.334119 10.927894

H 0 -0.407317 2.261951 8.833397

H 0 0.743768 1.136849 8.095538

H 0 -0.964900 1.069042 7.649872

C -1 -3.086009 -4.133081 12.675837

C 0 -3.259541 -5.445708 11.895206

O 0 -4.309130 -5.694219 11.308595

C 0 -3.079670 -2.945822 11.704690

H 0 -3.935337 -4.057012 13.361019

H 0 -2.234333 -3.008209 11.012067

H 0 -3.998184 -2.937617 11.113331

H 0 -3.011467 -2.001449 12.251456

N 0 -2.186760 -6.298022 11.875900

C -1 -2.114186 -7.479696 11.012286

C 0 -1.008546 -7.351633 9.949962

C 0 -1.121052 -6.112938 9.087615

C 0 -2.131697 -5.990780 8.124796

C 0 -0.213436 -5.057551 9.238778

C 0 -2.215628 -4.856765 7.317289

C 0 -0.295801 -3.916443 8.438157

C 0 -1.294792 -3.817725 7.469666

H 0 -3.097508 -7.560923 10.544861

H 0 -1.041126 -8.252982 9.325501

H 0 -0.030195 -7.357996 10.446637

H 0 -2.864849 -6.783642 8.006020

H 0 -1.334024 -5.986333 12.316866

H 0 0.577300 -5.138294 9.980814

H 0 -3.004918 -4.776226 6.579652

H 0 0.425795 -3.114371 8.560963

H 0 -1.359514 -2.938565 6.838568

C -1 0.153484 -6.401150 -0.187223

C 0 1.116914 -5.421076 -0.870016

C 0 0.640400 -5.050247 -2.280047

C 0 1.309909 -4.161476 -0.018096

H 0 0.546469 -6.733605 0.780355

H 0 -0.816593 -5.925389 -0.002532

H 0 2.092386 -5.919064 -0.964486

H 0 -0.328807 -4.541059 -2.239717

H 0 1.346259 -4.374133 -2.772173

H 0 0.522916 -5.939809 -2.908355

H 0 0.360749 -3.621423 0.083645

H 0 1.667660 -4.406418 0.988659

H 0 2.029855 -3.477304 -0.476722

C -1 -3.596300 -6.934477 3.871757

C 0 -2.310333 -6.113550 3.804835

S 0 -2.675227 -4.357423 3.400952

C 0 -0.970243 -3.708490 3.368975

H 0 -4.262476 -6.566677 4.656966

H 0 -4.143238 -6.897621 2.924932

H 0 -1.636384 -6.503975 3.034683

H 0 -1.786964 -6.139126 4.765898

H 0 -0.374512 -4.208027 2.600670

H 0 -1.023510 -2.643640 3.131748

H 0 -0.494297 -3.833240 4.345065

C -1 -8.591514 -6.747317 0.712460

C 0 -8.100211 -5.657748 1.646099

C 0 -6.757106 -5.272156 1.691743

C 0 -8.996087 -4.936664 2.451403

C 0 -6.320179 -4.175231 2.445246

C 0 -8.580786 -3.854888 3.220468

C 0 -7.243691 -3.438744 3.190499

O 0 -6.918092 -2.323811 3.912799

H 0 -9.244209 -7.459622 1.228230

H 0 -9.170943 -6.303766 -0.104805

H 0 -6.026607 -5.835180 1.119260

H 0 -10.047625 -5.208316 2.450230

H 0 -5.275913 -3.882541 2.429839

H 0 -9.286144 -3.293522 3.823824

H 0 -6.056542 -1.959053 3.654512

C -1 -3.718071 -4.902858 -2.923610

C 0 -3.136636 -4.588969 -1.546144

C 0 -4.196503 -4.311263 -0.460763

C 0 -4.775605 -2.922169 -0.546490

O 0 -5.488118 -2.702701 -1.634134

O 0 -4.562892 -2.074398 0.322624

H 0 -4.310662 -4.068925 -3.304868

H 0 -2.448901 -3.735933 -1.608131

H 0 -2.535522 -5.438225 -1.207130

H 0 -3.763015 -4.418613 0.535738

H 0 -5.014405 -5.029955 -0.568282

C -1 2.461238 -1.592604 -6.453616

C 0 2.743518 -1.391505 -4.979192

C 0 1.726926 -1.564759 -4.029476

C 0 4.021427 -1.046325 -4.519615

C 0 1.976031 -1.401141 -2.666423

C 0 4.286364 -0.895993 -3.155354

C 0 3.260786 -1.076936 -2.223451

H 0 3.203268 -1.083076 -7.074663

H 0 2.484121 -2.656665 -6.718213

H 0 0.725947 -1.822490 -4.362182

H 0 4.821526 -0.893174 -5.239694

H 0 1.169447 -1.515392 -1.949703

H 0 5.281640 -0.611271 -2.821880

H 0 3.453142 -0.954501 -1.161681

C -1 2.557459 4.062343 -4.609790

C 0 2.951251 3.840149 -3.146599

C 0 3.455514 2.422076 -2.890492

N 0 3.860491 2.249274 -1.480915

H 0 3.410552 3.904638 -5.278854

H 0 1.766797 3.366314 -4.909288

H 0 2.096583 4.031665 -2.487173

H 0 3.731788 4.560148 -2.860172

H 0 4.279796 2.182268 -3.577182

H 0 2.659189 1.700490 -3.095161

H 0 4.692791 2.803850 -1.279276

H 0 4.131366 1.279473 -1.328861

C -1 -2.423143 0.948920 -5.154349

C 0 -1.897572 0.772862 -3.723568

C 0 -1.954806 -0.702780 -3.301427

C 0 -0.480879 1.347187 -3.589665

H 0 -1.785478 0.411931 -5.867512

H 0 -2.557726 1.333951 -3.047712

H 0 -2.990564 -1.054020 -3.229537

H 0 -1.441762 -1.337833 -4.033399

H 0 -1.470826 -0.857643 -2.332114

H 0 0.214801 0.834472 -4.263615

H 0 -0.466523 2.414763 -3.839184

H 0 -0.098196 1.235042 -2.571460

C -1 -8.783598 -2.890511 -3.160672

C 0 -8.951388 -3.921703 -2.051709

S 0 -9.856922 -3.297436 -0.585062

C 0 -8.536210 -2.287126 0.161841

H 0 -9.752423 -2.540695 -3.522140

H 0 -8.226342 -2.018289 -2.813651

H 0 -7.979094 -4.294716 -1.713041

H 0 -9.529626 -4.781202 -2.405332

H 0 -8.234845 -1.471829 -0.496396

H 0 -8.935356 -1.862449 1.083007

H 0 -7.682196 -2.916424 0.412440

C -1 -11.413996 -0.216773 -4.324880

C 0 -10.536068 0.313602 -3.213001

C 0 -9.429710 1.124226 -3.477114

C 0 -10.816462 0.029829 -1.867819

C 0 -8.634792 1.646509 -2.450834

C 0 -10.041152 0.541879 -0.834147

C 0 -8.937588 1.364055 -1.106797

O 0 -8.214443 1.831067 -0.062049

H 0 -11.788696 -1.218570 -4.092357

H 0 -12.288305 0.425602 -4.487552

H 0 -9.176254 1.365551 -4.506519

H 0 -11.649862 -0.621037 -1.622300

H 0 -7.812585 2.311629 -2.685931

H 0 -10.270137 0.309321 0.199891

H 0 -7.540224 2.501589 -0.364968

C -1 -5.820503 4.558404 -3.595440

C 0 -4.818437 3.892146 -2.654460

C 0 -5.491963 3.128849 -1.524251

O 0 -6.490770 3.634581 -0.941071

O 0 -5.003426 1.990373 -1.221713

H 0 -6.451794 5.271747 -3.063551

H 0 -4.173619 4.652698 -2.196207

H 0 -4.160290 3.199985 -3.186513

C -1 -9.496081 6.948664 -2.624149

C 0 -9.081106 6.204398 -1.356474

C 0 -9.682470 4.803437 -1.304729

O 0 -7.657343 6.187096 -1.304953

H 0 -9.190297 6.380314 -3.508963

H 0 -9.450757 6.772663 -0.485011

H 0 -10.776060 4.850828 -1.280266

H 0 -9.391493 4.225842 -2.186378

H 0 -9.347299 4.254345 -0.420071

H 0 -7.333516 5.290719 -1.094090

N 0 -7.623054 5.043374 6.079221

C 0 -6.630537 4.039375 5.644352

C 0 -5.329109 4.609817 5.088829

O 0 -4.336205 3.878862 4.990940

C 0 -7.379827 3.219984 4.570601

C 0 -8.849083 3.352763 4.984868

C 0 -8.941053 4.806522 5.462512

H 0 -6.349741 3.415544 6.493207

H 0 -7.032827 2.186975 4.538527

H 0 -7.224124 3.662339 3.580093

H 0 -9.060620 2.676965 5.820502

H 0 -9.546430 3.129950 4.174172

H 0 -9.101153 5.488022 4.616483

H 0 -9.746651 4.967262 6.180880

N 0 -5.371695 5.884925 4.653686

C -1 -4.224615 6.582489 4.089191

C 0 -4.417213 7.016155 2.634164

C 0 -4.704892 5.899497 1.616543

C 0 -4.825777 6.513123 0.218162

C 0 -3.648340 4.786665 1.637490

H 0 -3.379921 5.897625 4.180448

H 0 -3.501891 7.544831 2.332708

H 0 -5.225458 7.758495 2.580205

H 0 -5.673860 5.447164 1.870499

H 0 -5.625806 7.255984 0.163469

H 0 -3.883269 6.993354 -0.072796

H 0 -6.223463 6.396265 4.835585

H 0 -5.064258 5.746192 -0.517676

H 0 -2.649584 5.186215 1.421800

H 0 -3.598724 4.277120 2.603076

H 0 -3.876971 4.038816 0.869684

C -1 -0.160150 5.154539 5.413290

C 0 -1.002595 3.893227 5.625420

S 0 -1.186384 2.800687 4.159260

C 0 0.546487 2.316377 3.839931

H 0 0.876949 4.908166 5.167335

H 0 -0.566793 5.762250 4.599871

H 0 -2.038033 4.149208 5.867108

H 0 -0.600528 3.295514 6.450766

H 0 1.033951 1.982481 4.760079

H 0 0.511424 1.485742 3.133005

H 0 1.119711 3.127490 3.386698

C -1 -0.793687 9.088389 -2.939513

C 0 -0.429312 7.598683 -2.824942

C 0 -1.345093 6.884548 -1.812797

C 0 1.055835 7.431240 -2.478652

C 0 -1.129254 5.370939 -1.696571

H 0 -0.633457 9.600460 -1.982999

H 0 -0.599604 7.132806 -3.807461

H 0 -2.388393 7.074672 -2.095388

H 0 -1.210868 7.348474 -0.825003

H 0 1.689285 7.953706 -3.203964

H 0 1.270649 7.848942 -1.487057

H 0 1.357649 6.380256 -2.468840

H 0 -0.151663 5.120445 -1.272552

H 0 -1.887905 4.922228 -1.047224

H 0 -1.203315 4.883509 -2.675974

C -1 -8.524967 6.610027 7.776985

C 0 -7.394532 5.731331 7.249395

O 0 -6.314464 5.651598 7.825367

H 0 -9.307120 5.994862 8.235528

H 0 -8.106960 7.266714 8.538932

H 0 -1.845008 9.220653 -3.215575

H 0 -0.179020 9.590779 -3.693825

H 0 2.186391 5.079147 -4.769413

H 0 2.773081 -2.654008 7.230985

H 0 1.494345 0.277969 10.424881

H 0 -1.948223 -8.377339 11.619098

H 0 -2.170306 -4.146285 13.278581

H 0 -3.358278 -7.980514 4.088133

H 0 -7.756897 -7.305018 0.277864

H 0 -8.228465 -3.320669 -4.002496

H 0 -4.370824 -5.781741 -2.875790

H 0 -2.919289 -5.116968 -3.641020

H 0 -0.020537 -7.290112 -0.802685

H 0 1.471160 -1.212328 -6.723255

H 0 -2.434914 2.004491 -5.447393

H 0 -3.441762 0.559360 -5.256283

H 0 -5.298031 5.087740 -4.398599

H 0 -9.010262 7.927907 -2.664291

H 0 -10.581130 7.095786 -2.660727

H 0 -6.470164 3.810847 -4.064903

H 0 -10.867879 -0.271751 -5.271097

H 0 -8.990306 7.205596 6.986506

H 0 -4.006088 7.459802 4.710343

H 0 -0.153541 5.757475 6.328004

O 0 7.167052 0.887010 -1.866370

O 0 -6.158010 -0.277408 -2.136580

H 0 7.915721 0.508598 -1.372421

H 0 6.922446 1.671555 -1.350404

H 0 -5.653998 0.533152 -1.895186

H 0 -7.078879 0.013369 -2.232134

C -1 5.112461 1.407609 8.853664

C 0 3.658154 1.769822 8.526687

C 0 3.102212 2.773044 9.543631

C 0 3.524744 2.312103 7.098376

H 0 5.508001 0.670418 8.146485

H 0 5.753349 2.295753 8.802330

H 0 3.060410 0.848343 8.594024

H 0 3.677297 3.706458 9.515314

H 0 2.056840 3.017540 9.332580

H 0 3.154518 2.379081 10.563742

H 0 2.480057 2.526472 6.848064

H 0 4.089690 3.244988 6.983057

H 0 3.906908 1.600511 6.360022

C -1 -7.140065 -6.831101 9.564072

C 0 -6.669136 -6.026353 8.377059

C 0 -5.881908 -4.883394 8.573115

C 0 -7.020623 -6.379168 7.068018

C 0 -5.472481 -4.109995 7.488917

C 0 -6.604316 -5.611603 5.978417

C 0 -5.825048 -4.472663 6.187643

H 0 -6.374176 -6.852436 10.344121

H 0 -8.043542 -6.390057 10.003649

H 0 -5.575524 -4.623193 9.581025

H 0 -7.629320 -7.264773 6.901295

H 0 -4.864300 -3.227823 7.660282

H 0 -6.891510 -5.889594 4.969013

H 0 -5.504384 -3.876490 5.343128

H 0 -7.385924 -7.858874 9.279098

H 0 5.201101 0.990651 9.862568

H 0 7.663973 -0.556408 2.115223

H 0 -5.767641 -1.731259 -1.728156

C -1 6.648273 5.726950 0.042445

C 0 7.053443 4.581017 -0.854965

O 0 6.663092 3.425884 -0.630718

H 0 6.955977 5.492064 1.064395

N 0 7.863335 4.881415 -1.895821

C 0 8.380277 3.892635 -2.829127

C 0 9.755019 3.325200 -2.467854

O 0 10.588286 3.095681 -3.336267

H 0 7.691451 3.041878 -2.861010

H 0 8.210283 5.825780 -1.972051

N 0 9.958103 3.081199 -1.136465

C -1 11.059178 2.194981 -0.759662

C 0 10.599142 0.767600 -0.978390

O 0 9.583658 0.366812 -0.388141

H 0 11.939127 2.475715 -1.333037

H 0 9.121050 2.964738 -0.575208

N 0 11.276058 0.002648 -1.843030

C -1 10.757302 -1.332562 -2.124991

C 0 11.783585 -1.918285 -3.101909

C 0 12.295043 -0.678074 -3.851093

C 0 12.366894 0.395720 -2.756698

H 0 11.346626 -2.671408 -3.761261

H 0 12.603578 -2.389917 -2.548914

H 0 11.569751 -0.372050 -4.612294

H 0 13.258261 -0.831344 -4.343172

H 0 13.330508 0.363226 -2.232595

H 0 12.193799 1.400229 -3.144459

H 0 9.761072 -1.251157 -2.576954

H 0 5.557451 5.801306 0.041992

H 0 7.073377 6.688257 -0.253903

H 0 8.456710 4.327295 -3.826163

H 0 11.267400 2.331572 0.304420

H 0 10.648178 -1.902799 -1.199782

N 0 -4.819600 0.550126 0.981839

C 0 -6.458936 1.966149 8.770769

C 0 -5.167346 2.464883 8.592558

C 0 -3.357515 0.707041 1.264338

C 0 -2.547352 0.602925 -0.027390

C 0 -4.332251 1.906036 7.625707

C 0 -4.774726 0.840562 6.834331

C 0 -1.063637 0.565778 0.239028

C 0 -3.883747 0.262088 5.736667

C 0 -0.281498 1.720031 0.103031

C 0 1.095550 1.688518 0.323069

C 0 -4.533838 0.713777 4.454680

C 0 1.692733 0.478328 0.727535

C 0 -6.077777 0.352894 7.007706

C 0 -6.912203 0.910324 7.977002

C 0 0.927895 -0.675540 0.867863

C 0 -0.444182 -0.631721 0.616527

O 0 -5.143837 -0.028419 3.689651

O 0 3.038689 0.431535 0.986965

O 0 1.864078 2.809314 0.205378

H 0 -4.825362 3.309899 9.179651

H 0 -3.338527 2.310662 7.466824

H 0 3.342535 1.350043 1.069192

H 0 -1.040357 -1.535916 0.710326

H 0 -3.080644 -0.087016 1.959142

H 0 -2.807511 1.437216 -0.680014

H 0 -4.475679 1.799045 4.269281

H 0 -6.443066 -0.456593 6.381600

H 0 -0.730488 2.661164 -0.198355

H 0 -7.917024 0.520264 8.108092

H 0 1.416745 -1.592633 1.173172

H 0 -5.127384 1.245518 0.250712

H 0 -2.852454 -0.312157 -0.543791

H 0 -3.201759 1.670182 1.756496

H 0 -5.348156 0.604386 1.860762

H 0 2.631938 2.625510 -0.446197

H 0 -4.961715 -0.405652 0.594316

H 0 -7.111918 2.408425 9.516629

C 0 -3.691038 -1.251700 5.815252

H 0 -3.042107 -1.609211 5.010938

H 0 -3.238888 -1.524954 6.771450

H 0 -4.641878 -1.778905 5.726169

H 0 -2.917842 0.780867 5.777567

**TS1R** (-8045.70101)

C -1 7.280818 0.460519 1.970017

C 0 5.974957 0.610685 2.759319

C 0 6.217757 0.467853 4.266071

C 0 5.287034 1.945754 2.444420

H 0 7.096904 0.479573 0.893426

H 0 7.977518 1.275413 2.200784

H 0 5.297367 -0.197451 2.450385

H 0 6.899037 1.250413 4.624131

H 0 5.285595 0.552256 4.831852

H 0 6.670923 -0.500135 4.505804

H 0 4.308118 2.020815 2.930371

H 0 5.897356 2.786627 2.796878

H 0 5.151438 2.095388 1.367609

C -1 2.867901 -2.653783 6.140192

C 0 3.247579 -2.052759 4.778741

C 0 2.839200 -2.974168 3.623167

C 0 2.634967 -0.661824 4.584849

H 0 1.779491 -2.757778 6.230684

H 0 3.305232 -3.649112 6.271817

H 0 4.340961 -1.942890 4.752109

H 0 1.752149 -3.113968 3.613056

H 0 3.128868 -2.545177 2.658810

H 0 3.300319 -3.963841 3.713117

H 0 1.540987 -0.710359 4.595755

H 0 2.943351 0.028103 5.377378

H 0 2.928584 -0.232810 3.622900

C -1 0.466613 0.416857 10.770591

C 0 1.323292 0.870876 9.584514

C 0 1.755127 -0.333761 8.743072

C 0 0.593067 1.917157 8.732383

H 0 -0.458923 -0.059383 10.423922

H 0 1.000290 -0.310863 11.391589

H 0 2.231272 1.339080 9.988609

H 0 0.885345 -0.900188 8.393799

H 0 2.323248 -0.025519 7.861160

H 0 2.382486 -1.020456 9.321847

H 0 -0.325003 1.505119 8.300781

H 0 0.315935 2.791701 9.330387

H 0 1.220614 2.262462 7.904409

C -1 -3.085966 -4.132903 12.675572

C 0 -3.278313 -5.476148 11.953809

O 0 -4.368278 -5.786078 11.479001

C 0 -3.089601 -2.992050 11.645876

H 0 -3.922028 -4.020958 13.371013

H 0 -2.274475 -3.112600 10.925000

H 0 -4.030902 -2.992848 11.090533

H 0 -2.976946 -2.021147 12.136965

N 0 -2.171344 -6.273523 11.842813

C -1 -2.114120 -7.480098 11.012461

C 0 -0.989612 -7.396321 9.965666

C 0 -1.124035 -6.233393 9.007241

C 0 -2.028307 -6.289667 7.939230

C 0 -0.356926 -5.072575 9.169508

C 0 -2.151276 -5.222899 7.050738

C 0 -0.481124 -3.998818 8.284306

C 0 -1.377413 -4.073605 7.217683

H 0 -3.091711 -7.557003 10.531671

H 0 -0.982609 -8.342433 9.411477

H 0 -0.023172 -7.336226 10.482735

H 0 -2.645052 -7.174195 7.803043

H 0 -1.299831 -5.915923 12.205202

H 0 0.357693 -5.015957 9.987508

H 0 -2.856592 -5.270309 6.231225

H 0 0.130217 -3.111337 8.418259

H 0 -1.484817 -3.243769 6.528742

C -1 0.152425 -6.400801 -0.187607

C 0 1.333850 -5.529623 -0.636478

C 0 1.253432 -5.205158 -2.132297

C 0 1.415809 -4.239465 0.186854

H 0 0.222316 -6.648307 0.878021

H 0 -0.798158 -5.876420 -0.343616

H 0 2.257535 -6.100426 -0.463825

H 0 0.374217 -4.585785 -2.339283

H 0 2.129456 -4.641205 -2.467105

H 0 1.175590 -6.113694 -2.739323

H 0 0.491498 -3.658258 0.085325

H 0 1.564656 -4.448795 1.251202

H 0 2.240142 -3.602117 -0.148687

C -1 -3.596689 -6.933811 3.872112

C 0 -2.304935 -6.165559 3.603317

S 0 -2.662374 -4.384255 3.330545

C 0 -0.946625 -3.771388 3.285676

H 0 -4.100764 -6.572919 4.772911

H 0 -4.297843 -6.827479 3.038163

H 0 -1.793017 -6.560075 2.718693

H 0 -1.623831 -6.249300 4.456370

H 0 -0.368928 -4.290841 2.517950

H 0 -0.978592 -2.709685 3.039943

H 0 -0.463506 -3.892230 4.258932

C -1 -8.593010 -6.748011 0.713019

C 0 -7.664363 -5.664345 1.202682

C 0 -6.425714 -5.438323 0.596415

C 0 -8.027503 -4.822466 2.265269

C 0 -5.588970 -4.398518 1.004150

C 0 -7.211786 -3.772887 2.677335

C 0 -5.996073 -3.532373 2.024446

O 0 -5.257425 -2.460198 2.419662

H 0 -8.963879 -7.366434 1.537839

H 0 -9.466865 -6.309819 0.216231

H 0 -6.098894 -6.088845 -0.211283

H 0 -8.980764 -4.976871 2.763607

H 0 -4.616648 -4.269628 0.549319

H 0 -7.513397 -3.110415 3.481731

H 0 -4.595191 -2.251813 1.734763

C -1 -3.717833 -4.902630 -2.924002

C 0 -2.586579 -3.888796 -2.737553

C 0 -2.526168 -3.261159 -1.341928

C 0 -3.685332 -2.367986 -0.956571

O 0 -4.584811 -2.199622 -1.903927

O 0 -3.756854 -1.835373 0.152133

H 0 -4.695247 -4.433806 -2.797036

H 0 -2.675929 -3.091499 -3.480839

H 0 -1.628072 -4.383611 -2.924757

H 0 -1.630785 -2.637444 -1.233651

H 0 -2.443049 -4.024664 -0.557610

C -1 2.462901 -1.588928 -6.455089

C 0 2.739465 -1.445558 -4.972665

C 0 1.702932 -1.571410 -4.037105

C 0 4.032541 -1.201624 -4.492136

C 0 1.944682 -1.451001 -2.668156

C 0 4.287640 -1.098140 -3.122009

C 0 3.241692 -1.220173 -2.203148

H 0 3.229519 -1.088857 -7.053348

H 0 2.447503 -2.643938 -6.754321

H 0 0.692698 -1.757021 -4.389243

H 0 4.850725 -1.089820 -5.199358

H 0 1.124257 -1.526124 -1.961528

H 0 5.297329 -0.894658 -2.774055

H 0 3.423864 -1.117407 -1.137643

C -1 2.557008 4.064075 -4.609262

C 0 2.965734 3.703103 -3.176145

C 0 3.659092 2.343499 -3.090100

N 0 4.104642 2.053272 -1.712706

H 0 3.431526 4.125980 -5.266513

H 0 1.880406 3.310984 -5.024414

H 0 2.091016 3.695071 -2.515598

H 0 3.640122 4.477415 -2.781760

H 0 4.499500 2.307773 -3.800133

H 0 2.965183 1.551031 -3.385456

H 0 4.820689 2.720553 -1.425471

H 0 4.563781 1.144361 -1.693150

C -1 -2.421899 0.949307 -5.153810

C 0 -1.702826 1.039825 -3.802406

C 0 -1.742188 -0.317344 -3.098050

C 0 -0.269508 1.563538 -3.943208

H 0 -1.927867 0.222260 -5.810038

H 0 -2.258033 1.748079 -3.175415

H 0 -2.777806 -0.629403 -2.940227

H 0 -1.254089 -1.086683 -3.706961

H 0 -1.231939 -0.281496 -2.131432

H 0 0.337915 0.899228 -4.568444

H 0 -0.258646 2.559686 -4.399831

H 0 0.219648 1.632391 -2.965681

C -1 -8.784042 -2.890612 -3.161081

C 0 -9.117636 -3.886723 -2.053734

S 0 -10.069672 -3.184954 -0.652709

C 0 -8.803717 -2.096551 0.084490

H 0 -9.686258 -2.407764 -3.541700

H 0 -8.111617 -2.108684 -2.797638

H 0 -8.211291 -4.346848 -1.645044

H 0 -9.748033 -4.693873 -2.439780

H 0 -8.686720 -1.167127 -0.473181

H 0 -9.136107 -1.854806 1.095045

H 0 -7.850752 -2.622168 0.159577

C -1 -11.413946 -0.217145 -4.324783

C 0 -10.484045 0.429234 -3.320207

C 0 -9.241843 0.950633 -3.694760

C 0 -10.853099 0.547422 -1.971867

C 0 -8.409872 1.606116 -2.780369

C 0 -10.039284 1.194327 -1.046631

C 0 -8.819670 1.748077 -1.450999

O 0 -8.107706 2.442608 -0.508664

H 0 -11.888367 -1.109946 -3.905855

H 0 -12.214614 0.470642 -4.621054

H 0 -8.913056 0.859589 -4.726430

H 0 -11.792001 0.116793 -1.637067

H 0 -7.462131 2.019652 -3.108376

H 0 -10.331576 1.281682 -0.005913

H 0 -7.201143 2.634199 -0.812074

C -1 -5.820116 4.559280 -3.594172

C 0 -4.553667 3.916616 -2.992246

C 0 -4.882204 2.720262 -2.125201

O 0 -5.248170 2.993890 -0.889739

O 0 -4.837202 1.571160 -2.585853

H 0 -6.535388 4.860953 -2.823420

H 0 -4.005802 4.653975 -2.399009

H 0 -3.901743 3.564324 -3.795756

C -1 -9.495877 6.948233 -2.625194

C 0 -9.723050 5.634282 -1.889039

C 0 -10.412933 4.597544 -2.775759

O 0 -8.440038 5.188906 -1.447251

H 0 -8.875231 6.778842 -3.511418

H 0 -10.364607 5.830356 -1.012777

H 0 -11.375826 4.976158 -3.135799

H 0 -9.784458 4.365356 -3.641104

H 0 -10.598369 3.665204 -2.236177

H 0 -8.543869 4.371716 -0.933947

N 0 -7.791159 5.186992 5.887239

C 0 -6.911667 4.159128 5.300676

C 0 -5.513961 4.644388 4.920098

O 0 -4.587205 3.841690 4.803392

C 0 -7.679371 3.703632 4.041040

C 0 -9.146941 3.935286 4.416283

C 0 -9.099876 5.245787 5.213021

H 0 -6.768294 3.340037 6.011826

H 0 -7.430154 2.676645 3.769023

H 0 -7.405590 4.348071 3.197584

H 0 -9.507029 3.123699 5.057863

H 0 -9.807490 3.999864 3.548170

H 0 -9.152901 6.114184 4.541859

H 0 -9.913352 5.333169 5.936463

N 0 -5.424245 5.958180 4.616893

C -1 -4.224802 6.582894 4.088992

C 0 -4.372696 7.009763 2.622450

C 0 -4.669789 5.872118 1.632342

C 0 -4.910753 6.430477 0.224449

C 0 -3.562973 4.812341 1.619111

H 0 -3.415300 5.860921 4.208746

H 0 -3.443906 7.515469 2.323051

H 0 -5.168098 7.765385 2.552402

H 0 -5.599192 5.381518 1.954142

H 0 -5.704683 7.185429 0.218694

H 0 -3.999566 6.897405 -0.169954

H 0 -6.237930 6.525672 4.802757

H 0 -5.209095 5.629250 -0.456373

H 0 -3.454023 4.326174 2.590885

H 0 -3.797943 4.041575 0.882103

H 0 -2.594998 5.251984 1.348795

C -1 -0.160279 5.153837 5.413064

C 0 -1.051089 3.918629 5.245641

S 0 -0.868178 3.059688 3.633782

C 0 0.747686 2.256184 3.889540

H 0 0.900412 4.891072 5.351098

H 0 -0.369096 5.892768 4.633834

H 0 -2.114721 4.169096 5.300931

H 0 -0.858521 3.186717 6.038131

H 0 1.565094 2.980327 3.934298

H 0 0.730535 1.659980 4.805413

H 0 0.911216 1.595252 3.038384

C -1 -0.793809 9.087927 -2.939900

C 0 -0.422853 7.598922 -2.848465

C 0 -1.349166 6.862933 -1.861225

C 0 1.058861 7.437499 -2.483103

C 0 -1.102154 5.354986 -1.746751

H 0 -0.646368 9.583117 -1.972880

H 0 -0.575439 7.149667 -3.841671

H 0 -2.389963 7.036345 -2.164976

H 0 -1.243763 7.323139 -0.868425

H 0 1.696206 7.990728 -3.181564

H 0 1.252372 7.826975 -1.475889

H 0 1.377235 6.391344 -2.501396

H 0 -0.114989 5.140316 -1.326936

H 0 -1.844585 4.885548 -1.092036

H 0 -1.159539 4.864128 -2.725574

C -1 -8.524965 6.609951 7.776917

C 0 -7.501754 5.676015 7.140712

O 0 -6.453865 5.380158 7.702848

H 0 -9.400595 6.046883 8.117913

H 0 -8.055095 7.081677 8.638440

H 0 -1.843312 9.220159 -3.224572

H 0 -0.174787 9.607340 -3.678777

H 0 2.042748 5.029385 -4.640668

H 0 3.211799 -2.022314 6.964468

H 0 0.184232 1.261640 11.407587

H 0 -1.973148 -8.365656 11.643677

H 0 -2.157722 -4.124296 13.258908

H 0 -3.380741 -7.998866 4.003176

H 0 -8.092605 -7.406880 -0.002168

H 0 -8.277075 -3.395456 -3.991599

H 0 -3.634228 -5.718985 -2.197021

H 0 -3.679607 -5.346800 -3.923613

H 0 0.110209 -7.340202 -0.749602

H 0 1.490727 -1.161788 -6.719788

H 0 -2.424385 1.915401 -5.672572

H 0 -3.461914 0.639140 -5.015238

H 0 -5.543444 5.444549 -4.173565

H 0 -8.979436 7.663301 -1.978931

H 0 -10.446730 7.385022 -2.945371

H 0 -6.320178 3.860805 -4.271886

H 0 -10.878254 -0.510209 -5.231773

H 0 -8.874674 7.375556 7.077837

H 0 -3.977086 7.454542 4.705209

H 0 -0.333575 5.620049 6.389893

O 0 7.116201 0.837280 -1.719500

O 0 -6.295035 -0.328185 -1.435922

H 0 7.892704 0.431974 -1.293719

H 0 6.980820 1.647411 -1.200777

H 0 -5.759128 0.382533 -1.879640

H 0 -7.157104 -0.308307 -1.875231

C -1 5.111305 1.406961 8.853962

C 0 4.475647 2.720989 8.380588

C 0 5.424670 3.902528 8.612398

C 0 4.054529 2.640540 6.908337

H 0 4.432284 0.560950 8.715198

H 0 6.025875 1.195289 8.286734

H 0 3.570172 2.894335 8.978170

H 0 6.347034 3.776854 8.032211

H 0 4.965500 4.848746 8.306798

H 0 5.704638 3.988817 9.667764

H 0 3.566867 3.565835 6.582299

H 0 4.923138 2.474834 6.260902

H 0 3.352534 1.818999 6.735422

C -1 -7.140103 -6.831038 9.563923

C 0 -6.648904 -6.049343 8.362854

C 0 -5.682494 -5.044104 8.511695

C 0 -7.156395 -6.294721 7.078947

C 0 -5.250796 -4.300834 7.411138

C 0 -6.721273 -5.558264 5.975202

C 0 -5.765122 -4.553291 6.137038

H 0 -6.376268 -6.862809 10.344692

H 0 -8.033900 -6.363242 9.995532

H 0 -5.253075 -4.870387 9.493765

H 0 -7.899648 -7.077105 6.942621

H 0 -4.488212 -3.539037 7.540635

H 0 -7.120269 -5.765331 4.986476

H 0 -5.411354 -3.989849 5.278804

H 0 -7.411631 -7.854965 9.287842

H 0 5.380023 1.455000 9.914720

H 0 7.786306 -0.482104 2.202429

H 0 -5.311588 -1.554716 -1.626119

C -1 6.647549 5.725771 0.043034

C 0 7.086466 4.555873 -0.805597

O 0 6.803287 3.392741 -0.484139

H 0 7.055797 5.597742 1.048740

N 0 7.804964 4.842796 -1.915666

C 0 8.324207 3.827782 -2.819931

C 0 9.713502 3.287183 -2.468593

O 0 10.533085 3.050237 -3.348860

H 0 7.646068 2.967317 -2.815130

H 0 8.077305 5.800862 -2.075687

N 0 9.941745 3.066051 -1.138606

C -1 11.056572 2.194051 -0.766060

C 0 10.605887 0.761350 -0.971356

O 0 9.603188 0.352731 -0.365272

H 0 11.928778 2.477801 -1.349679

H 0 9.115905 2.964034 -0.557100

N 0 11.274588 -0.000101 -1.846114

C -1 10.757113 -1.336918 -2.119975

C 0 11.772297 -1.920124 -3.110006

C 0 12.270358 -0.678697 -3.866192

C 0 12.352705 0.395775 -2.773264

H 0 11.329236 -2.675062 -3.763230

H 0 12.600732 -2.388953 -2.567331

H 0 11.534418 -0.375012 -4.618114

H 0 13.227623 -0.829276 -4.370605

H 0 13.322849 0.366144 -2.261134

H 0 12.171382 1.399324 -3.159457

H 0 9.754717 -1.259832 -2.559226

H 0 5.558196 5.701485 0.131260

H 0 6.955791 6.695641 -0.353402

H 0 8.378450 4.231068 -3.831153

H 0 11.272669 2.340807 0.294926

H 0 10.661849 -1.907138 -1.193228

N 0 -4.881856 1.130319 0.808887

C 0 -3.466501 1.079872 1.259051

C 0 -2.526133 1.228712 0.058855

C 0 -1.081411 0.888974 0.354552

C 0 -0.072628 1.832762 0.127198

C 0 1.273895 1.526315 0.329332

C 0 1.619166 0.246366 0.800773

C 0 0.629048 -0.704734 1.038189

C 0 -0.712425 -0.388388 0.804494

O 0 2.937076 -0.054128 1.040930

O 0 2.253426 2.463357 0.162821

H 0 3.417117 0.790708 1.000988

H 0 -1.477481 -1.142737 0.962698

H 0 -3.256824 0.126511 1.747710

H 0 -2.588127 2.248939 -0.331666

H 0 -0.323576 2.832749 -0.208315

H 0 0.918296 -1.681291 1.409037

H 0 -5.125108 2.131972 -0.152136

H 0 -2.893440 0.565762 -0.730362

H 0 -3.292429 1.869845 1.991809

H 0 -5.509075 1.177230 1.615551

H 0 2.903749 2.218968 -0.584268

H 0 -5.111318 0.245445 0.351107

C 0 -0.967473 -0.537739 6.145189

C 0 -1.427995 -0.585216 4.827347

C 0 -2.793190 -0.681241 4.577733

C 0 -3.725525 -0.700365 5.624694

C 0 -5.195946 -0.670288 5.253051

C 0 -5.492011 0.649442 4.555852

C 0 -3.253931 -0.659467 6.939720

C 0 -1.882903 -0.588569 7.196679

O 0 -6.270587 0.743570 3.618491

H 0 -0.730804 -0.533831 3.997533

H 0 -3.165114 -0.718817 3.559642

H 0 -4.988662 1.548164 4.963656

H 0 -3.950628 -0.680341 7.770401

H 0 -1.532367 -0.572640 8.223742

H 0 0.094458 -0.463867 6.350166

H 0 -5.398926 -1.458430 4.521437

C 0 -6.178518 -0.798188 6.430669

H 0 -6.004444 -1.726814 6.979866

H 0 -6.083777 0.040322 7.129542

H 0 -7.204488 -0.811945 6.054179

**Int2R** (-8045.69897)

C -1 7.280822 0.460511 1.970023

C 0 5.959888 0.609816 2.733579

C 0 6.173025 0.483891 4.246208

C 0 5.270080 1.937085 2.391794

H 0 7.115640 0.466721 0.890185

H 0 7.967316 1.283092 2.204396

H 0 5.293491 -0.206030 2.420286

H 0 6.839228 1.276462 4.610633

H 0 5.228047 0.565023 4.790998

H 0 6.629918 -0.477313 4.505393

H 0 4.284158 2.013883 2.863086

H 0 5.871209 2.785845 2.741033

H 0 5.148618 2.071291 1.311580

C -1 2.867917 -2.653787 6.140224

C 0 3.236386 -2.044745 4.779677

C 0 2.871091 -2.984699 3.624530

C 0 2.566518 -0.681668 4.579305

H 0 1.782131 -2.782678 6.229811

H 0 3.328402 -3.638800 6.273392

H 0 4.324747 -1.893384 4.760141

H 0 1.791950 -3.176928 3.616610

H 0 3.137978 -2.542291 2.659682

H 0 3.379435 -3.950995 3.714054

H 0 1.477462 -0.792632 4.575583

H 0 2.827655 0.019453 5.379072

H 0 2.852008 -0.235865 3.622745

C -1 0.466626 0.416863 10.770591

C 0 1.308400 0.872391 9.574525

C 0 1.708919 -0.329024 8.713704

C 0 0.576980 1.936731 8.746022

H 0 -0.467694 -0.049987 10.434656

H 0 1.003998 -0.319375 11.378139

H 0 2.229519 1.325582 9.965355

H 0 0.824831 -0.870076 8.361148

H 0 2.278160 -0.021398 7.832443

H 0 2.323197 -1.038926 9.278336

H 0 -0.354379 1.541814 8.327269

H 0 0.322810 2.808867 9.357583

H 0 1.193826 2.281844 7.909978

C -1 -3.085957 -4.132915 12.675590

C 0 -3.275677 -5.472561 11.946149

O 0 -4.360733 -5.774684 11.455898

C 0 -3.117425 -2.983717 11.656288

H 0 -3.911816 -4.035054 13.385492

H 0 -2.312749 -3.090188 10.921657

H 0 -4.067312 -2.989086 11.115966

H 0 -3.006395 -2.016133 12.154255

N 0 -2.174081 -6.279343 11.850700

C -1 -2.114104 -7.480106 11.012477

C 0 -1.006978 -7.378234 9.948385

C 0 -1.165080 -6.207122 9.004178

C 0 -2.089059 -6.257361 7.953073

C 0 -0.402452 -5.043545 9.164646

C 0 -2.234411 -5.182721 7.077721

C 0 -0.549108 -3.961418 8.293194

C 0 -1.463494 -4.030563 7.241458

H 0 -3.098283 -7.567632 10.547605

H 0 -1.001974 -8.318871 9.384813

H 0 -0.033084 -7.315960 10.450891

H 0 -2.704893 -7.143048 7.820418

H 0 -1.305106 -5.930364 12.227295

H 0 0.325110 -4.990178 9.971339

H 0 -2.960640 -5.225768 6.276523

H 0 0.055959 -3.070407 8.431006

H 0 -1.587908 -3.194430 6.562888

C -1 0.152412 -6.400832 -0.187597

C 0 1.342064 -5.532249 -0.623800

C 0 1.299018 -5.232187 -2.126726

C 0 1.401076 -4.229503 0.181614

H 0 0.199054 -6.630732 0.882687

H 0 -0.795417 -5.880806 -0.373340

H 0 2.262806 -6.097573 -0.420136

H 0 0.417555 -4.630486 -2.375055

H 0 2.176304 -4.660040 -2.443838

H 0 1.254458 -6.151946 -2.720042

H 0 0.474284 -3.657118 0.057033

H 0 1.534272 -4.423272 1.250842

H 0 2.226575 -3.590734 -0.148143

C -1 -3.596679 -6.933815 3.872112

C 0 -2.296210 -6.167118 3.648462

S 0 -2.647807 -4.388886 3.354140

C 0 -0.934042 -3.770933 3.347057

H 0 -4.134984 -6.563411 4.749133

H 0 -4.264945 -6.835635 3.010622

H 0 -1.750778 -6.565697 2.785930

H 0 -1.648337 -6.244165 4.527538

H 0 -0.338461 -4.287731 2.591469

H 0 -0.970519 -2.709377 3.102258

H 0 -0.471507 -3.888870 4.330609

C -1 -8.593022 -6.748017 0.713012

C 0 -7.654303 -5.672369 1.200278

C 0 -6.405194 -5.472910 0.606556

C 0 -8.019430 -4.809259 2.244797

C 0 -5.558038 -4.440819 1.012217

C 0 -7.192479 -3.768223 2.655695

C 0 -5.962367 -3.557913 2.019425

O 0 -5.207845 -2.497442 2.413083

H 0 -8.999802 -7.336453 1.542543

H 0 -9.443412 -6.303659 0.182092

H 0 -6.078749 -6.137930 -0.189399

H 0 -8.982332 -4.941478 2.730666

H 0 -4.579509 -4.331119 0.564777

H 0 -7.494366 -3.090866 3.447542

H 0 -4.507648 -2.342061 1.751649

C -1 -3.717779 -4.902603 -2.923999

C 0 -2.568110 -3.923969 -2.677097

C 0 -2.543517 -3.310122 -1.273633

C 0 -3.678673 -2.369180 -0.930990

O 0 -4.553990 -2.193627 -1.898935

O 0 -3.754969 -1.803326 0.161397

H 0 -4.686050 -4.408655 -2.826842

H 0 -2.606941 -3.118080 -3.415424

H 0 -1.615793 -4.442561 -2.831001

H 0 -1.626906 -2.728067 -1.120254

H 0 -2.526540 -4.083902 -0.494940

C -1 2.462891 -1.588929 -6.455099

C 0 2.749251 -1.444322 -4.975042

C 0 1.718342 -1.573752 -4.033888

C 0 4.043974 -1.195664 -4.502067

C 0 1.966926 -1.452440 -2.666461

C 0 4.306310 -1.091291 -3.133189

C 0 3.265918 -1.217101 -2.208877

H 0 3.246364 -1.123513 -7.059719

H 0 2.405488 -2.644923 -6.745616

H 0 0.707384 -1.765297 -4.380273

H 0 4.857753 -1.081362 -5.213943

H 0 1.150666 -1.530949 -1.955448

H 0 5.316933 -0.884447 -2.790107

H 0 3.454079 -1.114102 -1.144493

C -1 2.556987 4.064066 -4.609227

C 0 2.967567 3.701962 -3.177422

C 0 3.654943 2.339259 -3.094199

N 0 4.106231 2.049808 -1.718919

H 0 3.430502 4.122722 -5.268206

H 0 1.877901 3.312241 -5.022564

H 0 2.094608 3.698035 -2.514433

H 0 3.647018 4.472693 -2.784734

H 0 4.491511 2.299963 -3.808556

H 0 2.956516 1.549233 -3.385363

H 0 4.821420 2.719302 -1.434971

H 0 4.568842 1.142617 -1.701190

C -1 -2.421903 0.949303 -5.153803

C 0 -1.713588 1.025709 -3.794732

C 0 -1.727903 -0.345921 -3.115790

C 0 -0.289064 1.578176 -3.916811

H 0 -1.919240 0.232322 -5.814898

H 0 -2.286967 1.711561 -3.159255

H 0 -2.755441 -0.681398 -2.953446

H 0 -1.232286 -1.093299 -3.745413

H 0 -1.210234 -0.322935 -2.152689

H 0 0.332796 0.937692 -4.553037

H 0 -0.293935 2.584046 -4.351892

H 0 0.193975 1.634822 -2.935574

C -1 -8.784059 -2.890610 -3.161060

C 0 -9.019154 -3.874528 -2.017940

S 0 -9.994068 -3.202757 -0.617822

C 0 -8.784076 -2.024205 0.073479

H 0 -9.729648 -2.512543 -3.555725

H 0 -8.193515 -2.031128 -2.831724

H 0 -8.073866 -4.253154 -1.613912

H 0 -9.592802 -4.738805 -2.367015

H 0 -8.747151 -1.101748 -0.506977

H 0 -9.107176 -1.783174 1.087228

H 0 -7.796892 -2.485326 0.135188

C -1 -11.413968 -0.217155 -4.324802

C 0 -10.470339 0.430447 -3.333926

C 0 -9.209775 0.900246 -3.714157

C 0 -10.843192 0.601854 -1.992124

C 0 -8.361578 1.555875 -2.814488

C 0 -10.014796 1.250835 -1.081703

C 0 -8.774563 1.753167 -1.493143

O 0 -8.053832 2.459134 -0.566605

H 0 -11.899092 -1.097158 -3.891028

H 0 -12.206180 0.477513 -4.627923

H 0 -8.877190 0.767450 -4.740011

H 0 -11.799155 0.215131 -1.651423

H 0 -7.398386 1.925760 -3.149266

H 0 -10.312529 1.383064 -0.047199

H 0 -7.139515 2.638980 -0.860556

C -1 -5.820148 4.559253 -3.594181

C 0 -4.546363 3.995741 -2.937008

C 0 -4.883696 2.765069 -2.132213

O -1 -5.248247 2.993751 -0.889899

O 0 -4.877798 1.638543 -2.649367

H 0 -6.585200 4.808673 -2.852832

H 0 -4.101981 4.743195 -2.276625

H 0 -3.818004 3.712219 -3.701119

C -1 -9.495878 6.948241 -2.625195

C 0 -9.716698 5.611563 -1.930152

C 0 -10.321177 4.574759 -2.876932

O 0 -8.445439 5.201831 -1.424718

H 0 -8.830664 6.818510 -3.485391

H 0 -10.412946 5.766142 -1.087711

H 0 -11.264146 4.940841 -3.297483

H 0 -9.630181 4.369776 -3.700590

H 0 -10.525782 3.630791 -2.365914

H 0 -8.538963 4.345129 -0.976762

N 0 -7.784719 5.182813 5.898798

C 0 -6.901740 4.152939 5.323678

C 0 -5.506790 4.637597 4.929933

O 0 -4.590486 3.826376 4.788359

C 0 -7.675057 3.674575 4.075823

C 0 -9.141377 3.893494 4.462521

C 0 -9.101764 5.216275 5.238273

H 0 -6.752337 3.344945 6.046598

H 0 -7.424397 2.648093 3.809097

H 0 -7.417129 4.312551 3.222782

H 0 -9.483190 3.088629 5.122323

H 0 -9.812160 3.934143 3.600930

H 0 -9.173582 6.074806 4.556714

H 0 -9.908036 5.301838 5.969838

N 0 -5.408833 5.956279 4.653100

C -1 -4.224757 6.582919 4.088958

C 0 -4.431321 7.028844 2.635027

C 0 -4.754599 5.894552 1.648942

C 0 -5.078901 6.456046 0.259319

C 0 -3.626002 4.859145 1.576054

H 0 -3.419173 5.851599 4.161407

H 0 -3.519472 7.546005 2.305423

H 0 -5.236236 7.776989 2.604047

H 0 -5.655934 5.382955 2.014522

H 0 -5.873011 7.209525 0.302112

H 0 -4.193623 6.925940 -0.188062

H 0 -6.225904 6.520116 4.836365

H 0 -5.416856 5.657866 -0.406164

H 0 -3.509508 4.319336 2.518302

H 0 -3.840555 4.129551 0.792170

H 0 -2.667163 5.335778 1.338100

C -1 -0.160292 5.153808 5.413083

C 0 -1.079185 3.942496 5.230887

S 0 -0.912395 3.095417 3.611636

C 0 0.696895 2.273267 3.849030

H 0 0.894531 4.866775 5.356384

H 0 -0.346154 5.903367 4.638329

H 0 -2.134725 4.223763 5.283294

H 0 -0.910390 3.198503 6.017438

H 0 1.523760 2.987111 3.882218

H 0 0.684031 1.677733 4.765190

H 0 0.841606 1.609518 2.996715

C -1 -0.793784 9.087910 -2.939858

C 0 -0.443735 7.593181 -2.853442

C 0 -1.359364 6.869443 -1.847668

C 0 1.041896 7.406612 -2.517981

C 0 -1.154138 5.352785 -1.764218

H 0 -0.623713 9.581447 -1.975411

H 0 -0.623781 7.145977 -3.842988

H 0 -2.403822 7.075342 -2.117075

H 0 -1.211935 7.311288 -0.851932

H 0 1.675820 7.935089 -3.238340

H 0 1.265559 7.806352 -1.521094

H 0 1.335410 6.353126 -2.526253

H 0 -0.163909 5.094745 -1.376794

H 0 -1.891835 4.897637 -1.096250

H 0 -1.258650 4.881370 -2.748547

C -1 -8.525003 6.609942 7.776954

C 0 -7.496893 5.679192 7.149794

O 0 -6.448201 5.391289 7.713989

H 0 -9.387358 6.040330 8.140948

H 0 -8.053603 7.106336 8.624335

H 0 -1.845099 9.235915 -3.208497

H 0 -0.177914 9.597507 -3.688286

H 0 2.046047 5.031048 -4.641577

H 0 3.197814 -2.014735 6.964349

H 0 0.200526 1.259200 11.417765

H 0 -1.950567 -8.366148 11.637722

H 0 -2.148956 -4.119269 13.244672

H 0 -3.386385 -7.997536 4.022605

H 0 -8.088481 -7.435822 0.028442

H 0 -8.231135 -3.374032 -3.974783

H 0 -3.685951 -5.732987 -2.207820

H 0 -3.652053 -5.332440 -3.928453

H 0 0.125238 -7.348267 -0.735986

H 0 1.507105 -1.126820 -6.720930

H 0 -2.424017 1.921941 -5.659539

H 0 -3.461826 0.633735 -5.028369

H 0 -5.580280 5.467203 -4.154834

H 0 -9.030617 7.661950 -1.939706

H 0 -10.443683 7.365416 -2.978320

H 0 -6.240373 3.833595 -4.297943

H 0 -10.885997 -0.531326 -5.229282

H 0 -8.895589 7.356113 7.067970

H 0 -3.946927 7.442475 4.709932

H 0 -0.328654 5.616694 6.392451

O 0 7.114190 0.837863 -1.717688

O 0 -6.201261 -0.292719 -1.426104

H 0 7.894248 0.432915 -1.298302

H 0 6.984267 1.649363 -1.199763

H 0 -5.713992 0.429175 -1.911853

H 0 -7.105370 -0.283022 -1.768701

C -1 5.111302 1.406958 8.853951

C 0 4.465860 2.715677 8.379240

C 0 5.414618 3.901759 8.587873

C 0 4.025497 2.621235 6.913604

H 0 4.431475 0.558662 8.733099

H 0 6.017095 1.191603 8.274241

H 0 3.568826 2.891359 8.988757

H 0 6.329255 3.773601 7.996129

H 0 4.948512 4.843968 8.280390

H 0 5.708751 3.998029 9.638516

H 0 3.524250 3.539104 6.587297

H 0 4.887256 2.459355 6.256293

H 0 3.330813 1.790462 6.756065

C -1 -7.140091 -6.831043 9.563921

C 0 -6.654940 -6.017875 8.381526

C 0 -5.726442 -4.982585 8.562422

C 0 -7.126710 -6.264635 7.084584

C 0 -5.293747 -4.214624 7.480186

C 0 -6.692582 -5.500999 5.998614

C 0 -5.772118 -4.468755 6.192180

H 0 -6.374800 -6.875132 10.342841

H 0 -8.036543 -6.380044 10.007730

H 0 -5.325713 -4.805368 9.555516

H 0 -7.840183 -7.069622 6.923248

H 0 -4.552212 -3.436783 7.635554

H 0 -7.064007 -5.708754 4.999275

H 0 -5.414778 -3.888953 5.346410

H 0 -7.403977 -7.850182 9.264081

H 0 5.396416 1.464395 9.909884

H 0 7.788805 -0.475978 2.221587

H 0 -5.263199 -1.516657 -1.640522

C -1 6.647542 5.725766 0.043034

C 0 7.086590 4.556841 -0.806847

O 0 6.802717 3.393538 -0.487060

H 0 7.062402 5.600767 1.046478

N 0 7.806588 4.844394 -1.915834

C 0 8.325498 3.829390 -2.820257

C 0 9.714018 3.287054 -2.468659

O 0 10.532617 3.047186 -3.349052

H 0 7.646486 2.969580 -2.816144

H 0 8.079172 5.802475 -2.075336

N 0 9.942872 3.067450 -1.138502

C -1 11.056571 2.194052 -0.766063

C 0 10.605338 0.761788 -0.972448

O 0 9.601344 0.353929 -0.368086

H 0 11.929453 2.477560 -1.348902

H 0 9.117354 2.967375 -0.556242

N 0 11.275078 -0.000331 -1.845768

C -1 10.757114 -1.336919 -2.119974

C 0 11.773415 -1.921012 -3.108340

C 0 12.273149 -0.680117 -3.864307

C 0 12.354501 0.394778 -2.771705

H 0 11.330876 -2.675958 -3.761904

H 0 12.600842 -2.390065 -2.564324

H 0 11.538401 -0.376377 -4.617369

H 0 13.231041 -0.831365 -4.367332

H 0 13.323985 0.365059 -2.258338

H 0 12.173914 1.398222 -3.158513

H 0 9.755443 -1.259404 -2.560752

H 0 5.558935 5.696554 0.137746

H 0 6.949324 6.696519 -0.356219

H 0 8.380557 4.232946 -3.831332

H 0 11.272279 2.339892 0.295164

H 0 10.660141 -1.906769 -1.193180

N 0 -4.885629 1.196945 0.791209

C 0 -3.464472 1.143330 1.220875

C 0 -2.528707 1.255684 0.012554

C 0 -1.088730 0.900427 0.313685

C 0 -0.066585 1.833041 0.094757

C 0 1.273172 1.511660 0.318025

C 0 1.598000 0.228934 0.796269

C 0 0.594839 -0.710150 1.025381

C 0 -0.739648 -0.378420 0.774928

O 0 2.909005 -0.083041 1.054913

O 0 2.268485 2.436271 0.172171

H 0 3.397429 0.756672 1.006314

H 0 -1.516654 -1.120492 0.931709

H 0 -3.277556 0.201917 1.738277

H 0 -2.572484 2.273746 -0.387007

H 0 -0.299413 2.836665 -0.246360

H 0 0.868203 -1.688375 1.403999

H -1 -5.128396 2.155726 -0.172228

H 0 -2.910465 0.594711 -0.771803

H 0 -3.276728 1.952845 1.928433

H 0 -5.483340 1.314010 1.613085

H 0 2.910734 2.205091 -0.586350

H 0 -5.148906 0.295940 0.386027

C 0 -0.995499 -0.470858 6.122259

C 0 -1.439980 -0.548127 4.800823

C 0 -2.800204 -0.665870 4.535165

C 0 -3.746339 -0.667701 5.570355

C 0 -5.214883 -0.647349 5.186641

C 0 -5.480446 0.632548 4.411099

C 0 -3.290202 -0.598070 6.889813

C 0 -1.923115 -0.513475 7.163076

O 0 -6.183242 0.670434 3.412441

H 0 -0.734987 -0.498794 3.977479

H 0 -3.144463 -0.742009 3.510099

H 0 -5.019444 1.557696 4.809411

H 0 -3.996898 -0.605349 7.712144

H 0 -1.586300 -0.476309 8.194190

H 0 0.063032 -0.380734 6.335621

H 0 -5.423451 -1.472768 4.499688

C 0 -6.206557 -0.689992 6.361700

H 0 -6.070670 -1.600367 6.950031

H 0 -6.086286 0.174181 7.024244

H 0 -7.231149 -0.686544 5.980295

**TS2R** (-8045.68229)

C -1 7.281803 0.461211 1.969268

C 0 6.069563 0.749654 2.859957

C 0 6.394943 0.517160 4.339315

C 0 5.544566 2.173968 2.631445

H 0 7.020492 0.529502 0.909925

H 0 8.095791 1.172696 2.155145

H 0 5.272793 0.053253 2.575436

H 0 7.187477 1.196690 4.678034

H 0 5.519764 0.683829 4.974935

H 0 6.742255 -0.507654 4.511270

H 0 4.601916 2.349251 3.162272

H 0 6.267151 2.917433 2.992092

H 0 5.394291 2.381066 1.565555

C -1 2.868018 -2.655294 6.140052

C 0 2.863462 -3.240364 4.721438

C 0 1.432820 -3.403936 4.189252

C 0 3.715914 -2.388195 3.773028

H 0 2.434958 -1.647799 6.148083

H 0 2.278634 -3.273942 6.825287

H 0 3.318273 -4.240133 4.776119

H 0 0.947886 -2.430607 4.061887

H 0 1.423241 -3.907538 3.216959

H 0 0.817448 -3.994854 4.876868

H 0 3.289030 -1.387195 3.645852

H 0 4.736116 -2.266688 4.152199

H 0 3.782973 -2.843315 2.779072

C -1 0.465614 0.418026 10.771544

C 0 -0.215091 0.384685 9.392363

C 0 -1.668623 -0.088383 9.507027

C 0 -0.148955 1.747913 8.693782

H 0 -0.038208 1.135255 11.431047

H 0 0.427603 -0.564496 11.254196

H 0 0.327113 -0.340261 8.766943

H 0 -2.246492 0.592979 10.142938

H 0 -2.153876 -0.110320 8.524966

H 0 -1.733272 -1.091100 9.936881

H 0 -0.679989 2.508670 9.278234

H 0 0.883660 2.082816 8.567027

H 0 -0.610758 1.704888 7.702423

C -1 -3.085944 -4.133043 12.675998

C 0 -3.266648 -5.451979 11.903496

O 0 -4.290353 -5.668435 11.261114

C 0 -2.707513 -3.012758 11.696378

H 0 -4.042314 -3.916370 13.157898

H 0 -1.754111 -3.226546 11.204304

H 0 -3.467376 -2.920167 10.916776

H 0 -2.622096 -2.053335 12.214029

N 0 -2.221921 -6.343231 11.938382

C -1 -2.114052 -7.479941 11.011978

C 0 -1.029659 -7.250779 9.941141

C 0 -1.089052 -5.885784 9.285781

C 0 -2.239124 -5.439561 8.618670

C 0 0.016707 -5.029228 9.353428

C 0 -2.270862 -4.178082 8.026048

C 0 -0.011676 -3.763486 8.763946

C 0 -1.156671 -3.338177 8.091936

H 0 -3.101916 -7.577991 10.559177

H 0 -1.125769 -8.044239 9.188685

H 0 -0.039950 -7.380580 10.395284

H 0 -3.128250 -6.061081 8.581595

H 0 -1.374391 -6.038682 12.395686

H 0 0.913173 -5.359051 9.872703

H 0 -3.173744 -3.853171 7.519729

H 0 0.853759 -3.111463 8.834281

H 0 -1.189628 -2.351218 7.645368

C -1 0.152873 -6.400927 -0.188004

C 0 0.976713 -5.369089 -0.966276

C 0 0.061941 -4.417190 -1.745781

C 0 1.911594 -4.599877 -0.025826

H 0 0.796987 -7.107078 0.347414

H 0 -0.479864 -5.898728 0.554446

H 0 1.598064 -5.908006 -1.695956

H 0 -0.580684 -3.846431 -1.065838

H 0 0.642015 -3.703208 -2.336941

H 0 -0.588805 -4.974696 -2.427298

H 0 1.332955 -4.102799 0.759992

H 0 2.624877 -5.272515 0.462874

H 0 2.478020 -3.831846 -0.561505

C -1 -3.596870 -6.933987 3.872027

C 0 -2.362617 -6.319846 3.213846

S 0 -2.478666 -4.526548 2.821250

C 0 -2.614380 -3.824770 4.502791

H 0 -3.801502 -6.482521 4.847555

H 0 -4.485420 -6.792106 3.250815

H 0 -2.162042 -6.791733 2.247050

H 0 -1.471674 -6.464396 3.834307

H 0 -1.863994 -4.259176 5.166955

H 0 -2.416612 -2.753600 4.420437

H 0 -3.611955 -3.974153 4.923809

C -1 -8.592941 -6.748020 0.713102

C 0 -8.169795 -5.554627 1.539706

C 0 -6.868537 -5.041839 1.495170

C 0 -9.092151 -4.898079 2.368555

C 0 -6.495860 -3.907036 2.222985

C 0 -8.741873 -3.764308 3.093420

C 0 -7.444097 -3.239649 3.005833

O 0 -7.165935 -2.116120 3.720547

H 0 -9.310954 -6.446452 -0.058387

H 0 -7.735584 -7.212135 0.216659

H 0 -6.115490 -5.541652 0.889645

H 0 -10.110836 -5.271388 2.428129

H 0 -5.477773 -3.542051 2.166025

H 0 -9.464920 -3.252468 3.719717

H 0 -6.414378 -1.604334 3.334976

C -1 -3.717861 -4.902614 -2.923860

C 0 -3.871447 -3.798882 -1.885015

C 0 -5.336666 -3.413992 -1.668227

C 0 -5.473882 -2.309336 -0.652805

O 0 -4.736645 -2.200694 0.315174

O 0 -6.465806 -1.462202 -0.912736

H 0 -4.189520 -4.622549 -3.872551

H 0 -3.312104 -2.912142 -2.205864

H 0 -3.438636 -4.101894 -0.926568

H 0 -5.901644 -4.273458 -1.283696

H 0 -5.818115 -3.109740 -2.601450

C -1 2.463013 -1.588832 -6.455089

C 0 2.904179 -1.394958 -5.018585

C 0 1.999117 -1.581257 -3.965072

C 0 4.222422 -1.044672 -4.698313

C 0 2.391450 -1.426385 -2.634685

C 0 4.632211 -0.905556 -3.369380

C 0 3.716174 -1.101539 -2.332733

H 0 3.082189 -1.007758 -7.144893

H 0 2.539239 -2.641745 -6.753107

H 0 0.971350 -1.841268 -4.192949

H 0 4.938440 -0.879122 -5.499829

H 0 1.669043 -1.551510 -1.834810

H 0 5.651787 -0.613244 -3.134464

H 0 4.026016 -0.991470 -1.297653

C -1 2.557165 4.063992 -4.609097

C 0 2.939400 3.749044 -3.159727

C 0 3.670493 2.413651 -3.025821

N 0 4.052624 2.154860 -1.623091

H 0 3.443946 4.120683 -5.250112

H 0 1.901501 3.287811 -5.017322

H 0 2.046113 3.722622 -2.525022

H 0 3.576892 4.552826 -2.763626

H 0 4.546611 2.392808 -3.690526

H 0 3.017124 1.596583 -3.345116

H 0 4.777973 2.807328 -1.324787

H 0 4.476531 1.230947 -1.554264

C -1 -2.421988 0.948792 -5.153623

C 0 -1.595362 0.612551 -3.908457

C 0 -1.554386 -0.904850 -3.694803

C 0 -0.187735 1.215292 -3.991693

H 0 -1.956260 0.528418 -6.052765

H 0 -2.098708 1.063927 -3.040923

H 0 -2.559574 -1.299922 -3.510028

H 0 -1.162643 -1.407768 -4.586428

H 0 -0.923028 -1.176950 -2.844742

H 0 0.362891 0.811386 -4.849220

H 0 -0.234457 2.304519 -4.104266

H 0 0.393006 0.991126 -3.092810

C -1 -8.784728 -2.890160 -3.161155

C 0 -9.147525 -3.969200 -2.142810

S 0 -10.476209 -3.495057 -0.972415

C 0 -9.597447 -2.225999 0.001235

H 0 -9.665290 -2.596997 -3.736332

H 0 -8.377858 -2.005598 -2.670321

H 0 -8.274935 -4.267658 -1.552542

H 0 -9.524199 -4.866757 -2.643511

H 0 -8.680957 -2.640058 0.423262

H 0 -9.378136 -1.346542 -0.603681

H 0 -10.260681 -1.941103 0.819589

C -1 -11.412801 -0.217819 -4.324409

C 0 -10.658898 0.355501 -3.147141

C 0 -9.274286 0.556996 -3.212225

C 0 -11.308509 0.697323 -1.956171

C 0 -8.562139 1.087380 -2.139240

C 0 -10.615673 1.252320 -0.879819

C 0 -9.241274 1.458820 -0.974192

O 0 -8.582081 2.022309 0.104102

H 0 -11.072708 -1.230997 -4.561766

H 0 -12.484974 -0.269427 -4.119656

H 0 -8.736454 0.278932 -4.114499

H 0 -12.379306 0.537025 -1.869842

H 0 -7.485117 1.187374 -2.197337

H 0 -11.128683 1.535855 0.032649

H 0 -7.870470 2.609131 -0.245877

C -1 -5.818549 4.555048 -3.596584

C 0 -4.910311 3.666842 -2.741698

C 0 -5.607780 3.041755 -1.555668

O 0 -6.623761 3.514739 -1.035692

O 0 -5.033007 1.936460 -1.124283

H 0 -6.260575 5.362316 -3.009107

H 0 -4.067804 4.245923 -2.341220

H 0 -4.461329 2.856151 -3.324164

C -1 -9.497225 6.950430 -2.623752

C 0 -9.130753 6.230707 -1.329662

C 0 -9.762090 4.844431 -1.254108

O 0 -7.704652 6.178312 -1.223461

H 0 -9.186234 6.354499 -3.488748

H 0 -9.501909 6.829484 -0.482726

H 0 -10.851650 4.907990 -1.333035

H 0 -9.404399 4.211720 -2.073647

H 0 -9.521507 4.346143 -0.311483

H 0 -7.417629 5.255288 -1.133896

N 0 -7.430941 4.929135 6.322334

C 0 -6.306240 4.020498 6.028074

C 0 -5.064230 4.687133 5.431583

O 0 -3.957557 4.179114 5.593169

C 0 -6.929190 2.996268 5.055352

C 0 -8.399917 2.945671 5.482937

C 0 -8.713413 4.409816 5.814820

H 0 -5.961798 3.544007 6.950969

H 0 -6.428937 2.026689 5.088710

H 0 -6.849781 3.375138 4.031047

H 0 -8.509993 2.329079 6.381158

H 0 -9.057952 2.540143 4.711011

H 0 -9.025496 4.959619 4.916681

H 0 -9.504612 4.511647 6.560464

N 0 -5.299323 5.764998 4.637813

C -1 -4.225113 6.582889 4.089298

C 0 -4.427300 6.931982 2.613225

C 0 -4.267963 5.768934 1.621412

C 0 -4.686738 6.218398 0.218101

C 0 -2.831769 5.234271 1.603436

H 0 -3.302982 6.024529 4.253505

H 0 -3.707013 7.719314 2.351177

H 0 -5.422299 7.380243 2.486294

H 0 -4.935565 4.956922 1.941039

H 0 -5.739062 6.509816 0.166194

H 0 -4.079169 7.070488 -0.109738

H 0 -6.221401 6.173251 4.705786

H 0 -4.535041 5.415542 -0.507781

H 0 -2.499066 4.903903 2.590011

H 0 -2.742159 4.378523 0.925955

H 0 -2.136096 6.006749 1.254975

C -1 -0.159662 5.153531 5.413044

C 0 0.997952 5.800029 4.655927

S 0 1.895908 4.701222 3.484624

C 0 0.659936 4.556023 2.150557

H 0 -0.945503 4.790454 4.744281

H 0 0.185239 4.298649 6.001405

H 0 1.768728 6.138779 5.355193

H 0 0.662442 6.681493 4.098127

H 0 -0.284536 4.176155 2.541353

H 0 0.490230 5.522223 1.666879

H 0 1.051630 3.848295 1.416091

C -1 -0.794080 9.088027 -2.940088

C 0 -0.415168 7.598249 -2.893720

C 0 -1.332945 6.824395 -1.928157

C 0 1.067708 7.426469 -2.537935

C 0 -1.114411 5.306953 -1.910706

H 0 -0.653063 9.554048 -1.957363

H 0 -0.570302 7.179811 -3.899988

H 0 -2.376940 7.032478 -2.195715

H 0 -1.197726 7.223753 -0.912797

H 0 1.705193 7.985235 -3.231555

H 0 1.268102 7.800739 -1.526299

H 0 1.377840 6.378075 -2.571828

H 0 -0.134514 5.032646 -1.508459

H 0 -1.864503 4.812203 -1.285183

H 0 -1.188938 4.885149 -2.920392

C -1 -8.525007 6.609724 7.776791

C 0 -7.277971 5.862240 7.322314

O 0 -6.175985 6.076886 7.814052

H 0 -9.165349 5.954319 8.377823

H 0 -8.205125 7.447958 8.394156

H 0 -1.842528 9.223278 -3.225293

H 0 -0.173810 9.632763 -3.659110

H 0 2.029405 5.019691 -4.680213

H 0 3.885688 -2.578453 6.537454

H 0 1.515398 0.716936 10.693958

H 0 -1.903767 -8.397633 11.571548

H 0 -2.325188 -4.235325 13.459182

H 0 -3.443203 -8.008205 4.025915

H 0 -9.079182 -7.512873 1.328333

H 0 -8.026852 -3.267436 -3.859257

H 0 -4.182623 -5.835293 -2.587390

H 0 -2.662707 -5.105648 -3.128839

H 0 -0.500853 -6.976368 -0.852958

H 0 1.420622 -1.286267 -6.594314

H 0 -2.505925 2.031544 -5.299721

H 0 -3.435825 0.538125 -5.081964

H 0 -5.243767 4.991729 -4.417681

H 0 -8.989237 7.917539 -2.676848

H 0 -10.577396 7.120464 -2.688643

H 0 -6.636595 3.969917 -4.027657

H 0 -11.269877 0.391872 -5.223348

H 0 -9.120388 6.974261 6.934708

H 0 -4.145464 7.508853 4.675428

H 0 -0.620489 5.877424 6.094155

O 0 7.164897 0.864140 -1.770564

O 0 -6.354728 0.637081 0.729818

H 0 7.912806 0.468659 -1.288242

H 0 6.971582 1.670918 -1.266187

H 0 -7.263430 0.994673 0.820607

H 0 -5.978074 0.383696 1.627653

C -1 5.110890 1.406246 8.853588

C 0 3.719507 1.874452 8.409144

C 0 3.544339 3.378870 8.646958

C 0 3.447302 1.519066 6.942463

H 0 5.228233 0.325544 8.718649

H 0 5.892793 1.900370 8.263969

H 0 2.978736 1.345249 9.024882

H 0 4.268116 3.948624 8.051655

H 0 2.542515 3.715988 8.361073

H 0 3.698897 3.638044 9.699890

H 0 2.430708 1.793574 6.643772

H 0 4.139612 2.050859 6.279140

H 0 3.572404 0.445405 6.764146

C -1 -7.139889 -6.830958 9.563991

C 0 -6.779724 -5.894217 8.430615

C 0 -6.076108 -4.708785 8.688914

C 0 -7.173416 -6.156696 7.111270

C 0 -5.821900 -3.795653 7.664408

C 0 -6.904163 -5.253239 6.080635

C 0 -6.237650 -4.057026 6.356281

H 0 -6.356749 -6.833778 10.326717

H 0 -8.072295 -6.514018 10.047406

H 0 -5.725234 -4.513829 9.696758

H 0 -7.709294 -7.077038 6.890838

H 0 -5.306771 -2.866314 7.895430

H 0 -7.231498 -5.464590 5.067341

H 0 -6.087561 -3.327157 5.567460

H 0 -7.290423 -7.853502 9.204580

H 0 5.291167 1.637782 9.908579

H 0 7.679814 -0.543613 2.144227

C -1 6.647814 5.725777 0.042991

C 0 7.061987 4.571783 -0.837140

O 0 6.708094 3.412412 -0.575975

H 0 7.012189 5.537252 1.055964

N 0 7.838207 4.866136 -1.905181

C 0 8.352209 3.865554 -2.827496

C 0 9.732361 3.306083 -2.472303

O 0 10.559242 3.077024 -3.347491

H 0 7.664874 3.013175 -2.843549

H 0 8.161669 5.815755 -2.012587

N 0 9.946221 3.068098 -1.142989

C -1 11.057020 2.194005 -0.766023

C 0 10.606795 0.761181 -0.969368

O 0 9.608326 0.351818 -0.357017

H 0 11.930905 2.476673 -1.347767

H 0 9.116174 2.961374 -0.569442

N 0 11.271968 0.001358 -1.848138

C -1 10.756968 -1.336997 -2.120003

C 0 11.769176 -1.917702 -3.114542

C 0 12.263262 -0.674880 -3.871001

C 0 12.348435 0.398046 -2.776909

H 0 11.324372 -2.672213 -3.767064

H 0 12.600164 -2.386437 -2.575704

H 0 11.524334 -0.370969 -4.619911

H 0 13.218916 -0.823779 -4.378971

H 0 13.319618 0.366723 -2.266752

H 0 12.167691 1.402418 -3.161199

H 0 9.752263 -1.262037 -2.554063

H 0 5.556249 5.751612 0.096056

H 0 7.015100 6.694754 -0.301692

H 0 8.420046 4.287684 -3.830405

H 0 11.270667 2.341687 0.295365

H 0 10.667525 -1.907471 -1.192766

N 0 -2.944165 -0.822866 2.036516

C 0 -2.946320 0.013349 0.827861

C 0 -2.069131 -0.527268 -0.304940

C 0 -0.604001 -0.340268 -0.004727

C 0 0.006382 0.900211 -0.249482

C 0 1.332405 1.134392 0.101329

C 0 2.064311 0.107586 0.731329

C 0 1.474945 -1.126357 0.976569

C 0 0.145266 -1.347642 0.609471

O 0 3.362984 0.325655 1.101117

O 0 1.919923 2.353337 -0.085174

H 0 3.511009 1.285013 1.079691

H 0 -0.310979 -2.312327 0.811013

H 0 -2.586753 1.004443 1.094444

H 0 -2.343855 0.021257 -1.210009

H 0 -0.547426 1.707239 -0.721166

H 0 2.057195 -1.896827 1.464860

H 0 -3.324037 -1.736304 1.785266

H 0 -2.309953 -1.579227 -0.478837

H 0 -3.979416 0.085933 0.503369

H 0 -2.000582 -0.930200 2.405794

H 0 2.760814 2.264917 -0.676425

H 0 -5.531380 1.533606 -0.341075

H 0 -6.471256 -0.747686 -0.214262

C 0 0.625242 0.374139 4.616420

C 0 0.228156 1.281760 3.635739

C 0 -1.127335 1.547805 3.431274

C 0 -2.111316 0.914434 4.199660

C 0 -3.597613 1.078507 3.932314

C 0 -4.211735 -0.247646 3.448030

C 0 -1.694461 0.023668 5.201521

C 0 -0.342486 -0.252261 5.404047

O 0 -5.372079 -0.278584 2.941809

H 0 0.971972 1.780972 3.027856

H 0 -1.413831 2.248886 2.656791

H 0 -3.912473 -1.102853 4.073708

H 0 -2.437762 -0.458701 5.831670

H 0 -0.051326 -0.961345 6.171759

H 0 1.677693 0.159879 4.764234

C 0 -4.005090 2.278381 3.077752

H 0 -3.706205 3.193612 3.582009

H 0 -3.567002 2.263097 2.082188

H 0 -5.088460 2.289449 2.949358

H 0 -4.077618 1.229075 4.913332

**Int3R** (-8045.698678)

C -1 7.282113 0.461183 1.969153

C 0 6.015249 0.807320 2.762793

C 0 6.281601 0.790711 4.272739

C 0 5.437132 2.162981 2.335416

H 0 7.075261 0.401625 0.897330

H 0 8.060218 1.220576 2.111274

H 0 5.259084 0.041834 2.543596

H 0 7.061712 1.516002 4.536434

H 0 5.381800 1.052252 4.840010

H 0 6.616937 -0.196094 4.609110

H 0 4.477680 2.363117 2.827732

H 0 6.120417 2.979598 2.601132

H 0 5.304144 2.223225 1.250230

C -1 2.867936 -2.655328 6.140120

C 0 2.869650 -2.487302 4.613662

C 0 1.742431 -3.300062 3.963369

C 0 2.779396 -1.010679 4.210299

H 0 1.933556 -2.276595 6.573778

H 0 2.958580 -3.708010 6.428143

H 0 3.825567 -2.880350 4.238112

H 0 0.761721 -2.932798 4.287286

H 0 1.780052 -3.221917 2.871447

H 0 1.803288 -4.360949 4.228480

H 0 1.819244 -0.578772 4.513166

H 0 3.572387 -0.424325 4.683581

H 0 2.869972 -0.877651 3.128696

C -1 0.465953 0.417432 10.771273

C 0 -0.236621 0.712493 9.438766

C 0 -1.671807 0.173470 9.430329

C 0 -0.218143 2.213930 9.128524

H 0 -0.048192 0.918624 11.600261

H 0 0.472792 -0.657091 10.985708

H 0 0.317539 0.196358 8.642537

H 0 -2.278542 0.674473 10.194550

H 0 -2.148866 0.341794 8.459578

H 0 -1.696228 -0.900906 9.636358

H 0 -0.770509 2.772477 9.893758

H 0 0.803736 2.607214 9.103818

H 0 -0.689524 2.421126 8.163133

C -1 -3.085917 -4.133049 12.675978

C 0 -3.259838 -5.441365 11.884370

O 0 -4.277851 -5.648853 11.229411

C 0 -2.910762 -2.958561 11.702440

H 0 -3.992829 -4.001658 13.273037

H 0 -2.008286 -3.082844 11.097847

H 0 -3.762633 -2.906084 11.020642

H 0 -2.838188 -2.010567 12.242990

N 0 -2.223810 -6.341144 11.934059

C -1 -2.114084 -7.479956 11.012046

C 0 -1.028086 -7.249156 9.944054

C 0 -1.155293 -5.905109 9.261342

C 0 -2.272191 -5.602018 8.471615

C 0 -0.180042 -4.918737 9.447871

C 0 -2.407818 -4.347547 7.882452

C 0 -0.314515 -3.656473 8.865142

C 0 -1.433134 -3.368484 8.083764

H 0 -3.098959 -7.577142 10.552285

H 0 -1.091043 -8.066111 9.214174

H 0 -0.037946 -7.326154 10.409747

H 0 -3.054984 -6.341410 8.330372

H 0 -1.377727 -6.038910 12.395190

H 0 0.693111 -5.141989 10.056520

H 0 -3.274774 -4.138570 7.268204

H 0 0.449281 -2.900123 9.023790

H 0 -1.551959 -2.387102 7.641805

C -1 0.152865 -6.400941 -0.188135

C 0 0.695523 -5.082786 -0.753263

C 0 0.446642 -4.977235 -2.262659

C 0 0.095309 -3.878320 -0.020632

H 0 0.352199 -6.485702 0.885800

H 0 -0.933436 -6.463384 -0.330060

H 0 1.782707 -5.067403 -0.590844

H 0 -0.628957 -4.960333 -2.475480

H 0 0.883049 -4.061070 -2.673039

H 0 0.879237 -5.828252 -2.799866

H 0 -0.993241 -3.843717 -0.138780

H 0 0.311491 -3.914659 1.052905

H 0 0.497948 -2.941940 -0.414910

C -1 -3.596874 -6.933996 3.872175

C 0 -4.397025 -5.667683 3.611631

S 0 -3.544467 -4.136885 4.133894

C 0 -2.225740 -4.015656 2.875739

H 0 -2.659784 -6.945939 3.306885

H 0 -3.352482 -7.028346 4.933744

H 0 -5.327968 -5.663747 4.183077

H 0 -4.666745 -5.571300 2.557953

H 0 -2.643590 -3.855493 1.879570

H 0 -1.614023 -3.149825 3.141542

H 0 -1.582271 -4.897700 2.881015

C -1 -8.593058 -6.747962 0.713155

C 0 -8.252291 -5.528413 1.529215

C 0 -7.024170 -4.876785 1.392230

C 0 -9.179030 -4.981801 2.428704

C 0 -6.729687 -3.711543 2.101022

C 0 -8.909268 -3.811875 3.130163

C 0 -7.689578 -3.146242 2.948017

O 0 -7.486079 -1.974842 3.603809

H 0 -9.032573 -7.538835 1.330397

H 0 -9.324665 -6.498296 -0.064769

H 0 -6.275338 -5.290522 0.719965

H 0 -10.140992 -5.469578 2.560783

H 0 -5.761007 -3.241289 2.000621

H 0 -9.639183 -3.379327 3.806183

H 0 -6.824014 -1.430986 3.099315

C -1 -3.717993 -4.902577 -2.924051

C 0 -3.540385 -3.949152 -1.744983

C 0 -4.772513 -3.080739 -1.499330

C 0 -4.638092 -2.180316 -0.293652

O 0 -3.587205 -2.141390 0.368660

O 0 -5.706427 -1.487420 -0.011882

H 0 -3.899333 -4.354449 -3.855468

H 0 -2.672542 -3.301967 -1.907551

H 0 -3.321862 -4.518904 -0.834940

H 0 -5.671439 -3.690553 -1.342946

H 0 -4.998396 -2.446812 -2.366489

C -1 2.463014 -1.588921 -6.455125

C 0 2.692536 -1.363368 -4.976308

C 0 1.620063 -1.397451 -4.074434

C 0 3.976643 -1.137506 -4.464013

C 0 1.821916 -1.223423 -2.704335

C 0 4.191378 -0.975987 -3.092675

C 0 3.112268 -1.026437 -2.206427

H 0 3.252894 -1.129681 -7.056324

H 0 2.449798 -2.659540 -6.692786

H 0 0.614252 -1.557803 -4.452772

H 0 4.820703 -1.088796 -5.147537

H 0 0.974139 -1.225145 -2.026780

H 0 5.193992 -0.782224 -2.719282

H 0 3.269581 -0.895981 -1.140571

C -1 2.557107 4.063974 -4.609107

C 0 3.030752 3.843960 -3.169676

C 0 3.512636 2.415021 -2.929216

N 0 3.956782 2.232763 -1.532727

H 0 3.363853 3.877018 -5.326341

H 0 1.731482 3.387519 -4.853758

H 0 2.221327 4.062016 -2.463427

H 0 3.844919 4.545775 -2.936152

H 0 4.309154 2.156467 -3.641227

H 0 2.694125 1.711292 -3.106819

H 0 4.795278 2.783219 -1.347044

H 0 4.226959 1.261428 -1.390535

C -1 -2.422004 0.948918 -5.153626

C 0 -1.994411 0.986916 -3.678028

C 0 -2.068299 -0.409809 -3.045708

C 0 -0.595001 1.597556 -3.524322

H 0 -1.738548 0.318581 -5.735481

H 0 -2.701013 1.631571 -3.135446

H 0 -3.090558 -0.803779 -3.076035

H 0 -1.427947 -1.117658 -3.584780

H 0 -1.739941 -0.395584 -2.002463

H 0 0.152588 0.999912 -4.057282

H 0 -0.563939 2.615095 -3.930405

H 0 -0.294377 1.644278 -2.473480

C -1 -8.783957 -2.890673 -3.160865

C 0 -9.183542 -4.002805 -2.195841

S 0 -10.393945 -3.493258 -0.918467

C 0 -9.349897 -2.325707 0.017221

H 0 -9.656595 -2.499483 -3.688915

H 0 -8.310302 -2.057961 -2.634024

H 0 -8.307696 -4.412388 -1.679081

H 0 -9.662109 -4.828896 -2.730948

H 0 -8.396829 -2.791894 0.264382

H 0 -9.187577 -1.402655 -0.538501

H 0 -9.873608 -2.099613 0.946677

C -1 -11.414110 -0.217157 -4.324866

C 0 -10.782630 0.537776 -3.178510

C 0 -9.924266 1.618894 -3.410189

C 0 -11.046355 0.193050 -1.847541

C 0 -9.336264 2.325948 -2.363036

C 0 -10.466537 0.888146 -0.786918

C 0 -9.595024 1.948487 -1.039698

O 0 -9.022222 2.586919 0.033913

H 0 -10.694198 -0.396888 -5.130143

H 0 -11.798258 -1.185443 -3.992636

H 0 -9.704120 1.916427 -4.432698

H 0 -11.691258 -0.653973 -1.633974

H 0 -8.672457 3.158560 -2.565527

H 0 -10.666253 0.603657 0.240748

H 0 -8.302812 3.177656 -0.288255

C -1 -5.818502 4.555093 -3.596515

C 0 -5.147340 3.555200 -2.658556

C 0 -5.940097 3.251096 -1.407161

O 0 -6.926645 3.913923 -1.057110

O 0 -5.488834 2.220945 -0.735965

H 0 -6.009773 5.510587 -3.104430

H 0 -4.163606 3.918202 -2.335597

H 0 -4.955789 2.597575 -3.154787

C -1 -9.497212 6.950522 -2.623698

C 0 -8.730375 7.115391 -1.305215

C 0 -9.409201 6.388858 -0.142124

O 0 -7.368829 6.709654 -1.436564

H 0 -9.588473 5.889060 -2.884123

H 0 -8.683033 8.186430 -1.068650

H 0 -10.421361 6.770505 0.028275

H 0 -9.488696 5.313971 -0.340371

H 0 -8.827655 6.521309 0.775716

H 0 -7.326128 5.738413 -1.384652

N 0 -7.877412 5.277878 5.790086

C 0 -7.047018 4.249355 5.135438

C 0 -5.635564 4.700932 4.772290

O 0 -4.728266 3.886361 4.601400

C 0 -7.844996 3.908383 3.859351

C 0 -9.298921 4.158145 4.272045

C 0 -9.200379 5.410598 5.153309

H 0 -6.936387 3.382388 5.792664

H 0 -7.640871 2.900010 3.502798

H 0 -7.559321 4.597602 3.056361

H 0 -9.672499 3.317291 4.866200

H 0 -9.967188 4.295556 3.418715

H 0 -9.248989 6.324509 4.545537

H 0 -9.994545 5.464339 5.901225

N 0 -5.486710 6.019909 4.535011

C -1 -4.225188 6.582950 4.089000

C 0 -4.169964 6.805058 2.573302

C 0 -4.056354 5.527372 1.725376

C 0 -4.258876 5.868027 0.245132

C 0 -2.708243 4.831931 1.951030

H 0 -3.435751 5.905163 4.418159

H 0 -3.309018 7.450812 2.348577

H 0 -5.063298 7.367488 2.271357

H 0 -4.849290 4.831979 2.029921

H 0 -5.261775 6.253928 0.038116

H 0 -3.533341 6.621299 -0.082781

H 0 -6.264569 6.625415 4.748629

H 0 -4.104073 4.979449 -0.373502

H 0 -2.601821 4.471314 2.976616

H 0 -2.599664 3.968521 1.290418

H 0 -1.875311 5.511906 1.731735

C -1 -0.160151 5.153827 5.413238

C 0 1.011861 4.194187 5.217783

S 0 1.093214 3.415277 3.558371

C 0 1.365491 4.901325 2.533873

H 0 -0.073003 6.024118 4.756728

H 0 -1.115721 4.669507 5.193823

H 0 0.949887 3.356882 5.918870

H 0 1.963663 4.699231 5.415312

H 0 0.472288 5.528924 2.494418

H 0 2.208144 5.483054 2.920116

H 0 1.604211 4.538925 1.533149

C -1 -0.794130 9.088007 -2.940142

C 0 -0.351324 7.617918 -2.867883

C 0 -1.252469 6.825146 -1.903867

C 0 1.131290 7.514176 -2.487721

C 0 -0.968923 5.321080 -1.839611

H 0 -0.686579 9.572826 -1.962339

H 0 -0.473174 7.178145 -3.869526

H 0 -2.297489 6.978571 -2.201323

H 0 -1.159253 7.256538 -0.896913

H 0 1.755071 8.094302 -3.176405

H 0 1.298774 7.905680 -1.476538

H 0 1.486731 6.479962 -2.506843

H 0 0.009000 5.098562 -1.401840

H 0 -1.720193 4.814979 -1.225375

H 0 -0.995855 4.868077 -2.837476

C -1 -8.525049 6.609704 7.776827

C 0 -7.543145 5.690610 7.059880

O 0 -6.488324 5.338505 7.575016

H 0 -9.398005 6.043989 8.120832

H 0 -8.016617 7.029066 8.644023

H 0 -1.843840 9.172912 -3.239749

H 0 -0.189587 9.650771 -3.659149

H 0 2.204938 5.089277 -4.757553

H 0 3.693894 -2.102357 6.599564

H 0 1.504256 0.766964 10.761668

H 0 -1.907144 -8.395518 11.576686

H 0 -2.235732 -4.196934 13.365347

H 0 -4.175486 -7.814963 3.571439

H 0 -7.706634 -7.160091 0.221641

H 0 -8.068787 -3.266899 -3.902411

H 0 -4.568616 -5.575414 -2.766736

H 0 -2.827562 -5.520973 -3.070533

H 0 0.603710 -7.266611 -0.684910

H 0 1.503514 -1.172664 -6.776746

H 0 -2.410312 1.950110 -5.597698

H 0 -3.431663 0.539280 -5.268189

H 0 -5.181162 4.734601 -4.466709

H 0 -8.967003 7.458667 -3.433620

H 0 -10.507171 7.367514 -2.548158

H 0 -6.776883 4.169448 -3.957042

H 0 -12.253680 0.339023 -4.759940

H 0 -8.885619 7.415390 7.130430

H 0 -4.078817 7.536263 4.608466

H 0 -0.188722 5.517333 6.446447

O 0 7.128825 0.837101 -1.769739

O 0 -6.743688 1.760234 1.433810

H 0 7.902657 0.458778 -1.315702

H 0 6.943985 1.641925 -1.260256

H 0 -7.689018 1.706468 1.203952

H 0 -6.444206 0.908168 1.852791

C -1 5.110977 1.406497 8.853557

C 0 3.742481 1.995942 8.493425

C 0 3.878310 3.421041 7.944704

C 0 3.003293 1.100550 7.494574

H 0 5.011605 0.399673 9.272912

H 0 5.746838 1.334334 7.962611

H 0 3.142882 2.044257 9.413648

H 0 4.450535 3.420829 7.008849

H 0 2.897452 3.859618 7.733149

H 0 4.395304 4.076322 8.653832

H 0 2.014245 1.506050 7.264012

H 0 3.559077 1.025535 6.553481

H 0 2.867125 0.086226 7.882967

C -1 -7.139954 -6.830985 9.563991

C 0 -6.903201 -5.890385 8.403260

C 0 -6.088095 -4.759272 8.555696

C 0 -7.521341 -6.100949 7.163531

C 0 -5.932512 -3.849342 7.507574

C 0 -7.358761 -5.198061 6.111504

C 0 -6.569781 -4.057767 6.282545

H 0 -6.288161 -6.826749 10.248978

H 0 -8.024878 -6.526054 10.136551

H 0 -5.575310 -4.604256 9.500268

H 0 -8.146700 -6.980038 7.024648

H 0 -5.313828 -2.967623 7.651664

H 0 -7.857210 -5.369517 5.162400

H 0 -6.474214 -3.337007 5.477339

H 0 -7.315000 -7.855068 9.218606

H 0 5.633808 2.028517 9.587890

H 0 7.700351 -0.501305 2.280868

C -1 6.647997 5.725948 0.042982

C 0 7.070731 4.574995 -0.839416

O 0 6.728733 3.412759 -0.577853

H 0 7.012817 5.537836 1.055922

N 0 7.840948 4.873385 -1.910443

C 0 8.356421 3.868868 -2.828534

C 0 9.738068 3.313455 -2.471591

O 0 10.568004 3.088358 -3.344774

H 0 7.670234 3.015047 -2.837164

H 0 8.158081 5.824452 -2.023884

N 0 9.948144 3.071467 -1.141955

C -1 11.057032 2.194000 -0.766003

C 0 10.602874 0.763356 -0.973686

O 0 9.597684 0.358312 -0.369230

H 0 11.932046 2.476218 -1.346169

H 0 9.114870 2.958559 -0.573903

N 0 11.272227 0.000959 -1.846686

C -1 10.756967 -1.337000 -2.120002

C 0 11.776974 -1.921431 -3.104320

C 0 12.277562 -0.681276 -3.860925

C 0 12.354470 0.395510 -2.769897

H 0 11.337298 -2.677949 -3.757970

H 0 12.603334 -2.388608 -2.557103

H 0 11.544916 -0.379885 -4.616971

H 0 13.237233 -0.832251 -4.360586

H 0 13.322267 0.367186 -2.253293

H 0 12.175285 1.398567 -3.158583

H 0 9.756331 -1.261531 -2.563341

H 0 5.556025 5.744061 0.090905

H 0 7.010057 6.697780 -0.299427

H 0 8.421405 4.285343 -3.833884

H 0 11.270266 2.338336 0.296022

H 0 10.658482 -1.905032 -1.192242

N 0 -3.337268 -0.219993 2.283796

C 0 -0.583248 0.550039 5.734373

C 0 -1.477422 1.600613 5.537537

C 0 -3.339715 1.182184 1.756835

C 0 -2.621839 1.240684 0.399647

C 0 -2.808290 1.355748 5.196940

C 0 -3.271685 0.040622 5.057536

C 0 -1.129146 0.996246 0.466620

C 0 -4.663263 -0.261338 4.527888

C 0 -0.240333 2.059330 0.260268

C 0 1.141393 1.881603 0.334676

C 0 -4.577564 -0.759222 3.050074

C 0 1.645263 0.623991 0.716949

C 0 -2.377834 -1.013438 5.311999

C 0 -1.042354 -0.763048 5.632224

C 0 0.779135 -0.449054 0.888224

C 0 -0.598062 -0.273650 0.737547

O 0 -5.685911 -0.527823 2.289603

O 0 2.993250 0.436794 0.897462

O 0 1.991505 2.920832 0.107462

H 0 -1.142129 2.623701 5.634714

H 0 -3.478460 2.193063 5.039507

H 0 3.388780 1.305997 1.069471

H 0 -1.244898 -1.141524 0.809968

H 0 -2.865124 1.821085 2.501367

H 0 -2.817580 2.227081 -0.026485

H 0 -4.325089 -1.827580 3.091234

H 0 -2.735240 -2.036604 5.266158

H 0 -0.610749 3.048936 0.016219

H 0 -0.367662 -1.593653 5.809851

H 0 1.192494 -1.415446 1.150402

H 0 -3.305649 -0.889664 1.482184

H 0 -3.108062 0.527978 -0.275157

H 0 -4.377664 1.484830 1.650351

H 0 -2.503540 -0.375569 2.855393

H 0 2.764211 2.636784 -0.509369

H 0 0.455565 0.748315 5.971710

H 0 -6.027418 2.047203 0.128634

H 0 -5.652171 -1.060379 0.962694

C 0 -5.680198 0.863966 4.713337

H 0 -6.677036 0.507779 4.448214

H 0 -5.694499 1.175625 5.761242

H 0 -5.450629 1.746298 4.117739

H 0 -5.040447 -1.138829 5.067235

**TS3R** (-8045.699842)

C -1 7.281996 0.461308 1.969331

C 0 5.979261 0.718534 2.734504

C 0 6.162428 0.506461 4.242008

C 0 5.432732 2.124997 2.451067

H 0 7.125637 0.517244 0.888966

H 0 8.050066 1.199340 2.231168

H 0 5.233548 -0.006096 2.381947

H 0 6.923545 1.188748 4.641466

H 0 5.230895 0.691729 4.785513

H 0 6.483206 -0.517184 4.463183

H 0 4.453779 2.276777 2.921106

H 0 6.109609 2.890432 2.852078

H 0 5.352531 2.324800 1.375819

C -1 2.868023 -2.655428 6.139957

C 0 3.156922 -2.409831 4.651719

C 0 2.571947 -3.531427 3.785285

C 0 2.635472 -1.045029 4.182035

H 0 1.790678 -2.642929 6.338405

H 0 3.248365 -3.630582 6.466431

H 0 4.248545 -2.415618 4.518721

H 0 1.482538 -3.576356 3.901560

H 0 2.783439 -3.362673 2.723902

H 0 2.974246 -4.512162 4.061330

H 0 1.539889 -1.008911 4.193557

H 0 2.994135 -0.233416 4.822233

H 0 2.955832 -0.825902 3.159656

C -1 0.465887 0.417451 10.771267

C 0 -0.284329 0.693061 9.461416

C 0 -1.708122 0.122720 9.494845

C 0 -0.307805 2.193666 9.148774

H 0 -0.036664 0.904717 11.616101

H 0 0.506530 -0.657245 10.984384

H 0 0.256012 0.186962 8.650090

H 0 -2.306062 0.614524 10.271791

H 0 -2.212889 0.276344 8.535442

H 0 -1.704931 -0.951513 9.705310

H 0 -0.828817 2.745891 9.939907

H 0 0.705490 2.601629 9.069158

H 0 -0.832297 2.388685 8.207836

C -1 -3.085938 -4.133021 12.675986

C 0 -3.256958 -5.438415 11.877503

O 0 -4.260884 -5.633170 11.197460

C 0 -2.774171 -2.978715 11.711573

H 0 -4.031619 -3.954070 13.194567

H 0 -1.831685 -3.151752 11.184374

H 0 -3.563374 -2.892461 10.960995

H 0 -2.700409 -2.028987 12.248760

N 0 -2.229275 -6.346904 11.940642

C -1 -2.114062 -7.479947 11.011993

C 0 -0.967353 -7.277004 10.000695

C 0 -0.976578 -5.910789 9.346969

C 0 -2.023512 -5.514471 8.504240

C 0 0.054706 -4.999274 9.603155

C 0 -2.030666 -4.245424 7.927015

C 0 0.047010 -3.722582 9.036249

C 0 -0.998890 -3.341193 8.195460

H 0 -3.077567 -7.536595 10.504085

H 0 -1.039899 -8.069696 9.245555

H 0 -0.006081 -7.422550 10.508280

H 0 -2.852134 -6.189006 8.309921

H 0 -1.393309 -6.061481 12.430047

H 0 0.874951 -5.294626 10.253135

H 0 -2.852122 -3.968831 7.276129

H 0 0.854574 -3.028105 9.249324

H 0 -1.015550 -2.346817 7.763100

C -1 0.152992 -6.401025 -0.188070

C 0 0.623681 -5.048611 -0.723323

C 0 0.295426 -4.899609 -2.213461

C 0 0.023486 -3.895619 0.087728

H 0 0.408719 -6.520513 0.873254

H 0 -0.937197 -6.493806 -0.278014

H 0 1.716923 -5.004040 -0.615479

H 0 -0.789406 -4.902387 -2.370619

H 0 0.690325 -3.960982 -2.614531

H 0 0.721492 -5.721585 -2.799251

H 0 -1.071659 -3.932109 0.091630

H 0 0.355304 -3.928043 1.131322

H 0 0.324451 -2.935106 -0.342325

C -1 -3.596924 -6.933979 3.872113

C 0 -3.529058 -6.002395 2.664124

S 0 -3.039205 -4.275359 3.043793

C 0 -1.343193 -4.509018 3.671895

H 0 -2.617460 -7.052198 4.344890

H 0 -4.295547 -6.550663 4.619569

H 0 -4.517169 -5.898852 2.208809

H 0 -2.844926 -6.384662 1.898456

H 0 -0.745173 -5.089918 2.964891

H 0 -0.904588 -3.513317 3.757636

H 0 -1.334651 -4.984005 4.655382

C -1 -8.592984 -6.747953 0.713186

C 0 -8.171653 -5.601060 1.593345

C 0 -6.949824 -4.949383 1.405797

C 0 -9.027236 -5.104840 2.586989

C 0 -6.603796 -3.820988 2.149294

C 0 -8.704839 -3.970405 3.324821

C 0 -7.503132 -3.295997 3.082535

O 0 -7.253888 -2.140891 3.765011

H 0 -9.060785 -7.552416 1.290520

H 0 -9.325876 -6.406361 -0.026919

H 0 -6.259372 -5.313796 0.648546

H 0 -9.979219 -5.598663 2.762069

H 0 -5.659157 -3.325983 1.969801

H 0 -9.381724 -3.572844 4.073255

H 0 -6.721707 -1.547734 3.186727

C -1 -3.717971 -4.902507 -2.923972

C 0 -3.554797 -3.942760 -1.748971

C 0 -4.745550 -2.994767 -1.606085

C 0 -4.620280 -2.053727 -0.423550

O 0 -3.479402 -1.786181 0.028590

O 0 -5.723284 -1.596244 0.046915

H 0 -3.800088 -4.358278 -3.872012

H 0 -2.641968 -3.351337 -1.859496

H 0 -3.433652 -4.506384 -0.815679

H 0 -5.687812 -3.542647 -1.498042

H 0 -4.855694 -2.379777 -2.510665

C -1 2.463002 -1.588778 -6.455244

C 0 2.709072 -1.371294 -4.975038

C 0 1.640277 -1.403797 -4.065253

C 0 3.994231 -1.146989 -4.469943

C 0 1.848557 -1.231052 -2.696358

C 0 4.216598 -0.985053 -3.098038

C 0 3.143957 -1.035693 -2.205831

H 0 2.016865 -2.572859 -6.637376

H 0 1.770899 -0.842134 -6.858197

H 0 0.632678 -1.566693 -4.436307

H 0 4.834942 -1.102933 -5.157668

H 0 1.001505 -1.239465 -2.014052

H 0 5.222033 -0.795213 -2.730487

H 0 3.309198 -0.910093 -1.141251

C -1 2.557263 4.064039 -4.609072

C 0 3.052092 3.838383 -3.177920

C 0 3.531176 2.407139 -2.948114

N 0 4.013007 2.225403 -1.564750

H 0 3.353320 3.877041 -5.338358

H 0 1.726481 3.390823 -4.844154

H 0 2.256531 4.059781 -2.456602

H 0 3.873314 4.535888 -2.956293

H 0 4.306345 2.142840 -3.681317

H 0 2.704431 1.707494 -3.101808

H 0 4.856763 2.774721 -1.401225

H 0 4.286426 1.254917 -1.429148

C -1 -2.421986 0.948941 -5.153625

C 0 -1.974755 1.012698 -3.685455

C 0 -2.043851 -0.370614 -3.027331

C 0 -0.573329 1.624696 -3.555354

H 0 -1.747279 0.308062 -5.733782

H 0 -2.671996 1.670153 -3.146022

H 0 -3.068884 -0.755409 -3.016159

H 0 -1.426181 -1.092730 -3.572235

H 0 -1.689979 -0.344213 -1.994339

H 0 0.170353 1.013871 -4.079291

H 0 -0.542254 2.633884 -3.982302

H 0 -0.266618 1.691703 -2.506753

C -1 -8.784212 -2.890408 -3.161027

C 0 -9.102343 -4.001299 -2.164908

S 0 -10.238908 -3.509762 -0.813925

C 0 -9.156028 -2.347169 0.085167

H 0 -9.694900 -2.520131 -3.637698

H 0 -8.296026 -2.044961 -2.668618

H 0 -8.188077 -4.392355 -1.703730

H 0 -9.599260 -4.839770 -2.663053

H 0 -8.168799 -2.783786 0.232133

H 0 -9.067513 -1.396863 -0.441174

H 0 -9.609359 -2.182416 1.063991

C -1 -11.413796 -0.217465 -4.324651

C 0 -10.761602 0.528910 -3.182669

C 0 -9.931215 1.631615 -3.419215

C 0 -10.977151 0.153952 -1.851229

C 0 -9.329847 2.334716 -2.376471

C 0 -10.386774 0.847291 -0.794870

C 0 -9.551089 1.934212 -1.053539

O 0 -8.976467 2.579967 0.016033

H 0 -10.693320 -0.445525 -5.117398

H 0 -11.845687 -1.161016 -3.980702

H 0 -9.745580 1.950653 -4.442057

H 0 -11.594993 -0.711789 -1.632723

H 0 -8.688110 3.184715 -2.581898

H 0 -10.556171 0.544076 0.232856

H 0 -8.280959 3.197159 -0.309532

C -1 -5.818452 4.555078 -3.596507

C 0 -5.059490 3.726999 -2.561872

C 0 -5.885902 3.361400 -1.351410

O 0 -6.902004 3.988305 -1.017747

O 0 -5.425145 2.329954 -0.687676

H 0 -6.189953 5.490050 -3.171142

H 0 -4.182037 4.273368 -2.190974

H 0 -4.671675 2.796718 -2.987164

C -1 -9.497194 6.950520 -2.623693

C 0 -8.768489 7.154168 -1.293041

C 0 -9.436577 6.404209 -0.138986

O 0 -7.390226 6.806643 -1.402803

H 0 -9.532845 5.885207 -2.881784

H 0 -8.771787 8.227213 -1.060216

H 0 -10.465121 6.746941 0.014548

H 0 -9.471920 5.326804 -0.336346

H 0 -8.875243 6.560672 0.787602

H 0 -7.304045 5.841511 -1.310748

N 0 -7.832039 5.227151 5.843466

C 0 -6.967762 4.201871 5.230198

C 0 -5.560626 4.681344 4.881836

O 0 -4.617328 3.892803 4.817313

C 0 -7.741773 3.807177 3.955452

C 0 -9.206892 4.022949 4.348352

C 0 -9.155440 5.299394 5.198455

H 0 -6.844773 3.356651 5.913382

H 0 -7.508010 2.797605 3.622588

H 0 -7.469169 4.484611 3.138583

H 0 -9.560822 3.186169 4.960104

H 0 -9.870559 4.118296 3.485707

H 0 -9.232378 6.196065 4.568276

H 0 -9.954965 5.344243 5.941355

N 0 -5.466393 5.985109 4.539101

C -1 -4.225182 6.582959 4.088983

C 0 -4.266004 7.049721 2.629817

C 0 -4.574872 5.966545 1.583564

C 0 -4.590900 6.596818 0.187438

C 0 -3.593601 4.789848 1.642514

H 0 -3.452211 5.827603 4.237835

H 0 -3.289994 7.500237 2.399997

H 0 -5.003495 7.858044 2.529189

H 0 -5.579207 5.570297 1.787810

H 0 -5.381172 7.343021 0.079366

H 0 -3.627073 7.070020 -0.031678

H 0 -6.277556 6.565040 4.694964

H 0 -4.773396 5.842302 -0.581221

H 0 -3.624640 4.278735 2.607873

H 0 -3.849952 4.059322 0.869660

H 0 -2.564139 5.124046 1.459596

C -1 -0.160108 5.153839 5.413367

C 0 1.067679 4.248660 5.500762

S 0 1.426505 3.299923 3.969323

C 0 1.962274 4.669915 2.889411

H 0 -0.037205 5.920101 4.642422

H 0 -1.059507 4.577628 5.170762

H 0 0.936940 3.494842 6.283533

H 0 1.961234 4.824469 5.766522

H 0 1.157094 5.393242 2.739656

H 0 2.835115 5.174797 3.314438

H 0 2.220688 4.228168 1.925220

C -1 -0.794037 9.087919 -2.939976

C 0 -0.452280 7.591147 -2.881707

C 0 -1.361448 6.867473 -1.871980

C 0 1.035794 7.387358 -2.569944

C 0 -1.187944 5.346766 -1.812140

H 0 -0.608917 9.565101 -1.969938

H 0 -0.650844 7.159113 -3.874333

H 0 -2.405420 7.097276 -2.118326

H 0 -1.186896 7.292791 -0.873224

H 0 1.664537 7.919713 -3.291951

H 0 1.277260 7.771890 -1.571160

H 0 1.319610 6.331835 -2.596939

H 0 -0.194429 5.056470 -1.457098

H 0 -1.918725 4.901603 -1.128655

H 0 -1.331929 4.889271 -2.797956

C -1 -8.525036 6.609689 7.776824

C 0 -7.511903 5.699069 7.095640

O 0 -6.446606 5.401341 7.623454

H 0 -9.380662 6.027955 8.137644

H 0 -8.034730 7.076011 8.630046

H 0 -1.847790 9.246198 -3.192394

H 0 -0.184834 9.606622 -3.687746

H 0 2.206158 5.091049 -4.749659

H 0 3.332067 -1.885069 6.763653

H 0 1.493061 0.792870 10.731627

H 0 -1.966025 -8.409054 11.572119

H 0 -2.298201 -4.231214 13.431968

H 0 -3.935251 -7.927062 3.556790

H 0 -7.741118 -7.172647 0.173658

H 0 -8.107762 -3.259413 -3.941463

H 0 -4.621614 -5.513159 -2.816761

H 0 -2.865188 -5.584446 -3.007038

H 0 0.604311 -7.233762 -0.736494

H 0 3.393834 -1.531644 -7.025117

H 0 -2.417577 1.942204 -5.616906

H 0 -3.433289 0.536962 -5.245969

H 0 -5.162461 4.794828 -4.437572

H 0 -8.973231 7.479911 -3.423997

H 0 -10.527334 7.319852 -2.573981

H 0 -6.675516 3.998002 -3.987290

H 0 -12.220443 0.369874 -4.779861

H 0 -8.909154 7.379609 7.101214

H 0 -3.979589 7.432703 4.738150

H 0 -0.327914 5.664048 6.367070

O 0 7.150148 0.847729 -1.781223

O 0 -6.735044 1.792646 1.434261

H 0 7.915895 0.457292 -1.323315

H 0 6.970488 1.650559 -1.266722

H 0 -7.672559 1.753787 1.164549

H 0 -6.454478 0.917625 1.794801

C -1 5.110899 1.406539 8.853492

C 0 3.713836 1.982106 8.596009

C 0 3.784903 3.464556 8.213950

C 0 2.977699 1.181754 7.517398

H 0 5.057790 0.355719 9.158658

H 0 5.723653 1.457386 7.945084

H 0 3.140255 1.901140 9.530693

H 0 4.320047 3.593956 7.265413

H 0 2.782756 3.890160 8.091423

H 0 4.308307 4.049813 8.977597

H 0 1.965964 1.564236 7.364963

H 0 3.503237 1.247881 6.557960

H 0 2.897285 0.123525 7.785668

C -1 -7.139956 -6.830979 9.563999

C 0 -6.722635 -6.035681 8.343982

C 0 -5.921698 -4.891299 8.480638

C 0 -7.147938 -6.396133 7.058435

C 0 -5.583957 -4.122352 7.365027

C 0 -6.801030 -5.634648 5.939669

C 0 -6.018531 -4.488212 6.089495

H 0 -6.348694 -6.821792 10.318537

H 0 -8.037791 -6.397912 10.022608

H 0 -5.557858 -4.617031 9.465976

H 0 -7.764943 -7.283007 6.932940

H 0 -4.982794 -3.226379 7.496406

H 0 -7.152439 -5.921193 4.953181

H 0 -5.764028 -3.886297 5.224571

H 0 -7.373207 -7.867230 9.303849

H 0 5.632840 1.960850 9.641085

H 0 7.689651 -0.530567 2.191194

C -1 6.648096 5.725862 0.042876

C 0 7.082687 4.575031 -0.833031

O 0 6.760129 3.409728 -0.559181

H 0 7.014935 5.547573 1.056693

N 0 7.838853 4.876477 -1.912867

C 0 8.359728 3.874054 -2.830686

C 0 9.740779 3.318233 -2.471108

O 0 10.573337 3.098026 -3.343164

H 0 7.675123 3.019139 -2.846214

H 0 8.141294 5.831186 -2.035266

N 0 9.946797 3.069881 -1.142322

C -1 11.056825 2.193892 -0.765947

C 0 10.604067 0.762528 -0.972377

O 0 9.601418 0.355256 -0.364789

H 0 11.931324 2.476549 -1.346341

H 0 9.112229 2.955135 -0.576254

N 0 11.271632 0.001455 -1.848030

C -1 10.756981 -1.336960 -2.119984

C 0 11.773712 -1.919651 -3.108663

C 0 12.270336 -0.678434 -3.866178

C 0 12.350471 0.397443 -2.774489

H 0 11.332260 -2.676045 -3.761261

H 0 12.602593 -2.386506 -2.564980

H 0 11.534538 -0.377049 -4.619163

H 0 13.228211 -0.828186 -4.369653

H 0 13.320071 0.368940 -2.261232

H 0 12.169700 1.400900 -3.161452

H 0 9.754485 -1.262488 -2.559476

H 0 5.555873 5.732161 0.090886

H 0 6.999169 6.699798 -0.305492

H 0 8.428457 4.294244 -3.834142

H 0 11.269733 2.338936 0.296035

H 0 10.662688 -1.905632 -1.192160

N 0 -3.303864 -0.236507 2.142727

C 0 -0.474659 0.185583 5.720585

C 0 -1.305699 1.299403 5.599476

C 0 -3.267643 1.206401 1.743671

C 0 -2.497466 1.371045 0.423334

C 0 -2.642393 1.155464 5.219940

C 0 -3.170492 -0.117613 4.963474

C 0 -1.012480 1.079870 0.502644

C 0 -4.572175 -0.311019 4.411522

C 0 -0.092918 2.125160 0.341192

C 0 1.282692 1.902663 0.399744

C 0 -4.489097 -0.792175 2.935696

C 0 1.751915 0.615872 0.721627

C 0 -2.339410 -1.238433 5.126183

C 0 -0.999584 -1.086980 5.484869

C 0 0.856249 -0.438194 0.854354

C 0 -0.515768 -0.215869 0.716119

O 0 -5.649433 -0.559849 2.225676

O 0 3.096050 0.385942 0.878453

O 0 2.171075 2.920384 0.207200

H 0 -0.912168 2.292616 5.780879

H 0 -3.267199 2.037640 5.124845

H 0 3.519491 1.245736 1.032650

H 0 -1.185229 -1.068486 0.741114

H 0 -2.813533 1.773835 2.556768

H 0 -2.645886 2.402314 0.097061

H 0 -4.242607 -1.860102 2.946697

H 0 -2.748085 -2.232423 4.962857

H 0 -0.435785 3.135253 0.142448

H 0 -0.366402 -1.962061 5.581985

H 0 1.239856 -1.427526 1.074473

H 0 -3.329787 -0.838836 1.252585

H 0 -2.977204 0.738576 -0.329362

H 0 -4.296894 1.534295 1.622752

H 0 -2.445458 -0.469524 2.647840

H 0 2.885856 2.643540 -0.478406

H 0 0.570476 0.302433 5.987488

H 0 -5.982736 2.127133 0.155530

H 0 -5.640166 -1.056045 1.156864

H 0 -5.025391 -1.163294 4.932328

C 0 -5.509035 0.880986 4.598763

H 0 -6.524565 0.598629 4.316051

H 0 -5.516600 1.180384 5.649341

H 0 -5.214950 1.756454 4.021628

**Int4R** (-8045.703233)

C -1 7.281530 0.460774 1.969179

C 0 5.756064 0.363379 1.922381

C 0 5.258221 -0.817534 2.764076

C 0 5.112933 1.683111 2.371378

H 0 7.649653 1.302595 1.376968

H 0 7.624311 0.604029 3.002049

H 0 5.485045 0.181069 0.871414

H 0 5.511845 -0.666364 3.820450

H 0 4.175222 -0.935898 2.682736

H 0 5.721728 -1.755866 2.440984

H 0 4.019492 1.643071 2.335695

H 0 5.396438 1.910890 3.406125

H 0 5.438598 2.512845 1.736340

C -1 2.867078 -2.655582 6.140410

C 0 2.197125 -1.671214 5.170943

C 0 1.397533 -2.397092 4.083052

C 0 1.298550 -0.683366 5.920784

H 0 2.115450 -3.267848 6.654231

H 0 3.545951 -3.332722 5.611302

H 0 2.990492 -1.097693 4.675535

H 0 0.555599 -2.944093 4.524826

H 0 0.987079 -1.690655 3.354563

H 0 2.019131 -3.113184 3.534870

H 0 0.481058 -1.208464 6.429319

H 0 1.856418 -0.126693 6.678048

H 0 0.846157 0.040905 5.236163

C -1 0.466073 0.417256 10.771099

C 0 -0.001051 0.229506 9.319506

C 0 -1.393545 -0.408463 9.251846

C 0 0.022789 1.557825 8.554120

H 0 -0.203522 1.098779 11.309314

H 0 0.475151 -0.535733 11.311420

H 0 0.708010 -0.452044 8.828035

H 0 -2.143264 0.234375 9.726831

H 0 -1.715910 -0.564167 8.217118

H 0 -1.414853 -1.378585 9.756487

H 0 -0.684393 2.271158 8.993737

H 0 1.016739 2.015750 8.571243

H 0 -0.262698 1.414106 7.506909

C -1 -3.085950 -4.133154 12.676025

C 0 -3.270937 -5.474132 11.949191

O 0 -4.352683 -5.772637 11.449040

C 0 -3.199309 -2.987578 11.660379

H 0 -3.879922 -4.060115 13.424867

H 0 -2.424630 -3.074141 10.892759

H 0 -4.170124 -3.023369 11.160553

H 0 -3.092127 -2.014914 12.148330

N 0 -2.172795 -6.286343 11.860350

C -1 -2.114307 -7.479783 11.011691

C 0 -0.974108 -7.390121 9.980583

C 0 -1.052763 -6.189659 9.062762

C 0 -1.883645 -6.198360 7.934945

C 0 -0.296063 -5.039779 9.321203

C 0 -1.940754 -5.099019 7.078328

C 0 -0.357388 -3.930933 8.474237

C 0 -1.177119 -3.961131 7.345415

H 0 -3.086773 -7.541789 10.518927

H 0 -0.986456 -8.314917 9.392053

H 0 -0.014302 -7.377767 10.512908

H 0 -2.493529 -7.072722 7.723641

H 0 -1.306389 -5.947331 12.251505

H 0 0.362264 -5.017903 10.186389

H 0 -2.581159 -5.125245 6.204272

H 0 0.240500 -3.050629 8.689321

H 0 -1.225132 -3.106025 6.678324

C -1 0.152968 -6.400885 -0.188182

C 0 0.725520 -5.054787 -0.648067

C 0 -0.407347 -4.076188 -0.981764

C 0 1.673551 -4.479852 0.411496

H 0 0.948160 -7.123290 0.027027

H 0 -0.438935 -6.263131 0.724100

H 0 1.307859 -5.224596 -1.565041

H 0 -1.026500 -3.880327 -0.098159

H 0 -0.022320 -3.117542 -1.341227

H 0 -1.058613 -4.484689 -1.760562

H 0 1.125753 -4.259038 1.335666

H 0 2.469510 -5.190988 0.659007

H 0 2.150761 -3.556574 0.068310

C -1 -3.593685 -6.933653 3.872204

C 0 -2.801342 -6.334484 2.712431

S 0 -1.694353 -4.939350 3.170000

C 0 -0.354849 -5.874760 3.985710

H 0 -2.929384 -7.333794 4.644236

H 0 -4.244453 -6.188688 4.335532

H 0 -3.473464 -5.921272 1.956324

H 0 -2.189749 -7.092641 2.211498

H 0 0.063295 -6.614359 3.297506

H 0 0.423832 -5.157827 4.248310

H 0 -0.700814 -6.359041 4.900568

C -1 -8.593112 -6.748077 0.712692

C 0 -7.935544 -5.619343 1.471754

C 0 -6.543503 -5.515799 1.579496

C 0 -8.710355 -4.621423 2.078078

C 0 -5.941820 -4.445803 2.237363

C 0 -8.124300 -3.546392 2.744374

C 0 -6.733744 -3.448651 2.802069

O 0 -6.079087 -2.415019 3.417964

H 0 -9.327369 -6.352989 0.003351

H 0 -7.855607 -7.334570 0.157655

H 0 -5.915130 -6.274514 1.120277

H 0 -9.791389 -4.672920 2.004312

H 0 -4.865495 -4.335531 2.286036

H 0 -8.743393 -2.778835 3.201200

H 0 -6.661747 -1.659751 3.578241

C -1 -3.718560 -4.901584 -2.924626

C 0 -3.988860 -3.580971 -2.201698

C 0 -4.647028 -3.783887 -0.827939

C 0 -4.912420 -2.447242 -0.149859

O 0 -3.944311 -1.907898 0.463268

O 0 -6.068264 -1.941957 -0.293275

H 0 -4.649789 -5.448723 -3.109880

H 0 -4.638798 -2.945943 -2.816776

H 0 -3.051903 -3.032615 -2.062043

H 0 -3.977694 -4.373186 -0.192349

H 0 -5.592318 -4.322446 -0.933737

C -1 2.463000 -1.589011 -6.455082

C 0 2.892260 -1.424657 -5.015670

C 0 1.965923 -1.566263 -3.973535

C 0 4.227228 -1.159048 -4.684499

C 0 2.363968 -1.468618 -2.640619

C 0 4.635828 -1.067996 -3.352042

C 0 3.704163 -1.235065 -2.325459

H 0 3.123455 -1.042896 -7.134974

H 0 2.484801 -2.643990 -6.754336

H 0 0.922661 -1.757896 -4.207928

H 0 4.955392 -1.026932 -5.481231

H 0 1.630427 -1.563483 -1.848080

H 0 5.669317 -0.842399 -3.106500

H 0 4.003966 -1.174379 -1.285416

C -1 2.557164 4.064007 -4.608863

C 0 2.955868 3.698254 -3.173035

C 0 3.611947 2.320907 -3.076938

N 0 4.059480 2.030858 -1.699051

H 0 3.430761 4.081104 -5.269615

H 0 1.846974 3.335538 -5.013568

H 0 2.080090 3.719729 -2.513314

H 0 3.652243 4.455221 -2.783257

H 0 4.447985 2.256866 -3.789223

H 0 2.898502 1.541367 -3.360977

H 0 4.774871 2.698318 -1.410318

H 0 4.522161 1.122745 -1.689150

C -1 -2.421990 0.948894 -5.153499

C 0 -1.835394 0.867361 -3.736577

C 0 -1.899644 -0.570285 -3.209770

C 0 -0.404007 1.418293 -3.690005

H 0 -1.831418 0.340819 -5.849601

H 0 -2.455341 1.487705 -3.073292

H 0 -2.934817 -0.919626 -3.150664

H 0 -1.356516 -1.253453 -3.874602

H 0 -1.458531 -0.651159 -2.212928

H 0 0.261869 0.834162 -4.336288

H 0 -0.369529 2.461328 -4.026359

H 0 0.001125 1.376532 -2.674324

C -1 -8.784248 -2.890484 -3.160912

C 0 -9.007512 -3.961338 -2.097137

S 0 -10.343220 -3.556560 -0.908223

C 0 -9.587247 -2.099863 -0.106273

H 0 -9.706993 -2.678524 -3.707159

H 0 -8.433662 -1.963476 -2.705485

H 0 -8.090727 -4.135332 -1.524060

H 0 -9.308917 -4.912143 -2.547916

H 0 -8.553742 -2.318251 0.169890

H 0 -9.627310 -1.232387 -0.761704

H 0 -10.168315 -1.889227 0.793917

C -1 -11.413384 -0.217118 -4.324342

C 0 -10.647890 0.379103 -3.169128

C 0 -9.253600 0.471078 -3.201022

C 0 -11.302367 0.811646 -2.007816

C 0 -8.527170 0.946765 -2.111694

C 0 -10.594428 1.320689 -0.921288

C 0 -9.201155 1.387424 -0.969227

O 0 -8.525979 1.874064 0.131363

H 0 -11.723517 -1.243973 -4.096239

H 0 -12.320060 0.354676 -4.546220

H 0 -8.718795 0.138462 -4.085722

H 0 -12.386020 0.751157 -1.952742

H 0 -7.445625 0.953439 -2.147235

H 0 -11.104656 1.669055 -0.029810

H 0 -7.784513 2.443157 -0.187336

C -1 -5.818631 4.554536 -3.596910

C 0 -4.931570 3.611532 -2.767884

C 0 -5.643808 2.861112 -1.662635

O 0 -6.554011 3.353758 -0.982121

O 0 -5.190148 1.644977 -1.460553

H 0 -6.331375 5.283000 -2.968417

H 0 -4.141591 4.188292 -2.267503

H 0 -4.422597 2.876139 -3.396510

C -1 -9.497364 6.950069 -2.623557

C 0 -9.172539 5.658686 -1.896029

C 0 -9.320339 4.454605 -2.825189

O 0 -7.850491 5.820328 -1.389383

H 0 -8.821409 7.074981 -3.475721

H 0 -9.873690 5.530997 -1.052629

H 0 -10.372278 4.302255 -3.087249

H 0 -8.757577 4.609115 -3.750495

H 0 -8.964513 3.536532 -2.358453

H 0 -7.486680 4.952089 -1.147916

N 0 -7.460681 4.949818 6.256137

C 0 -6.369557 4.006346 5.926767

C 0 -5.155396 4.615633 5.232018

O 0 -4.124302 3.949043 5.097223

C 0 -7.037832 2.961240 5.006156

C 0 -8.511201 3.008047 5.424028

C 0 -8.747126 4.498718 5.695694

H 0 -5.997020 3.544631 6.845103

H 0 -6.571151 1.981693 5.104282

H 0 -6.932584 3.266885 3.959416

H 0 -8.660058 2.437779 6.347449

H 0 -9.186467 2.609804 4.662746

H 0 -8.971037 5.035670 4.764167

H 0 -9.565474 4.681091 6.394238

N 0 -5.311397 5.852953 4.720462

C -1 -4.224663 6.582620 4.088736

C 0 -4.510263 6.969207 2.634056

C 0 -4.713499 5.798722 1.658936

C 0 -4.988557 6.330851 0.248213

C 0 -3.517099 4.840625 1.657753

H 0 -3.343128 5.944968 4.159599

H 0 -3.665425 7.579173 2.284637

H 0 -5.392271 7.623424 2.599255

H 0 -5.597287 5.233299 1.986283

H 0 -5.887749 6.952055 0.208084

H 0 -4.140944 6.926586 -0.113580

H 0 -6.170280 6.334049 4.944117

H 0 -5.145987 5.506291 -0.450319

H 0 -3.406340 4.325154 2.614684

H 0 -3.645623 4.080910 0.880650

H 0 -2.580018 5.370332 1.448109

C -1 -0.160256 5.153772 5.413229

C 0 -0.771472 3.813505 5.011033

S 0 -0.381187 3.302391 3.291912

C 0 1.381696 2.855687 3.464280

H 0 0.933258 5.133672 5.373304

H 0 -0.507585 5.951500 4.750507

H 0 -1.862479 3.863153 5.050704

H 0 -0.452122 3.014276 5.688880

H 0 1.928876 3.644295 3.986247

H 0 1.498473 1.912389 4.004752

H 0 1.792882 2.757173 2.459230

C -1 -0.794238 9.087977 -2.940206

C 0 -0.544192 7.572233 -2.882570

C 0 -1.482884 6.898894 -1.863409

C 0 0.932860 7.280786 -2.585843

C 0 -1.392121 5.369807 -1.814051

H 0 -0.568231 9.554520 -1.973858

H 0 -0.777668 7.153310 -3.873243

H 0 -2.516153 7.186557 -2.097322

H 0 -1.273038 7.307401 -0.864681

H 0 1.584407 7.782454 -3.309642

H 0 1.204749 7.642215 -1.586384

H 0 1.155963 6.210937 -2.624659

H 0 -0.408347 5.026390 -1.479615

H 0 -2.130076 4.962509 -1.115783

H 0 -1.578849 4.927029 -2.799622

C -1 -8.525068 6.609706 7.776866

C 0 -7.304028 5.808207 7.323128

O 0 -6.213166 5.933642 7.868830

H 0 -9.227967 5.966623 8.317636

H 0 -8.174186 7.389948 8.450870

H 0 -1.839307 9.310123 -3.180506

H 0 -0.163535 9.565712 -3.696953

H 0 2.085082 5.049962 -4.647374

H 0 3.445996 -2.125559 6.904616

H 0 1.476283 0.839126 10.811781

H 0 -1.985670 -8.376888 11.629003

H 0 -2.124366 -4.093051 13.201282

H 0 -4.223520 -7.755965 3.514767

H 0 -9.124124 -7.432284 1.385257

H 0 -8.020867 -3.219234 -3.875945

H 0 -3.068113 -5.549942 -2.327040

H 0 -3.229245 -4.735580 -3.889957

H 0 -0.502382 -6.837633 -0.950089

H 0 1.441260 -1.229190 -6.610234

H 0 -2.422658 1.978078 -5.530283

H 0 -3.452248 0.577545 -5.179038

H 0 -5.198982 5.084213 -4.325666

H 0 -9.368110 7.809310 -1.958986

H 0 -10.527820 6.948667 -2.993687

H 0 -6.574283 3.985999 -4.145562

H 0 -10.802956 -0.249844 -5.231015

H 0 -9.062235 7.057176 6.935597

H 0 -4.021564 7.487821 4.674303

H 0 -0.450098 5.407220 6.439227

O 0 7.029603 0.766320 -1.531364

O 0 -6.206964 0.378214 0.547719

H 0 7.822421 0.369554 -1.129101

H 0 6.898763 1.575426 -1.011164

H 0 -5.616956 1.184631 -0.638151

H 0 -7.142114 0.664801 0.572496

C -1 5.110756 1.406368 8.853636

C 0 4.093473 1.477395 7.707435

C 0 3.580043 2.907277 7.502326

C 0 4.682811 0.921783 6.405691

H 0 5.457156 0.379982 9.013800

H 0 5.989421 2.024283 8.631857

H 0 3.235011 0.850290 7.986188

H 0 4.398250 3.573124 7.201899

H 0 2.815130 2.944575 6.719076

H 0 3.141670 3.312821 8.420411

H 0 3.956779 0.964459 5.588199

H 0 5.560772 1.503655 6.099404

H 0 4.997703 -0.120841 6.518769

C -1 -7.139749 -6.830867 9.564121

C 0 -6.660818 -6.071053 8.346668

C 0 -5.592126 -5.170841 8.448292

C 0 -7.283377 -6.227049 7.099328

C 0 -5.171173 -4.437828 7.337738

C 0 -6.862512 -5.498372 5.985838

C 0 -5.803763 -4.594202 6.103221

H 0 -6.368924 -6.852374 10.337767

H 0 -8.029608 -6.355825 9.995817

H 0 -5.088131 -5.057613 9.402712

H 0 -8.109269 -6.928116 7.001559

H 0 -4.344729 -3.743316 7.439611

H 0 -7.355788 -5.628462 5.026808

H 0 -5.481727 -4.018941 5.242451

H 0 -7.416856 -7.859300 9.309007

H 0 4.677614 1.767347 9.792634

H 0 7.758244 -0.442939 1.579779

C -1 6.647838 5.725848 0.042988

C 0 7.048857 4.516804 -0.767475

O 0 6.768885 3.371398 -0.384649

H 0 7.102317 5.645811 1.033851

N 0 7.729634 4.749627 -1.913060

C 0 8.227589 3.693299 -2.781217

C 0 9.643411 3.205161 -2.461200

O 0 10.436402 2.947698 -3.359365

H 0 7.570981 2.820459 -2.690284

H 0 7.999758 5.698603 -2.123717

N 0 9.925321 3.049279 -1.131189

C -1 11.057094 2.194067 -0.766068

C 0 10.617151 0.755568 -0.960702

O 0 9.623562 0.343182 -0.342461

H 0 11.919842 2.483910 -1.360798

H 0 9.119438 2.956152 -0.521461

N 0 11.280043 -0.003028 -1.842237

C -1 10.756992 -1.337019 -2.120028

C 0 11.755527 -1.913806 -3.130334

C 0 12.247521 -0.666842 -3.881061

C 0 12.348349 0.396524 -2.779098

H 0 11.299957 -2.660382 -3.784528

H 0 12.589747 -2.391386 -2.604461

H 0 11.502223 -0.352448 -4.619212

H 0 13.197183 -0.815669 -4.400133

H 0 13.324374 0.358463 -2.279000

H 0 12.165267 1.403073 -3.155582

H 0 9.747735 -1.253626 -2.542003

H 0 5.563767 5.707600 0.181701

H 0 6.937160 6.675454 -0.412389

H 0 8.225597 4.035694 -3.816119

H 0 11.282508 2.351005 0.291241

H 0 10.675309 -1.915833 -1.197246

H 0 -6.173389 -0.635661 0.204788

N 0 -3.383154 0.029760 2.089698

C 0 -5.407902 -0.006488 8.395887

C 0 -4.708799 1.166779 8.108331

C 0 -3.252028 1.389070 1.486192

C 0 -2.566468 1.282552 0.117527

C 0 -4.122869 1.343806 6.855781

C 0 -4.220385 0.353628 5.867352

C 0 -1.131179 0.827752 0.211996

C 0 -3.486428 0.582307 4.550792

C 0 -0.087079 1.759112 0.138329

C 0 1.241793 1.361596 0.261769

C 0 -4.185748 -0.093069 3.367478

C 0 1.529410 0.008086 0.520725

C 0 -4.914079 -0.824648 6.170109

C 0 -5.511027 -0.998140 7.421414

C 0 0.504671 -0.929195 0.584579

C 0 -0.820324 -0.525325 0.409091

O 0 -5.453980 0.455651 3.175552

O 0 2.822860 -0.389322 0.702663

O 0 2.263945 2.268257 0.245131

H 0 -4.621785 1.950826 8.855426

H 0 -3.613117 2.270965 6.622083

H 0 3.361702 0.417469 0.738981

H 0 -1.608633 -1.273937 0.395346

H 0 -2.671374 2.011454 2.167540

H 0 -2.616370 2.269552 -0.350194

H 0 -4.235393 -1.177151 3.509953

H 0 -5.010290 -1.616558 5.436077

H 0 -0.295162 2.813490 -0.015083

H 0 -6.047678 -1.917306 7.629550

H 0 0.751308 -1.966238 0.768070

H 0 -3.792208 -0.672904 1.372679

H 0 -3.141740 0.604310 -0.517097

H 0 -4.258625 1.791899 1.397623

H 0 -2.441775 -0.334366 2.262736

H 0 2.902269 2.130572 -0.546979

H 0 -5.868685 -0.147612 9.369060

H 0 -5.758511 0.308868 2.245139

H 0 -3.491178 1.661648 4.367378

C 0 -2.028920 0.098611 4.685006

H 0 -1.390333 0.460213 3.872122

H 0 -1.601450 0.484284 5.612149

H 0 -1.976214 -0.995852 4.730111

**Int5R** (-8045.695427)

C -1 7.281810 0.461276 1.969280

C 0 5.962814 0.725167 2.707296

C 0 6.116302 0.521588 4.219087

C 0 5.423305 2.130068 2.404939

H 0 7.147083 0.513521 0.886196

H 0 8.045163 1.199150 2.244383

H 0 5.222608 0.000075 2.343986

H 0 6.869881 1.205861 4.629241

H 0 5.173867 0.711566 4.742656

H 0 6.431037 -0.500779 4.454486

H 0 4.436941 2.289021 2.857332

H 0 6.095024 2.898050 2.810013

H 0 5.359481 2.317813 1.327475

C -1 2.868028 -2.655444 6.139988

C 0 3.177754 -2.516591 4.641650

C 0 2.584138 -3.686670 3.846951

C 0 2.685304 -1.179240 4.075712

H 0 1.787471 -2.619896 6.325930

H 0 3.235416 -3.607876 6.537040

H 0 4.270805 -2.548972 4.525342

H 0 1.492467 -3.706682 3.951736

H 0 2.812952 -3.599329 2.779187

H 0 2.967460 -4.650866 4.198068

H 0 1.591731 -1.133667 4.065725

H 0 3.043867 -0.333700 4.670762

H 0 3.025407 -1.024747 3.047860

C -1 0.465990 0.417413 10.771221

C 0 -0.219842 0.668566 9.420677

C 0 -1.629996 0.067403 9.387055

C 0 -0.261020 2.166508 9.096288

H 0 -0.089078 0.901415 11.583720

H 0 0.517750 -0.653936 10.996222

H 0 0.372824 0.170844 8.640514

H 0 -2.276149 0.552223 10.129112

H 0 -2.089932 0.202418 8.402697

H 0 -1.613719 -1.004225 9.607922

H 0 -0.846774 2.708243 9.848683

H 0 0.743270 2.603381 9.080503

H 0 -0.728495 2.345369 8.123404

C -1 -3.085949 -4.133029 12.675978

C 0 -3.258117 -5.438579 11.880037

O 0 -4.268892 -5.639361 11.212169

C 0 -2.798889 -2.975601 11.707141

H 0 -4.024507 -3.961731 13.209783

H 0 -1.864434 -3.142085 11.163661

H 0 -3.601345 -2.894734 10.970134

H 0 -2.722399 -2.025502 12.243276

N 0 -2.223590 -6.340160 11.932099

C -1 -2.114078 -7.479889 11.011959

C 0 -0.976736 -7.289623 9.990664

C 0 -1.013552 -5.944520 9.298500

C 0 -2.062168 -5.600786 8.434556

C 0 -0.010204 -4.998821 9.541122

C 0 -2.097507 -4.347834 7.824618

C 0 -0.047311 -3.738785 8.940819

C 0 -1.094892 -3.409994 8.080976

H 0 -3.082135 -7.540734 10.511420

H 0 -1.041223 -8.105058 9.259372

H 0 -0.010966 -7.406013 10.497461

H 0 -2.869232 -6.303758 8.250127

H 0 -1.384476 -6.050502 12.413435

H 0 0.810502 -5.254218 10.207305

H 0 -2.920755 -4.102322 7.163348

H 0 0.737591 -3.015718 9.143510

H 0 -1.138040 -2.430194 7.621655

C -1 0.152933 -6.400937 -0.188023

C 0 0.620603 -5.045072 -0.731700

C 0 0.279141 -4.895208 -2.218801

C 0 0.029926 -3.889972 0.085188

H 0 0.419960 -6.520717 0.868138

H 0 -0.938021 -6.491311 -0.267837

H 0 1.714770 -5.000692 -0.634969

H 0 -0.806366 -4.894984 -2.368029

H 0 0.674096 -3.957555 -2.622181

H 0 0.696631 -5.718099 -2.809387

H 0 -1.065003 -3.935441 0.109908

H 0 0.384906 -3.914245 1.121304

H 0 0.311794 -2.923990 -0.341770

C -1 -3.596863 -6.933972 3.872074

C 0 -3.553783 -6.084944 2.605844

S 0 -3.075948 -4.330247 2.856951

C 0 -1.384983 -4.512150 3.519358

H 0 -2.611237 -7.008930 4.341025

H 0 -4.292070 -6.511321 4.602404

H 0 -4.548767 -6.023184 2.157666

H 0 -2.875417 -6.513304 1.859664

H 0 -0.792482 -5.175440 2.884894

H 0 -0.933725 -3.518680 3.499405

H 0 -1.390144 -4.878338 4.548467

C -1 -8.592976 -6.747972 0.713188

C 0 -8.210264 -5.612627 1.624657

C 0 -6.997512 -4.935045 1.475604

C 0 -9.091934 -5.156410 2.614952

C 0 -6.682320 -3.823862 2.257834

C 0 -8.800037 -4.040450 3.391859

C 0 -7.602466 -3.343723 3.195164

O 0 -7.376791 -2.218357 3.932144

H 0 -9.323532 -6.407328 -0.029938

H 0 -7.723962 -7.141087 0.176806

H 0 -6.289239 -5.265820 0.719075

H 0 -10.038486 -5.670156 2.759392

H 0 -5.737640 -3.316813 2.115761

H 0 -9.494408 -3.677533 4.141810

H 0 -6.824534 -1.607731 3.399388

C -1 -3.718020 -4.902512 -2.924005

C 0 -3.654993 -3.994401 -1.701710

C 0 -4.797381 -2.975371 -1.686574

C 0 -4.771386 -2.068965 -0.481129

O 0 -3.576704 -1.783829 -0.040294

O 0 -5.823656 -1.650076 0.025854

H 0 -3.631795 -4.323559 -3.850473

H 0 -2.703143 -3.457729 -1.674659

H 0 -3.692041 -4.591270 -0.781800

H 0 -5.778491 -3.458872 -1.712576

H 0 -4.739906 -2.336509 -2.578517

C -1 2.463022 -1.588775 -6.455159

C 0 2.698775 -1.363138 -4.977198

C 0 1.630145 -1.397504 -4.070679

C 0 3.984928 -1.136666 -4.470611

C 0 1.837623 -1.223578 -2.701412

C 0 4.205276 -0.974775 -3.100162

C 0 3.130144 -1.025779 -2.209183

H 0 3.252275 -1.132492 -7.059419

H 0 2.445054 -2.659444 -6.692386

H 0 0.622990 -1.558205 -4.445197

H 0 4.826016 -1.087360 -5.157792

H 0 0.992849 -1.224818 -2.019874

H 0 5.209385 -0.779797 -2.731376

H 0 3.291455 -0.893969 -1.144044

C -1 2.557195 4.063997 -4.609076

C 0 3.039372 3.843964 -3.172806

C 0 3.527271 2.416254 -2.937269

N 0 3.977148 2.233366 -1.543177

H 0 3.361226 3.883090 -5.330981

H 0 1.734251 3.383427 -4.850697

H 0 2.232913 4.058086 -2.462235

H 0 3.852455 4.548250 -2.942622

H 0 4.321385 2.161748 -3.653626

H 0 2.710122 1.710452 -3.112746

H 0 4.815398 2.784463 -1.359404

H 0 4.247053 1.262027 -1.401534

C -1 -2.422031 0.948889 -5.153631

C 0 -1.964611 1.016339 -3.687809

C 0 -2.022418 -0.366858 -3.027135

C 0 -0.563738 1.631359 -3.570574

H 0 -1.749576 0.308064 -5.736924

H 0 -2.661235 1.670851 -3.143957

H 0 -3.043839 -0.761884 -3.026601

H 0 -1.392720 -1.084090 -3.566351

H 0 -1.676099 -0.333329 -1.990900

H 0 0.176546 1.020860 -4.099384

H 0 -0.539123 2.638850 -4.000988

H 0 -0.249087 1.702824 -2.524911

C -1 -8.783919 -2.890665 -3.160825

C 0 -9.118730 -4.024313 -2.195672

S 0 -10.284478 -3.569037 -0.856474

C 0 -9.224405 -2.429437 0.097340

H 0 -9.685933 -2.511431 -3.646542

H 0 -8.307098 -2.055324 -2.640547

H 0 -8.212780 -4.423860 -1.725044

H 0 -9.602475 -4.852712 -2.722655

H 0 -8.245274 -2.875936 0.266400

H 0 -9.112887 -1.470202 -0.408319

H 0 -9.707191 -2.280663 1.064600

C -1 -11.414081 -0.217213 -4.324900

C 0 -10.783978 0.533200 -3.174940

C 0 -10.003471 1.673463 -3.401504

C 0 -10.968394 0.124904 -1.848921

C 0 -9.419912 2.381671 -2.353417

C 0 -10.392746 0.822369 -0.786642

C 0 -9.606394 1.947151 -1.035683

O 0 -9.043672 2.601384 0.035818

H 0 -10.682308 -0.434728 -5.110539

H 0 -11.838667 -1.166921 -3.988874

H 0 -9.845121 2.018677 -4.420407

H 0 -11.548674 -0.768872 -1.640014

H 0 -8.815503 3.259878 -2.551001

H 0 -10.537462 0.494277 0.237253

H 0 -8.329790 3.195490 -0.294774

C -1 -5.818475 4.555062 -3.596506

C 0 -5.154038 3.546617 -2.664489

C 0 -5.969572 3.214762 -1.437870

O 0 -6.944295 3.886306 -1.073126

O 0 -5.545662 2.152783 -0.795340

H 0 -6.025968 5.501044 -3.092995

H 0 -4.186208 3.921001 -2.308601

H 0 -4.934140 2.601721 -3.172471

C -1 -9.497207 6.950491 -2.623701

C 0 -8.727547 7.111418 -1.305775

C 0 -9.419213 6.401930 -0.139693

O 0 -7.371425 6.686951 -1.430023

H 0 -9.606271 5.889131 -2.878031

H 0 -8.665973 8.183070 -1.075395

H 0 -10.425448 6.800086 0.028003

H 0 -9.516016 5.327341 -0.332772

H 0 -8.836240 6.529498 0.777725

H 0 -7.343035 5.715200 -1.387324

N 0 -7.790750 5.182888 5.895885

C 0 -6.899256 4.164655 5.311153

C 0 -5.495790 4.670621 4.974335

O 0 -4.523522 3.917478 4.961276

C 0 -7.648873 3.743921 4.029889

C 0 -9.123584 3.939774 4.395665

C 0 -9.107317 5.223855 5.235035

H 0 -6.770862 3.330695 6.006858

H 0 -7.398297 2.734135 3.712988

H 0 -7.372236 4.414810 3.209135

H 0 -9.474761 3.102632 5.008458

H 0 -9.773447 4.016936 3.520659

H 0 -9.195812 6.113958 4.596955

H 0 -9.916672 5.257982 5.968013

N 0 -5.441663 5.961419 4.578092

C -1 -4.225201 6.582962 4.088969

C 0 -4.153504 6.638561 2.559151

C 0 -3.913927 5.285991 1.869852

C 0 -4.071713 5.439311 0.353524

C 0 -2.534446 4.720839 2.229724

H 0 -3.387387 6.020108 4.503893

H 0 -3.342848 7.324603 2.274927

H 0 -5.082304 7.085918 2.180894

H 0 -4.674938 4.576217 2.220760

H 0 -5.071594 5.788926 0.076270

H 0 -3.344475 6.157065 -0.043236

H 0 -6.280673 6.512980 4.675110

H 0 -3.892613 4.480721 -0.142405

H 0 -2.468687 4.463154 3.288242

H 0 -2.313449 3.813770 1.664912

H 0 -1.744851 5.446333 1.996270

C -1 -0.159921 5.153820 5.413270

C 0 1.084398 4.278310 5.279871

S 0 1.250698 3.428384 3.661710

C 0 1.505307 4.879005 2.584375

H 0 -0.165702 5.956046 4.670346

H 0 -1.073194 4.567099 5.278929

H 0 1.071889 3.475073 6.022759

H 0 1.994468 4.860914 5.460963

H 0 0.601242 5.488220 2.516304

H 0 2.334388 5.489884 2.954869

H 0 1.752752 4.485390 1.597537

C -1 -0.794041 9.087957 -2.940014

C 0 -0.312111 7.629438 -2.888563

C 0 -1.215482 6.785666 -1.970347

C 0 1.161778 7.558006 -2.468735

C 0 -0.883051 5.290483 -1.938254

H 0 -0.726900 9.552916 -1.949045

H 0 -0.393038 7.210771 -3.903339

H 0 -2.256419 6.914446 -2.294020

H 0 -1.164081 7.193741 -0.950701

H 0 1.787237 8.173277 -3.124614

H 0 1.289429 7.927827 -1.443699

H 0 1.547396 6.535051 -2.503515

H 0 0.080823 5.087179 -1.461964

H 0 -1.641498 4.736243 -1.377003

H 0 -0.848266 4.869833 -2.949746

C -1 -8.525034 6.609678 7.776808

C 0 -7.490084 5.695422 7.136138

O 0 -6.426514 5.430181 7.683835

H 0 -9.377631 6.026954 8.142889

H 0 -8.053382 7.107973 8.622810

H 0 -1.837172 9.150986 -3.266873

H 0 -0.186047 9.683441 -3.629134

H 0 2.198378 5.087597 -4.753949

H 0 3.331137 -1.846988 6.714375

H 0 1.487081 0.814529 10.780407

H 0 -1.962999 -8.404531 11.579505

H 0 -2.285315 -4.224466 13.419523

H 0 -3.926255 -7.949973 3.627877

H 0 -9.049649 -7.575770 1.265878

H 0 -8.092387 -3.241204 -3.936433

H 0 -4.665199 -5.452345 -2.965520

H 0 -2.908095 -5.637981 -2.916273

H 0 0.598174 -7.233272 -0.743414

H 0 1.503798 -1.169200 -6.773422

H 0 -2.422017 1.940909 -5.618227

H 0 -3.432931 0.534856 -5.238872

H 0 -5.168701 4.754301 -4.453102

H 0 -8.959179 7.444839 -3.436997

H 0 -10.500326 7.384066 -2.550641

H 0 -6.767222 4.166520 -3.979230

H 0 -12.222086 0.361783 -4.788038

H 0 -8.910381 7.354576 7.074118

H 0 -4.174027 7.597575 4.499336

H 0 -0.194867 5.617100 6.405744

O 0 7.154985 0.851289 -1.798300

O 0 -6.809105 1.780327 1.414552

H 0 7.917914 0.467205 -1.331017

H 0 6.961021 1.652927 -1.287231

H 0 -7.756706 1.778044 1.177583

H 0 -6.574595 0.932512 1.847877

C -1 5.110867 1.406531 8.853624

C 0 3.768693 2.042940 8.470909

C 0 3.965000 3.457692 7.913725

C 0 3.009517 1.166172 7.469401

H 0 4.969964 0.406735 9.277935

H 0 5.755208 1.305882 7.971722

H 0 3.159036 2.119795 9.382727

H 0 4.541760 3.428554 6.981296

H 0 3.004253 3.934293 7.693712

H 0 4.503912 4.095666 8.622371

H 0 2.040243 1.603999 7.212623

H 0 3.580809 1.056293 6.540550

H 0 2.826260 0.164827 7.871933

C -1 -7.139973 -6.830993 9.564010

C 0 -6.711555 -6.030031 8.353645

C 0 -5.937165 -4.870968 8.509570

C 0 -7.099114 -6.399159 7.058723

C 0 -5.589539 -4.094124 7.403576

C 0 -6.740031 -5.631117 5.948544

C 0 -5.984835 -4.469214 6.117945

H 0 -6.357219 -6.823800 10.327434

H 0 -8.044084 -6.403392 10.014967

H 0 -5.599120 -4.593022 9.502727

H 0 -7.695287 -7.298048 6.919062

H 0 -5.008058 -3.187824 7.548241

H 0 -7.061579 -5.924603 4.953937

H 0 -5.723439 -3.861026 5.259723

H 0 -7.367465 -7.867622 9.296643

H 0 5.645155 2.014838 9.591110

H 0 7.681885 -0.530429 2.203932

C -1 6.648091 5.725934 0.042906

C 0 7.078327 4.578688 -0.840547

O 0 6.740658 3.414006 -0.582801

H 0 6.991832 5.526698 1.060940

N 0 7.848312 4.883074 -1.909890

C 0 8.367608 3.884395 -2.832336

C 0 9.746165 3.323384 -2.471952

O 0 10.580286 3.103365 -3.342563

H 0 7.680244 3.031873 -2.852974

H 0 8.161964 5.835707 -2.019587

N 0 9.948560 3.071180 -1.143272

C -1 11.056872 2.193906 -0.765959

C 0 10.603955 0.762964 -0.973460

O 0 9.600885 0.355871 -0.367070

H 0 11.932375 2.476360 -1.345300

H 0 9.112625 2.955754 -0.579542

N 0 11.272009 0.001488 -1.848377

C -1 10.756978 -1.336951 -2.119982

C 0 11.773816 -1.920033 -3.108457

C 0 12.270961 -0.679060 -3.866069

C 0 12.351255 0.396943 -2.774499

H 0 11.332240 -2.676375 -3.761047

H 0 12.602506 -2.387074 -2.564627

H 0 11.535356 -0.377558 -4.619194

H 0 13.228877 -0.829173 -4.369375

H 0 13.320744 0.368102 -2.261015

H 0 12.171085 1.400464 -3.161626

H 0 9.754585 -1.262346 -2.559418

H 0 5.555424 5.752365 0.070313

H 0 7.023631 6.697532 -0.285327

H 0 8.439693 4.309174 -3.833689

H 0 11.268888 2.338209 0.296332

H 0 10.662690 -1.905356 -1.192009

N 0 -3.458592 -0.325326 2.070453

C 0 -0.399302 0.100536 5.613382

C 0 -1.208756 1.230882 5.519148

C 0 -3.390225 1.115943 1.727537

C 0 -2.613421 1.320528 0.412684

C 0 -2.557358 1.116685 5.175796

C 0 -3.118098 -0.142587 4.927367

C 0 -1.123633 1.051206 0.489896

C 0 -4.547060 -0.304500 4.438058

C 0 -0.219538 2.100608 0.275918

C 0 1.160751 1.906199 0.338290

C 0 -4.519876 -0.821560 2.972274

C 0 1.654119 0.643013 0.711602

C 0 -2.305144 -1.280077 5.054500

C 0 -0.953911 -1.159243 5.379131

C 0 0.775405 -0.418616 0.889258

C 0 -0.600763 -0.224948 0.752836

O 0 -5.788713 -0.605594 2.373858

O 0 3.003016 0.438407 0.875799

O 0 2.021504 2.938054 0.106392

H 0 -0.789976 2.214222 5.693272

H 0 -3.165288 2.012428 5.104348

H 0 3.411458 1.303484 1.036744

H 0 -1.253796 -1.085746 0.832365

H 0 -2.931620 1.683701 2.542061

H 0 -2.769525 2.355959 0.099825

H 0 -4.325259 -1.899223 3.018290

H 0 -2.735729 -2.264992 4.891242

H 0 -0.577259 3.095727 0.035157

H 0 -0.337060 -2.048605 5.453078

H 0 1.178402 -1.391113 1.145980

H 0 -3.573960 -1.152068 0.832031

H 0 -3.080347 0.703917 -0.361221

H 0 -4.407809 1.480349 1.598047

H 0 -2.566876 -0.599500 2.482434

H 0 2.785454 2.646232 -0.514499

H 0 0.651597 0.197356 5.859221

H 0 -6.072251 1.995073 0.067830

H 0 -5.793848 -1.024479 1.452418

H 0 -5.005186 -1.125115 5.005523

C 0 -5.431298 0.925659 4.633640

H 0 -6.470658 0.674717 4.413850

H 0 -5.374102 1.264176 5.670000

H 0 -5.135071 1.768703 4.010569

**TS4R** (-8045.683682)

C -1 7.281936 0.461239 1.969289

C 0 5.953355 0.635776 2.715379

C 0 6.131931 0.455750 4.227304

C 0 5.319504 1.997994 2.403934

H 0 7.135457 0.497976 0.887570

H 0 7.991629 1.253895 2.235852

H 0 5.262862 -0.144053 2.364809

H 0 6.819150 1.211029 4.629725

H 0 5.178677 0.554588 4.755405

H 0 6.546190 -0.529875 4.465289

H 0 4.329132 2.097484 2.861831

H 0 5.944441 2.812350 2.791785

H 0 5.223634 2.169661 1.326803

C -1 2.867967 -2.655476 6.139820

C 0 3.036196 -2.066780 4.732380

C 0 2.382100 -2.957882 3.669506

C 0 2.491646 -0.636898 4.635911

H 0 1.812412 -2.772720 6.404837

H 0 3.333093 -3.644715 6.212170

H 0 4.113935 -2.023328 4.519820

H 0 1.298594 -3.008206 3.828205

H 0 2.558482 -2.558420 2.666176

H 0 2.772972 -3.980841 3.703284

H 0 1.404682 -0.615389 4.772024

H 0 2.937031 0.011639 5.397012

H 0 2.706859 -0.211789 3.651893

C -1 0.465887 0.417563 10.771225

C 0 0.944585 0.999575 9.432708

C 0 0.377629 0.219783 8.239807

C 0 0.591022 2.487540 9.324478

H 0 -0.626296 0.476210 10.851496

H 0 0.748523 -0.635476 10.873544

H 0 2.039677 0.911216 9.403604

H 0 -0.716259 0.283385 8.209382

H 0 0.750755 0.615814 7.289038

H 0 0.644913 -0.840329 8.281661

H 0 -0.496431 2.628773 9.337239

H 0 1.011208 3.062015 10.157121

H 0 0.972369 2.919216 8.393316

C -1 -3.085931 -4.133122 12.675983

C 0 -3.292517 -5.513160 11.998747

O 0 -4.378308 -5.840563 11.531922

C 0 -4.263614 -3.200693 12.420838

H 0 -2.937549 -4.300177 13.750804

H 0 -4.350050 -2.981293 11.353977

H 0 -5.199341 -3.662447 12.742154

H 0 -4.129822 -2.257119 12.957789

N 0 -2.199448 -6.359635 11.971185

C -1 -2.114086 -7.479865 11.011957

C 0 -1.326561 -7.090610 9.722905

C 0 -1.433367 -5.611577 9.422598

C 0 -2.649293 -5.032985 9.032663

C 0 -0.344068 -4.768333 9.673895

C 0 -2.778232 -3.648951 8.945774

C 0 -0.468304 -3.380719 9.573568

C 0 -1.693476 -2.816211 9.224749

H 0 -3.148432 -7.727188 10.770579

H 0 -1.705389 -7.694881 8.890759

H 0 -0.270043 -7.352288 9.846680

H 0 -3.514510 -5.659211 8.840920

H 0 -1.318034 -5.929187 12.217740

H 0 0.608370 -5.203680 9.967253

H 0 -3.725898 -3.222458 8.640921

H 0 0.389323 -2.745605 9.772527

H 0 -1.796028 -1.740076 9.142579

C -1 0.152556 -6.400890 -0.188291

C 0 0.890529 -5.243709 -0.874764

C 0 0.206812 -4.849240 -2.189347

C 0 0.999252 -4.028757 0.053369

H 0 0.669856 -6.716598 0.723501

H 0 -0.865412 -6.095384 0.086200

H 0 1.909296 -5.583588 -1.110605

H 0 -0.806927 -4.480841 -1.995713

H 0 0.757878 -4.052654 -2.699516

H 0 0.129342 -5.701826 -2.873315

H 0 0.000452 -3.644997 0.293591

H 0 1.497846 -4.283214 0.994695

H 0 1.563404 -3.220220 -0.421068

C -1 -3.596617 -6.934013 3.872246

C 0 -3.427004 -6.438622 2.436884

S 0 -2.757727 -4.736944 2.251742

C 0 -1.102399 -4.933889 2.984505

H 0 -2.644815 -6.949833 4.411424

H 0 -4.294699 -6.299536 4.425285

H 0 -4.397734 -6.393868 1.936047

H 0 -2.785294 -7.114374 1.860906

H 0 -0.545371 -5.728770 2.485337

H 0 -0.579423 -3.988903 2.831886

H 0 -1.161377 -5.120278 4.056754

C -1 -8.593130 -6.747972 0.713285

C 0 -7.947181 -5.522112 1.323324

C 0 -6.682374 -5.068828 0.931686

C 0 -8.620930 -4.770241 2.297988

C 0 -6.104264 -3.919463 1.481402

C 0 -8.066416 -3.619977 2.848114

C 0 -6.800935 -3.172228 2.438850

O 0 -6.323887 -2.038306 3.012288

H 0 -8.968512 -7.429426 1.484241

H 0 -9.445515 -6.462800 0.085404

H 0 -6.125166 -5.623223 0.179215

H 0 -9.609257 -5.085222 2.621683

H 0 -5.115313 -3.605922 1.171158

H 0 -8.602530 -3.043537 3.594479

H 0 -5.466244 -1.729114 2.619943

C -1 -3.717542 -4.902546 -2.923715

C 0 -3.706826 -3.815403 -1.849976

C 0 -4.946232 -2.916980 -1.901577

C 0 -5.013551 -1.879123 -0.797142

O 0 -3.875122 -1.703758 -0.167185

O 0 -6.063965 -1.288575 -0.533525

H 0 -3.745561 -4.470352 -3.930347

H 0 -2.812708 -3.193267 -1.952605

H 0 -3.632756 -4.272310 -0.856846

H 0 -5.869237 -3.502595 -1.826083

H 0 -5.007798 -2.387274 -2.861685

C -1 2.462994 -1.588731 -6.455212

C 0 2.658124 -1.372134 -4.969240

C 0 1.561027 -1.360216 -4.096130

C 0 3.936532 -1.205270 -4.421268

C 0 1.733279 -1.203309 -2.719331

C 0 4.120350 -1.057964 -3.044000

C 0 3.017419 -1.063772 -2.185927

H 0 2.421090 -2.658284 -6.694162

H 0 1.527164 -1.140947 -6.803042

H 0 0.559323 -1.474369 -4.501427

H 0 4.800618 -1.193535 -5.081000

H 0 0.869412 -1.182779 -2.062360

H 0 5.121297 -0.912543 -2.645358

H 0 3.150501 -0.944759 -1.114931

C -1 2.557142 4.063955 -4.609240

C 0 2.998474 3.793455 -3.165357

C 0 3.508051 2.366941 -2.967975

N 0 3.963601 2.144628 -1.580006

H 0 3.385552 3.914434 -5.310405

H 0 1.747271 3.386987 -4.901256

H 0 2.169043 3.969681 -2.469683

H 0 3.791192 4.502555 -2.884687

H 0 4.309173 2.152470 -3.690386

H 0 2.705394 1.651544 -3.169305

H 0 4.762700 2.743764 -1.371529

H 0 4.317893 1.192788 -1.502070

C -1 -2.422134 0.948935 -5.153470

C 0 -2.093020 1.019740 -3.654980

C 0 -2.139984 -0.373393 -3.015722

C 0 -0.735699 1.696674 -3.419837

H 0 -1.674293 0.345731 -5.682910

H 0 -2.867103 1.628770 -3.165133

H 0 -3.131454 -0.823478 -3.127963

H 0 -1.417102 -1.045956 -3.492401

H 0 -1.910273 -0.338148 -1.948041

H 0 0.068170 1.130238 -3.904122

H 0 -0.722918 2.714126 -3.828405

H 0 -0.498252 1.755830 -2.353612

C -1 -8.784572 -2.890253 -3.161181

C 0 -9.142277 -3.890060 -2.064087

S 0 -10.365919 -3.274016 -0.847089

C 0 -9.385583 -1.942286 -0.070460

H 0 -9.677615 -2.564519 -3.700259

H 0 -8.297737 -2.006773 -2.740669

H 0 -8.251778 -4.207089 -1.509750

H 0 -9.599613 -4.788971 -2.489874

H 0 -8.408060 -2.318706 0.233019

H 0 -9.268533 -1.093674 -0.744281

H 0 -9.934353 -1.621779 0.816972

C -1 -11.413301 -0.217540 -4.324437

C 0 -10.708269 0.454768 -3.167923

C 0 -9.338670 0.733188 -3.205704

C 0 -11.410248 0.813839 -2.008660

C 0 -8.685138 1.354905 -2.139751

C 0 -10.779618 1.450098 -0.943360

C 0 -9.413029 1.733219 -1.008321

O 0 -8.822635 2.347774 0.075928

H 0 -11.842199 -1.177946 -4.017271

H 0 -12.234171 0.399665 -4.706511

H 0 -8.761308 0.447689 -4.080825

H 0 -12.472610 0.595260 -1.940511

H 0 -7.614109 1.515468 -2.178636

H 0 -11.330398 1.736029 -0.053522

H 0 -8.086525 2.927797 -0.240992

C -1 -5.817614 4.555198 -3.595775

C 0 -4.974245 4.184714 -2.370555

C 0 -5.795000 3.423760 -1.360028

O 0 -6.841280 3.896536 -0.881492

O 0 -5.333581 2.240896 -1.062887

H 0 -6.612228 5.250007 -3.320568

H 0 -4.610810 5.096720 -1.884712

H 0 -4.107256 3.580901 -2.645494

C -1 -9.497210 6.950489 -2.623759

C 0 -8.742727 7.039270 -1.291543

C 0 -9.424161 6.250899 -0.171616

O 0 -7.382314 6.633107 -1.447722

H 0 -9.582124 5.906609 -2.946946

H 0 -8.696775 8.095913 -0.994943

H 0 -10.447117 6.603894 -0.004585

H 0 -9.471320 5.182872 -0.413099

H 0 -8.861870 6.360514 0.761659

H 0 -7.311390 5.677324 -1.263266

N 0 -8.031191 5.559997 5.578838

C 0 -7.378767 4.523212 4.756201

C 0 -5.931538 4.810634 4.362426

O 0 -5.258782 3.923646 3.827792

C 0 -8.252203 4.452646 3.480025

C 0 -9.621873 4.960237 3.943911

C 0 -9.261745 6.063970 4.946053

H 0 -7.370352 3.574763 5.300823

H 0 -8.272960 3.449615 3.052393

H 0 -7.843135 5.122304 2.715438

H 0 -10.169473 4.162382 4.456609

H 0 -10.241059 5.325072 3.120899

H 0 -9.069597 7.014123 4.428877

H 0 -10.042978 6.237565 5.688045

N 0 -5.510213 6.078924 4.545397

C -1 -4.224791 6.582964 4.089082

C 0 -4.360623 7.645044 2.988905

C 0 -5.196600 7.223590 1.769536

C 0 -5.296206 8.368009 0.754298

C 0 -4.663325 5.955168 1.094103

H 0 -3.669283 5.713880 3.731427

H 0 -3.348093 7.918994 2.661887

H 0 -4.797497 8.556680 3.420591

H 0 -6.213276 7.004076 2.126500

H 0 -5.712016 9.274666 1.209109

H 0 -4.303395 8.620219 0.360350

H 0 -6.152783 6.705137 5.008254

H 0 -5.930941 8.073533 -0.086482

H 0 -4.646436 5.102165 1.775867

H 0 -5.307564 5.699014 0.250886

H 0 -3.645492 6.116725 0.716140

C -1 -0.160156 5.153555 5.413530

C 0 -0.743502 3.745827 5.495396

S 0 -0.527199 2.739784 3.976795

C 0 1.291778 2.630132 3.898948

H 0 0.924541 5.130697 5.273379

H 0 -0.597412 5.707414 4.577634

H 0 -1.826921 3.787314 5.640236

H 0 -0.322369 3.195186 6.343567

H 0 1.743269 3.575178 3.591954

H 0 1.698128 2.310264 4.861024

H 0 1.521578 1.875648 3.148654

C -1 -0.794932 9.087920 -2.940778

C 0 -0.598911 7.566259 -2.857049

C 0 -1.474462 6.956571 -1.745290

C 0 0.885759 7.225578 -2.673264

C 0 -1.426859 5.427070 -1.661624

H 0 -0.471214 9.571693 -2.011187

H 0 -0.928708 7.129815 -3.812003

H 0 -2.513190 7.275643 -1.902847

H 0 -1.169471 7.384780 -0.779709

H 0 1.492756 7.680359 -3.463737

H 0 1.253287 7.604930 -1.711649

H 0 1.065840 6.147672 -2.695953

H 0 -0.430163 5.062415 -1.395921

H 0 -2.123149 5.052948 -0.904148

H 0 -1.701111 4.967925 -2.618379

C -1 -8.524729 6.609098 7.776292

C 0 -7.702126 5.656142 6.914215

O 0 -6.766956 5.011376 7.373735

H 0 -9.515433 6.187598 7.978932

H 0 -7.999138 6.736716 8.721612

H 0 -1.846980 9.346155 -3.101051

H 0 -0.211537 9.518719 -3.761103

H 0 2.195670 5.089508 -4.726845

H 0 3.329623 -2.010388 6.896196

H 0 0.893074 0.964874 11.617933

H 0 -1.656184 -8.345074 11.498891

H 0 -2.154845 -3.700182 12.290649

H 0 -3.993507 -7.955317 3.868594

H 0 -7.885241 -7.301921 0.089758

H 0 -8.090033 -3.344891 -3.878471

H 0 -4.591973 -5.554904 -2.821001

H 0 -2.823013 -5.529487 -2.855914

H 0 0.075335 -7.271969 -0.848374

H 0 3.284638 -1.155602 -7.032836

H 0 -2.429047 1.944885 -5.609720

H 0 -3.402306 0.491043 -5.325763

H 0 -5.188974 5.038414 -4.348759

H 0 -8.958236 7.506284 -3.395644

H 0 -10.507545 7.364344 -2.534221

H 0 -6.264370 3.665584 -4.050544

H 0 -10.724030 -0.407511 -5.151584

H 0 -8.668809 7.581482 7.296178

H 0 -3.675171 6.996125 4.943196

H 0 -0.364298 5.703156 6.339302

O 0 7.083274 0.834176 -1.733834

O 0 -6.468742 0.994166 0.789286

H 0 7.872562 0.449806 -1.312902

H 0 6.934732 1.646401 -1.223027

H 0 -7.425915 1.184000 0.762160

H 0 -6.341152 0.059372 0.482083

C -1 5.110882 1.406495 8.853568

C 0 4.358388 2.672271 8.425075

C 0 5.303752 3.877005 8.355796

C 0 3.643224 2.461747 7.085534

H 0 4.434147 0.548564 8.932469

H 0 5.885469 1.150247 8.120657

H 0 3.593609 2.885086 9.184907

H 0 6.086345 3.711105 7.605425

H 0 4.765844 4.790233 8.079513

H 0 5.796702 4.053885 9.317641

H 0 3.072834 3.350525 6.793920

H 0 4.364635 2.253545 6.286678

H 0 2.948637 1.617570 7.134808

C -1 -7.139767 -6.830907 9.564104

C 0 -6.896465 -6.007636 8.322068

C 0 -6.301406 -4.741039 8.418004

C 0 -7.289462 -6.461669 7.056708

C 0 -6.133309 -3.944474 7.285202

C 0 -7.115509 -5.670394 5.919272

C 0 -6.542462 -4.403679 6.031025

H 0 -8.054385 -6.503240 10.073676

H 0 -7.264530 -7.890473 9.321068

H 0 -5.970855 -4.389304 9.391939

H 0 -7.744909 -7.444712 6.964788

H 0 -5.682145 -2.960764 7.373951

H 0 -7.437182 -6.027805 4.945372

H 0 -6.429937 -3.782877 5.149663

H 0 -6.314969 -6.726957 10.275140

H 0 5.599233 1.541635 9.824315

H 0 7.753656 -0.497837 2.205904

C -1 6.647950 5.725777 0.042918

C 0 7.067417 4.558250 -0.819059

O 0 6.747069 3.398788 -0.521284

H 0 7.046260 5.574281 1.049448

N 0 7.812107 4.843402 -1.912188

C 0 8.327513 3.829225 -2.819313

C 0 9.716390 3.288450 -2.467948

O 0 10.536220 3.049039 -3.346927

H 0 7.648047 2.969758 -2.812441

H 0 8.112034 5.796525 -2.051606

N 0 9.945021 3.070998 -1.136613

C -1 11.056722 2.193861 -0.765948

C 0 10.600691 0.763874 -0.976939

O 0 9.588620 0.361732 -0.382355

H 0 11.930263 2.476939 -1.347826

H 0 9.118302 2.963166 -0.557690

N 0 11.276395 -0.001638 -1.842443

C -1 10.756982 -1.336955 -2.119978

C 0 11.779852 -1.924479 -3.099445

C 0 12.287952 -0.685622 -3.853133

C 0 12.362755 0.390814 -2.761486

H 0 11.340940 -2.679330 -3.755531

H 0 12.602004 -2.394503 -2.548322

H 0 11.559790 -0.381671 -4.612456

H 0 13.249512 -0.839383 -4.348306

H 0 13.328615 0.361258 -2.241396

H 0 12.185931 1.393819 -3.151295

H 0 9.759096 -1.257061 -2.568746

H 0 5.557805 5.724223 0.123000

H 0 6.980746 6.694086 -0.337154

H 0 8.380629 4.232136 -3.830871

H 0 11.272635 2.336407 0.295719

H 0 10.651029 -1.905807 -1.193549

H 0 -5.860434 1.755964 -0.290229

O 0 -3.921552 -1.049862 2.209211

N 0 -3.866347 1.572747 2.486618

C 0 -1.672303 -3.296859 5.956529

C 0 -3.032850 -3.401177 5.675501

C 0 -3.239028 2.354342 1.415559

C 0 -2.595228 1.549289 0.267877

C 0 -3.734633 -2.292591 5.200653

C 0 -3.088639 -1.070323 4.997205

C 0 -1.156124 1.130925 0.484880

C 0 -3.850176 0.137579 4.479472

C 0 -0.132390 2.080043 0.332276

C 0 1.207026 1.737463 0.494920

C 0 -3.354314 0.505553 3.086965

C 0 1.535002 0.417926 0.862675

C 0 -1.715010 -0.978410 5.263997

C 0 -1.016500 -2.084172 5.742883

C 0 0.536639 -0.537171 1.013230

C 0 -0.800649 -0.183869 0.806945

O 0 2.849630 0.089580 1.087885

O 0 2.210402 2.662125 0.386459

H 0 -3.558709 -4.334362 5.840238

H 0 -4.788494 -2.375002 4.961016

H 0 3.339191 0.929848 1.085255

H 0 -1.572353 -0.939335 0.902130

H 0 -2.495221 3.030550 1.851555

H 0 -2.634003 2.196019 -0.614666

H 0 -2.299610 0.344843 2.892408

H 0 -1.191602 -0.040131 5.098871

H 0 -0.366712 3.108647 0.075858

H 0 0.038674 -1.990467 5.964585

H 0 0.809531 -1.546318 1.296392

H 0 -4.774917 1.906714 2.784178

H 0 -3.217906 0.686212 0.031364

H 0 -4.039221 2.966950 1.003506

H 0 2.825510 2.444310 -0.397446

H 0 -3.378190 -1.764981 2.580873

H 0 -3.953042 -1.280936 0.795650

H 0 -1.131696 -4.143731 6.366551

C 0 -3.733754 1.328471 5.452507

H 0 -4.293943 2.194893 5.095359

H 0 -4.125176 1.032173 6.429854

H 0 -2.690074 1.620712 5.582792

H 0 -4.907885 -0.127039 4.382947

**Int6R** (-8045.700062)

C -1 7.281562 0.460786 1.969170

C 0 5.753271 0.485641 2.004212

C 0 5.208976 -0.626796 2.907643

C 0 5.241417 1.865948 2.442050

H 0 7.685467 1.249004 1.329185

H 0 7.692259 0.606074 2.976771

H 0 5.410265 0.293934 0.977386

H 0 5.531078 -0.470684 3.944277

H 0 4.117404 -0.655843 2.883371

H 0 5.575113 -1.608347 2.588182

H 0 4.146677 1.918821 2.457659

H 0 5.588261 2.095591 3.457166

H 0 5.605830 2.648540 1.769784

C -1 2.867004 -2.655587 6.140396

C 0 2.052607 -1.777439 5.177051

C 0 1.256084 -2.627107 4.181582

C 0 1.112488 -0.833344 5.934719

H 0 2.206972 -3.324396 6.706252

H 0 3.584613 -3.276684 5.594605

H 0 2.759725 -1.160941 4.607418

H 0 0.495861 -3.217674 4.705737

H 0 0.739439 -1.999088 3.449073

H 0 1.903158 -3.316821 3.630208

H 0 0.396808 -1.400800 6.541719

H 0 1.663753 -0.169160 6.606945

H 0 0.536919 -0.209525 5.241558

C -1 0.466056 0.417268 10.771098

C 0 0.022161 0.174546 9.322032

C 0 -1.329491 -0.544548 9.259310

C 0 -0.029306 1.484566 8.528030

H 0 -0.243939 1.071690 11.291107

H 0 0.523322 -0.522700 11.330440

H 0 0.772034 -0.473941 8.848362

H 0 -2.114039 0.058078 9.732912

H 0 -1.639675 -0.726277 8.225381

H 0 -1.295286 -1.511287 9.766902

H 0 -0.784794 2.161893 8.944816

H 0 0.932170 2.006636 8.543487

H 0 -0.290918 1.295005 7.481814

C -1 -3.085943 -4.133143 12.676019

C 0 -3.273112 -5.453233 11.909223

O 0 -4.327725 -5.709656 11.335106

C 0 -2.738638 -3.011978 11.685631

H 0 -4.031156 -3.923761 13.182138

H 0 -1.809649 -3.239758 11.154763

H 0 -3.527982 -2.907006 10.936945

H 0 -2.617674 -2.053796 12.198647

N 0 -2.190456 -6.293808 11.870205

C -1 -2.114308 -7.479788 11.011691

C 0 -1.026331 -7.350093 9.931999

C 0 -1.181000 -6.142995 9.032166

C 0 -2.224510 -6.065770 8.099464

C 0 -0.278360 -5.076167 9.110077

C 0 -2.346700 -4.965163 7.251921

C 0 -0.400857 -3.966767 8.270131

C 0 -1.432238 -3.912984 7.333142

H 0 -3.103484 -7.577881 10.560117

H 0 -1.042442 -8.270499 9.334931

H 0 -0.042007 -7.316723 10.415552

H 0 -2.952344 -6.869820 8.034883

H 0 -1.339276 -5.981907 12.314389

H 0 0.538780 -5.121038 9.826049

H 0 -3.156613 -4.926529 6.533648

H 0 0.311613 -3.151070 8.344553

H 0 -1.525010 -3.060057 6.668414

C -1 0.152996 -6.400884 -0.188202

C 0 0.729233 -5.011571 -0.493632

C 0 -0.393526 -3.995786 -0.729847

C 0 1.656783 -4.540548 0.633192

H 0 0.946935 -7.143735 -0.052873

H 0 -0.438838 -6.366834 0.734057

H 0 1.322548 -5.083075 -1.416472

H 0 -0.997689 -3.868512 0.175784

H 0 0.006959 -3.015718 -1.002900

H 0 -1.057895 -4.320260 -1.536294

H 0 1.090442 -4.423697 1.564506

H 0 2.463836 -5.258870 0.816377

H 0 2.117609 -3.576759 0.391050

C -1 -3.593702 -6.933662 3.872229

C 0 -2.810092 -6.225432 2.770519

S 0 -1.585396 -4.999330 3.371350

C 0 -0.448126 -6.099304 4.281448

H 0 -2.933045 -7.493034 4.540353

H 0 -4.162705 -6.222818 4.478312

H 0 -3.478375 -5.649652 2.123050

H 0 -2.289138 -6.947869 2.132667

H 0 -0.125185 -6.928160 3.645350

H 0 0.423774 -5.500368 4.547793

H 0 -0.901629 -6.476682 5.199933

C -1 -8.593107 -6.748069 0.712690

C 0 -7.963177 -5.472053 1.236457

C 0 -6.759470 -5.472528 1.957247

C 0 -8.573174 -4.233366 1.006561

C 0 -6.182515 -4.286046 2.412572

C 0 -8.003149 -3.037127 1.441290

C 0 -6.799288 -3.059542 2.145546

O 0 -6.208156 -1.918221 2.629442

H 0 -9.443612 -6.522037 0.064659

H 0 -7.872222 -7.337568 0.135623

H 0 -6.253703 -6.413510 2.159784

H 0 -9.517131 -4.215466 0.474000

H 0 -5.245670 -4.293637 2.958898

H 0 -8.488516 -2.087581 1.233220

H 0 -6.327204 -1.171172 2.016404

C -1 -3.718560 -4.901589 -2.924623

C 0 -3.808351 -3.545220 -2.220577

C 0 -4.148167 -3.648196 -0.721869

C 0 -4.467487 -2.287673 -0.112011

O 0 -3.709797 -1.787507 0.755540

O 0 -5.541452 -1.738433 -0.555245

H 0 -4.674926 -5.434272 -2.873144

H 0 -4.560728 -2.928923 -2.720158

H 0 -2.856744 -3.013882 -2.327739

H 0 -3.324229 -4.107750 -0.170471

H 0 -5.041488 -4.270466 -0.590589

C -1 2.463000 -1.589014 -6.455083

C 0 2.722076 -1.393890 -4.978050

C 0 1.663020 -1.420905 -4.060865

C 0 4.019410 -1.206983 -4.483888

C 0 1.890194 -1.278665 -2.692182

C 0 4.258487 -1.079020 -3.113268

C 0 3.192443 -1.123181 -2.211175

H 0 3.254878 -1.141617 -7.062887

H 0 2.416668 -2.654859 -6.709719

H 0 0.648317 -1.553032 -4.425083

H 0 4.852906 -1.164697 -5.180910

H 0 1.054003 -1.273911 -2.001252

H 0 5.269666 -0.917749 -2.748891

H 0 3.364457 -1.021752 -1.144669

C -1 2.557164 4.064019 -4.608881

C 0 2.974514 3.771090 -3.162138

C 0 3.526457 2.357241 -2.986919

N 0 3.963724 2.116972 -1.595979

H 0 3.407065 3.965244 -5.293060

H 0 1.781313 3.365401 -4.938736

H 0 2.124263 3.902675 -2.481996

H 0 3.736752 4.498261 -2.845444

H 0 4.346305 2.184415 -3.699581

H 0 2.751369 1.620547 -3.217520

H 0 4.749638 2.723075 -1.360604

H 0 4.329018 1.168054 -1.531433

C -1 -2.421995 0.948898 -5.153503

C 0 -1.930829 0.964841 -3.699764

C 0 -2.020395 -0.441026 -3.096840

C 0 -0.518399 1.553546 -3.592057

H 0 -1.769385 0.319535 -5.770664

H 0 -2.604355 1.610829 -3.118378

H 0 -3.057296 -0.789022 -3.085978

H 0 -1.441607 -1.157683 -3.691656

H 0 -1.633019 -0.469468 -2.075114

H 0 0.196395 0.965029 -4.177500

H 0 -0.491800 2.584070 -3.966354

H 0 -0.168870 1.560433 -2.555266

C -1 -8.784270 -2.890404 -3.160900

C 0 -9.094893 -4.200732 -2.454783

S 0 -10.863170 -4.404127 -1.980785

C 0 -11.228985 -2.796159 -1.192525

H 0 -9.414573 -2.751054 -4.044426

H 0 -8.934214 -2.040908 -2.495710

H 0 -8.472626 -4.306810 -1.562442

H 0 -8.898888 -5.061292 -3.101777

H 0 -10.539217 -2.575680 -0.374688

H 0 -11.200502 -1.976308 -1.908780

H 0 -12.239030 -2.877688 -0.785374

C -1 -11.413380 -0.217171 -4.324352

C 0 -10.660760 0.359362 -3.154159

C 0 -9.262133 0.384383 -3.152514

C 0 -11.326582 0.814067 -2.008919

C 0 -8.546652 0.823429 -2.044201

C 0 -10.624138 1.279697 -0.897751

C 0 -9.229963 1.288072 -0.918026

O 0 -8.550519 1.746763 0.196604

H 0 -11.508170 -1.306757 -4.234424

H 0 -12.425403 0.192609 -4.395234

H 0 -8.706620 0.019010 -4.010534

H 0 -12.413240 0.804804 -1.985467

H 0 -7.467958 0.759126 -2.068363

H 0 -11.139801 1.649475 -0.017948

H 0 -7.833721 2.342983 -0.119512

C -1 -5.818619 4.554548 -3.596897

C 0 -4.956060 3.640417 -2.716807

C 0 -5.697983 2.858336 -1.656238

O 0 -6.640767 3.309532 -0.993889

O 0 -5.216027 1.653101 -1.445664

H 0 -6.382570 5.270027 -2.997704

H 0 -4.219318 4.241785 -2.166142

H 0 -4.382370 2.929435 -3.316125

C -1 -9.497379 6.950049 -2.623579

C 0 -9.238314 5.626732 -1.925433

C 0 -9.426967 4.440124 -2.869953

O 0 -7.911480 5.715734 -1.415048

H 0 -8.818216 7.060194 -3.475205

H 0 -9.946371 5.516350 -1.085671

H 0 -10.474207 4.360701 -3.178857

H 0 -8.814261 4.561942 -3.768356

H 0 -9.154073 3.496274 -2.394104

H 0 -7.574535 4.828005 -1.203411

N 0 -7.466535 4.943441 6.257457

C 0 -6.382910 3.995167 5.913244

C 0 -5.193812 4.572013 5.144704

O 0 -4.248304 3.829635 4.852213

C 0 -7.079866 2.914278 5.055797

C 0 -8.546763 2.995387 5.487416

C 0 -8.755376 4.499273 5.698527

H 0 -5.972265 3.567786 6.832802

H 0 -6.628056 1.935267 5.209872

H 0 -6.986425 3.166226 3.993773

H 0 -8.690534 2.465999 6.435836

H 0 -9.235203 2.574228 4.750933

H 0 -8.953483 5.003091 4.743043

H 0 -9.577504 4.727959 6.377880

N 0 -5.287871 5.864824 4.773841

C -1 -4.224644 6.582585 4.088768

C 0 -4.695260 7.321885 2.836096

C 0 -5.413031 6.466328 1.779747

C 0 -5.735800 7.326642 0.552247

C 0 -4.610304 5.219458 1.385558

H 0 -3.468497 5.835310 3.841938

H 0 -3.809693 7.788297 2.382642

H 0 -5.355936 8.150571 3.126439

H 0 -6.361096 6.122487 2.216887

H 0 -6.250760 8.249588 0.843629

H 0 -4.810951 7.616638 0.036461

H 0 -6.079984 6.380820 5.127196

H 0 -6.379225 6.801026 -0.156911

H 0 -4.445507 4.552172 2.236419

H 0 -5.145539 4.657625 0.614502

H 0 -3.628085 5.500580 0.983288

C -1 -0.160246 5.153726 5.413248

C 0 -0.872679 3.802955 5.385618

S 0 -0.636561 2.839922 3.839760

C 0 1.147670 2.458282 3.905657

H 0 0.926047 5.038642 5.348021

H 0 -0.485383 5.782799 4.579588

H 0 -1.957617 3.931942 5.446746

H 0 -0.559017 3.181920 6.230609

H 0 1.755504 3.342573 3.706734

H 0 1.411736 2.028428 4.875063

H 0 1.336907 1.721633 3.124049

C -1 -0.794238 9.087968 -2.940210

C 0 -0.599717 7.565416 -2.865601

C 0 -1.504382 6.947570 -1.782407

C 0 0.879325 7.222313 -2.644761

C 0 -1.469256 5.416780 -1.714939

H 0 -0.497918 9.562842 -1.997108

H 0 -0.904175 7.137716 -3.832848

H 0 -2.537367 7.273970 -1.960809

H 0 -1.219589 7.361917 -0.804931

H 0 1.506988 7.677964 -3.418314

H 0 1.222377 7.598963 -1.673146

H 0 1.058719 6.144139 -2.664991

H 0 -0.481447 5.039890 -1.433455

H 0 -2.181559 5.043049 -0.971263

H 0 -1.728298 4.969146 -2.681717

C -1 -8.525069 6.609711 7.776862

C 0 -7.308081 5.798676 7.326493

O 0 -6.216538 5.920953 7.872755

H 0 -9.237268 5.972494 8.312302

H 0 -8.169538 7.384021 8.455252

H 0 -1.841219 9.347452 -3.128976

H 0 -0.187397 9.526678 -3.739069

H 0 2.160010 5.078358 -4.708136

H 0 3.424940 -2.045954 6.859378

H 0 1.451052 0.894928 10.810030

H 0 -1.928134 -8.370348 11.623169

H 0 -2.304188 -4.228947 13.438917

H 0 -4.300456 -7.648095 3.434350

H 0 -8.950468 -7.385288 1.530340

H 0 -7.735646 -2.860161 -3.474514

H 0 -2.958632 -5.544032 -2.464247

H 0 -3.458425 -4.778945 -3.980686

H 0 -0.501173 -6.747532 -0.995812

H 0 1.509783 -1.142601 -6.754238

H 0 -2.423732 1.953934 -5.590976

H 0 -3.436894 0.542435 -5.222968

H 0 -5.173817 5.098336 -4.292427

H 0 -9.321228 7.783842 -1.937932

H 0 -10.527526 7.011546 -2.989324

H 0 -6.528140 3.965010 -4.184338

H 0 -10.898571 -0.016106 -5.268364

H 0 -9.052686 7.065662 6.934332

H 0 -3.761484 7.293236 4.785612

H 0 -0.384486 5.677006 6.349409

O 0 7.015334 0.770289 -1.556978

O 0 -6.167277 0.371758 0.552481

H 0 7.809524 0.377217 -1.154455

H 0 6.869887 1.567428 -1.023308

H 0 -5.648653 1.186518 -0.617207

H 0 -7.127054 0.554403 0.536704

C -1 5.110791 1.406399 8.853652

C 0 4.028351 1.476334 7.767401

C 0 3.493898 2.904961 7.611456

C 0 4.549928 0.942928 6.428031

H 0 5.475762 0.382363 8.985331

H 0 5.968928 2.034573 8.585908

H 0 3.192522 0.835911 8.085234

H 0 4.292538 3.582794 7.286638

H 0 2.694836 2.952737 6.863827

H 0 3.094809 3.290305 8.555913

H 0 3.775528 0.969519 5.655148

H 0 5.392313 1.548068 6.071684

H 0 4.897426 -0.091555 6.514702

C -1 -7.139747 -6.830862 9.564115

C 0 -6.721379 -5.961946 8.396737

C 0 -5.943081 -4.813509 8.608082

C 0 -7.119528 -6.261313 7.087025

C 0 -5.601530 -3.977065 7.544649

C 0 -6.769357 -5.433745 6.017641

C 0 -6.014075 -4.282215 6.245116

H 0 -6.354982 -6.858950 10.324736

H 0 -8.045466 -6.434634 10.039718

H 0 -5.592987 -4.594887 9.612076

H 0 -7.714718 -7.152716 6.903285

H 0 -5.004402 -3.088272 7.725845

H 0 -7.089709 -5.677023 5.008650

H 0 -5.751806 -3.630906 5.417719

H 0 -7.361118 -7.852689 9.240983

H 0 4.729326 1.756156 9.818958

H 0 7.661403 -0.491360 1.588080

C -1 6.647836 5.725844 0.042986

C 0 7.050423 4.518545 -0.769598

O 0 6.771876 3.372616 -0.387558

H 0 7.105984 5.646632 1.032221

N 0 7.731251 4.752345 -1.914678

C 0 8.231362 3.695820 -2.781847

C 0 9.648350 3.212121 -2.460837

O 0 10.444585 2.959584 -3.357516

H 0 7.577314 2.820984 -2.688526

H 0 8.000553 5.701590 -2.125197

N 0 9.926724 3.051563 -1.130278

C -1 11.057096 2.194067 -0.766067

C 0 10.614587 0.756735 -0.963274

O 0 9.617355 0.346244 -0.349998

H 0 11.920521 2.483546 -1.359941

H 0 9.118065 2.952028 -0.525020

N 0 11.280527 -0.003404 -1.841422

C -1 10.756992 -1.337019 -2.120027

C 0 11.758325 -1.915160 -3.126821

C 0 12.252633 -0.669064 -3.877492

C 0 12.350638 0.395533 -2.776354

H 0 11.304498 -2.662315 -3.781575

H 0 12.591003 -2.392321 -2.598084

H 0 11.509397 -0.355454 -4.618035

H 0 13.203692 -0.818598 -4.393826

H 0 13.325757 0.358508 -2.274331

H 0 12.168159 1.401724 -3.154290

H 0 9.749182 -1.253166 -2.545232

H 0 5.564240 5.703275 0.184639

H 0 6.932404 6.676852 -0.412592

H 0 8.227793 4.036725 -3.817212

H 0 11.282418 2.348649 0.291672

H 0 10.672010 -1.915172 -1.197125

H 0 -5.946477 -0.554142 0.090461

N 0 -3.435637 0.564425 2.226228

C 0 -5.984652 0.393899 7.656723

C 0 -4.730256 1.006634 7.687379

C 0 -3.422295 1.703783 1.291979

C 0 -2.622082 1.349641 0.032084

C 0 -3.809645 0.760538 6.669739

C 0 -4.131235 -0.096781 5.611377

C 0 -1.177131 0.991327 0.282879

C 0 -3.105660 -0.351622 4.504446

C 0 -0.178160 1.970995 0.199669

C 0 1.164392 1.645915 0.382898

C 0 -3.263100 0.727711 3.486342

C 0 1.505387 0.316646 0.696302

C 0 -5.400538 -0.684756 5.567431

C 0 -6.316249 -0.449820 6.595090

C 0 0.524629 -0.665422 0.786148

C 0 -0.812532 -0.331316 0.567621

O 0 2.815288 -0.010380 0.912664

O 0 2.153266 2.589576 0.328774

H 0 -4.463339 1.668039 8.505884

H 0 -2.833971 1.236680 6.698630

H 0 3.311593 0.824043 0.923847

H 0 -1.572871 -1.105557 0.602691

H 0 -2.981783 2.554388 1.814712

H 0 -2.686614 2.213958 -0.634612

H 0 -3.214178 1.752508 3.847804

H 0 -5.680867 -1.316042 4.731685

H 0 -0.428766 3.003120 -0.025069

H 0 -7.286626 -0.934826 6.563799

H 0 0.817036 -1.683442 1.015877

H 0 -3.581316 -0.377722 1.777113

H 0 -3.126336 0.525758 -0.475745

H 0 -4.458694 1.926636 1.040606

H 0 2.798443 2.394127 -0.437235

H 0 -6.696846 0.569753 8.457202

O 0 -5.814755 -0.732126 -3.155471

H 0 -5.823374 -1.234085 -2.315716

H 0 -5.319589 0.062104 -2.912884

C 0 -3.075207 -1.772240 3.941192

H 0 -2.946963 -2.489520 4.753533

H 0 -3.988251 -2.021185 3.398997

H 0 -2.237566 -1.895468 3.251538

H 0 -2.119976 -0.121749 4.938172

**Int7R** (-8045.707141)

C -1 7.281668 0.460928 1.968787

C 0 5.751423 0.488541 1.964081

C 0 5.188336 -0.615300 2.865921

C 0 5.226750 1.871216 2.377641

H 0 7.703356 1.235577 1.324009

H 0 7.664483 0.624859 2.984379

H 0 5.432152 0.290033 0.929631

H 0 5.511453 -0.462248 3.902587

H 0 4.097311 -0.631189 2.846432

H 0 5.540791 -1.602626 2.548382

H 0 4.133429 1.933615 2.333595

H 0 5.525774 2.098177 3.408685

H 0 5.628433 2.650523 1.723484

C -1 2.866908 -2.656138 6.141207

C 0 2.199612 -1.850673 5.018644

C 0 2.199362 -2.641781 3.707150

C 0 0.776218 -1.422915 5.402954

H 0 2.310179 -3.580530 6.338432

H 0 3.891941 -2.931395 5.873137

H 0 2.791810 -0.940023 4.859740

H 0 1.623203 -3.567600 3.813672

H 0 1.765526 -2.058276 2.891370

H 0 3.215139 -2.916535 3.408209

H 0 0.142931 -2.299498 5.582502

H 0 0.771016 -0.816836 6.316943

H 0 0.312739 -0.833572 4.603388

C -1 0.459322 0.413205 10.775798

C 0 -0.195902 0.494994 9.389290

C 0 -1.325848 -0.531036 9.245273

C 0 -0.711556 1.910282 9.104051

H 0 -0.268273 0.648401 11.561935

H 0 0.848758 -0.591624 10.971505

H 0 0.574811 0.260014 8.640258

H 0 -2.125854 -0.330939 9.968242

H 0 -1.774842 -0.497887 8.246987

H 0 -0.971630 -1.550981 9.417236

H 0 -1.492484 2.190480 9.821209

H 0 0.087516 2.655046 9.172333

H 0 -1.146710 1.977095 8.101147

C -1 -3.085938 -4.133166 12.676100

C 0 -3.268061 -5.441800 11.888666

O 0 -4.312842 -5.682104 11.289988

C 0 -2.776960 -2.990290 11.696051

H 0 -4.025337 -3.946040 13.201891

H 0 -1.846648 -3.181587 11.153097

H 0 -3.576267 -2.900865 10.956093

H 0 -2.678570 -2.035834 12.220893

N 0 -2.190756 -6.290424 11.864216

C -1 -2.114357 -7.480125 11.011909

C 0 -1.104046 -7.322743 9.863412

C 0 -1.355321 -6.123126 8.975001

C 0 -2.539335 -6.005705 8.234776

C 0 -0.400682 -5.104813 8.870521

C 0 -2.758962 -4.906004 7.406334

C 0 -0.615192 -3.998600 8.045786

C 0 -1.798320 -3.895909 7.313955

H 0 -3.124432 -7.627970 10.625468

H 0 -1.131537 -8.246053 9.270087

H 0 -0.091068 -7.257802 10.279272

H 0 -3.307232 -6.769363 8.312776

H 0 -1.344143 -5.985528 12.321553

H 0 0.525682 -5.183499 9.434234

H 0 -3.685703 -4.831483 6.850301

H 0 0.139523 -3.221983 7.973625

H 0 -1.976398 -3.029917 6.683594

C -1 0.153098 -6.400938 -0.188108

C 0 0.748773 -5.050328 -0.610190

C 0 -0.346803 -4.098425 -1.104908

C 0 1.540575 -4.413542 0.537830

H 0 0.929987 -7.092932 0.154911

H 0 -0.562797 -6.257040 0.629965

H 0 1.444943 -5.229126 -1.441872

H 0 -1.060354 -3.884396 -0.300781

H 0 0.076290 -3.149194 -1.444759

H 0 -0.902721 -4.534365 -1.940989

H 0 0.877695 -4.188363 1.381344

H 0 2.327811 -5.081303 0.904775

H 0 2.015716 -3.477953 0.225293

C -1 -3.593502 -6.933648 3.872192

C 0 -3.025726 -6.456376 2.538021

S 0 -2.518310 -4.694449 2.506137

C 0 -1.109313 -4.724218 3.662363

H 0 -2.857269 -6.852858 4.677031

H 0 -4.468636 -6.346556 4.165329

H 0 -3.778801 -6.529625 1.747357

H 0 -2.172739 -7.069752 2.228085

H 0 -0.338237 -5.420234 3.320916

H 0 -0.691256 -3.716644 3.665169

H 0 -1.418216 -4.974261 4.679079

C -1 -8.593144 -6.748043 0.712697

C 0 -8.004791 -5.459330 1.255451

C 0 -6.759223 -5.423203 1.899738

C 0 -8.691808 -4.246652 1.119456

C 0 -6.204493 -4.225886 2.355650

C 0 -8.148325 -3.038541 1.558093

C 0 -6.891209 -3.022873 2.163590

O 0 -6.301078 -1.862246 2.606958

H 0 -9.438458 -6.538719 0.052448

H 0 -7.846803 -7.314087 0.145336

H 0 -6.201042 -6.346482 2.036825

H 0 -9.667642 -4.254972 0.646176

H 0 -5.227167 -4.204066 2.825741

H 0 -8.690808 -2.107367 1.419454

H 0 -6.471001 -1.116360 2.000866

C -1 -3.718631 -4.901625 -2.924638

C 0 -3.853599 -3.530008 -2.263728

C 0 -4.278415 -3.635881 -0.784492

C 0 -4.682422 -2.299025 -0.218265

O 0 -3.984602 -1.619005 0.529011

O 0 -5.890189 -1.919783 -0.638063

H 0 -4.678925 -5.429113 -2.934687

H 0 -4.577397 -2.923780 -2.816540

H 0 -2.901589 -2.993915 -2.317188

H 0 -3.462327 -4.034285 -0.178305

H 0 -5.143058 -4.301724 -0.692312

C -1 2.462980 -1.589028 -6.455013

C 0 2.728830 -1.386275 -4.979957

C 0 1.670026 -1.377599 -4.062046

C 0 4.030596 -1.225749 -4.488021

C 0 1.902035 -1.229279 -2.694794

C 0 4.274154 -1.091684 -3.118659

C 0 3.208293 -1.104193 -2.215726

H 0 3.270124 -1.176920 -7.067559

H 0 2.378957 -2.655506 -6.696978

H 0 0.651873 -1.483964 -4.424079

H 0 4.864268 -1.208691 -5.185857

H 0 1.066312 -1.195372 -2.004002

H 0 5.288514 -0.948645 -2.756209

H 0 3.381007 -1.004221 -1.149215

C -1 2.557034 4.064127 -4.608964

C 0 2.920292 3.760636 -3.149108

C 0 3.519585 2.366234 -2.973865

N 0 3.926927 2.118634 -1.573879

H 0 3.440045 4.009790 -5.255166

H 0 1.823354 3.343556 -4.984596

H 0 2.034354 3.846586 -2.508343

H 0 3.637346 4.511341 -2.785537

H 0 4.367481 2.235927 -3.662499

H 0 2.780674 1.604754 -3.239119

H 0 4.681384 2.752665 -1.309098

H 0 4.335661 1.186496 -1.520909

C -1 -2.421964 0.948875 -5.153517

C 0 -1.919613 0.973243 -3.704604

C 0 -1.995554 -0.433513 -3.101542

C 0 -0.513343 1.578047 -3.605498

H 0 -1.770255 0.322226 -5.775312

H 0 -2.599230 1.610181 -3.122087

H 0 -3.035540 -0.770524 -3.077683

H 0 -1.426737 -1.150730 -3.706227

H 0 -1.592069 -0.461155 -2.085990

H 0 0.204731 1.002835 -4.200421

H 0 -0.502744 2.610873 -3.974213

H 0 -0.154397 1.584243 -2.571525

C -1 -8.784099 -2.890382 -3.160715

C 0 -9.076838 -4.135597 -2.337983

S 0 -10.847493 -4.337468 -1.883474

C 0 -11.204275 -2.713463 -1.125113

H 0 -9.393178 -2.855645 -4.068790

H 0 -8.975028 -1.984228 -2.586593

H 0 -8.475010 -4.138696 -1.425075

H 0 -8.842167 -5.048148 -2.894798

H 0 -10.488761 -2.476134 -0.334396

H 0 -11.196337 -1.909835 -1.859985

H 0 -12.201987 -2.785673 -0.687228

C -1 -11.413623 -0.217114 -4.324539

C 0 -10.656758 0.383221 -3.168606

C 0 -9.258644 0.441420 -3.186213

C 0 -11.316384 0.841288 -2.021191

C 0 -8.538461 0.920161 -2.097601

C 0 -10.610063 1.345038 -0.929068

C 0 -9.216372 1.392629 -0.969084

O 0 -8.534036 1.908721 0.112106

H 0 -11.486165 -1.308194 -4.228692

H 0 -12.434480 0.172234 -4.383754

H 0 -8.707647 0.085973 -4.052036

H 0 -12.402410 0.809283 -1.983154

H 0 -7.458500 0.917536 -2.134946

H 0 -11.123874 1.723346 -0.051611

H 0 -7.787316 2.485299 -0.243101

C -1 -5.818714 4.554605 -3.596919

C 0 -4.980785 3.628899 -2.698570

C 0 -5.733343 2.824282 -1.639085

O 0 -6.660224 3.378432 -0.972195

O 0 -5.351812 1.630892 -1.424206

H 0 -6.363249 5.296224 -3.013097

H 0 -4.247850 4.231476 -2.143675

H 0 -4.405127 2.919514 -3.298638

C -1 -9.497460 6.950095 -2.623687

C 0 -9.185616 5.627547 -1.945270

C 0 -9.340020 4.457454 -2.917398

O 0 -7.866177 5.756000 -1.433722

H 0 -8.816495 7.101366 -3.467493

H 0 -9.891751 5.474332 -1.109395

H 0 -10.392017 4.325995 -3.191403

H 0 -8.767925 4.638225 -3.832234

H 0 -8.993569 3.521581 -2.478789

H 0 -7.486922 4.872325 -1.244042

N 0 -7.501220 4.984642 6.192474

C 0 -6.456008 4.003713 5.826952

C 0 -5.231471 4.572090 5.110841

O 0 -4.299768 3.815234 4.804010

C 0 -7.179219 3.000138 4.897616

C 0 -8.657163 3.150777 5.271695

C 0 -8.785372 4.652220 5.551866

H 0 -6.085952 3.515548 6.732490

H 0 -6.792326 1.990574 5.030820

H 0 -7.026393 3.284874 3.851015

H 0 -8.875670 2.586153 6.184881

H 0 -9.334587 2.809959 4.485190

H 0 -8.904212 5.213959 4.615825

H 0 -9.624491 4.898807 6.203392

N 0 -5.280332 5.878821 4.796858

C -1 -4.224607 6.582910 4.088923

C 0 -4.738134 7.374771 2.884703

C 0 -5.538301 6.570719 1.846428

C 0 -5.925717 7.476211 0.671631

C 0 -4.785625 5.325880 1.359748

H 0 -3.505598 5.821674 3.781811

H 0 -3.865174 7.828806 2.396153

H 0 -5.355508 8.213164 3.236822

H 0 -6.463372 6.226182 2.330961

H 0 -6.432979 8.382342 1.024055

H 0 -5.028480 7.794607 0.124935

H 0 -6.081306 6.395420 5.129698

H 0 -6.590999 6.966175 -0.029918

H 0 -4.580295 4.631167 2.179771

H 0 -5.372961 4.795257 0.605039

H 0 -3.824477 5.606752 0.908188

C -1 -0.158623 5.154719 5.411923

C 0 -0.928031 3.836724 5.319929

S 0 -0.674616 2.914338 3.750892

C 0 1.020326 2.276074 3.980591

H 0 0.922704 4.995238 5.361355

H 0 -0.440909 5.825980 4.596179

H 0 -2.007888 4.009615 5.356592

H 0 -0.667952 3.173814 6.152522

H 0 1.748515 3.087770 4.030198

H 0 1.082032 1.661318 4.882608

H 0 1.244085 1.659869 3.109325

C -1 -0.794258 9.088147 -2.940244

C 0 -0.605851 7.565565 -2.854133

C 0 -1.498116 6.962666 -1.752514

C 0 0.874778 7.218263 -2.650967

C 0 -1.470666 5.432550 -1.671174

H 0 -0.483229 9.570312 -2.005511

H 0 -0.925861 7.130166 -3.812709

H 0 -2.531788 7.291776 -1.919718

H 0 -1.197089 7.385380 -0.783320

H 0 1.493512 7.663040 -3.438031

H 0 1.233337 7.603538 -1.688229

H 0 1.049003 6.139106 -2.662463

H 0 -0.479270 5.053552 -1.404469

H 0 -2.172984 5.070350 -0.912679

H 0 -1.751909 4.977308 -2.627677

C -1 -8.525168 6.609823 7.776912

C 0 -7.340348 5.754643 7.324820

O 0 -6.270341 5.768911 7.923824

H 0 -9.301653 5.985543 8.232203

H 0 -8.156803 7.311043 8.524408

H 0 -1.842506 9.350390 -3.117329

H 0 -0.196313 9.516957 -3.751211

H 0 2.126345 5.064512 -4.708549

H 0 2.905522 -2.083543 7.075299

H 0 1.290106 1.121077 10.866881

H 0 -1.852824 -8.352369 11.621597

H 0 -2.289713 -4.228161 13.423701

H 0 -3.893314 -7.985344 3.801243

H 0 -8.949128 -7.399590 1.519802

H 0 -7.728241 -2.864594 -3.452747

H 0 -2.998973 -5.531747 -2.390432

H 0 -3.377119 -4.805154 -3.959652

H 0 -0.381836 -6.876607 -1.017407

H 0 1.526047 -1.112690 -6.759201

H 0 -2.435358 1.952846 -5.593097

H 0 -3.435056 0.536936 -5.205430

H 0 -5.160203 5.069372 -4.303500

H 0 -9.361899 7.781159 -1.925011

H 0 -10.526702 6.974170 -2.997414

H 0 -6.543648 3.975126 -4.175847

H 0 -10.916325 -0.010546 -5.276822

H 0 -8.980655 7.156166 6.946117

H 0 -3.706419 7.256156 4.784113

H 0 -0.379512 5.652829 6.362540

O 0 6.994173 0.774303 -1.566243

O 0 -6.438912 0.283545 0.611131

H 0 7.791701 0.387106 -1.164784

H 0 6.854868 1.581309 -1.045574

H 0 -5.961122 0.825685 -0.087828

H 0 -7.331302 0.702347 0.601329

C -1 5.115849 1.409532 8.849456

C 0 4.004387 1.174044 7.816866

C 0 2.910963 2.242619 7.929457

C 0 4.573258 1.122460 6.394263

H 0 5.885610 0.633041 8.789287

H 0 5.603577 2.376854 8.678084

H 0 3.544254 0.198445 8.031547

H 0 3.315065 3.237466 7.705943

H 0 2.092401 2.051847 7.226986

H 0 2.486747 2.274012 8.938201

H 0 3.787262 0.944784 5.653767

H 0 5.062075 2.069984 6.136516

H 0 5.316479 0.325325 6.290039

C -1 -7.139765 -6.830894 9.564181

C 0 -6.813726 -5.891068 8.423418

C 0 -6.215000 -4.647747 8.677932

C 0 -7.121297 -6.218133 7.096182

C 0 -5.967249 -3.747945 7.640662

C 0 -6.860356 -5.327290 6.051785

C 0 -6.290567 -4.082398 6.322633

H 0 -6.339849 -6.822352 10.310001

H 0 -8.064549 -6.524893 10.068304

H 0 -5.922985 -4.405084 9.695258

H 0 -7.575277 -7.181923 6.878581

H 0 -5.506110 -2.788310 7.854872

H 0 -7.109158 -5.594879 5.028668

H 0 -6.093988 -3.386544 5.513190

H 0 -7.284095 -7.855227 9.207768

H 0 4.717607 1.412986 9.869699

H 0 7.669093 -0.499052 1.615781

C -1 6.647786 5.725803 0.042900

C 0 7.051799 4.523463 -0.776083

O 0 6.766629 3.375902 -0.404120

H 0 7.098159 5.636882 1.034862

N 0 7.742336 4.762220 -1.914173

C 0 8.241718 3.708917 -2.785523

C 0 9.655426 3.217496 -2.461898

O 0 10.451144 2.958601 -3.357145

H 0 7.583146 2.836734 -2.699707

H 0 8.015547 5.711858 -2.117784

N 0 9.931812 3.057412 -1.130761

C -1 11.057429 2.194070 -0.765997

C 0 10.609826 0.758899 -0.967826

O 0 9.605899 0.352197 -0.362934

H 0 11.923606 2.481240 -1.356930

H 0 9.122147 2.961720 -0.526322

N 0 11.281098 -0.004124 -1.839375

C -1 10.757127 -1.337091 -2.120031

C 0 11.763428 -1.917519 -3.120537

C 0 12.263274 -0.672798 -3.869789

C 0 12.356411 0.392904 -2.769281

H 0 11.312439 -2.664864 -3.777035

H 0 12.592596 -2.395030 -2.586622

H 0 11.524498 -0.359217 -4.614776

H 0 13.216994 -0.823956 -4.380694

H 0 13.328900 0.355872 -2.262238

H 0 12.176481 1.398805 -3.149262

H 0 9.751891 -1.252096 -2.551059

H 0 5.563095 5.706927 0.176387

H 0 6.939540 6.679247 -0.402923

H 0 8.243237 4.055253 -3.819110

H 0 11.280966 2.345119 0.292663

H 0 10.665992 -1.914398 -1.197156

H 0 -6.168739 -1.065449 -0.175164

O 0 -5.625866 -0.494639 -3.275091

H 0 -5.888491 -1.109436 -2.574832

H 0 -5.354133 0.295699 -2.772026

N 0 -3.554982 0.826723 2.135106

C 0 -6.064601 0.490847 7.604161

C 0 -4.823026 1.128953 7.640727

C 0 -3.516763 1.931599 1.159333

C 0 -2.676183 1.536234 -0.063160

C 0 -3.912515 0.938241 6.602847

C 0 -4.227346 0.107216 5.522460

C 0 -1.264910 1.089775 0.239383

C 0 -3.207290 -0.088664 4.400050

C 0 -0.199224 2.000417 0.209092

C 0 1.112691 1.578996 0.420312

C 0 -3.389719 1.001355 3.396455

C 0 1.358327 0.222845 0.707558

C 0 -5.481174 -0.512480 5.476466

C 0 -6.390643 -0.326360 6.520152

C 0 0.311726 -0.692903 0.743897

C 0 -0.992278 -0.260125 0.499918

O 0 2.638987 -0.188378 0.953974

O 0 2.169800 2.447832 0.424265

H 0 -4.559817 1.770447 8.475899

H 0 -2.951943 1.443339 6.628464

H 0 3.182875 0.617512 0.971546

H 0 -1.797493 -0.987467 0.483453

H 0 -3.096950 2.796772 1.673767

H 0 -2.668522 2.407116 -0.724200

H 0 -3.367310 2.026468 3.758052

H 0 -5.755709 -1.131846 4.629325

H 0 -0.372663 3.051903 0.003347

H 0 -7.350559 -0.831564 6.485332

H 0 0.525830 -1.737421 0.936701

H 0 -3.679997 -0.115310 1.733629

H 0 -3.210652 0.755166 -0.605297

H 0 -4.539042 2.152563 0.852222

H 0 2.771951 2.308092 -0.390040

H 0 -6.771476 0.627115 8.416833

C 0 -3.140019 -1.496796 3.806818

H 0 -2.980958 -2.224294 4.604973

H 0 -4.053509 -1.776725 3.280197

H 0 -2.306799 -1.574136 3.102987

H 0 -2.223806 0.160250 4.827337

**TS5R** (-8045.698545)

C -1 7.281364 0.460780 1.969323

C 0 5.809344 0.859396 1.808080

C 0 4.996872 0.529513 3.066479

C 0 5.683504 2.344065 1.445294

H 0 7.862926 0.678905 1.070211

H 0 7.732056 1.005031 2.809175

H 0 5.400243 0.273959 0.970759

H 0 5.381107 1.085621 3.929571

H 0 3.940418 0.787527 2.947081

H 0 5.047163 -0.537501 3.304440

H 0 4.637899 2.630692 1.293985

H 0 6.090880 2.968102 2.251978

H 0 6.240504 2.559327 0.527840

C -1 2.866947 -2.655866 6.140102

C 0 2.231446 -1.754316 5.073411

C 0 2.056339 -2.498931 3.745940

C 0 0.891387 -1.186270 5.556372

H 0 2.218536 -3.512709 6.358636

H 0 3.834874 -3.046010 5.806527

H 0 2.913368 -0.912221 4.897684

H 0 1.348278 -3.329164 3.853312

H 0 1.683258 -1.829314 2.966716

H 0 3.006190 -2.910944 3.388218

H 0 0.145357 -1.982127 5.652885

H 0 0.986384 -0.695837 6.529792

H 0 0.489103 -0.449343 4.853261

C -1 0.465936 0.417132 10.771183

C 0 0.315439 0.369165 9.244776

C 0 -1.078246 -0.120540 8.838301

C 0 0.615527 1.735344 8.615809

H 0 -0.244078 1.130747 11.206343

H 0 0.268305 -0.562982 11.218952

H 0 1.053664 -0.350501 8.858721

H 0 -1.851399 0.558877 9.216525

H 0 -1.189575 -0.161382 7.751511

H 0 -1.285746 -1.119537 9.234249

H 0 -0.114251 2.482233 8.950216

H 0 1.612777 2.095130 8.891311

H 0 0.563497 1.687936 7.522756

C -1 -3.085846 -4.133046 12.675884

C 0 -3.278202 -5.457146 11.913973

O 0 -4.326926 -5.701902 11.324813

C 0 -2.753469 -3.020366 11.666595

H 0 -4.027729 -3.920460 13.186990

H 0 -1.816614 -3.232848 11.141790

H 0 -3.542305 -2.950418 10.913455

H 0 -2.658701 -2.051236 12.164707

N 0 -2.203118 -6.312202 11.896276

C -1 -2.114478 -7.479919 11.011462

C 0 -1.032317 -7.312273 9.936086

C 0 -1.165084 -6.029139 9.147265

C 0 -2.349263 -5.722342 8.462745

C 0 -0.105576 -5.117219 9.095352

C 0 -2.459444 -4.545805 7.726369

C 0 -0.216479 -3.929430 8.367673

C 0 -1.393065 -3.645241 7.676134

H 0 -3.105125 -7.580213 10.564345

H 0 -1.084029 -8.183122 9.269039

H 0 -0.041381 -7.347140 10.405574

H 0 -3.198934 -6.396381 8.509770

H 0 -1.347417 -5.988201 12.323279

H 0 0.816520 -5.341388 9.626463

H 0 -3.377726 -4.345912 7.187229

H 0 0.613547 -3.230197 8.336768

H 0 -1.483652 -2.729412 7.106606

C -1 0.152978 -6.400765 -0.188131

C 0 0.832695 -5.046599 -0.429729

C 0 -0.121854 -4.071429 -1.127954

C 0 1.353570 -4.455861 0.885567

H 0 0.837779 -7.110768 0.288264

H 0 -0.713627 -6.282262 0.473313

H 0 1.692258 -5.211584 -1.095388

H 0 -0.982783 -3.848010 -0.487954

H 0 0.373313 -3.124071 -1.359244

H 0 -0.506889 -4.485673 -2.065859

H 0 0.527152 -4.281799 1.584699

H 0 2.053950 -5.137040 1.380445

H 0 1.873045 -3.506034 0.724892

C -1 -3.593254 -6.933531 3.872204

C 0 -2.343136 -6.381564 3.188461

S 0 -1.310449 -5.284333 4.238164

C 0 -0.675319 -6.477449 5.459733

H 0 -4.214783 -6.124926 4.263225

H 0 -4.192838 -7.509277 3.158973

H 0 -2.616135 -5.752627 2.335504

H 0 -1.710009 -7.187201 2.800815

H 0 -0.076976 -7.251932 4.972140

H 0 -0.049105 -5.912640 6.152017

H 0 -1.486300 -6.927905 6.032992

C -1 -8.593455 -6.748018 0.713303

C 0 -7.770717 -5.628169 1.311533

C 0 -6.371765 -5.689877 1.354562

C 0 -8.382905 -4.500771 1.877256

C 0 -5.615370 -4.679675 1.945448

C 0 -7.641001 -3.484520 2.479580

C 0 -6.243689 -3.568651 2.522747

O 0 -5.451417 -2.646443 3.122813

H 0 -8.936071 -7.450028 1.483904

H 0 -9.481100 -6.351992 0.210471

H 0 -5.861452 -6.544768 0.916107

H 0 -9.464478 -4.414514 1.838360

H 0 -4.532857 -4.727909 1.966607

H 0 -8.138668 -2.624189 2.917413

H 0 -5.862453 -1.750473 3.230738

C -1 -3.718720 -4.901164 -2.924653

C 0 -3.733948 -3.739796 -1.926494

C 0 -5.151169 -3.308842 -1.562754

C 0 -5.227447 -2.074385 -0.691301

O 0 -6.476630 -1.748293 -0.406485

O 0 -4.246268 -1.443728 -0.302601

H 0 -4.209476 -4.620728 -3.863302

H 0 -3.191459 -2.885445 -2.344302

H 0 -3.200288 -4.019633 -1.012509

H 0 -5.685160 -4.106138 -1.031552

H 0 -5.744568 -3.101017 -2.462237

C -1 2.463009 -1.589066 -6.454948

C 0 2.902201 -1.369248 -5.024593

C 0 1.974727 -1.452186 -3.975860

C 0 4.234480 -1.082528 -4.704631

C 0 2.365539 -1.262458 -2.651925

C 0 4.638739 -0.905756 -3.378005

C 0 3.703696 -1.000642 -2.345736

H 0 1.707494 -0.854078 -6.756048

H 0 3.304577 -1.509666 -7.148028

H 0 0.935149 -1.669641 -4.197378

H 0 4.967872 -1.006610 -5.503594

H 0 1.631191 -1.316693 -1.856688

H 0 5.674610 -0.672504 -3.153118

H 0 3.997329 -0.884511 -1.307202

C -1 2.557948 4.064248 -4.608727

C 0 2.953087 3.737357 -3.167853

C 0 3.749875 2.440333 -3.088097

N 0 4.275536 2.228603 -1.708901

H 0 3.443232 4.220511 -5.234408

H 0 1.972202 3.252482 -5.049288

H 0 2.064314 3.651784 -2.532875

H 0 3.558416 4.556494 -2.756761

H 0 4.609306 2.458808 -3.763747

H 0 3.139470 1.572952 -3.344739

H 0 4.842117 3.039901 -1.432316

H 0 4.937523 1.437964 -1.679463

C -1 -2.421723 0.949091 -5.153677

C 0 -1.570808 0.920199 -3.880704

C 0 -1.559499 -0.485829 -3.268722

C 0 -0.152658 1.439130 -4.143315

H 0 -1.993889 0.294717 -5.923243

H 0 -2.035613 1.597361 -3.155046

H 0 -2.545545 -0.751567 -2.874472

H 0 -1.287716 -1.235176 -4.022287

H 0 -0.844192 -0.560068 -2.445739

H 0 0.372899 0.813109 -4.874230

H 0 -0.181198 2.462482 -4.533222

H 0 0.437539 1.441344 -3.221056

C -1 -8.784474 -2.890615 -3.161129

C 0 -9.103091 -3.944708 -2.102954

S 0 -10.545430 -3.538300 -1.044711

C 0 -9.911780 -2.042517 -0.208175

H 0 -9.659895 -2.671780 -3.778126

H 0 -8.451050 -1.963718 -2.694072

H 0 -8.242827 -4.115384 -1.448105

H 0 -9.363464 -4.902172 -2.565355

H 0 -8.944458 -2.251838 0.250688

H 0 -9.827604 -1.208427 -0.902211

H 0 -10.635509 -1.782579 0.567200

C -1 -11.413168 -0.217085 -4.324311

C 0 -10.670412 0.420352 -3.175527

C 0 -9.274150 0.489061 -3.171403

C 0 -11.348442 0.924628 -2.057731

C 0 -8.566925 1.016757 -2.093190

C 0 -10.660879 1.485958 -0.983528

C 0 -9.264550 1.539058 -0.997488

O 0 -8.611935 2.113609 0.065475

H 0 -10.772813 -0.310986 -5.205790

H 0 -11.757808 -1.223409 -4.056854

H 0 -8.721638 0.106178 -4.024913

H 0 -12.434416 0.883146 -2.029763

H 0 -7.483640 1.025345 -2.101402

H 0 -11.189862 1.897610 -0.130453

H 0 -7.825674 2.638537 -0.296154

C -1 -5.818689 4.554308 -3.597387

C 0 -4.945914 3.723266 -2.641460

C 0 -5.715618 2.880841 -1.624978

O 0 -6.655683 3.442549 -0.970479

O 0 -5.363744 1.682649 -1.447741

H 0 -6.416479 5.283546 -3.052931

H 0 -4.307682 4.401220 -2.057093

H 0 -4.282350 3.049843 -3.191734

C -1 -9.497310 6.950001 -2.623473

C 0 -9.151697 5.619055 -1.985210

C 0 -9.270976 4.481466 -3.001407

O 0 -7.841620 5.777870 -1.463775

H 0 -8.811253 7.148542 -3.453404

H 0 -9.856742 5.414736 -1.159557

H 0 -10.322095 4.307481 -3.254585

H 0 -8.732970 4.727519 -3.921967

H 0 -8.867956 3.548314 -2.609950

H 0 -7.448663 4.901536 -1.256718

N 0 -7.572561 5.007916 6.135646

C 0 -6.547039 4.030815 5.720859

C 0 -5.252038 4.635887 5.186591

O 0 -4.241333 3.935964 5.088267

C 0 -7.242184 3.217196 4.604510

C 0 -8.730210 3.322802 4.957208

C 0 -8.865304 4.761564 5.472052

H 0 -6.268665 3.403126 6.572273

H 0 -6.870235 2.192958 4.540578

H 0 -7.056551 3.696488 3.636424

H 0 -8.980623 2.619265 5.758537

H 0 -9.384511 3.118794 4.106564

H 0 -9.013335 5.463282 4.640073

H 0 -9.695513 4.887653 6.169999

N 0 -5.334109 5.903805 4.732735

C -1 -4.223931 6.582082 4.088835

C 0 -4.642233 7.328619 2.821043

C 0 -5.393287 6.492707 1.770794

C 0 -5.676756 7.350045 0.531439

C 0 -4.651688 5.203689 1.394423

H 0 -3.487700 5.809973 3.861598

H 0 -3.730740 7.744584 2.370030

H 0 -5.263892 8.193393 3.093511

H 0 -6.358879 6.198977 2.206713

H 0 -6.150324 8.299141 0.810265

H 0 -4.739582 7.592055 0.012979

H 0 -6.189716 6.406439 4.917392

H 0 -6.341203 6.843171 -0.172415

H 0 -4.501594 4.546628 2.256847

H 0 -5.222507 4.648672 0.644769

H 0 -3.663088 5.432156 0.973611

C -1 -0.160164 5.153762 5.413030

C 0 -0.862806 3.907745 4.874186

S 0 -0.385141 3.479983 3.150670

C 0 1.254126 2.716768 3.425870

H 0 0.927508 5.030820 5.434587

H 0 -0.389737 6.025859 4.794499

H 0 -1.947158 4.049663 4.846955

H 0 -0.674114 3.033134 5.505634

H 0 1.856477 3.332671 4.098127

H 0 1.156070 1.709526 3.840556

H 0 1.755571 2.660939 2.458305

C -1 -0.794525 9.087960 -2.940487

C 0 -0.312913 7.635911 -3.085409

C 0 -1.377579 6.648351 -2.571904

C 0 1.037951 7.444176 -2.382648

C 0 -1.041478 5.170693 -2.797940

H 0 -0.952800 9.339778 -1.885156

H 0 -0.166845 7.434254 -4.157864

H 0 -2.331255 6.874834 -3.064938

H 0 -1.541345 6.826492 -1.499821

H 0 1.777732 8.163187 -2.751310

H 0 0.934957 7.595669 -1.301197

H 0 1.443405 6.439910 -2.539111

H 0 -0.161919 4.857632 -2.226387

H 0 -1.873691 4.528922 -2.494473

H 0 -0.839191 4.966955 -3.856099

C -1 -8.525092 6.609787 7.776847

C 0 -7.371335 5.740552 7.282930

O 0 -6.294624 5.703854 7.868932

H 0 -9.309410 5.989886 8.225081

H 0 -8.130940 7.281539 8.538247

H 0 -1.742240 9.246189 -3.465223

H 0 -0.060752 9.792795 -3.345501

H 0 1.950985 4.972464 -4.648718

H 0 3.028199 -2.110906 7.077200

H 0 1.474435 0.728051 11.065522

H 0 -1.920534 -8.381422 11.603926

H 0 -2.297040 -4.220835 13.432279

H 0 -3.333930 -7.595032 4.703866

H 0 -8.012895 -7.321388 -0.016091

H 0 -7.978628 -3.245318 -3.816032

H 0 -4.244651 -5.775698 -2.525868

H 0 -2.694438 -5.207023 -3.160366

H 0 -0.199984 -6.845199 -1.124793

H 0 2.013996 -2.580062 -6.581570

H 0 -2.484026 1.960158 -5.570993

H 0 -3.442363 0.607047 -4.951812

H 0 -5.181403 5.082163 -4.315113

H 0 -9.394675 7.762692 -1.897823

H 0 -10.522284 6.956543 -3.010186

H 0 -6.494918 3.907158 -4.163776

H 0 -12.296690 0.364450 -4.607952

H 0 -8.981509 7.188987 6.968657

H 0 -3.750572 7.279141 4.793466

H 0 -0.493500 5.359136 6.436431

O 0 6.830037 1.158413 -1.840702

O 0 -6.443199 0.587987 0.698173

H 0 7.609295 0.734976 -1.436860

H 0 7.056790 2.099643 -1.881237

H 0 -7.334972 1.003080 0.689681

H 0 -5.983386 1.024299 -0.089276

C -1 5.111265 1.406267 8.853287

C 0 4.505802 2.035650 7.591660

C 0 5.580539 2.731544 6.747887

C 0 3.758771 0.987377 6.762163

H 0 4.337159 0.941293 9.473089

H 0 5.837224 0.628160 8.587573

H 0 3.779025 2.797487 7.908263

H 0 6.332871 2.008320 6.409116

H 0 5.144711 3.198658 5.857751

H 0 6.099295 3.509390 7.318730

H 0 3.279044 1.435102 5.885497

H 0 4.448194 0.213985 6.404422

H 0 2.980824 0.493039 7.350245

C -1 -7.139743 -6.830887 9.563961

C 0 -6.771045 -5.889798 8.439612

C 0 -6.348635 -4.584398 8.729084

C 0 -6.863976 -6.270533 7.094920

C 0 -6.064109 -3.683627 7.704467

C 0 -6.558800 -5.375446 6.065038

C 0 -6.162086 -4.071440 6.364355

H 0 -6.367531 -6.822132 10.339309

H 0 -8.083123 -6.528058 10.033636

H 0 -6.233411 -4.285905 9.767048

H 0 -7.185887 -7.280121 6.850727

H 0 -5.768521 -2.669068 7.953564

H 0 -6.644971 -5.686042 5.027661

H 0 -5.925989 -3.375995 5.564639

H 0 -7.265572 -7.855659 9.201523

H 0 5.630003 2.152864 9.464208

H 0 7.378289 -0.609337 2.182000

C -1 6.647166 5.725224 0.043059

C 0 7.163324 4.961802 -1.150998

O 0 6.536806 4.016093 -1.650647

H 0 6.584289 5.039371 0.892612

N 0 8.373724 5.337695 -1.634590

C 0 9.003645 4.691425 -2.772263

C 0 10.086797 3.664889 -2.429164

O 0 10.831739 3.239253 -3.303420

H 0 8.224610 4.176731 -3.339590

H 0 8.846913 6.115803 -1.199778

N 0 10.166402 3.283203 -1.126107

C -1 11.056814 2.193971 -0.766111

C 0 10.467658 0.850327 -1.148895

O 0 9.322987 0.552733 -0.775503

H 0 12.026457 2.380979 -1.222970

H 0 9.393054 3.485632 -0.511463

N 0 11.248020 0.008670 -1.834519

C -1 10.756982 -1.336995 -2.120072

C 0 11.947467 -2.011694 -2.810407

C 0 12.629882 -0.844201 -3.540091

C 0 12.505206 0.319333 -2.546317

H 0 11.637592 -2.816693 -3.479920

H 0 12.624700 -2.437649 -2.062064

H 0 12.083335 -0.602467 -4.457565

H 0 13.669022 -1.045845 -3.809080

H 0 13.348540 0.331231 -1.844771

H 0 12.427810 1.291007 -3.037407

H 0 9.880910 -1.281413 -2.777618

H 0 5.631611 6.068660 -0.168404

H 0 7.267676 6.582001 0.312977

H 0 9.458449 5.437498 -3.427336

H 0 11.181463 2.197382 0.320382

H 0 10.439711 -1.825995 -1.196103

O 0 -5.993036 -0.030823 3.356338

H 0 -6.046739 0.313088 2.437895

H 0 -5.119058 0.238880 3.680204

N 0 -3.215796 0.646956 1.559308

C 0 -3.898939 -0.128525 6.647611

C 0 -3.644262 -1.442339 6.261878

C 0 -3.223384 1.898006 0.837892

C 0 -2.432039 1.754075 -0.507313

C 0 -3.010742 -1.723459 5.050670

C 0 -2.604819 -0.688669 4.197619

C 0 -1.082491 1.183841 -0.234409

C 0 -1.904181 -1.009858 2.863181

C 0 0.063301 1.963087 -0.251857

C 0 1.319935 1.431687 0.121536

C 0 -1.987949 0.212470 1.961605

C 0 1.374416 0.050999 0.557775

C 0 -2.846907 0.633856 4.612157

C 0 -3.495207 0.914942 5.817033

C 0 0.259235 -0.734931 0.583390

C 0 -1.024653 -0.169471 0.267247

O 0 2.614050 -0.403918 0.894706

O 0 2.429908 2.097424 0.136598

H 0 -3.945998 -2.260277 6.902437

H 0 -2.851479 -2.758696 4.776981

H 0 3.167326 0.402457 0.835175

H 0 -2.751625 2.662743 1.459160

H 0 -2.360902 2.741308 -0.971061

H 0 -1.348995 1.029579 2.291016

H 0 -2.557337 1.473296 3.993504

H 0 0.017546 3.005944 -0.549450

H 0 -3.697645 1.950615 6.065674

H 0 0.337781 -1.774775 0.876946

H 0 -3.836503 -0.067429 1.172840

H 0 -3.033902 1.114447 -1.153846

H 0 -4.247451 2.190371 0.624372

H 0 3.486417 2.105422 -0.993462

H 0 -4.406549 0.078730 7.584947

H 0 -6.495413 -0.872515 0.102754

H 0 -1.789426 -0.852543 -0.089507

C 0 -2.409913 -2.304780 2.213368

H 0 -2.198880 -3.152591 2.864992

H 0 -3.483614 -2.278117 2.038511

H 0 -1.900392 -2.487053 1.266134

H 0 -0.837301 -1.140695 3.080060

**Int8R** (-8045.704561)

C -1 7.281283 0.460769 1.969313

C 0 5.795904 0.780057 1.755242

C 0 4.955357 0.393582 2.978922

C 0 5.604113 2.260424 1.401161

H 0 7.882937 0.712186 1.092199

H 0 7.669542 1.025693 2.826871

H 0 5.450574 0.181951 0.897195

H 0 5.267468 0.969410 3.858096

H 0 3.889824 0.581356 2.817394

H 0 5.065270 -0.668789 3.217401

H 0 4.550757 2.497722 1.217950

H 0 5.948485 2.896458 2.227107

H 0 6.181410 2.518397 0.507156

C -1 2.866939 -2.655864 6.140093

C 0 2.199495 -1.760578 5.087209

C 0 2.010775 -2.506340 3.762559

C 0 0.860338 -1.213355 5.594520

H 0 2.234659 -3.523301 6.364999

H 0 3.834980 -3.030128 5.789243

H 0 2.867081 -0.908739 4.900812

H 0 1.314419 -3.344577 3.881760

H 0 1.612422 -1.841091 2.992120

H 0 2.958731 -2.906394 3.386573

H 0 0.134853 -2.023187 5.726619

H 0 0.970830 -0.702853 6.555656

H 0 0.419897 -0.501344 4.889005

C -1 0.465941 0.417129 10.771158

C 0 0.305662 0.354635 9.246479

C 0 -1.084281 -0.156398 8.854974

C 0 0.582888 1.719315 8.603489

H 0 -0.251089 1.124097 11.205477

H 0 0.285453 -0.561949 11.228006

H 0 1.050673 -0.358609 8.861444

H 0 -1.862415 0.518120 9.231527

H 0 -1.204358 -0.209976 7.769839

H 0 -1.277285 -1.153166 9.263308

H 0 -0.152944 2.459621 8.939114

H 0 1.578430 2.094234 8.865672

H 0 0.519247 1.662214 7.511555

C -1 -3.085849 -4.133049 12.675885

C 0 -3.279954 -5.462706 11.923454

O 0 -4.334717 -5.717838 11.350461

C 0 -2.779940 -3.023137 11.655233

H 0 -4.020805 -3.925749 13.201714

H 0 -1.849247 -3.229646 11.117331

H 0 -3.580611 -2.965680 10.913695

H 0 -2.686958 -2.050150 12.146127

N 0 -2.199350 -6.311263 11.895797

C -1 -2.114480 -7.479895 11.011438

C 0 -1.060287 -7.300035 9.910171

C 0 -1.222424 -6.016398 9.127524

C 0 -2.425031 -5.716364 8.472217

C 0 -0.170490 -5.097460 9.051441

C 0 -2.560865 -4.539258 7.740717

C 0 -0.306283 -3.910211 8.327027

C 0 -1.501050 -3.632654 7.664601

H 0 -3.113557 -7.595274 10.587551

H 0 -1.120365 -8.169980 9.242524

H 0 -0.057990 -7.327161 10.355505

H 0 -3.268954 -6.395910 8.539353

H 0 -1.338199 -5.974817 12.301807

H 0 0.765424 -5.316092 9.560409

H 0 -3.494093 -4.339872 7.227210

H 0 0.518440 -3.205779 8.276721

H 0 -1.613329 -2.718070 7.096789

C -1 0.152990 -6.400700 -0.188137

C 0 0.799349 -5.027608 -0.419917

C 0 -0.193468 -4.055318 -1.066869

C 0 1.350551 -4.450299 0.888996

H 0 0.866309 -7.107727 0.248521

H 0 -0.693784 -6.314161 0.503230

H 0 1.640872 -5.161742 -1.115157

H 0 -1.046432 -3.874653 -0.403497

H 0 0.273887 -3.087936 -1.276006

H 0 -0.587204 -4.447810 -2.010354

H 0 0.546672 -4.306245 1.620396

H 0 2.083169 -5.123966 1.346352

H 0 1.842720 -3.485527 0.726322

C -1 -3.593268 -6.933488 3.872188

C 0 -2.339561 -6.387993 3.190389

S 0 -1.307179 -5.292371 4.241767

C 0 -0.709664 -6.480086 5.487157

H 0 -4.212711 -6.122029 4.260251

H 0 -4.193453 -7.509208 3.159306

H 0 -2.607694 -5.760337 2.334693

H 0 -1.709314 -7.197890 2.806509

H 0 -0.122562 -7.275116 5.019111

H 0 -0.079653 -5.917414 6.177622

H 0 -1.535775 -6.903768 6.059066

C -1 -8.593472 -6.748030 0.713308

C 0 -7.780426 -5.622607 1.314561

C 0 -6.383575 -5.692487 1.396167

C 0 -8.400867 -4.484316 1.848806

C 0 -5.638134 -4.681237 1.998613

C 0 -7.669588 -3.466886 2.462175

C 0 -6.274733 -3.561020 2.549721

O 0 -5.493564 -2.649405 3.176447

H 0 -8.961940 -7.433716 1.486650

H 0 -9.465028 -6.357052 0.179274

H 0 -5.867186 -6.555542 0.981285

H 0 -9.480427 -4.392361 1.778189

H 0 -4.557250 -4.735956 2.053925

H 0 -8.173893 -2.599973 2.878727

H 0 -5.864810 -1.728016 3.231761

C -1 -3.718700 -4.901128 -2.924647

C 0 -3.753573 -3.791107 -1.872409

C 0 -5.175930 -3.337920 -1.560529

C 0 -5.259490 -2.088960 -0.711100

O 0 -6.510952 -1.773219 -0.424041

O 0 -4.282264 -1.437876 -0.349276

H 0 -4.148252 -4.558161 -3.872715

H 0 -3.165438 -2.932807 -2.212808

H 0 -3.277020 -4.131816 -0.947102

H 0 -5.738473 -4.121516 -1.039371

H 0 -5.735458 -3.130530 -2.482010

C -1 2.463011 -1.589078 -6.454967

C 0 2.893107 -1.363417 -5.022619

C 0 1.961849 -1.430719 -3.976103

C 0 4.232681 -1.114585 -4.698160

C 0 2.356981 -1.273474 -2.647833

C 0 4.639852 -0.968594 -3.369516

C 0 3.701655 -1.054733 -2.338693

H 0 1.501394 -1.109579 -6.662925

H 0 3.200454 -1.197062 -7.160665

H 0 0.916510 -1.615198 -4.202210

H 0 4.968475 -1.046027 -5.495475

H 0 1.620243 -1.326199 -1.853990

H 0 5.682517 -0.769207 -3.142919

H 0 4.001607 -0.969015 -1.299378

C -1 2.557949 4.064228 -4.608678

C 0 2.989252 3.697171 -3.189027

C 0 3.832269 2.427553 -3.171375

N 0 4.349711 2.163065 -1.795810

H 0 3.425411 4.265467 -5.246335

H 0 1.982006 3.254648 -5.066011

H 0 2.115581 3.555625 -2.543599

H 0 3.573213 4.520189 -2.755406

H 0 4.700961 2.513688 -3.829135

H 0 3.258739 1.551460 -3.478772

H 0 4.865663 2.986805 -1.460952

H 0 5.055107 1.405763 -1.793642

C -1 -2.421699 0.949080 -5.153687

C 0 -1.625304 0.990773 -3.842988

C 0 -1.623061 -0.383282 -3.160226

C 0 -0.200808 1.507582 -4.078528

H 0 -1.953533 0.261843 -5.869418

H 0 -2.124727 1.697877 -3.168254

H 0 -2.620497 -0.647805 -2.795497

H 0 -1.303195 -1.165539 -3.859654

H 0 -0.940253 -0.403266 -2.305869

H 0 0.351684 0.851682 -4.761796

H 0 -0.218162 2.511985 -4.515054

H 0 0.359102 1.554582 -3.138587

C -1 -8.784388 -2.890558 -3.161080

C 0 -9.122611 -3.932284 -2.097064

S 0 -10.577015 -3.508099 -1.062763

C 0 -9.945423 -2.010745 -0.227170

H 0 -9.651834 -2.669084 -3.788006

H 0 -8.446301 -1.962865 -2.698658

H 0 -8.272067 -4.100431 -1.429308

H 0 -9.382036 -4.892810 -2.553715

H 0 -8.986292 -2.223962 0.246662

H 0 -9.844877 -1.182211 -0.925656

H 0 -10.678733 -1.740521 0.535527

C -1 -11.413151 -0.217079 -4.324283

C 0 -10.679106 0.436055 -3.180540

C 0 -9.282980 0.503468 -3.168627

C 0 -11.364887 0.954227 -2.074280

C 0 -8.582911 1.043813 -2.092646

C 0 -10.684320 1.527611 -1.002232

C 0 -9.288170 1.579400 -1.008127

O 0 -8.643279 2.162017 0.054314

H 0 -10.779010 -0.290863 -5.212361

H 0 -11.729540 -1.233024 -4.057494

H 0 -8.725436 0.109548 -4.013778

H 0 -12.451043 0.913533 -2.053784

H 0 -7.499693 1.052079 -2.094755

H 0 -11.218130 1.949592 -0.157273

H 0 -7.843914 2.670855 -0.300577

C -1 -5.818666 4.554279 -3.597391

C 0 -4.942847 3.737078 -2.631927

C 0 -5.714924 2.891757 -1.619311

O 0 -6.652288 3.455764 -0.961732

O 0 -5.373444 1.689595 -1.454942

H 0 -6.416413 5.289888 -3.061915

H 0 -4.313756 4.424073 -2.048604

H 0 -4.271917 3.065984 -3.175983

C -1 -9.497291 6.949971 -2.623454

C 0 -9.133737 5.621015 -1.991980

C 0 -9.248463 4.487473 -3.013382

O 0 -7.821285 5.793400 -1.480610

H 0 -8.815375 7.159922 -3.454009

H 0 -9.829757 5.405403 -1.161847

H 0 -10.300016 4.297815 -3.253015

H 0 -8.727240 4.747697 -3.939729

H 0 -8.825379 3.558950 -2.633154

H 0 -7.426334 4.920902 -1.258746

N 0 -7.514803 4.969733 6.209321

C 0 -6.450873 4.019036 5.829595

C 0 -5.177189 4.656169 5.282438

O 0 -4.131528 4.005955 5.238672

C 0 -7.110584 3.148171 4.735653

C 0 -8.600585 3.196636 5.092120

C 0 -8.799608 4.642457 5.565491

H 0 -6.153056 3.427378 6.699969

H 0 -6.692529 2.140929 4.689931

H 0 -6.950012 3.614741 3.756848

H 0 -8.816081 2.505935 5.914519

H 0 -9.248476 2.938767 4.251226

H 0 -8.987914 5.311227 4.714661

H 0 -9.629465 4.749798 6.267315

N 0 -5.314545 5.889453 4.750098

C -1 -4.223933 6.582040 4.088825

C 0 -4.689804 7.362852 2.860077

C 0 -5.461057 6.549882 1.806356

C 0 -5.801001 7.442529 0.607098

C 0 -4.707346 5.289484 1.364151

H 0 -3.496124 5.817143 3.813427

H 0 -3.798778 7.806013 2.394459

H 0 -5.313469 8.209903 3.180089

H 0 -6.406170 6.221852 2.262275

H 0 -6.296758 8.365227 0.932460

H 0 -4.884587 7.733240 0.076694

H 0 -6.189448 6.367794 4.906152

H 0 -6.463297 6.938251 -0.100994

H 0 -4.524987 4.606165 2.199359

H 0 -5.284990 4.747964 0.610105

H 0 -3.733441 5.550959 0.928287

C -1 -0.160219 5.153709 5.413047

C 0 -0.843774 3.903226 4.862646

S 0 -0.311876 3.450268 3.162156

C 0 1.288348 2.639601 3.512021

H 0 0.926057 5.030508 5.476433

H 0 -0.366382 6.019906 4.778262

H 0 -1.926411 4.045498 4.804530

H 0 -0.676590 3.034398 5.508154

H 0 1.940655 3.292610 4.097541

H 0 1.141195 1.696488 4.045781

H 0 1.768092 2.440903 2.551905

C -1 -0.794542 9.087912 -2.940478

C 0 -0.312401 7.636001 -3.086938

C 0 -1.377696 6.645691 -2.579331

C 0 1.036509 7.443796 -2.380344

C 0 -1.039566 5.169619 -2.813331

H 0 -0.956029 9.337660 -1.885133

H 0 -0.162797 7.437093 -4.159454

H 0 -2.330692 6.873656 -3.072763

H 0 -1.543349 6.818285 -1.506744

H 0 1.777081 8.163821 -2.745453

H 0 0.929991 7.593370 -1.298992

H 0 1.443160 6.439851 -2.537209

H 0 -0.158339 4.856022 -2.244078

H 0 -1.869953 4.522487 -2.515912

H 0 -0.835882 4.973923 -3.872824

C -1 -8.525060 6.609756 7.776833

C 0 -7.337754 5.766463 7.316381

O 0 -6.256852 5.805827 7.894900

H 0 -9.279104 5.979892 8.261616

H 0 -8.155004 7.334595 8.500695

H 0 -1.740638 9.246961 -3.467690

H 0 -0.059400 9.793255 -3.342046

H 0 1.928135 4.957632 -4.603166

H 0 3.034283 -2.112500 7.076584

H 0 1.472028 0.744852 11.056426

H 0 -1.894151 -8.376943 11.601613

H 0 -2.283756 -4.210640 13.419310

H 0 -3.338130 -7.594057 4.705880

H 0 -7.996526 -7.338324 0.011059

H 0 -7.975890 -3.258206 -3.805773

H 0 -4.293667 -5.775733 -2.600086

H 0 -2.693732 -5.231508 -3.121318

H 0 -0.221912 -6.829707 -1.123736

H 0 2.342576 -2.658833 -6.662362

H 0 -2.472347 1.937991 -5.622004

H 0 -3.446886 0.606776 -4.980027

H 0 -5.183041 5.073349 -4.323118

H 0 -9.403303 7.761239 -1.895108

H 0 -10.522949 6.945365 -3.008316

H 0 -6.494329 3.898442 -4.154297

H 0 -12.314193 0.341795 -4.598364

H 0 -9.011153 7.127992 6.944915

H 0 -3.722252 7.259374 4.793759

H 0 -0.532511 5.369350 6.420719

O 0 6.902356 1.165545 -1.951681

O 0 -6.514855 0.569492 0.650618

H 0 7.657551 0.731085 -1.512632

H 0 7.117760 2.110381 -1.926899

H 0 -7.394497 1.008101 0.643395

H 0 -6.026014 1.013582 -0.112397

C -1 5.111257 1.406267 8.853282

C 0 4.496668 2.036803 7.596763

C 0 5.565028 2.735873 6.747361

C 0 3.746132 0.989932 6.768696

H 0 4.341821 0.937745 9.476098

H 0 5.837475 0.630491 8.581524

H 0 3.771216 2.796921 7.919950

H 0 6.314786 2.013846 6.400120

H 0 5.121993 3.206471 5.862438

H 0 6.088212 3.511828 7.316703

H 0 3.262545 1.440200 5.895362

H 0 4.433875 0.216920 6.406643

H 0 2.970144 0.494333 7.358080

C -1 -7.139722 -6.830816 9.563946

C 0 -6.794607 -5.883556 8.437677

C 0 -6.436402 -4.558196 8.722575

C 0 -6.847207 -6.278776 7.095107

C 0 -6.176748 -3.652812 7.695591

C 0 -6.566444 -5.378435 6.062827

C 0 -6.235477 -4.055176 6.357256

H 0 -6.373422 -6.793409 10.344486

H 0 -8.096076 -6.557543 10.025534

H 0 -6.354244 -4.246233 9.759820

H 0 -7.118621 -7.303960 6.854477

H 0 -5.929555 -2.624187 7.940664

H 0 -6.620691 -5.700528 5.026800

H 0 -6.017783 -3.356474 5.555292

H 0 -7.228254 -7.860870 9.205753

H 0 5.631280 2.152771 9.463059

H 0 7.428490 -0.602741 2.183838

C -1 6.647147 5.725223 0.043056

C 0 7.163641 4.952238 -1.144379

O 0 6.540864 3.996029 -1.630005

H 0 6.569297 5.042979 0.894361

N 0 8.367442 5.331450 -1.639633

C 0 8.993159 4.680123 -2.777272

C 0 10.071819 3.648370 -2.434828

O 0 10.800176 3.204701 -3.313864

H 0 8.212465 4.168161 -3.344616

H 0 8.835831 6.120034 -1.218565

N 0 10.166584 3.282386 -1.128864

C -1 11.056788 2.193966 -0.766114

C 0 10.467856 0.849456 -1.146613

O 0 9.324099 0.551236 -0.768031

H 0 12.027448 2.378787 -1.222014

H 0 9.410670 3.508929 -0.501530

N 0 11.246031 0.009537 -1.835417

C -1 10.756972 -1.336989 -2.120082

C 0 11.948950 -2.011265 -2.808165

C 0 12.630037 -0.844089 -3.539449

C 0 12.502934 0.321115 -2.548028

H 0 11.640764 -2.817922 -3.476409

H 0 12.626014 -2.434825 -2.058369

H 0 12.083935 -0.604847 -4.457818

H 0 13.669686 -1.044616 -3.807123

H 0 13.345776 0.336072 -1.846115

H 0 12.422991 1.291472 -3.041115

H 0 9.882100 -1.282709 -2.779540

H 0 5.637292 6.080005 -0.177544

H 0 7.274927 6.575825 0.315588

H 0 9.450661 5.424192 -3.432631

H 0 11.180222 2.199949 0.320453

H 0 10.438737 -1.824996 -1.196078

O 0 -5.913345 -0.020219 3.283848

H 0 -6.084153 0.285479 2.366319

H 0 -4.979747 0.212687 3.424560

N 0 -3.316639 0.618220 1.674138

C 0 -3.872605 -0.095125 6.669498

C 0 -3.734771 -1.416321 6.252542

C 0 -3.333439 1.906345 1.018591

C 0 -2.536791 1.875964 -0.332807

C 0 -3.115021 -1.722557 5.040171

C 0 -2.605330 -0.710163 4.217032

C 0 -1.203060 1.263832 -0.103015

C 0 -1.925990 -1.050575 2.884086

C 0 -0.034231 1.959145 -0.260679

C 0 1.227733 1.375719 0.057214

C 0 -1.986407 0.160462 1.937368

C 0 1.252093 -0.000191 0.552678

C 0 -2.731943 0.617641 4.665149

C 0 -3.365826 0.927472 5.868997

C 0 0.115451 -0.709988 0.713907

C 0 -1.209296 -0.092273 0.506685

O 0 2.494479 -0.504654 0.797380

O 0 2.342930 1.981331 -0.046860

H 0 -4.118547 -2.220358 6.866215

H 0 -3.050875 -2.760049 4.739115

H 0 3.087518 0.260188 0.665727

H 0 -2.879956 2.646444 1.684015

H 0 -2.443870 2.889836 -0.733204

H 0 -1.427344 0.980580 2.393136

H 0 -2.367667 1.440256 4.063944

H 0 -0.039921 2.983389 -0.619328

H 0 -3.487734 1.969450 6.143274

H 0 0.167781 -1.739368 1.045892

H 0 -3.854636 -0.071711 1.146358

H 0 -3.137215 1.285011 -1.028880

H 0 -4.359989 2.204607 0.814133

H 0 3.561283 1.968295 -1.129182

H 0 -4.371463 0.135169 7.606403

H 0 -6.542384 -0.886088 0.066229

H 0 -1.884235 -0.765001 -0.030490

C 0 -2.456818 -2.337685 2.239730

H 0 -2.247190 -3.195496 2.879441

H 0 -3.533239 -2.294155 2.083298

H 0 -1.973879 -2.524414 1.277912

H 0 -0.860161 -1.203876 3.099763

**Int9R** (-8045.697735)

C -1 7.281498 0.460668 1.968964

C 0 5.783478 0.651125 1.699778

C 0 4.940473 0.004817 2.804853

C 0 5.434132 2.136357 1.546971

H 0 7.894205 0.862525 1.156158

H 0 7.568469 0.961766 2.902185

H 0 5.546875 0.158307 0.746094

H 0 5.191508 0.434968 3.779919

H 0 3.871185 0.162775 2.640096

H 0 5.115532 -1.074498 2.864601

H 0 4.365717 2.265023 1.346486

H 0 5.670653 2.687631 2.465972

H 0 5.999553 2.586677 0.723540

C -1 2.866910 -2.655468 6.140462

C 0 2.050342 -1.522082 5.506760

C 0 1.127662 -2.051395 4.404920

C 0 1.263897 -0.746766 6.569917

H 0 2.207284 -3.404409 6.591907

H 0 3.484502 -3.164660 5.392618

H 0 2.754654 -0.821341 5.035844

H 0 0.404823 -2.770311 4.806304

H 0 0.559475 -1.235463 3.950717

H 0 1.696180 -2.539129 3.608367

H 0 0.582685 -1.402765 7.121513

H 0 1.937601 -0.277894 7.294305

H 0 0.645472 0.036459 6.121138

C -1 0.466508 0.417653 10.770298

C 0 1.198975 1.385245 9.835100

C 0 0.250952 1.968168 8.780534

C 0 1.892645 2.499701 10.626252

H 0 -0.329791 0.934520 11.319396

H 0 0.004259 -0.401113 10.207871

H 0 1.975292 0.810688 9.312175

H 0 -0.524927 2.582273 9.253226

H 0 0.791044 2.607959 8.072653

H 0 -0.251104 1.183096 8.209204

H 0 1.157894 3.091681 11.185130

H 0 2.611147 2.093517 11.346568

H 0 2.429643 3.183616 9.960721

C -1 -3.085948 -4.133031 12.675987

C 0 -3.259664 -5.560373 12.123721

O 0 -4.372289 -6.056612 11.961498

C 0 -3.031292 -3.122291 11.516978

H 0 -3.945817 -3.939410 13.319908

H 0 -2.185266 -3.328484 10.852813

H 0 -3.946232 -3.173768 10.920210

H 0 -2.927208 -2.100746 11.893907

N 0 -2.114067 -6.220635 11.770539

C -1 -2.114164 -7.480116 11.011723

C 0 -0.890234 -7.568202 10.093071

C 0 -0.779783 -6.396548 9.145207

C 0 -1.649090 -6.273315 8.053524

C 0 0.162570 -5.386638 9.370628

C 0 -1.564513 -5.178484 7.195948

C 0 0.244313 -4.281299 8.522190

C 0 -0.612623 -4.182768 7.426885

H 0 -3.042616 -7.504979 10.434371

H 0 -0.959855 -8.509047 9.535603

H 0 0.019053 -7.634910 10.703250

H 0 -2.404362 -7.034373 7.877867

H 0 -1.240554 -5.719356 11.839093

H 0 0.848506 -5.474231 10.210523

H 0 -2.239329 -5.092990 6.351299

H 0 0.979800 -3.504814 8.709052

H 0 -0.546085 -3.332833 6.760112

C -1 0.152987 -6.400941 -0.188149

C 0 0.926724 -5.359434 -1.007421

C 0 -0.032640 -4.392175 -1.710407

C 0 1.921302 -4.587640 -0.130317

H 0 0.831959 -7.111672 0.296802

H 0 -0.433718 -5.905908 0.596913

H 0 1.496575 -5.895543 -1.780595

H 0 -0.609716 -3.817077 -0.976735

H 0 0.513712 -3.681824 -2.337786

H 0 -0.744688 -4.924404 -2.348267

H 0 1.401666 -4.008651 0.637965

H 0 2.617477 -5.266704 0.374968

H 0 2.509413 -3.878689 -0.720607

C -1 -3.593924 -6.933382 3.872328

C 0 -3.032783 -5.988624 2.809421

S 0 -1.716949 -4.839907 3.388727

C 0 -0.400143 -6.008813 3.863012

H 0 -4.033653 -6.374678 4.703577

H 0 -4.377828 -7.559659 3.432838

H 0 -3.818426 -5.326084 2.432599

H 0 -2.638229 -6.545852 1.952964

H 0 -0.139846 -6.663300 3.027088

H 0 0.470061 -5.402053 4.122102

H 0 -0.680832 -6.599766 4.737064

C -1 -8.594208 -6.748315 0.712880

C 0 -8.078084 -5.442701 1.277969

C 0 -6.910078 -4.837077 0.800778

C 0 -8.800380 -4.754600 2.263758

C 0 -6.502391 -3.573597 1.239594

C 0 -8.414275 -3.493454 2.707295

C 0 -7.284632 -2.871366 2.161769

O 0 -7.010981 -1.595164 2.567324

H 0 -8.992401 -7.398952 1.498322

H 0 -9.406471 -6.556292 0.001650

H 0 -6.305762 -5.351291 0.056664

H 0 -9.705993 -5.200705 2.666218

H 0 -5.597813 -3.122291 0.852648

H 0 -9.003402 -2.951288 3.439401

H 0 -6.508084 -1.108042 1.890778

C -1 -3.718768 -4.900948 -2.924955

C 0 -3.785595 -3.790995 -1.876534

C 0 -5.009542 -2.911060 -2.091710

C 0 -5.173072 -1.750011 -1.120771

O 0 -4.238828 -1.394703 -0.370223

O 0 -6.332644 -1.188823 -1.166619

H 0 -3.629672 -4.483079 -3.934272

H 0 -2.881861 -3.174710 -1.910871

H 0 -3.815151 -4.224817 -0.869033

H 0 -5.930591 -3.502521 -2.042597

H 0 -4.989825 -2.473268 -3.098992

C -1 2.463052 -1.588926 -6.455131

C 0 2.812317 -1.407855 -4.996134

C 0 1.820578 -1.498905 -4.009339

C 0 4.131697 -1.176598 -4.587930

C 0 2.135298 -1.373397 -2.656479

C 0 4.458957 -1.066694 -3.234231

C 0 3.460448 -1.170129 -2.263910

H 0 3.223293 -1.149243 -7.106711

H 0 2.386128 -2.652665 -6.709302

H 0 0.791262 -1.674640 -4.305141

H 0 4.913785 -1.091924 -5.338242

H 0 1.350036 -1.443311 -1.911640

H 0 5.487252 -0.880216 -2.941361

H 0 3.696350 -1.084404 -1.209812

C -1 2.557168 4.063993 -4.608790

C 0 2.833327 3.647644 -3.155802

C 0 3.591687 2.324019 -3.070176

N 0 4.057562 2.035521 -1.673481

H 0 3.491015 4.196530 -5.164808

H 0 1.957012 3.309092 -5.125554

H 0 1.888449 3.565944 -2.605781

H 0 3.426281 4.428629 -2.661227

H 0 4.489306 2.349112 -3.692779

H 0 2.982081 1.477060 -3.389484

H 0 4.500290 2.871554 -1.276234

H 0 4.819067 1.333503 -1.689577

C -1 -2.422069 0.948771 -5.153381

C 0 -1.749178 0.910507 -3.776435

C 0 -1.823462 -0.501380 -3.181908

C 0 -0.307763 1.432594 -3.846395

H 0 -1.893813 0.293775 -5.857712

H 0 -2.309562 1.579771 -3.109246

H 0 -2.862502 -0.789462 -3.003163

H 0 -1.388107 -1.237201 -3.868803

H 0 -1.286429 -0.570505 -2.231919

H 0 0.292318 0.837922 -4.544865

H 0 -0.284582 2.474739 -4.187165

H 0 0.180199 1.379588 -2.867821

C -1 -8.784218 -2.890275 -3.160702

C 0 -9.196641 -4.006108 -2.206446

S 0 -10.543811 -3.544115 -1.051647

C 0 -9.758750 -2.107857 -0.237598

H 0 -9.643201 -2.514758 -3.722535

H 0 -8.328075 -2.062513 -2.615330

H 0 -8.341552 -4.340628 -1.609315

H 0 -9.581794 -4.873702 -2.752074

H 0 -8.714766 -2.331244 -0.018999

H 0 -9.826177 -1.218588 -0.861259

H 0 -10.286535 -1.936048 0.701655

C -1 -11.412886 -0.217101 -4.324232

C 0 -10.683526 0.424645 -3.171219

C 0 -9.298787 0.607362 -3.206359

C 0 -11.365735 0.825426 -2.014402

C 0 -8.608067 1.157134 -2.129416

C 0 -10.692655 1.392174 -0.934496

C 0 -9.307313 1.558251 -0.989038

O 0 -8.666166 2.098959 0.107004

H 0 -10.769550 -0.296962 -5.204670

H 0 -11.745768 -1.228397 -4.061766

H 0 -8.734171 0.299007 -4.081626

H 0 -12.442769 0.690975 -1.957090

H 0 -7.532609 1.238243 -2.184803

H 0 -11.223076 1.708817 -0.042882

H 0 -7.932502 2.666296 -0.227534

C -1 -5.818508 4.554136 -3.597005

C 0 -4.996796 3.553318 -2.797129

C 0 -5.717499 3.011519 -1.583953

O 0 -6.725785 3.542538 -1.102758

O 0 -5.174043 1.923316 -1.099071

H 0 -6.109623 5.418578 -2.996128

H 0 -4.057886 3.998375 -2.444684

H 0 -4.717578 2.696252 -3.416174

C -1 -9.497373 6.949808 -2.623506

C 0 -9.119210 6.354143 -1.264673

C 0 -9.837317 5.031274 -1.011803

O 0 -7.700120 6.216854 -1.159885

H 0 -9.245284 6.249227 -3.427624

H 0 -9.413734 7.070762 -0.483484

H 0 -10.923554 5.166755 -1.019695

H 0 -9.589844 4.303073 -1.791899

H 0 -9.551381 4.599164 -0.049518

H 0 -7.460547 5.281896 -1.278078

N 0 -7.208941 4.889081 6.551498

C 0 -5.977370 4.087383 6.397224

C 0 -4.830520 4.769335 5.653145

O 0 -3.674915 4.384576 5.829426

C 0 -6.451452 2.826549 5.643808

C 0 -7.918388 2.687297 6.067251

C 0 -8.405228 4.141178 6.121869

H 0 -5.575946 3.841779 7.384815

H 0 -5.834914 1.958429 5.881703

H 0 -6.384762 2.996098 4.563633

H 0 -7.982234 2.239200 7.064684

H 0 -8.505797 2.073817 5.380503

H 0 -8.737833 4.481805 5.132419

H 0 -9.231611 4.284604 6.820757

N 0 -5.189226 5.713689 4.749764

C -1 -4.224613 6.582383 4.088864

C 0 -4.462705 6.715977 2.581788

C 0 -3.926813 5.551691 1.734894

C 0 -4.448285 5.651108 0.298490

C 0 -2.393081 5.524363 1.740673

H 0 -3.238915 6.169795 4.307606

H 0 -3.989154 7.645835 2.238513

H 0 -5.538140 6.840386 2.399660

H 0 -4.294862 4.614739 2.176373

H 0 -5.539826 5.667207 0.247469

H 0 -4.081862 6.566872 -0.181643

H 0 -6.149559 6.025476 4.799273

H 0 -4.089714 4.804982 -0.294912

H 0 -1.978694 5.400105 2.743948

H 0 -2.005626 4.704821 1.129537

H 0 -1.993957 6.459730 1.328800

C -1 -0.159562 5.153625 5.413312

C 0 -0.692337 3.823341 4.878951

S 0 0.081875 3.239646 3.315564

C 0 1.576549 2.406944 3.951878

H 0 0.901262 5.091393 5.680079

H 0 -0.274358 5.947190 4.669389

H 0 -1.760006 3.911098 4.671632

H 0 -0.593414 3.022259 5.618189

H 0 2.231650 3.102204 4.483737

H 0 1.303311 1.581018 4.612233

H 0 2.112543 2.007537 3.087498

C -1 -0.794156 9.088031 -2.940156

C 0 -0.415565 7.598347 -2.938793

C 0 -1.368834 6.790903 -2.038147

C 0 1.052934 7.417621 -2.531419

C 0 -1.155034 5.274094 -2.067497

H 0 -0.695149 9.513332 -1.934288

H 0 -0.529924 7.219291 -3.965804

H 0 -2.401636 7.008074 -2.337893

H 0 -1.272245 7.150720 -1.004636

H 0 1.715501 8.003096 -3.178115

H 0 1.210834 7.754335 -1.499348

H 0 1.368696 6.371518 -2.590762

H 0 -0.177828 4.989105 -1.663548

H 0 -1.912585 4.767001 -1.463098

H 0 -1.223096 4.880596 -3.088612

C -1 -8.525072 6.609738 7.776866

C 0 -7.190174 5.961468 7.415913

O 0 -6.129277 6.376310 7.870187

H 0 -9.090531 5.965556 8.459156

H 0 -8.310075 7.551721 8.279535

H 0 -1.830235 9.233641 -3.262105

H 0 -0.146821 9.662509 -3.610854

H 0 2.006838 5.007733 -4.637729

H 0 3.532225 -2.278578 6.926362

H 0 1.149581 -0.019784 11.506893

H 0 -2.132358 -8.336876 11.696507

H 0 -2.176610 -4.063286 13.285082

H 0 -2.821170 -7.597393 4.271206

H 0 -7.807037 -7.295252 0.185091

H 0 -8.038696 -3.262469 -3.874531

H 0 -4.625907 -5.516242 -2.905351

H 0 -2.864870 -5.566364 -2.761744

H 0 -0.541009 -6.969528 -0.815857

H 0 1.499291 -1.129274 -6.694199

H 0 -2.417601 1.961287 -5.573512

H 0 -3.460033 0.608843 -5.083113

H 0 -5.246608 4.907677 -4.459596

H 0 -8.947115 7.879104 -2.796667

H 0 -10.570697 7.163401 -2.676652

H 0 -6.734194 4.084970 -3.968311

H 0 -12.303753 0.354244 -4.607492

H 0 -9.149354 6.791119 6.897055

H 0 -4.267821 7.573433 4.559557

H 0 -0.724609 5.434854 6.307970

O 0 6.702146 1.149395 -1.860848

O 0 -6.363561 0.557281 0.592699

H 0 7.493657 0.712927 -1.497907

H 0 6.856484 2.097147 -1.722131

H 0 -7.273618 0.896579 0.705843

H 0 -6.411753 -0.213385 -0.133530

C -1 5.110195 1.406289 8.853716

C 0 4.303614 1.971413 7.676152

C 0 4.486039 3.488064 7.553958

C 0 4.668781 1.267282 6.365522

H 0 4.949488 0.328373 8.960911

H 0 6.183840 1.571190 8.703385

H 0 3.241189 1.778662 7.870071

H 0 5.533408 3.739202 7.344769

H 0 3.875532 3.897014 6.741133

H 0 4.199832 4.000261 8.478444

H 0 4.060097 1.637907 5.534560

H 0 5.722891 1.437893 6.111993

H 0 4.513127 0.186027 6.434144

C -1 -7.140083 -6.831084 9.564061

C 0 -6.473469 -5.961192 8.518337

C 0 -5.379999 -5.153914 8.859937

C 0 -6.944816 -5.914394 7.198598

C 0 -4.788500 -4.313414 7.915376

C 0 -6.355597 -5.075644 6.249945

C 0 -5.273462 -4.266535 6.607434

H 0 -6.484460 -6.973493 10.426851

H 0 -8.067345 -6.368837 9.923456

H 0 -5.003012 -5.191170 9.876811

H 0 -7.789465 -6.536755 6.912039

H 0 -3.946567 -3.691594 8.201750

H 0 -6.748757 -5.040489 5.237286

H 0 -4.822227 -3.597095 5.880985

H 0 -7.405778 -7.812630 9.157245

H 0 4.824927 1.885137 9.795557

H 0 7.531611 -0.600312 2.074369

C -1 6.647824 5.725831 0.042994

C 0 7.017977 4.855374 -1.130120

O 0 6.299716 3.914770 -1.499058

H 0 6.618814 5.100965 0.940751

N 0 8.200373 5.134077 -1.732803

C 0 8.741496 4.361306 -2.835890

C 0 9.931740 3.472105 -2.471282

O 0 10.630059 2.980273 -3.347801

H 0 7.945832 3.711669 -3.209438

H 0 8.735798 5.920174 -1.394637

N 0 10.150171 3.268839 -1.140140

C -1 11.057080 2.194073 -0.766076

C 0 10.462159 0.853856 -1.160475

O 0 9.287484 0.593522 -0.859690

H 0 12.028647 2.381838 -1.218072

H 0 9.387749 3.464268 -0.508339

N 0 11.264885 -0.010251 -1.790222

C -1 10.756967 -1.337001 -2.120040

C 0 11.976011 -2.046406 -2.717716

C 0 12.732802 -0.901078 -3.407478

C 0 12.560887 0.278414 -2.438697

H 0 11.695977 -2.850976 -3.400749

H 0 12.588383 -2.479134 -1.919081

H 0 12.258519 -0.660390 -4.364446

H 0 13.784286 -1.125888 -3.598862

H 0 13.365293 0.301107 -1.693572

H 0 12.515206 1.239610 -2.953888

H 0 9.938563 -1.252931 -2.846016

H 0 5.638776 6.117017 -0.107878

H 0 7.335451 6.558367 0.204637

H 0 9.053138 5.015982 -3.653041

H 0 11.175320 2.206679 0.320768

H 0 10.352666 -1.818799 -1.226962

O 0 -5.501863 0.235559 -3.461912

H 0 -5.034035 0.658218 -2.726692

H 0 -6.080768 -0.373118 -2.967822

H 0 -5.709634 1.439773 -0.300662

H 0 3.305318 1.682067 -1.017548

N 0 -3.263308 0.740959 1.934792

C 0 -2.766271 0.147002 7.378278

C 0 -2.512921 -1.188598 7.058498

C 0 -2.984622 1.916290 1.150549

C 0 -2.281382 1.539015 -0.195387

C 0 -2.405272 -1.579649 5.724493

C 0 -2.545661 -0.651168 4.682247

C 0 -1.083350 0.727072 0.131169

C 0 -2.412384 -1.157938 3.247089

C 0 0.186100 1.203284 -0.058333

C 0 1.329701 0.453829 0.341417

C 0 -2.100622 -0.017693 2.264118

C 0 1.110411 -0.854759 0.958914

C 0 -2.812075 0.683109 5.018000

C 0 -2.916682 1.082877 6.353881

C 0 -0.135856 -1.344462 1.159946

C 0 -1.337810 -0.537956 0.865115

O 0 2.244226 -1.539035 1.267104

O 0 2.538354 0.825819 0.186889

H 0 -2.386000 -1.924997 7.846363

H 0 -2.212526 -2.619477 5.476564

H 0 2.967675 -0.928438 1.028308

H 0 -2.325940 2.573040 1.724532

H 0 -2.014707 2.442105 -0.752861

H 0 -1.364215 0.652379 2.719023

H 0 -2.961679 1.414875 4.234361

H 0 0.348153 2.173761 -0.518570

H 0 -3.123200 2.126960 6.572978

H 0 -0.257981 -2.339714 1.576883

H 0 -3.985291 0.171134 1.496173

H 0 -2.997176 0.956724 -0.776877

H 0 -3.911188 2.448744 0.937103

H 0 -2.847747 0.453717 8.416892

H 0 -2.118073 -1.128958 0.370824

C 0 -3.643627 -1.984935 2.845810

H 0 -3.728991 -2.859646 3.494224

H 0 -4.563995 -1.411810 2.966717

H 0 -3.577434 -2.324116 1.809016

H 0 -1.552875 -1.832402 3.243903

**TS6R** (-8045.678754)

C -1 7.287463 0.464980 1.968559

C 0 5.838668 0.674046 2.429106

C 0 5.769528 1.469324 3.737405

C 0 5.020026 1.356599 1.328893

H 0 7.339544 -0.115961 1.043530

H 0 7.775655 1.427113 1.768156

H 0 5.389455 -0.312176 2.612465

H 0 6.201099 2.470387 3.607595

H 0 4.734583 1.591806 4.072046

H 0 6.323890 0.969853 4.538853

H 0 3.973161 1.478298 1.620415

H 0 5.424815 2.349015 1.095087

H 0 5.067135 0.762094 0.410354

C -1 2.866442 -2.654569 6.140004

C 0 2.848466 -2.197578 4.676847

C 0 1.930270 -3.085489 3.829267

C 0 2.443531 -0.721892 4.557892

H 0 1.855608 -2.616887 6.566985

H 0 3.222099 -3.685213 6.234209

H 0 3.870387 -2.295448 4.280529

H 0 0.889856 -3.010567 4.167673

H 0 1.966042 -2.794822 2.775727

H 0 2.214669 -4.141063 3.895672

H 0 1.434049 -0.563851 4.958782

H 0 3.125721 -0.077678 5.123031

H 0 2.450008 -0.396026 3.513440

C -1 0.466472 0.415840 10.770383

C 0 -0.096973 -0.489931 9.664311

C 0 -1.588359 -0.233965 9.425657

C 0 0.688688 -0.324789 8.357019

H 0 0.380670 1.472092 10.487456

H 0 -0.077246 0.278630 11.710895

H 0 0.014013 -1.531100 9.998506

H 0 -1.756713 0.780853 9.048424

H 0 -1.997135 -0.926057 8.683791

H 0 -2.167687 -0.348775 10.347688

H 0 0.623731 0.708497 7.996143

H 0 1.749538 -0.566490 8.483752

H 0 0.288202 -0.968517 7.567941

C -1 -3.085827 -4.133172 12.675941

C 0 -3.275200 -5.481772 11.963467

O 0 -4.363033 -5.796313 11.486407

C 0 -2.966118 -3.013389 11.629646

H 0 -3.966796 -3.978993 13.303815

H 0 -2.096360 -3.168757 10.983618

H 0 -3.855269 -2.993698 10.994430

H 0 -2.864666 -2.037440 12.112531

N 0 -2.168181 -6.279197 11.852356

C -1 -2.114497 -7.479997 11.011486

C 0 -0.915946 -7.446612 10.051127

C 0 -0.882892 -6.233229 9.149046

C 0 -1.759825 -6.118578 8.062122

C 0 0.022235 -5.193416 9.387181

C 0 -1.716220 -5.004522 7.224702

C 0 0.059490 -4.067563 8.561942

C 0 -0.810079 -3.970633 7.475284

H 0 -3.059799 -7.504355 10.464455

H 0 -0.946080 -8.364299 9.451618

H 0 0.012386 -7.491560 10.634855

H 0 -2.485098 -6.904027 7.867092

H 0 -1.295568 -5.922610 12.213240

H 0 0.717432 -5.272299 10.219938

H 0 -2.374590 -4.947420 6.366114

H 0 0.776700 -3.277706 8.760517

H 0 -0.786919 -3.102118 6.824882

C -1 0.152811 -6.400787 -0.188190

C 0 0.985307 -5.156778 -0.524791

C 0 0.963305 -4.877020 -2.032238

C 0 0.511007 -3.934366 0.272996

H 0 0.178511 -6.612454 0.886057

H 0 -0.897378 -6.247648 -0.467925

H 0 2.026158 -5.365888 -0.235727

H 0 -0.059258 -4.660371 -2.364438

H 0 1.585305 -4.015072 -2.289674

H 0 1.323627 -5.739223 -2.605443

H 0 -0.498091 -3.627744 -0.021292

H 0 0.492003 -4.151405 1.345809

H 0 1.172850 -3.075424 0.115243

C -1 -3.593469 -6.933951 3.871635

C 0 -2.508710 -6.392648 2.945284

S 0 -0.992822 -5.782496 3.781415

C 0 -0.456225 -7.269816 4.688483

H 0 -3.266581 -7.839734 4.392006

H 0 -3.886575 -6.194845 4.620229

H 0 -2.875221 -5.515838 2.404512

H 0 -2.216675 -7.137133 2.196445

H 0 -0.360200 -8.127833 4.017163

H 0 0.523225 -7.039175 5.112192

H 0 -1.135761 -7.508800 5.508976

C -1 -8.593110 -6.747994 0.713054

C 0 -7.746162 -5.689349 1.388413

C 0 -6.397033 -5.505639 1.059732

C 0 -8.296195 -4.823553 2.345043

C 0 -5.630022 -4.499792 1.649322

C 0 -7.543743 -3.813869 2.945814

C 0 -6.198361 -3.636259 2.594011

O 0 -5.392567 -2.704335 3.159038

H 0 -9.349933 -6.278203 0.075902

H 0 -7.980470 -7.407663 0.092625

H 0 -5.934072 -6.158661 0.323426

H 0 -9.342552 -4.932304 2.616715

H 0 -4.589204 -4.357912 1.380492

H 0 -7.991369 -3.159110 3.687451

H 0 -5.846899 -1.897209 3.518822

C -1 -3.718330 -4.900773 -2.924495

C 0 -3.113388 -3.992233 -1.846246

C 0 -4.123312 -2.973370 -1.324037

C 0 -3.640192 -2.014573 -0.241943

O 0 -4.495755 -1.281872 0.309231

O 0 -2.389757 -1.995883 0.041647

H 0 -4.061931 -4.316798 -3.785767

H 0 -2.237363 -3.465144 -2.238784

H 0 -2.746455 -4.598651 -1.010014

H 0 -5.010466 -3.472971 -0.919049

H 0 -4.493460 -2.344698 -2.145499

C -1 2.463035 -1.590003 -6.456241

C 0 2.684123 -1.437681 -4.967430

C 0 1.597278 -1.394702 -4.083123

C 0 3.976070 -1.377310 -4.428279

C 0 1.787061 -1.313459 -2.703042

C 0 4.176134 -1.316838 -3.046816

C 0 3.081357 -1.293356 -2.179104

H 0 2.409661 -2.648960 -6.735600

H 0 1.524513 -1.122937 -6.768797

H 0 0.588390 -1.434037 -4.481574

H 0 4.834173 -1.396118 -5.095417

H 0 0.930259 -1.284086 -2.038232

H 0 5.187173 -1.282353 -2.650374

H 0 3.219976 -1.275785 -1.103578

C -1 2.529262 4.058657 -4.610463

C 0 2.868394 3.573395 -3.200866

C 0 3.624418 2.250579 -3.229845

N 0 3.993380 1.834944 -1.843319

H 0 3.433905 4.206419 -5.210811

H 0 1.892610 3.336301 -5.130306

H 0 1.961857 3.442031 -2.600980

H 0 3.486608 4.323759 -2.688939

H 0 4.551287 2.325147 -3.804934

H 0 3.019233 1.445083 -3.650871

H 0 4.493848 2.595471 -1.369915

H 0 4.646579 1.042646 -1.867580

C -1 -2.422348 0.948513 -5.155416

C 0 -1.849618 1.084573 -3.738265

C 0 -1.949789 -0.244864 -2.981516

C 0 -0.410551 1.617889 -3.787162

H 0 -1.852708 0.208574 -5.731852

H 0 -2.465408 1.812881 -3.191765

H 0 -2.994697 -0.561341 -2.901289

H 0 -1.406352 -1.042021 -3.503270

H 0 -1.546570 -0.168617 -1.969436

H 0 0.217645 0.982422 -4.421873

H 0 -0.385862 2.631451 -4.205361

H 0 0.051383 1.645138 -2.797075

C -1 -8.784710 -2.890441 -3.161266

C 0 -9.046824 -3.984168 -2.128610

S 0 -9.923849 -3.405326 -0.626724

C 0 -8.612543 -2.349165 0.070837

H 0 -9.721325 -2.433993 -3.486657

H 0 -8.151823 -2.096361 -2.756385

H 0 -8.113260 -4.460033 -1.806486

H 0 -9.684558 -4.769008 -2.547460

H 0 -7.700772 -2.929331 0.222659

H 0 -8.416311 -1.492999 -0.571424

H 0 -8.960953 -1.997483 1.042550

C -1 -11.412720 -0.217271 -4.324025

C 0 -10.675817 0.367028 -3.146723

C 0 -9.647027 1.296681 -3.336348

C 0 -10.971948 -0.020884 -1.836467

C 0 -8.916657 1.802468 -2.265677

C 0 -10.255694 0.480351 -0.752429

C 0 -9.210184 1.378990 -0.963507

O 0 -8.482409 1.779780 0.126753

H 0 -12.264249 -0.820063 -3.997150

H 0 -11.791626 0.562623 -4.994220

H 0 -9.400473 1.624876 -4.343269

H 0 -11.755594 -0.749839 -1.656951

H 0 -8.126295 2.522191 -2.436183

H 0 -10.475726 0.158506 0.259342

H 0 -7.755222 2.382041 -0.160048

C -1 -5.818323 4.553613 -3.597719

C 0 -4.958857 4.213335 -2.366456

C 0 -5.627296 3.108358 -1.590816

O 0 -6.463869 3.351512 -0.701253

O 0 -5.306234 1.906953 -1.985522

H 0 -6.795164 4.921140 -3.285713

H 0 -4.866345 5.089683 -1.722314

H 0 -3.962396 3.883240 -2.670119

C -1 -9.497253 6.949793 -2.623640

C 0 -9.125990 6.157396 -1.371728

C 0 -10.042776 4.958395 -1.146760

O 0 -7.768715 5.754896 -1.547214

H 0 -9.470745 6.295629 -3.500966

H 0 -9.194730 6.825869 -0.496490

H 0 -11.071825 5.284354 -0.963051

H 0 -10.039975 4.298410 -2.018527

H 0 -9.718811 4.367596 -0.282785

H 0 -7.540890 5.000141 -0.977819

N 0 -7.542998 4.979437 6.175537

C 0 -6.492804 4.014932 5.787028

C 0 -5.220120 4.627375 5.207780

O 0 -4.191322 3.952764 5.149957

C 0 -7.181803 3.124112 4.729149

C 0 -8.662737 3.189643 5.118891

C 0 -8.842803 4.646189 5.565816

H 0 -6.181471 3.436684 6.661827

H 0 -6.771331 2.112938 4.701592

H 0 -7.039400 3.564459 3.735492

H 0 -8.862091 2.518474 5.961381

H 0 -9.332365 2.915883 4.300337

H 0 -9.044343 5.298603 4.705223

H 0 -9.657462 4.774240 6.281563

N 0 -5.343242 5.866576 4.678409

C -1 -4.224044 6.582281 4.088592

C 0 -4.593099 7.315632 2.800484

C 0 -5.202978 6.438326 1.696453

C 0 -5.426767 7.275361 0.431068

C 0 -4.349162 5.199329 1.405828

H 0 -3.447421 5.838472 3.906780

H 0 -3.677673 7.790332 2.421183

H 0 -5.286677 8.137400 3.029193

H 0 -6.182088 6.087290 2.052264

H 0 -5.964346 8.203129 0.660869

H 0 -4.464482 7.557276 -0.015984

H 0 -6.199661 6.363449 4.876304

H 0 -6.013352 6.738616 -0.318831

H 0 -4.236931 4.566339 2.289132

H 0 -4.817082 4.592755 0.628603

H 0 -3.343206 5.483699 1.072360

C -1 -0.159138 5.154735 5.412540

C 0 -0.894386 3.909661 4.923527

S 0 -0.747117 3.597599 3.120297

C 0 0.984920 3.024984 3.021343

H 0 0.919726 5.087225 5.238955

H 0 -0.528760 6.048635 4.901955

H 0 -1.970374 4.000412 5.095596

H 0 -0.548306 3.015613 5.454535

H 0 1.670425 3.763661 3.444054

H 0 1.109232 2.067615 3.533887

H 0 1.218059 2.889860 1.964486

C -1 -0.794538 9.087853 -2.940508

C 0 -0.544226 7.574260 -2.840387

C 0 -1.480677 6.925388 -1.803058

C 0 0.933562 7.292011 -2.537856

C 0 -1.374990 5.399368 -1.705860

H 0 -0.568852 9.581460 -1.987623

H 0 -0.777700 7.127687 -3.818929

H 0 -2.515226 7.196389 -2.050916

H 0 -1.279609 7.367644 -0.817039

H 0 1.584411 7.764912 -3.281700

H 0 1.208741 7.689844 -1.553126

H 0 1.152134 6.220054 -2.535057

H 0 -0.398220 5.076508 -1.332907

H 0 -2.128306 5.000315 -1.020579

H 0 -1.530526 4.927777 -2.683638

C -1 -8.525105 6.609403 7.776691

C 0 -7.345449 5.772238 7.283191

O 0 -6.252126 5.811483 7.837719

H 0 -9.263223 5.974073 8.278449

H 0 -8.139498 7.332875 8.493662

H 0 -1.839750 9.302037 -3.186452

H 0 -0.164657 9.545160 -3.710855

H 0 1.991561 5.009858 -4.573107

H 0 3.509568 -2.013304 6.753466

H 0 1.525354 0.205965 10.957882

H 0 -2.064988 -8.379553 11.637367

H 0 -2.202949 -4.149622 13.325968

H 0 -4.486965 -7.183508 3.288732

H 0 -9.119944 -7.368032 1.446266

H 0 -8.275897 -3.310167 -4.037577

H 0 -4.581609 -5.452689 -2.535580

H 0 -2.986992 -5.631392 -3.283861

H 0 0.515031 -7.283958 -0.726894

H 0 3.277970 -1.140739 -7.031067

H 0 -2.379175 1.898901 -5.699359

H 0 -3.467197 0.622026 -5.130677

H 0 -5.328562 5.334525 -4.187012

H 0 -8.785271 7.764934 -2.779880

H 0 -10.502027 7.374625 -2.534182

H 0 -5.935637 3.672298 -4.236101

H 0 -10.761798 -0.865271 -4.923537

H 0 -9.034615 7.128777 6.959662

H 0 -3.816001 7.297143 4.816743

H 0 -0.314856 5.286180 6.489432

O 0 6.749374 0.997645 -1.832547

O 0 -6.446717 0.022305 -0.791375

H 0 7.568239 0.570011 -1.523251

H 0 6.742934 1.848755 -1.365916

H 0 -5.783063 -0.574509 -0.343458

H 0 -7.074846 0.346785 -0.124335

C -1 5.110600 1.406538 8.853379

C 0 3.752238 2.112492 8.762442

C 0 3.801371 3.492879 9.426939

C 0 3.283900 2.219589 7.306377

H 0 5.070550 0.407794 8.405169

H 0 5.881524 1.978675 8.321817

H 0 3.017237 1.503980 9.307209

H 0 4.519123 4.145549 8.914566

H 0 2.823106 3.983996 9.394489

H 0 4.107213 3.420740 10.476195

H 0 2.309491 2.715062 7.234820

H 0 3.998540 2.800260 6.709459

H 0 3.187512 1.232403 6.845206

C -1 -7.139770 -6.830897 9.563923

C 0 -6.629119 -6.111754 8.337955

C 0 -5.503847 -5.281700 8.417734

C 0 -7.280899 -6.234058 7.102893

C 0 -5.064386 -4.573636 7.297890

C 0 -6.840980 -5.530639 5.982326

C 0 -5.733371 -4.684094 6.077303

H 0 -6.374490 -6.866256 10.343238

H 0 -8.014118 -6.318078 9.981960

H 0 -4.978613 -5.194655 9.363625

H 0 -8.147642 -6.886417 7.021875

H 0 -4.190361 -3.938350 7.384437

H 0 -7.357497 -5.632601 5.033323

H 0 -5.408575 -4.116236 5.211441

H 0 -7.451342 -7.853984 9.326738

H 0 5.435321 1.297134 9.893956

H 0 7.879037 -0.054085 2.729884

C -1 6.655679 5.733956 0.052919

C 0 6.901158 4.674452 -0.992428

O 0 6.191504 3.662666 -1.073400

H 0 6.825303 5.292715 1.039769

N 0 7.967204 4.880084 -1.803652

C 0 8.419123 3.938241 -2.811325

C 0 9.744699 3.256921 -2.477950

O 0 10.434487 2.764144 -3.360378

H 0 7.669662 3.145074 -2.896413

H 0 8.507124 5.722860 -1.672181

N 0 10.078188 3.203625 -1.151676

C -1 11.059216 2.194959 -0.765466

C 0 10.498999 0.821010 -1.097322

O 0 9.348656 0.532093 -0.734510

H 0 12.004531 2.408777 -1.259287

H 0 9.322310 3.347412 -0.496803

N 0 11.285672 -0.024296 -1.772636

C -1 10.755839 -1.337516 -2.120291

C 0 11.930383 -2.020905 -2.826951

C 0 12.638416 -0.847963 -3.521645

C 0 12.530927 0.294591 -2.500445

H 0 11.603734 -2.798965 -3.520069

H 0 12.595562 -2.483172 -2.089365

H 0 12.099499 -0.571895 -4.433789

H 0 13.674642 -1.061975 -3.792603

H 0 13.385221 0.297501 -1.812833

H 0 12.444137 1.270416 -2.980618

H 0 9.890693 -1.223396 -2.785731

H 0 5.606596 6.036141 0.012623

H 0 7.290128 6.615406 -0.059151

H 0 8.523563 4.422629 -3.784659

H 0 11.211174 2.259203 0.314910

H 0 10.411599 -1.857516 -1.223568

O 0 -5.979091 -0.261218 4.077928

H 0 -5.125040 0.039595 3.647241

H 0 -5.740523 -0.329050 5.014594

H 0 -5.811205 1.148442 -1.468791

N 0 -3.727095 0.287996 2.551206

C 0 -4.501812 0.176146 7.386870

C 0 -3.710478 1.127699 6.740266

C 0 -3.920821 1.545390 1.814640

C 0 -3.077609 1.634963 0.525158

C 0 -2.895036 0.747460 5.678529

C 0 -2.857460 -0.576685 5.210824

C 0 -1.669718 1.111680 0.671736

C 0 -2.032786 -0.928322 3.970085

C 0 -0.642596 1.631139 -0.089241

C 0 0.690966 1.145924 0.012943

C 0 -2.303864 0.126089 2.870542

C 0 0.918391 0.045957 0.919622

C 0 -3.639846 -1.522233 5.884801

C 0 -4.453738 -1.151944 6.962306

C 0 -0.078058 -0.452410 1.702162

C 0 -1.446781 -0.002425 1.572040

O 0 2.209971 -0.417539 1.006252

O 0 1.687671 1.654915 -0.623476

H 0 -3.745633 2.172885 7.026602

H 0 -2.317526 1.514947 5.179278

H 0 2.711213 0.207536 0.453801

H 0 -1.992041 -1.020975 0.829838

H 0 -3.666812 2.364996 2.492802

H 0 -3.055170 2.666202 0.157311

H 0 -1.988405 1.082919 3.293305

H 0 -3.657292 -2.549083 5.542982

H 0 -0.824354 2.462775 -0.763091

H 0 -5.066007 -1.902996 7.448821

H 0 0.162783 -1.242020 2.402183

H 0 -4.017183 -0.473049 1.929771

H 0 -3.569976 1.034432 -0.247202

H 0 -4.980348 1.646427 1.559937

H 0 -5.146222 0.463966 8.212245

H 0 3.107738 1.607508 -1.295969

H 0 -0.978125 -0.786069 4.248176

C 0 -2.201871 -2.375215 3.496014

H 0 -1.892701 -3.085291 4.266322

H 0 -3.239502 -2.589108 3.235887

H 0 -1.588688 -2.571775 2.614572

**Int10R** (-8045.703792)

C -1 7.281733 0.461973 1.970354

C 0 5.792525 0.726598 1.727858

C 0 4.917708 -0.378043 2.332150

C 0 5.379800 2.102064 2.263920

H 0 7.908789 1.223348 1.498657

H 0 7.503116 0.458244 3.045504

H 0 5.635104 0.724829 0.640556

H 0 5.029676 -0.408488 3.423607

H 0 3.863207 -0.205750 2.103145

H 0 5.194614 -1.364368 1.942849

H 0 4.336500 2.311287 2.015578

H 0 5.484029 2.138610 3.356106

H 0 6.000659 2.890641 1.829422

C -1 2.870291 -2.656441 6.141448

C 0 2.174623 -1.589888 5.288413

C 0 1.885897 -2.125346 3.882302

C 0 0.890070 -1.097303 5.964674

H 0 2.219718 -3.529631 6.275324

H 0 3.798721 -3.001092 5.673261

H 0 2.857250 -0.735465 5.192115

H 0 1.180297 -2.963320 3.921477

H 0 1.445808 -1.351809 3.245875

H 0 2.800171 -2.474493 3.391519

H 0 0.133133 -1.888870 5.979946

H 0 1.079883 -0.790027 6.999371

H 0 0.466193 -0.236744 5.442021

C -1 0.465274 0.413984 10.770304

C 0 -0.278412 0.718333 9.463513

C 0 -1.391283 -0.304326 9.214793

C 0 -0.839728 2.144644 9.457273

H 0 -0.219321 0.464672 11.626109

H 0 0.899831 -0.591323 10.751553

H 0 0.443954 0.638455 8.639301

H 0 -2.164381 -0.230671 9.990246

H 0 -1.873676 -0.143283 8.245814

H 0 -1.007504 -1.327265 9.230772

H 0 -1.563152 2.280352 10.270604

H 0 -0.049223 2.890924 9.586802

H 0 -1.359172 2.359482 8.516738

C -1 -3.085867 -4.132818 12.675968

C 0 -3.280179 -5.452496 11.906079

O 0 -4.322609 -5.684162 11.299368

C 0 -2.689437 -3.025547 11.684456

H 0 -4.040867 -3.900475 13.152916

H 0 -1.739813 -3.256597 11.192764

H 0 -3.446290 -2.931739 10.901893

H 0 -2.588715 -2.061171 12.190449

N 0 -2.214234 -6.318440 11.904631

C -1 -2.114314 -7.480980 11.012136

C 0 -1.081372 -7.269690 9.894514

C 0 -1.269899 -5.977831 9.129122

C 0 -2.503388 -5.652126 8.547339

C 0 -0.208853 -5.076522 8.992229

C 0 -2.662493 -4.464127 7.839680

C 0 -0.365190 -3.884098 8.279728

C 0 -1.594633 -3.576812 7.698569

H 0 -3.116388 -7.620781 10.603446

H 0 -1.139073 -8.132157 9.216700

H 0 -0.071938 -7.288508 10.323502

H 0 -3.354533 -6.315493 8.660858

H 0 -1.361396 -5.997659 12.340114

H 0 0.752975 -5.313694 9.440782

H 0 -3.621936 -4.238969 7.391826

H 0 0.471627 -3.200999 8.174957

H 0 -1.725699 -2.660919 7.134516

C -1 0.152596 -6.400163 -0.187832

C 0 0.660737 -4.952511 -0.160189

C 0 -0.417229 -3.981434 -0.654115

C 0 1.142460 -4.562827 1.241933

H 0 0.923743 -7.101486 0.148573

H 0 -0.714547 -6.515311 0.474636

H 0 1.516622 -4.881905 -0.846989

H 0 -1.282593 -3.985946 0.018705

H 0 -0.039256 -2.954824 -0.694319

H 0 -0.768197 -4.247254 -1.656857

H 0 0.326937 -4.629011 1.972014

H 0 1.948867 -5.220453 1.585072

H 0 1.518839 -3.535223 1.259472

C -1 -3.593173 -6.932687 3.872310

C 0 -2.543782 -6.014772 3.247310

S 0 -1.581280 -5.015786 4.444265

C 0 -0.771645 -6.317082 5.428087

H 0 -3.133222 -7.724534 4.470424

H 0 -4.271295 -6.367030 4.517486

H 0 -3.024281 -5.262314 2.617394

H 0 -1.850095 -6.581233 2.615427

H 0 -0.170907 -6.973310 4.792314

H 0 -0.122284 -5.806790 6.140710

H 0 -1.501458 -6.899662 5.992884

C -1 -8.592645 -6.746953 0.713624

C 0 -7.501874 -5.743771 1.005414

C 0 -6.172926 -5.976726 0.629990

C 0 -7.782934 -4.543281 1.672224

C 0 -5.162582 -5.057678 0.904803

C 0 -6.784364 -3.612531 1.957801

C 0 -5.459562 -3.864568 1.574838

O 0 -4.440634 -3.005065 1.823776

H 0 -8.891360 -7.291058 1.618085

H 0 -9.484665 -6.248083 0.321238

H 0 -5.921983 -6.894347 0.103679

H 0 -8.807291 -4.326731 1.961342

H 0 -4.141187 -5.241513 0.590177

H 0 -7.025677 -2.681470 2.462321

H 0 -4.770255 -2.127980 2.130615

C -1 -3.718466 -4.900759 -2.924291

C 0 -3.749790 -3.580015 -2.151642

C 0 -5.175659 -3.065476 -2.006884

C 0 -5.321041 -1.838108 -1.141516

O 0 -6.611677 -1.581578 -0.892362

O 0 -4.406029 -1.149187 -0.718545

H 0 -4.131097 -4.783462 -3.932901

H 0 -3.129625 -2.825525 -2.647669

H 0 -3.320930 -3.712688 -1.154711

H 0 -5.819400 -3.836984 -1.571567

H 0 -5.614443 -2.827931 -2.985257

C -1 2.462308 -1.589867 -6.458750

C 0 2.692769 -1.364619 -4.979216

C 0 1.611794 -1.294214 -4.089057

C 0 3.985499 -1.237993 -4.454903

C 0 1.812451 -1.095277 -2.722758

C 0 4.196246 -1.055956 -3.085454

C 0 3.107795 -0.981646 -2.213479

H 0 3.325778 -1.270874 -7.049153

H 0 2.288664 -2.650909 -6.674943

H 0 0.599623 -1.389395 -4.473022

H 0 4.838636 -1.281985 -5.127472

H 0 0.958510 -1.017688 -2.057258

H 0 5.207949 -0.946634 -2.703340

H 0 3.257124 -0.816231 -1.151521

C -1 2.558023 4.063756 -4.609324

C 0 3.756140 3.828437 -3.687388

C 0 3.905146 2.348028 -3.301062

N 0 4.375046 2.193004 -1.900903

H 0 2.603207 3.421777 -5.496078

H 0 1.634272 3.839810 -4.073998

H 0 3.617951 4.432484 -2.783736

H 0 4.684190 4.181045 -4.156255

H 0 4.576885 1.838454 -4.001116

H 0 2.939069 1.840784 -3.372163

H 0 5.122720 2.853031 -1.694565

H 0 4.820869 1.279963 -1.803710

C -1 -2.421662 0.948863 -5.153368

C 0 -1.756396 1.385066 -3.839312

C 0 -1.822320 0.280631 -2.776487

C 0 -0.317317 1.844923 -4.091016

H 0 -1.889636 0.093554 -5.587684

H 0 -2.310778 2.250759 -3.449570

H 0 -2.853076 0.027571 -2.510839

H 0 -1.336666 -0.635145 -3.135991

H 0 -1.310131 0.589334 -1.859447

H 0 0.293665 1.029532 -4.491399

H 0 -0.289131 2.668153 -4.812364

H 0 0.156413 2.185866 -3.166716

C -1 -8.784347 -2.890662 -3.160957

C 0 -9.051469 -3.944331 -2.088513

S 0 -10.453078 -3.553795 -0.969934

C 0 -9.802034 -2.067646 -0.131639

H 0 -9.686688 -2.682087 -3.741714

H 0 -8.432325 -1.964393 -2.708910

H 0 -8.164712 -4.111316 -1.470339

H 0 -9.325922 -4.903793 -2.538183

H 0 -8.828876 -2.282856 0.312144

H 0 -9.720168 -1.229024 -0.821394

H 0 -10.516645 -1.810658 0.653319

C -1 -11.412714 -0.216654 -4.323515

C 0 -10.701311 0.419512 -3.156346

C 0 -9.307236 0.502108 -3.128116

C 0 -11.405225 0.910069 -2.048231

C 0 -8.629256 1.037221 -2.035731

C 0 -10.745169 1.478351 -0.960357

C 0 -9.352072 1.548551 -0.955060

O 0 -8.724865 2.132279 0.130633

H 0 -11.728220 -1.237515 -4.077195

H 0 -12.311215 0.343327 -4.601943

H 0 -8.733632 0.124676 -3.969037

H 0 -12.490433 0.855273 -2.039573

H 0 -7.548367 1.044391 -2.032809

H 0 -11.294218 1.880903 -0.116019

H 0 -7.938772 2.624511 -0.201297

C -1 -5.818081 4.553772 -3.597205

C 0 -4.948115 3.622878 -2.741925

C 0 -5.683294 2.881543 -1.653386

O 0 -6.579320 3.381829 -0.962826

O 0 -5.260950 1.646242 -1.465220

H 0 -6.335051 5.295326 -2.988875

H 0 -4.174996 4.206888 -2.224428

H 0 -4.419419 2.885202 -3.349440

C -1 -9.497499 6.950096 -2.623536

C 0 -9.157209 5.630483 -1.962995

C 0 -9.276321 4.476968 -2.958981

O 0 -7.838655 5.795643 -1.446024

H 0 -8.807502 7.135847 -3.452801

H 0 -9.855062 5.442721 -1.129376

H 0 -10.327273 4.300494 -3.209082

H 0 -8.739681 4.707732 -3.883806

H 0 -8.879133 3.546181 -2.554904

H 0 -7.473803 4.929474 -1.198273

N 0 -7.299282 4.912863 6.435469

C 0 -6.136135 4.030802 6.206644

C 0 -4.973604 4.641007 5.417313

O 0 -3.936798 3.994264 5.263408

C 0 -6.720729 2.829885 5.423524

C 0 -8.214536 2.849761 5.768466

C 0 -8.524489 4.348814 5.848968

H 0 -5.723222 3.728016 7.173268

H 0 -6.214140 1.899197 5.682818

H 0 -6.580781 2.990552 4.348989

H 0 -8.387614 2.387445 6.746333

H 0 -8.833429 2.331907 5.031470

H 0 -8.699443 4.765170 4.847532

H 0 -9.396222 4.573478 6.464791

N 0 -5.214633 5.846202 4.856177

C -1 -4.223998 6.582909 4.089238

C 0 -4.880984 7.462632 3.024704

C 0 -5.717222 6.718809 1.969604

C 0 -6.401925 7.716678 1.028565

C 0 -4.872946 5.713724 1.178498

H 0 -3.552072 5.844619 3.647142

H 0 -4.088327 8.030910 2.520278

H 0 -5.515015 8.210262 3.522946

H 0 -6.498833 6.153186 2.497081

H 0 -7.039444 8.412712 1.586073

H 0 -5.652993 8.313792 0.493087

H 0 -6.055960 6.323498 5.144529

H 0 -7.014972 7.203676 0.282047

H 0 -4.435394 4.952572 1.829564

H 0 -5.479810 5.200686 0.428253

H 0 -4.052421 6.224107 0.657753

C -1 -0.162009 5.155518 5.412735

C 0 0.452818 3.825464 5.839518

S 0 0.585872 2.581021 4.498446

C 0 1.904175 3.333519 3.489728

H 0 0.405907 5.620067 4.601233

H 0 -1.193739 5.008621 5.080008

H 0 -0.165279 3.346424 6.604383

H 0 1.449793 3.964669 6.269345

H 0 1.575727 4.267165 3.028032

H 0 2.797917 3.504683 4.095544

H 0 2.140716 2.620957 2.701091

C -1 -0.794444 9.088083 -2.940457

C 0 -0.459745 7.611265 -3.204619

C 0 -1.745119 6.786208 -3.401816

C 0 0.421797 7.041173 -2.084678

C 0 -1.508804 5.355989 -3.894884

H 0 -1.374448 9.194805 -2.015755

H 0 0.110835 7.559114 -4.144588

H 0 -2.391755 7.308005 -4.119227

H 0 -2.301146 6.765271 -2.453040

H 0 1.323911 7.648081 -1.950366

H 0 -0.119952 7.041822 -1.130524

H 0 0.743507 6.014571 -2.280960

H 0 -0.940522 4.758921 -3.177388

H 0 -2.455011 4.835851 -4.075778

H 0 -0.946691 5.353141 -4.835588

C -1 -8.524974 6.609636 7.776693

C 0 -7.238811 5.862122 7.432650

O 0 -6.179435 6.105222 7.999400

H 0 -9.216584 5.959267 8.323298

H 0 -8.255876 7.449814 8.415311

H 0 -1.387273 9.514578 -3.756460

H 0 0.115121 9.688246 -2.832448

H 0 2.513228 5.103234 -4.950048

H 0 3.118234 -2.269127 7.136642

H 0 1.274951 1.129167 10.948636

H 0 -1.861786 -8.372581 11.596658

H 0 -2.327857 -4.240900 13.460633

H 0 -4.189094 -7.406562 3.084562

H 0 -8.262178 -7.487507 -0.020432

H 0 -8.005217 -3.244772 -3.846997

H 0 -4.312431 -5.665142 -2.410683

H 0 -2.697162 -5.281728 -3.022974

H 0 -0.158515 -6.696190 -1.195773

H 0 1.585124 -1.038906 -6.811824

H 0 -2.412792 1.756960 -5.893028

H 0 -3.462114 0.643677 -4.993873

H 0 -5.182203 5.068945 -4.322592

H 0 -9.402403 7.774930 -1.911414

H 0 -10.519675 6.946494 -3.015785

H 0 -6.565684 3.981034 -4.151706

H 0 -10.763162 -0.273578 -5.200961

H 0 -9.043256 6.972786 6.884452

H 0 -3.614255 7.203890 4.759554

H 0 -0.170723 5.854205 6.257207

O 0 7.125959 0.845488 -1.684929

O 0 -6.570053 0.572043 0.628567

H 0 7.858298 0.359423 -1.266551

H 0 7.056457 1.635640 -1.125025

H 0 -6.165680 0.251987 1.483695

H 0 -7.435909 1.001920 0.780228

C -1 5.108489 1.406909 8.854551

C 0 3.781560 1.454350 8.084906

C 0 2.892216 2.589811 8.602068

C 0 4.010030 1.590350 6.574061

H 0 5.741322 0.582998 8.507729

H 0 5.669637 2.339135 8.715321

H 0 3.252617 0.506221 8.260631

H 0 3.360629 3.564286 8.414434

H 0 1.917697 2.584860 8.107108

H 0 2.721699 2.506694 9.680418

H 0 3.060657 1.597131 6.028259

H 0 4.538330 2.525361 6.346681

H 0 4.616362 0.765768 6.184287

C -1 -7.139667 -6.830577 9.563542

C 0 -6.896265 -6.027561 8.308231

C 0 -6.418565 -4.712561 8.397393

C 0 -7.163683 -6.550058 7.036371

C 0 -6.252658 -3.935251 7.251821

C 0 -6.977465 -5.781915 5.884785

C 0 -6.529514 -4.465010 5.988667

H 0 -6.315270 -6.704104 10.271737

H 0 -8.057783 -6.499939 10.064261

H 0 -6.165910 -4.309148 9.373946

H 0 -7.523855 -7.571970 6.948274

H 0 -5.900229 -2.913248 7.347779

H 0 -7.187133 -6.203594 4.906001

H 0 -6.395746 -3.866176 5.092745

H 0 -7.253362 -7.895383 9.339527

H 0 4.940862 1.275875 9.928970

H 0 7.590936 -0.505183 1.561941

C -1 6.646834 5.723901 0.042075

C 0 7.155982 4.575370 -0.793233

O 0 6.970973 3.402148 -0.435961

H 0 7.087800 5.651248 1.039341

N 0 7.815714 4.874411 -1.934093

C 0 8.348352 3.856081 -2.830560

C 0 9.735792 3.310671 -2.473280

O 0 10.557445 3.077803 -3.353149

H 0 7.672205 2.993507 -2.829239

H 0 8.012017 5.841875 -2.141566

N 0 9.951608 3.073163 -1.146080

C -1 11.056577 2.193625 -0.765876

C 0 10.603540 0.761955 -0.973677

O 0 9.606446 0.349148 -0.362336

H 0 11.936289 2.471730 -1.341365

H 0 9.119871 2.986857 -0.569821

N 0 11.269311 0.002864 -1.853187

C -1 10.757000 -1.336903 -2.120334

C 0 11.770937 -1.918909 -3.112596

C 0 12.262765 -0.677540 -3.873114

C 0 12.345018 0.399354 -2.782628

H 0 11.328499 -2.676942 -3.762678

H 0 12.602599 -2.383714 -2.571445

H 0 11.523734 -0.377895 -4.623684

H 0 13.218987 -0.826345 -4.380031

H 0 13.316227 0.371874 -2.272246

H 0 12.161774 1.402345 -3.169519

H 0 9.752516 -1.266077 -2.556090

H 0 5.565452 5.612849 0.157072

H 0 6.865454 6.706544 -0.381773

H 0 8.408161 4.259667 -3.840853

H 0 11.264808 2.337773 0.297037

H 0 10.668131 -1.904530 -1.191304

O 0 -5.177318 -0.442758 2.597132

H 0 -4.319344 0.083058 2.337476

H 0 -5.220968 -0.408412 3.563423

N 0 -3.158894 0.900707 1.601278

C 0 -4.178599 -0.769963 6.269949

C 0 -3.865507 -1.866684 5.465761

C 0 -3.575077 2.280140 1.283605

C 0 -2.693739 2.901460 0.193721

C 0 -2.969173 -1.730164 4.403993

C 0 -2.358950 -0.504688 4.120944

C 0 -1.231814 2.512779 0.298006

C 0 -1.424901 -0.334694 2.922850

C 0 -0.302384 3.145422 -0.525718

C 0 1.038699 2.772503 -0.539849

C 0 -1.791490 0.957622 2.175124

C 0 1.457151 1.693247 0.258400

C 0 -2.658327 0.583102 4.960555

C 0 -3.564733 0.460045 6.012141

C 0 0.536489 1.082911 1.108206

C 0 -0.800448 1.483329 1.152555

O 0 2.752218 1.254743 0.301513

O 0 1.893324 3.478308 -1.337134

H 0 -4.310461 -2.836788 5.650624

H 0 -2.777911 -2.589612 3.779284

H 0 3.291816 1.622816 -0.432960

H 0 -3.508976 2.849035 2.217241

H 0 -0.424599 -0.161433 3.336270

H 0 -2.802102 3.991917 0.201650

H 0 -1.841161 1.739234 2.935898

H 0 -2.205957 1.553724 4.794350

H 0 -0.607487 3.964347 -1.169433

H 0 -3.788261 1.335978 6.613221

H 0 0.908197 0.290401 1.746186

H 0 -3.146391 0.351284 0.737162

H 0 -3.054593 2.555884 -0.781538

H 0 -4.624805 2.282587 0.985981

H 0 2.784130 3.068589 -1.335816

H 0 -4.877202 -0.870637 7.095619

C 0 -1.353636 -1.552692 1.995078

H 0 -0.972619 -2.430635 2.521454

H 0 -2.328385 -1.813415 1.575539

H 0 -0.677509 -1.350022 1.162134

H 0 -5.692656 1.236615 -0.656362

H 0 -6.667647 -0.787305 -0.295636

**E:PR** (-8045.715002)

C -1 7.281726 0.461975 1.970360

C 0 5.789447 0.695653 1.714094

C 0 4.932708 -0.429173 2.306852

C 0 5.342104 2.060351 2.250014

H 0 7.896053 1.239240 1.507861

H 0 7.492798 0.458504 3.047549

H 0 5.642548 0.693541 0.625444

H 0 5.035649 -0.461571 3.399174

H 0 3.876591 -0.277779 2.069644

H 0 5.233020 -1.408112 1.916472

H 0 4.293154 2.242419 2.003447

H 0 5.447525 2.100486 3.342058

H 0 5.941639 2.864494 1.813916

C -1 2.870272 -2.656422 6.141438

C 0 2.201894 -1.590787 5.265395

C 0 1.897212 -2.149859 3.871964

C 0 0.932454 -1.046841 5.931445

H 0 2.198560 -3.510411 6.292265

H 0 3.790246 -3.032970 5.681436

H 0 2.907266 -0.757724 5.148192

H 0 1.168464 -2.966726 3.929071

H 0 1.482019 -1.377508 3.217018

H 0 2.800799 -2.536240 3.389775

H 0 0.156649 -1.819229 5.974616

H 0 1.133762 -0.713159 6.955715

H 0 0.527639 -0.191367 5.384116

C -1 0.465271 0.413986 10.770289

C 0 -0.254546 0.758354 9.459408

C 0 -1.366325 -0.252259 9.158863

C 0 -0.810487 2.186530 9.487282

H 0 -0.233190 0.446438 11.615528

H 0 0.894263 -0.593126 10.732020

H 0 0.482846 0.699893 8.646464

H 0 -2.150518 -0.203992 9.924900

H 0 -1.835357 -0.055932 8.188989

H 0 -0.984977 -1.276194 9.144801

H 0 -1.549350 2.299152 10.289976

H 0 -0.020019 2.924843 9.656030

H 0 -1.310886 2.434228 8.544435

C -1 -3.085867 -4.132835 12.675960

C 0 -3.277642 -5.445372 11.893905

O 0 -4.309504 -5.662363 11.263701

C 0 -2.673367 -3.019796 11.697351

H 0 -4.044997 -3.899052 13.143869

H 0 -1.719724 -3.252053 11.213830

H 0 -3.421929 -2.915816 10.908117

H 0 -2.573305 -2.060102 12.212373

N 0 -2.221100 -6.321932 11.908273

C -1 -2.114325 -7.480958 11.012136

C 0 -1.060283 -7.270603 9.914212

C 0 -1.234147 -5.979097 9.144556

C 0 -2.459487 -5.651303 8.547346

C 0 -0.168535 -5.081671 9.016669

C 0 -2.606022 -4.466205 7.832805

C 0 -0.312662 -3.890995 8.298137

C 0 -1.534094 -3.582560 7.700872

H 0 -3.109907 -7.612701 10.585507

H 0 -1.105843 -8.132777 9.235112

H 0 -0.059113 -7.290181 10.362050

H 0 -3.314622 -6.310520 8.653669

H 0 -1.375910 -6.015864 12.368458

H 0 0.787091 -5.320050 9.477535

H 0 -3.559844 -4.241583 7.373755

H 0 0.527345 -3.210267 8.202164

H 0 -1.656001 -2.667712 7.132731

C -1 0.152563 -6.400156 -0.187782

C 0 0.761553 -4.992252 -0.171905

C 0 -0.227679 -3.959075 -0.721800

C 0 1.222946 -4.608121 1.238607

H 0 0.859731 -7.146615 0.189635

H 0 -0.742238 -6.438206 0.446178

H 0 1.642743 -4.997176 -0.829550

H 0 -1.114364 -3.890536 -0.081762

H 0 0.224728 -2.963293 -0.768242

H 0 -0.562787 -4.219533 -1.731395

H 0 0.383550 -4.616521 1.944071

H 0 1.977433 -5.306722 1.616860

H 0 1.659712 -3.604427 1.252939

C -1 -3.593143 -6.932652 3.872254

C 0 -2.501858 -6.061828 3.251953

S 0 -1.527478 -5.073748 4.448526

C 0 -0.760413 -6.379392 5.459953

H 0 -3.171716 -7.727570 4.494286

H 0 -4.265639 -6.333037 4.492261

H 0 -2.945573 -5.303454 2.602974

H 0 -1.815905 -6.661118 2.642576

H 0 -0.159696 -7.052051 4.841765

H 0 -0.116388 -5.874383 6.181246

H 0 -1.512389 -6.943264 6.014317

C -1 -8.592658 -6.746955 0.713635

C 0 -7.493302 -5.769933 1.046633

C 0 -6.167120 -6.002700 0.664277

C 0 -7.766889 -4.593507 1.757848

C 0 -5.148257 -5.105235 0.976735

C 0 -6.759914 -3.686771 2.082855

C 0 -5.438347 -3.939737 1.693117

O 0 -4.413269 -3.095796 1.983097

H 0 -8.917762 -7.304500 1.600646

H 0 -9.468231 -6.223910 0.315688

H 0 -5.925416 -6.901373 0.102359

H 0 -8.790742 -4.376362 2.047563

H 0 -4.128673 -5.285662 0.654917

H 0 -6.990643 -2.770645 2.618149

H 0 -4.754346 -2.231242 2.298211

C -1 -3.718458 -4.900774 -2.924308

C 0 -3.642037 -3.634627 -2.061677

C 0 -5.021069 -3.015399 -1.876300

C 0 -5.097722 -1.829887 -0.939948

O 0 -6.368780 -1.551808 -0.662949

O 0 -4.140896 -1.202991 -0.499846

H 0 -4.119458 -4.680192 -3.919958

H 0 -2.960596 -2.902324 -2.508026

H 0 -3.225656 -3.873645 -1.078891

H 0 -5.725475 -3.760332 -1.491543

H 0 -5.436067 -2.684310 -2.837993

C -1 2.462310 -1.589852 -6.458754

C 0 2.716804 -1.374313 -4.982415

C 0 1.648722 -1.295513 -4.077652

C 0 4.018026 -1.264289 -4.475884

C 0 1.870208 -1.106075 -2.713564

C 0 4.249508 -1.094280 -3.108301

C 0 3.173920 -1.014464 -2.221238

H 0 3.328288 -1.297559 -7.059128

H 0 2.252325 -2.644504 -6.673645

H 0 0.630592 -1.376366 -4.447999

H 0 4.861001 -1.313532 -5.160721

H 0 1.025489 -1.020511 -2.037456

H 0 5.266915 -0.998777 -2.738218

H 0 3.340381 -0.863325 -1.160002

C -1 2.558021 4.063752 -4.609320

C 0 3.775580 3.816999 -3.716055

C 0 3.937446 2.331894 -3.348199

N 0 4.393141 2.163906 -1.943786

H 0 2.588173 3.438023 -5.508128

H 0 1.645331 3.827129 -4.061098

H 0 3.657302 4.411000 -2.802644

H 0 4.692545 4.177118 -4.200242

H 0 4.624968 1.839073 -4.044653

H 0 2.979249 1.812643 -3.437862

H 0 5.130588 2.830225 -1.720451

H 0 4.849684 1.255066 -1.853835

C -1 -2.421680 0.948867 -5.153378

C 0 -1.727810 1.365201 -3.849156

C 0 -1.793833 0.250141 -2.797331

C 0 -0.288310 1.807712 -4.127168

H 0 -1.909493 0.089676 -5.604224

H 0 -2.265817 2.232805 -3.442136

H 0 -2.824861 0.037698 -2.498915

H 0 -1.357556 -0.679873 -3.183401

H 0 -1.237373 0.526558 -1.896072

H 0 0.300147 0.993070 -4.561514

H 0 -0.266694 2.645884 -4.831260

H 0 0.215830 2.122191 -3.209863

C -1 -8.784354 -2.890657 -3.160972

C 0 -9.029769 -3.951628 -2.089926

S 0 -10.363630 -3.536524 -0.899931

C 0 -9.642903 -2.059572 -0.102941

H 0 -9.705752 -2.650083 -3.696881

H 0 -8.393787 -1.975525 -2.714908

H 0 -8.118219 -4.153920 -1.518506

H 0 -9.352760 -4.896573 -2.538331

H 0 -8.646221 -2.287139 0.278419

H 0 -9.596326 -1.219674 -0.794641

H 0 -10.303357 -1.796145 0.726141

C -1 -11.412695 -0.216641 -4.323480

C 0 -10.672521 0.408235 -3.166326

C 0 -9.277421 0.496635 -3.164629

C 0 -11.356331 0.891232 -2.042777

C 0 -8.577024 1.034984 -2.086694

C 0 -10.676368 1.462019 -0.969245

C 0 -9.282712 1.544819 -0.991446

O 0 -8.638706 2.139007 0.070727

H 0 -11.767368 -1.222273 -4.066906

H 0 -12.290207 0.374257 -4.606157

H 0 -8.720480 0.125607 -4.020211

H 0 -12.441224 0.831061 -2.012616

H 0 -7.494062 1.059299 -2.097657

H 0 -11.210101 1.862757 -0.113987

H 0 -7.866490 2.679768 -0.292529

C -1 -5.818062 4.553763 -3.597209

C 0 -4.959576 3.704777 -2.646984

C 0 -5.714097 2.912946 -1.581104

O 0 -6.674704 3.475123 -0.961042

O 0 -5.318373 1.739603 -1.322266

H 0 -6.380144 5.313776 -3.056355

H 0 -4.266746 4.362615 -2.103856

H 0 -4.343605 2.990752 -3.199743

C -1 -9.497433 6.950040 -2.623488

C 0 -9.126049 5.639925 -1.962748

C 0 -9.244209 4.483635 -2.957657

O 0 -7.810125 5.829985 -1.463143

H 0 -8.819474 7.143100 -3.461133

H 0 -9.815802 5.441430 -1.123050

H 0 -10.297019 4.288270 -3.186622

H 0 -8.729944 4.724643 -3.892767

H 0 -8.817231 3.563872 -2.561897

H 0 -7.410162 4.958953 -1.250754

N 0 -7.412298 4.963790 6.280132

C 0 -6.329448 4.005418 5.980930

C 0 -5.135577 4.572343 5.204293

O 0 -4.197240 3.829402 4.901450

C 0 -7.015261 2.895255 5.143590

C 0 -8.511880 3.063933 5.436751

C 0 -8.657741 4.581202 5.598554

H 0 -5.929250 3.624236 6.924852

H 0 -6.625177 1.908593 5.399831

H 0 -6.817345 3.059333 4.079468

H 0 -8.777384 2.570323 6.377864

H 0 -9.149877 2.661879 4.645998

H 0 -8.724692 5.072475 4.618571

H 0 -9.531629 4.870159 6.182861

N 0 -5.234775 5.868355 4.848345

C -1 -4.224028 6.582904 4.089200

C 0 -4.836528 7.438790 2.978275

C 0 -5.693972 6.680153 1.951561

C 0 -6.260140 7.652477 0.910617

C 0 -4.914594 5.545135 1.277741

H 0 -3.548035 5.826436 3.687007

H 0 -4.014207 7.947189 2.456973

H 0 -5.442869 8.236002 3.431709

H 0 -6.538483 6.225679 2.489873

H 0 -6.840310 8.449642 1.390521

H 0 -5.445054 8.130559 0.352321

H 0 -6.034626 6.376525 5.195640

H 0 -6.902339 7.139100 0.189285

H 0 -4.570454 4.808172 2.009311

H 0 -5.537030 5.026048 0.544646

H 0 -4.033148 5.941889 0.756505

C -1 -0.161997 5.155507 5.412741

C 0 0.377966 3.766911 5.747984

S 0 0.482125 2.621486 4.318436

C 0 1.837159 3.394577 3.375868

H 0 0.459313 5.660918 4.667851

H 0 -1.182227 5.084361 5.024303

H 0 -0.278800 3.260603 6.461486

H 0 1.370353 3.824267 6.206168

H 0 1.553654 4.378737 2.997097

H 0 2.735723 3.471221 3.993313

H 0 2.042468 2.739696 2.530152

C -1 -0.794442 9.088079 -2.940456

C 0 -0.452790 7.616597 -3.224802

C 0 -1.732853 6.768222 -3.342197

C 0 0.504246 7.063338 -2.159512

C 0 -1.505558 5.349639 -3.871677

H 0 -1.323219 9.185495 -1.984550

H 0 0.061224 7.572954 -4.197280

H 0 -2.439941 7.284448 -4.004008

H 0 -2.220265 6.723657 -2.357397

H 0 1.410302 7.675531 -2.090685

H 0 0.025324 7.071682 -1.172303

H 0 0.816025 6.035521 -2.366152

H 0 -0.852573 4.764151 -3.219048

H 0 -2.449834 4.804743 -3.963459

H 0 -1.036566 5.371395 -4.862019

C -1 -8.524975 6.609638 7.776702

C 0 -7.304433 5.779756 7.386735

O 0 -6.251777 5.848329 8.011090

H 0 -9.303144 5.974440 8.213686

H 0 -8.203826 7.334143 8.523696

H 0 -1.439429 9.504746 -3.721018

H 0 0.110211 9.702793 -2.883726

H 0 2.503405 5.108890 -4.930281

H 0 3.126979 -2.254615 7.128567

H 0 1.275969 1.119508 10.980173

H 0 -1.877453 -8.376695 11.596745

H 0 -2.337390 -4.250112 13.468324

H 0 -4.188701 -7.400834 3.081217

H 0 -8.261190 -7.477665 -0.029777

H 0 -8.043892 -3.252676 -3.884801

H 0 -4.373638 -5.646810 -2.460777

H 0 -2.730946 -5.354939 -3.053240

H 0 -0.144978 -6.696428 -1.199645

H 0 1.598779 -1.011128 -6.800966

H 0 -2.415060 1.763322 -5.885807

H 0 -3.463448 0.660120 -4.976591

H 0 -5.171468 5.046342 -4.331034

H 0 -9.405492 7.779848 -1.916029

H 0 -10.524349 6.931819 -3.004547

H 0 -6.526461 3.925485 -4.144415

H 0 -10.769386 -0.307048 -5.203005

H 0 -8.961359 7.129703 6.919067

H 0 -3.632879 7.216345 4.763956

H 0 -0.179388 5.779912 6.312972

O 0 7.132683 0.841724 -1.681736

O 0 -6.497044 0.660217 0.721373

H 0 7.867566 0.358650 -1.263984

H 0 7.061501 1.633196 -1.123754

H 0 -5.858702 -0.051650 2.082984

H 0 -7.394462 1.067190 0.696348

C -1 5.108483 1.406914 8.854546

C 0 3.761523 1.481966 8.121452

C 0 2.918284 2.648526 8.647242

C 0 3.951044 1.591649 6.602853

H 0 5.706447 0.559339 8.503244

H 0 5.692414 2.319833 8.685080

H 0 3.212333 0.551069 8.324932

H 0 3.414308 3.606255 8.445624

H 0 1.934710 2.672915 8.169895

H 0 2.763571 2.576574 9.728825

H 0 2.988051 1.603610 6.080797

H 0 4.486830 2.514575 6.346145

H 0 4.533722 0.751926 6.209873

C -1 -7.139662 -6.830583 9.563551

C 0 -6.885645 -6.000197 8.328952

C 0 -6.332346 -4.718036 8.448919

C 0 -7.218180 -6.462846 7.049057

C 0 -6.157071 -3.911620 7.325150

C 0 -7.022246 -5.666496 5.918646

C 0 -6.499416 -4.380314 6.054048

H 0 -6.313060 -6.733747 10.273435

H 0 -8.052335 -6.498711 10.073520

H 0 -6.026601 -4.366196 9.430100

H 0 -7.636550 -7.460057 6.937973

H 0 -5.742437 -2.916140 7.446862

H 0 -7.281318 -6.040545 4.932336

H 0 -6.356987 -3.759378 5.174974

H 0 -7.270744 -7.887722 9.313718

H 0 4.967151 1.296973 9.934976

H 0 7.616066 -0.495921 1.560342

C -1 6.646826 5.723900 0.042076

C 0 7.147561 4.572714 -0.794831

O 0 6.949862 3.400153 -0.441373

H 0 7.082026 5.642851 1.041231

N 0 7.813908 4.869847 -1.932117

C 0 8.344322 3.851506 -2.829587

C 0 9.731652 3.305413 -2.473307

O 0 10.551070 3.068617 -3.353904

H 0 7.667563 2.989576 -2.827939

H 0 8.018641 5.836543 -2.135214

N 0 9.950699 3.072574 -1.145526

C -1 11.056572 2.193623 -0.765876

C 0 10.604191 0.761684 -0.972787

O 0 9.607307 0.349161 -0.360251

H 0 11.935491 2.472125 -1.342335

H 0 9.120726 2.987273 -0.566952

N 0 11.269044 0.002912 -1.852751

C -1 10.757000 -1.336902 -2.120334

C 0 11.772366 -1.919459 -3.110758

C 0 12.265431 -0.678508 -3.871106

C 0 12.346114 0.398955 -2.781115

H 0 11.330827 -2.677716 -3.761155

H 0 12.603095 -2.384109 -2.568080

H 0 11.527684 -0.379186 -4.623043

H 0 13.222443 -0.827641 -4.376362

H 0 13.316430 0.371868 -2.269144

H 0 12.163137 1.401624 -3.168837

H 0 9.753293 -1.265894 -2.557892

H 0 5.563620 5.625089 0.150920

H 0 6.878594 6.705531 -0.377006

H 0 8.403568 4.255113 -3.839928

H 0 11.265741 2.338631 0.296718

H 0 10.666486 -1.904105 -1.191216

O 0 -5.243149 -0.537152 2.694573

H 0 -3.805266 0.391592 2.178336

H 0 -5.437514 -0.315530 3.614984

N 0 -3.134576 0.836304 1.515368

C 0 -4.244525 -0.774137 6.151905

C 0 -3.836026 -1.901556 5.437833

C 0 -3.556294 2.244516 1.215744

C 0 -2.681718 2.798152 0.097583

C 0 -2.911799 -1.783163 4.400541

C 0 -2.363596 -0.544975 4.054669

C 0 -1.219661 2.417967 0.203551

C 0 -1.409540 -0.412811 2.862329

C 0 -0.287870 3.064299 -0.605483

C 0 1.055997 2.700312 -0.608594

C 0 -1.740209 0.871745 2.095100

C 0 1.476797 1.610682 0.177560

C 0 -2.750651 0.576201 4.810794

C 0 -3.691736 0.469792 5.834648

C 0 0.553380 0.980535 1.008445

C 0 -0.784114 1.379126 1.043065

O 0 2.773456 1.188946 0.231059

O 0 1.913292 3.424081 -1.378553

H 0 -4.236222 -2.880994 5.667407

H 0 -2.649838 -2.669674 3.844427

H 0 3.316276 1.565077 -0.498782

H 0 -3.449354 2.793572 2.154096

H 0 -0.410611 -0.236111 3.276938

H 0 -2.798592 3.885653 0.073635

H 0 -1.792580 1.668856 2.835963

H 0 -2.347386 1.561672 4.608747

H 0 -0.592519 3.886488 -1.244169

H 0 -3.988981 1.370330 6.362138

H 0 0.923039 0.185018 1.643345

H 0 -3.206608 0.247998 0.667666

H 0 -3.077403 2.417709 -0.849787

H 0 -4.603912 2.236827 0.931881

H 0 2.806443 3.015991 -1.384224

H 0 -4.974154 -0.860245 6.951638

C 0 -1.345352 -1.646999 1.954417

H 0 -0.948235 -2.508921 2.493905

H 0 -2.324665 -1.925250 1.556837

H 0 -0.682875 -1.456390 1.108399

H 0 -6.003983 1.115641 -0.050346

H 0 -6.441353 -0.736399 -0.086869

**E:SS** (-8045.710719)

C -1 7.280845 0.460535 1.969997

C 0 5.938032 0.620362 2.690246

C 0 6.099115 0.488703 4.208349

C 0 5.271904 1.955209 2.331531

H 0 7.151198 0.469897 0.885518

H 0 7.967551 1.276490 2.227402

H 0 5.274288 -0.188101 2.354238

H 0 6.751877 1.278695 4.601296

H 0 5.134112 0.569146 4.718201

H 0 6.542540 -0.475056 4.480917

H 0 4.270765 2.035896 2.768835

H 0 5.865915 2.797479 2.707425

H 0 5.191136 2.096278 1.248488

C -1 2.867943 -2.653791 6.140224

C 0 3.002132 -1.933869 4.789987

C 0 3.040107 -2.928165 3.623375

C 0 1.883205 -0.906220 4.581355

H 0 1.923494 -3.209806 6.191367

H 0 3.682859 -3.369747 6.291057

H 0 3.956815 -1.391338 4.793434

H 0 2.108450 -3.504573 3.576857

H 0 3.160636 -2.405238 2.669499

H 0 3.865158 -3.641080 3.730012

H 0 0.900224 -1.392890 4.581219

H 0 1.878250 -0.151219 5.374450

H 0 2.000078 -0.392497 3.623030

C -1 0.466644 0.416877 10.770611

C 0 0.867342 0.589095 9.300228

C 0 0.487395 -0.647303 8.478481

C 0 0.247576 1.855961 8.698400

H 0 -0.620552 0.303568 10.861662

H 0 0.932302 -0.472987 11.208882

H 0 1.959456 0.700379 9.264224

H 0 -0.594706 -0.815936 8.504181

H 0 0.777526 -0.530859 7.429510

H 0 0.980852 -1.546074 8.865278

H 0 -0.845191 1.792193 8.696747

H 0 0.535499 2.749376 9.263617

H 0 0.571685 1.996492 7.662389

C -1 -3.085941 -4.132846 12.675543

C 0 -3.264924 -5.431612 11.873001

O 0 -4.318405 -5.673673 11.290394

C 0 -3.086947 -2.931758 11.718235

H 0 -3.929836 -4.065817 13.367902

H 0 -2.255813 -2.997017 11.008453

H 0 -4.017210 -2.914879 11.145392

H 0 -2.997189 -1.990975 12.269233

N 0 -2.184158 -6.271728 11.835882

C -1 -2.114161 -7.480190 11.012476

C 0 -1.097957 -7.356541 9.868400

C 0 -1.385985 -6.233194 8.896249

C 0 -2.554320 -6.238287 8.121991

C 0 -0.482016 -5.177398 8.733610

C 0 -2.794215 -5.235376 7.186075

C 0 -0.718249 -4.166130 7.798821

C 0 -1.869574 -4.202173 7.013271

H 0 -3.123769 -7.632380 10.625057

H 0 -1.082000 -8.316657 9.336151

H 0 -0.093373 -7.225692 10.289638

H 0 -3.285258 -7.031612 8.248103

H 0 -1.335527 -5.963166 12.286558

H 0 0.425736 -5.155726 9.331777

H 0 -3.701351 -5.252769 6.595092

H 0 -0.005180 -3.356913 7.683334

H 0 -2.049302 -3.441718 6.261594

C -1 0.152438 -6.400874 -0.187664

C 0 1.387151 -5.590416 -0.610771

C 0 1.334842 -5.212597 -2.095721

C 0 1.539140 -4.334402 0.253979

H 0 0.220058 -6.706766 0.862706

H 0 -0.759292 -5.802696 -0.298966

H 0 2.274609 -6.220618 -0.454357

H 0 0.492005 -4.542119 -2.295263

H 0 2.243123 -4.685860 -2.404596

H 0 1.217285 -6.096675 -2.731936

H 0 0.649703 -3.700492 0.162037

H 0 1.662861 -4.586212 1.312159

H 0 2.402783 -3.735397 -0.052435

C -1 -3.596721 -6.933807 3.872113

C 0 -2.258592 -6.201622 3.810068

S 0 -2.505087 -4.439602 3.352966

C 0 -0.767477 -3.892626 3.414493

H 0 -4.248576 -6.504546 4.637860

H 0 -4.125269 -6.877450 2.915591

H 0 -1.599495 -6.659319 3.064210

H 0 -1.757623 -6.234421 4.782348

H 0 -0.153309 -4.469406 2.719585

H 0 -0.740192 -2.843914 3.113243

H 0 -0.363684 -3.984074 4.426963

C -1 -8.593004 -6.748007 0.713021

C 0 -7.637935 -5.705991 1.233696

C 0 -6.370896 -5.538304 0.669012

C 0 -8.007204 -4.838188 2.271078

C 0 -5.506854 -4.532666 1.099539

C 0 -7.162843 -3.820179 2.705160

C 0 -5.911563 -3.646189 2.102820

O 0 -5.126458 -2.612158 2.512685

H 0 -9.067601 -7.306891 1.526972

H 0 -9.396355 -6.277434 0.133183

H 0 -6.046435 -6.205233 -0.126001

H 0 -8.985683 -4.945198 2.730877

H 0 -4.521121 -4.434379 0.667109

H 0 -7.469961 -3.138309 3.491120

H 0 -4.419186 -2.483865 1.843338

C -1 -3.717831 -4.902605 -2.923981

C 0 -2.525765 -4.022876 -2.538156

C 0 -2.566325 -3.452385 -1.115841

C 0 -3.652229 -2.413717 -0.841097

O 0 -4.431825 -2.102029 -1.779394

O 0 -3.677141 -1.892194 0.326273

H 0 -4.647828 -4.332733 -2.868735

H 0 -2.448861 -3.192141 -3.245114

H 0 -1.605308 -4.608283 -2.641759

H 0 -1.614466 -2.959246 -0.882268

H 0 -2.663202 -4.248927 -0.366320

C -1 2.462906 -1.588925 -6.455080

C 0 2.784539 -1.448014 -4.981685

C 0 1.780322 -1.604506 -4.015760

C 0 4.085065 -1.174945 -4.539979

C 0 2.060006 -1.484985 -2.654685

C 0 4.379158 -1.072173 -3.177167

C 0 3.364885 -1.224182 -2.228582

H 0 3.245110 -1.146437 -7.077781

H 0 2.370440 -2.644096 -6.740066

H 0 0.764572 -1.814633 -4.334767

H 0 4.878425 -1.041145 -5.271323

H 0 1.263350 -1.586016 -1.924879

H 0 5.393137 -0.847055 -2.856852

H 0 3.578140 -1.123856 -1.168864

C -1 2.557011 4.064074 -4.609265

C 0 3.008984 3.709517 -3.188633

C 0 3.683198 2.339874 -3.114304

N 0 4.169679 2.058668 -1.748818

H 0 3.409385 4.106203 -5.296720

H 0 1.856135 3.316935 -4.994236

H 0 2.158046 3.720903 -2.497774

H 0 3.709474 4.475806 -2.824885

H 0 4.499215 2.283879 -3.850345

H 0 2.968448 1.554662 -3.377344

H 0 4.895689 2.725037 -1.487032

H 0 4.623775 1.146999 -1.731553

C -1 -2.421912 0.949306 -5.153818

C 0 -1.687000 0.933153 -3.808441

C 0 -1.692312 -0.481664 -3.224453

C 0 -0.263481 1.489414 -3.930635

H 0 -1.923922 0.288515 -5.874480

H 0 -2.241741 1.584786 -3.119935

H 0 -2.711497 -0.829260 -3.035862

H 0 -1.232525 -1.184392 -3.928775

H 0 -1.131130 -0.533392 -2.287107

H 0 0.337880 0.888228 -4.622623

H 0 -0.275067 2.520704 -4.302118

H 0 0.245058 1.482406 -2.961356

C -1 -8.784044 -2.890610 -3.161080

C 0 -8.907468 -3.839078 -1.971874

S 0 -9.786539 -3.138495 -0.523577

C 0 -8.527137 -1.955778 0.068492

H 0 -9.767569 -2.566436 -3.508481

H 0 -8.216696 -1.992777 -2.899839

H 0 -7.926353 -4.185465 -1.629366

H 0 -9.485646 -4.726378 -2.248305

H 0 -8.554840 -1.022496 -0.495960

H 0 -8.753036 -1.738350 1.113503

H 0 -7.534647 -2.409345 0.025897

C -1 -11.413949 -0.217148 -4.324785

C 0 -10.519581 0.423885 -3.289879

C 0 -9.258802 0.926255 -3.624245

C 0 -10.938303 0.559510 -1.958876

C 0 -8.452545 1.575497 -2.686212

C 0 -10.147088 1.198810 -1.008915

C 0 -8.902367 1.725161 -1.370641

O 0 -8.208332 2.418178 -0.413547

H 0 -11.894712 -1.118643 -3.931454

H 0 -12.212199 0.466329 -4.638245

H 0 -8.898036 0.827435 -4.644539

H 0 -11.898776 0.151459 -1.657697

H 0 -7.496493 1.982645 -2.990884

H 0 -10.476345 1.299727 0.019613

H 0 -7.251221 2.467495 -0.630904

C -1 -5.820098 4.559257 -3.594158

C 0 -4.689998 4.077827 -2.661158

C 0 -5.092545 2.783946 -2.006412

O 0 -5.525659 2.729650 -0.841981

O 0 -5.016483 1.734039 -2.786395

H 0 -6.746551 4.716863 -3.034995

H 0 -4.498708 4.812730 -1.877341

H 0 -3.770395 3.909555 -3.229138

C -1 -9.495882 6.948242 -2.625199

C 0 -9.717966 5.622503 -1.902717

C 0 -10.476838 4.618880 -2.770895

O 0 -8.428661 5.126535 -1.533739

H 0 -8.933455 6.782884 -3.550147

H 0 -10.307406 5.817081 -0.990702

H 0 -11.446599 5.026556 -3.076663

H 0 -9.897724 4.386857 -3.670174

H 0 -10.656907 3.681810 -2.237344

H 0 -8.531851 4.295162 -1.039177

N 0 -7.528742 4.992237 6.187885

C 0 -6.484427 4.022422 5.808568

C 0 -5.205807 4.629545 5.238056

O 0 -4.202699 3.922823 5.112631

C 0 -7.167756 3.146627 4.733893

C 0 -8.655610 3.236427 5.090254

C 0 -8.814411 4.696117 5.532840

H 0 -6.188606 3.435084 6.682694

H 0 -6.766902 2.132954 4.717243

H 0 -6.995685 3.581699 3.742723

H 0 -8.886282 2.570701 5.929026

H 0 -9.313316 2.977083 4.257225

H 0 -8.968164 5.352828 4.665654

H 0 -9.649829 4.845119 6.219278

N 0 -5.293499 5.902591 4.801959

C -1 -4.224811 6.582929 4.089000

C 0 -4.665729 7.069172 2.702895

C 0 -5.224027 5.976807 1.775731

C 0 -5.665865 6.579756 0.436209

C 0 -4.224725 4.833477 1.559689

H 0 -3.403234 5.869459 4.014484

H 0 -3.799780 7.546266 2.224025

H 0 -5.421302 7.858978 2.821257

H 0 -6.115749 5.555463 2.261802

H 0 -6.331202 7.438233 0.583304

H 0 -4.796132 6.931475 -0.134009

H 0 -6.158536 6.389650 4.985448

H 0 -6.205982 5.851810 -0.175759

H 0 -4.006087 4.300774 2.489993

H 0 -4.627182 4.120713 0.835332

H 0 -3.274449 5.216971 1.165752

C -1 -0.160279 5.153853 5.413066

C 0 -0.846997 3.827544 5.081885

S 0 -0.571778 3.233150 3.365617

C 0 1.219205 2.877223 3.428992

H 0 0.929578 5.079391 5.348032

H 0 -0.486466 5.941976 4.727991

H 0 -1.932778 3.921166 5.166017

H 0 -0.534417 3.040110 5.775542

H 0 1.776758 3.743878 3.790853

H 0 1.424369 2.015154 4.068107

H 0 1.546132 2.663139 2.412340

C -1 -0.793811 9.087940 -2.939903

C 0 -0.448597 7.591688 -2.873195

C 0 -1.365291 6.859013 -1.874839

C 0 1.036912 7.396670 -2.541957

C 0 -1.162473 5.340858 -1.805246

H 0 -0.619428 9.569280 -1.968851

H 0 -0.631058 7.157935 -3.868266

H 0 -2.409586 7.069288 -2.142117

H 0 -1.214920 7.290012 -0.874905

H 0 1.670847 7.937135 -3.253275

H 0 1.262175 7.777958 -1.538247

H 0 1.329323 6.343249 -2.570188

H 0 -0.167219 5.080603 -1.432430

H 0 -1.892895 4.882141 -1.130873

H 0 -1.279272 4.875726 -2.791229

C -1 -8.524972 6.609964 7.776927

C 0 -7.353131 5.745647 7.326626

O 0 -6.286467 5.729216 7.930328

H 0 -9.302845 5.992725 8.240043

H 0 -8.152736 7.314801 8.519428

H 0 -1.845157 9.244435 -3.203082

H 0 -0.177951 9.605905 -3.682239

H 0 2.057029 5.036847 -4.633995

H 0 2.881631 -1.943369 6.972852

H 0 0.763994 1.283841 11.370089

H 0 -1.861738 -8.338914 11.645702

H 0 -2.165283 -4.154099 13.270145

H 0 -3.435203 -7.988584 4.115989

H 0 -8.084592 -7.466413 0.063491

H 0 -8.260531 -3.380683 -3.989988

H 0 -3.805091 -5.765482 -2.251642

H 0 -3.606263 -5.287592 -3.942862

H 0 0.037684 -7.304811 -0.795196

H 0 1.513918 -1.102886 -6.701680

H 0 -2.445604 1.955875 -5.586891

H 0 -3.455649 0.610072 -5.037722

H 0 -5.535847 5.507017 -4.059903

H 0 -8.923358 7.634407 -1.995016

H 0 -10.451911 7.415782 -2.878524

H 0 -5.995066 3.828762 -4.389039

H 0 -10.850301 -0.497458 -5.218860

H 0 -8.982828 7.153315 6.945245

H 0 -3.868199 7.431331 4.686231

H 0 -0.408796 5.460413 6.435465

O 0 7.147400 0.846047 -1.737802

O 0 -6.031627 -0.186686 -1.519405

H 0 7.915194 0.428723 -1.308199

H 0 7.019083 1.655112 -1.216284

H 0 -5.418231 0.904534 -2.297523

H 0 -6.907997 -0.406736 -1.861390

C -1 5.111279 1.407014 8.853953

C 0 4.121778 2.450360 8.317925

C 0 4.791716 3.820418 8.165463

C 0 3.505080 1.994652 6.990225

H 0 4.623986 0.436844 8.998497

H 0 5.938939 1.261413 8.149446

H 0 3.308809 2.550695 9.049915

H 0 5.613000 3.770349 7.440009

H 0 4.079866 4.573345 7.810585

H 0 5.207767 4.170845 9.116177

H 0 2.759869 2.710956 6.628583

H 0 4.277315 1.900169 6.219170

H 0 3.012484 1.022320 7.092774

C -1 -7.140131 -6.831086 9.563923

C 0 -6.769260 -5.940367 8.398294

C 0 -6.222028 -4.669212 8.628563

C 0 -6.982387 -6.337547 7.071877

C 0 -5.934432 -3.810733 7.567664

C 0 -6.675835 -5.489862 6.003681

C 0 -6.157271 -4.217719 6.249583

H 0 -6.354729 -6.810237 10.325367

H 0 -8.068290 -6.488433 10.036717

H 0 -6.000012 -4.373703 9.649641

H 0 -7.397200 -7.322719 6.872826

H 0 -5.525962 -2.825554 7.769615

H 0 -6.849169 -5.810585 4.980779

H 0 -5.911063 -3.567077 5.416169

H 0 -7.296387 -7.865149 9.242586

H 0 5.538046 1.717768 9.813600

H 0 7.771969 -0.480865 2.235754

H 0 -5.427694 -1.022803 -1.593629

C -1 6.647544 5.725761 0.043033

C 0 7.105165 4.566521 -0.810131

O 0 6.838858 3.397929 -0.493605

H 0 7.059150 5.601407 1.047568

N 0 7.820285 4.865275 -1.918725

C 0 8.345131 3.856437 -2.827513

C 0 9.729323 3.305759 -2.470382

O 0 10.556107 3.079392 -3.346757

H 0 7.663819 2.998582 -2.837447

H 0 8.080147 5.826818 -2.078565

N 0 9.944437 3.067227 -1.141820

C -1 11.056571 2.194044 -0.766056

C 0 10.606777 0.761317 -0.970480

O 0 9.608307 0.350460 -0.358991

H 0 11.930434 2.476795 -1.347853

H 0 9.113962 2.964768 -0.566912

N 0 11.271408 0.001962 -1.850025

C -1 10.757114 -1.336919 -2.119973

C 0 11.772380 -1.919441 -3.110425

C 0 12.266132 -0.678222 -3.869873

C 0 12.347594 0.398532 -2.779147

H 0 11.330305 -2.676832 -3.761451

H 0 12.602856 -2.384983 -2.568042

H 0 11.528358 -0.378070 -4.621426

H 0 13.223015 -0.827409 -4.375419

H 0 13.318412 0.371395 -2.268073

H 0 12.164413 1.401469 -3.166216

H 0 9.753977 -1.263840 -2.558152

H 0 5.558769 5.681330 0.131889

H 0 6.938011 6.701956 -0.351052

H 0 8.409810 4.269827 -3.833912

H 0 11.270575 2.340861 0.295415

H 0 10.664870 -1.905099 -1.191704

N 0 -4.616453 0.559489 0.939870

C 0 -3.506633 1.083488 8.899042

C 0 -3.398009 -0.307920 8.865874

C 0 -3.290747 1.230915 1.118315

C 0 -2.461054 1.192060 -0.178175

C 0 -3.485661 -0.991792 7.651082

C 0 -3.677913 -0.288307 6.456007

C 0 -1.011159 0.852495 0.088637

C 0 -3.776971 -1.010303 5.111436

C 0 -0.000349 1.799557 -0.101227

C 0 1.331556 1.496527 0.184726

C 0 -5.119900 -0.610022 4.542126

C 0 1.652224 0.227088 0.696039

C 0 -3.779314 1.109129 6.496032

C 0 -3.692855 1.791217 7.709163

C 0 0.656607 -0.728894 0.880493

C 0 -0.667809 -0.418838 0.572082

O 0 -5.302086 0.228042 3.674279

O 0 2.948029 -0.054617 1.043813

O 0 2.315624 2.442593 0.098825

H 0 -3.244436 -0.867309 9.783312

H 0 -3.391715 -2.072758 7.635398

H 0 3.425932 0.791495 1.010835

H 0 -1.448819 -1.158557 0.723885

H 0 -2.784031 0.696576 1.916356

H 0 -2.531598 2.153925 -0.692882

H 0 -5.988120 -1.081083 5.044440

H 0 -3.933326 1.684815 5.589715

H 0 -0.235495 2.801611 -0.446347

H 0 -3.773395 2.873653 7.709311

H 0 0.927870 -1.697012 1.285245

H 0 -5.195482 1.048433 0.243561

H 0 -2.890656 0.443745 -0.851267

H 0 -3.465599 2.248596 1.463532

H 0 -5.098906 0.517214 1.846858

H 0 2.999547 2.224973 -0.636330

H 0 -4.442585 -0.434432 0.616594

H 0 -3.442944 1.612191 9.845373

H 0 -3.824831 -2.090449 5.291891

C 0 -2.597125 -0.690912 4.190826

H 0 -1.668893 -1.050178 4.642562

H 0 -2.719152 -1.179824 3.220250

H 0 -2.491445 0.385440 4.038154

**Int1S** (-8045.709972)

C -1 7.283383 0.459626 1.969566

C 0 5.911553 0.637616 2.630648

C 0 5.939716 0.228126 4.107622

C 0 5.410147 2.080070 2.478544

H 0 7.236687 0.664162 0.897671

H 0 8.025452 1.138928 2.407134

H 0 5.198473 -0.017673 2.114707

H 0 6.636346 0.857194 4.675912

H 0 4.949964 0.328459 4.565322

H 0 6.259005 -0.812949 4.226806

H 0 4.397103 2.195141 2.882149

H 0 6.057931 2.775436 3.027770

H 0 5.414871 2.402849 1.431638

C -1 2.868849 -2.654461 6.139395

C 0 2.291372 -1.359749 5.556201

C 0 2.384442 -1.354432 4.027297

C 0 0.844326 -1.147407 6.015708

H 0 2.294707 -3.523629 5.796344

H 0 3.910401 -2.797987 5.832823

H 0 2.891865 -0.520842 5.936555

H 0 1.777457 -2.164311 3.603962

H 0 2.021733 -0.415416 3.602003

H 0 3.413795 -1.495498 3.684204

H 0 0.205426 -1.965676 5.664822

H 0 0.771793 -1.115250 7.107358

H 0 0.432072 -0.212781 5.623807

C -1 0.465806 0.417408 10.771955

C 0 -0.565624 0.303843 9.638507

C 0 -1.995690 0.468503 10.165814

C 0 -0.283649 1.318784 8.523598

H 0 0.428205 1.412374 11.230875

H 0 0.272618 -0.320863 11.557679

H 0 -0.483040 -0.704936 9.206731

H 0 -2.135681 1.466726 10.598124

H 0 -2.734609 0.351526 9.367190

H 0 -2.219388 -0.267130 10.943860

H 0 -0.367427 2.342503 8.908510

H 0 0.721679 1.196266 8.110595

H 0 -0.998941 1.213053 7.702567

C -1 -3.086023 -4.133050 12.675816

C 0 -3.265036 -5.455073 11.912052

O 0 -4.331575 -5.730319 11.369472

C 0 -3.146278 -2.951358 11.698738

H 0 -3.906375 -4.069935 13.396642

H 0 -2.331872 -3.001785 10.969231

H 0 -4.089926 -2.963901 11.148424

H 0 -3.072520 -2.002251 12.236618

N 0 -2.176949 -6.286161 11.859187

C -1 -2.114213 -7.479759 11.012314

C 0 -1.056103 -7.346812 9.903519

C 0 -1.263350 -6.149321 9.003576

C 0 -2.296862 -6.133602 8.058269

C 0 -0.434889 -5.025675 9.104611

C 0 -2.482889 -5.034946 7.221371

C 0 -0.617596 -3.919688 8.271228

C 0 -1.639783 -3.926083 7.322152

H 0 -3.113466 -7.591197 10.586854

H 0 -1.073422 -8.271647 9.313967

H 0 -0.060283 -7.291899 10.361045

H 0 -2.968001 -6.984296 7.978247

H 0 -1.315640 -5.956934 12.269563

H 0 0.372466 -5.022979 9.832993

H 0 -3.292662 -5.031034 6.502751

H 0 0.044469 -3.063464 8.356458

H 0 -1.786486 -3.076595 6.664079

C -1 0.153500 -6.401155 -0.187210

C 0 1.085708 -5.391274 -0.869213

C 0 0.599874 -5.037745 -2.280567

C 0 1.236850 -4.124869 -0.019101

H 0 0.551049 -6.713875 0.784999

H 0 -0.834522 -5.959207 -0.012987

H 0 2.077055 -5.857353 -0.961236

H 0 -0.384203 -4.557374 -2.242097

H 0 1.286051 -4.341973 -2.773236

H 0 0.509691 -5.931593 -2.907271

H 0 0.272017 -3.611770 0.076199

H 0 1.595877 -4.357026 0.990256

H 0 1.939133 -3.421307 -0.475538

C -1 -3.596313 -6.934479 3.871748

C 0 -2.358315 -6.043813 3.782893

S 0 -2.820442 -4.300208 3.418415

C 0 -1.146431 -3.580949 3.308736

H 0 -4.267469 -6.605489 4.670836

H 0 -4.162911 -6.923737 2.935895

H 0 -1.689207 -6.386248 2.986276

H 0 -1.807167 -6.057364 4.728069

H 0 -0.566453 -4.056445 2.513435

H 0 -1.252908 -2.519674 3.073698

H 0 -0.618385 -3.682080 4.260656

C -1 -8.591536 -6.747411 0.712402

C 0 -8.182927 -5.647785 1.670304

C 0 -6.867233 -5.181161 1.746350

C 0 -9.137351 -4.998607 2.468268

C 0 -6.512535 -4.072811 2.525611

C 0 -8.804042 -3.907930 3.262412

C 0 -7.494270 -3.410415 3.267570

O 0 -7.253908 -2.298500 4.021491

H 0 -9.220532 -7.499202 1.201276

H 0 -9.170087 -6.324623 -0.116647

H 0 -6.093648 -5.685774 1.176969

H 0 -10.169877 -5.334879 2.440697

H 0 -5.487417 -3.716721 2.531675

H 0 -9.552760 -3.399983 3.860855

H 0 -6.446742 -1.832411 3.742427

C -1 -3.718089 -4.902871 -2.923630

C 0 -3.195402 -4.636482 -1.511676

C 0 -4.293838 -4.332345 -0.471348

C 0 -4.832098 -2.927413 -0.577428

O 0 -5.547875 -2.707949 -1.662684

O 0 -4.585775 -2.069816 0.273219

H 0 -4.258320 -4.039638 -3.317951

H 0 -2.470673 -3.812596 -1.522790

H 0 -2.647322 -5.516469 -1.159811

H 0 -3.902670 -4.450429 0.541585

H 0 -5.123941 -5.030694 -0.615245

C -1 2.461237 -1.592605 -6.453614

C 0 2.730026 -1.384015 -4.977438

C 0 1.702354 -1.541429 -4.036908

C 0 4.006355 -1.047610 -4.506850

C 0 1.939397 -1.372184 -2.672208

C 0 4.258968 -0.891159 -3.140941

C 0 3.222642 -1.057575 -2.218145

H 0 3.213422 -1.092609 -7.070126

H 0 2.478996 -2.658521 -6.710873

H 0 0.702034 -1.790950 -4.377913

H 0 4.815165 -0.906178 -5.219531

H 0 1.124237 -1.474333 -1.963293

H 0 5.253561 -0.612967 -2.799849

H 0 3.405476 -0.931152 -1.155076

C -1 2.557460 4.062341 -4.609792

C 0 2.938867 3.850432 -3.141631

C 0 3.429990 2.431020 -2.869686

N 0 3.821593 2.264700 -1.455197

H 0 3.413910 3.890974 -5.271091

H 0 1.762918 3.370354 -4.908456

H 0 2.080864 4.054635 -2.490322

H 0 3.722765 4.567024 -2.855888

H 0 4.258612 2.179980 -3.547055

H 0 2.630192 1.713838 -3.076386

H 0 4.654927 2.817250 -1.250710

H 0 4.087639 1.294248 -1.298294

C -1 -2.423132 0.948925 -5.154382

C 0 -1.934615 0.788427 -3.708223

C 0 -1.997973 -0.683002 -3.273102

C 0 -0.523726 1.369171 -3.543705

H 0 -1.765673 0.406755 -5.845153

H 0 -2.614332 1.353260 -3.055224

H 0 -3.034027 -1.037868 -3.228171

H 0 -1.460532 -1.323548 -3.982438

H 0 -1.543211 -0.825922 -2.287942

H 0 0.190160 0.853821 -4.196197

H 0 -0.506681 2.434823 -3.801250

H 0 -0.165889 1.266573 -2.515480

C -1 -8.783551 -2.890551 -3.160661

C 0 -8.967638 -3.932286 -2.064648

S 0 -9.915923 -3.327888 -0.617036

C 0 -8.624485 -2.304318 0.162372

H 0 -9.747032 -2.560669 -3.554373

H 0 -8.259751 -2.008460 -2.788354

H 0 -7.999662 -4.295685 -1.704132

H 0 -9.526075 -4.796071 -2.438983

H 0 -8.324532 -1.478255 -0.483186

H 0 -9.045761 -1.895945 1.081212

H 0 -7.765673 -2.923278 0.421123

C -1 -11.414053 -0.216740 -4.324928

C 0 -10.538925 0.318621 -3.213104

C 0 -9.433486 1.130565 -3.476551

C 0 -10.822155 0.037972 -1.867563

C 0 -8.639877 1.654568 -2.449609

C 0 -10.048938 0.552621 -0.833581

C 0 -8.943496 1.372558 -1.105934

O 0 -8.219671 1.837881 -0.060513

H 0 -11.771894 -1.225958 -4.097905

H 0 -12.299005 0.413373 -4.478390

H 0 -9.178704 1.370898 -4.505848

H 0 -11.655893 -0.612603 -1.622517

H 0 -7.817371 2.319424 -2.684800

H 0 -10.280002 0.323040 0.200657

H 0 -7.545810 2.509511 -0.361529

C -1 -5.820507 4.558468 -3.595519

C 0 -4.817017 3.931015 -2.627807

C 0 -5.498306 3.142521 -1.520201

O 0 -6.487107 3.645538 -0.917271

O 0 -5.031215 1.985195 -1.257112

H 0 -6.485612 5.252451 -3.079206

H 0 -4.216479 4.717324 -2.154109

H 0 -4.120924 3.264015 -3.142607

C -1 -9.496080 6.948669 -2.624139

C 0 -9.080579 6.208925 -1.354394

C 0 -9.690583 4.812129 -1.291721

O 0 -7.657088 6.183107 -1.311932

H 0 -9.198916 6.372853 -3.507042

H 0 -9.442323 6.784472 -0.484249

H 0 -10.783692 4.866516 -1.260819

H 0 -9.408702 4.228073 -2.172072

H 0 -9.353589 4.265512 -0.406144

H 0 -7.336150 5.289265 -1.085683

N 0 -7.649278 5.068883 6.040004

C 0 -6.683819 4.048979 5.583212

C 0 -5.362664 4.596864 5.046901

O 0 -4.393930 3.838766 4.924067

C 0 -7.443890 3.287362 4.473501

C 0 -8.917516 3.474653 4.851981

C 0 -8.957668 4.913997 5.378985

H 0 -6.426452 3.391934 6.416305

H 0 -7.137738 2.241930 4.418744

H 0 -7.244111 3.746485 3.498808

H 0 -9.190394 2.781382 5.654712

H 0 -9.598366 3.314104 4.013050

H 0 -9.061771 5.630950 4.553627

H 0 -9.775704 5.087268 6.080092

N 0 -5.374532 5.887559 4.653241

C -1 -4.224714 6.582509 4.089191

C 0 -4.427253 7.047055 2.645148

C 0 -4.749412 5.955323 1.610599

C 0 -4.853022 6.594523 0.222486

C 0 -3.727971 4.810181 1.612498

H 0 -3.387422 5.885354 4.155564

H 0 -3.505152 7.563026 2.342372

H 0 -5.220992 7.806235 2.615049

H 0 -5.731500 5.528783 1.858604

H 0 -5.622247 7.370443 0.184965

H 0 -3.893715 7.042011 -0.065891

H 0 -6.217115 6.408365 4.850520

H 0 -5.125662 5.850278 -0.524639

H 0 -2.716357 5.181963 1.408171

H 0 -3.698799 4.280535 2.567937

H 0 -3.978758 4.085202 0.830276

C -1 -0.160192 5.154542 5.413186

C 0 -1.059521 3.929487 5.584358

S 0 -1.253457 2.875370 4.092159

C 0 0.457366 2.285530 3.842208

H 0 0.869210 4.867377 5.178072

H 0 -0.524312 5.797212 4.605866

H 0 -2.087718 4.223023 5.813011

H 0 -0.698475 3.300663 6.404917

H 0 0.889733 1.938045 4.785070

H 0 0.400237 1.447129 3.146310

H 0 1.094428 3.054858 3.400661

C -1 -0.793677 9.088379 -2.939512

C 0 -0.434618 7.598265 -2.815807

C 0 -1.353185 6.893968 -1.799315

C 0 1.049713 7.427793 -2.467722

C 0 -1.145934 5.379850 -1.675252

H 0 -0.632533 9.605545 -1.985880

H 0 -0.606270 7.126871 -3.795450

H 0 -2.395660 7.088415 -2.082026

H 0 -1.215458 7.362289 -0.814066

H 0 1.685593 7.942391 -3.196525

H 0 1.265619 7.851994 -1.479128

H 0 1.347308 6.375802 -2.449943

H 0 -0.167651 5.125719 -1.255011

H 0 -1.903607 4.940354 -1.018518

H 0 -1.228885 4.886849 -2.651068

C -1 -8.525052 6.610124 7.777037

C 0 -7.419146 5.706049 7.238271

O 0 -6.354838 5.562681 7.831886

H 0 -9.343384 6.010476 8.190634

H 0 -8.098538 7.216205 8.575262

H 0 -1.844244 9.222738 -3.217380

H 0 -0.176541 9.584281 -3.696134

H 0 2.196384 5.080755 -4.781531

H 0 2.836285 -2.649237 7.234625

H 0 1.484567 0.260708 10.402002

H 0 -1.902655 -8.362388 11.627261

H 0 -2.145231 -4.122989 13.238455

H 0 -3.296915 -7.966121 4.080133

H 0 -7.718379 -7.257199 0.294114

H 0 -8.189104 -3.301846 -3.984807

H 0 -4.405921 -5.755717 -2.926691

H 0 -2.891860 -5.134670 -3.603359

H 0 0.015438 -7.299036 -0.798868

H 0 1.476804 -1.206762 -6.735656

H 0 -2.429934 2.001498 -5.457809

H 0 -3.437771 0.555500 -5.278734

H 0 -5.298474 5.102419 -4.388873

H 0 -9.002951 7.923877 -2.672254

H 0 -10.580140 7.103992 -2.656603

H 0 -6.432879 3.786701 -4.075843

H 0 -10.871669 -0.256096 -5.274142

H 0 -8.944918 7.257273 7.001741

H 0 -3.987600 7.443610 4.725770

H 0 -0.140584 5.739009 6.339872

O 0 7.165440 0.890116 -1.874423

O 0 -6.182279 -0.278172 -2.180843

H 0 7.915002 0.516545 -1.377969

H 0 6.909154 1.670177 -1.357393

H 0 -5.675680 0.530648 -1.940237

H 0 -7.102019 0.017054 -2.274329

C -1 5.112524 1.407644 8.853690

C 0 3.672051 1.788412 8.489041

C 0 3.111444 2.817927 9.476686

C 0 3.578470 2.305962 7.048544

H 0 5.510479 0.651536 8.168496

H 0 5.769578 2.284365 8.800467

H 0 3.056411 0.879967 8.558437

H 0 3.704769 3.739925 9.448456

H 0 2.076307 3.078574 9.236868

H 0 3.131683 2.438611 10.503561

H 0 2.542214 2.533561 6.774446

H 0 4.163231 3.225915 6.927404

H 0 3.963398 1.572976 6.332481

C -1 -7.140040 -6.831047 9.564046

C 0 -6.686456 -5.933085 8.436711

C 0 -5.951842 -4.770618 8.709739

C 0 -7.004455 -6.221881 7.103319

C 0 -5.567066 -3.914112 7.678837

C 0 -6.617956 -5.369428 6.067224

C 0 -5.899671 -4.207399 6.354595

H 0 -6.383665 -6.873520 10.352213

H 0 -8.065762 -6.453593 10.016896

H 0 -5.664320 -4.559348 9.734681

H 0 -7.567237 -7.123809 6.874595

H 0 -4.986671 -3.025570 7.910584

H 0 -6.882633 -5.599658 5.040367

H 0 -5.608071 -3.544085 5.549426

H 0 -7.342014 -7.846328 9.209039

H 0 5.172434 1.007863 9.871478

H 0 7.659214 -0.561349 2.090353

H 0 -5.806893 -1.731258 -1.767283

C -1 6.648272 5.726951 0.042444

C 0 7.044597 4.579619 -0.857482

O 0 6.642440 3.427478 -0.638874

H 0 6.947342 5.483831 1.065070

N 0 7.861574 4.876955 -1.893845

C 0 8.377321 3.888539 -2.828055

C 0 9.752663 3.322087 -2.467778

O 0 10.584193 3.089468 -3.337027

H 0 7.689081 3.037272 -2.858807

H 0 8.215836 5.819024 -1.964788

N 0 9.958466 3.082057 -1.136019

C -1 11.059176 2.194980 -0.759663

C 0 10.597968 0.768279 -0.979892

O 0 9.580033 0.368902 -0.392747

H 0 11.939336 2.475516 -1.332768

H 0 9.122333 2.966483 -0.573349

N 0 11.276752 0.002207 -1.841971

C -1 10.757302 -1.332561 -2.124990

C 0 11.783872 -1.918585 -3.101478

C 0 12.297332 -0.678363 -3.849236

C 0 12.369370 0.394447 -2.753945

H 0 11.346633 -2.670669 -3.761828

H 0 12.602847 -2.391610 -2.548171

H 0 11.573002 -0.370883 -4.610765

H 0 13.260806 -0.832291 -4.340608

H 0 13.332249 0.360172 -2.228625

H 0 12.197992 1.399439 -3.141164

H 0 9.761389 -1.250219 -2.577379

H 0 5.558532 5.815457 0.036291

H 0 7.087363 6.683931 -0.247414

H 0 8.452697 4.323061 -3.825271

H 0 11.267323 2.330840 0.304526

H 0 10.647313 -1.903250 -1.200131

N 0 -4.892951 0.559228 0.961639

C 0 -6.106916 1.810864 8.976104

C 0 -4.997525 2.346836 8.319771

C 0 -3.442432 0.734561 1.291843

C 0 -2.601706 0.638564 0.017245

C 0 -4.385790 1.635550 7.287488

C 0 -4.884765 0.384983 6.896888

C 0 -1.119881 0.598318 0.293743

C 0 -4.288154 -0.361437 5.711867

C 0 -0.331933 1.748651 0.154780

C 0 1.047364 1.707446 0.359244

C 0 -4.760238 0.383980 4.489758

C 0 1.641445 0.492638 0.754277

C 0 -6.008789 -0.138901 7.547855

C 0 -6.607839 0.565615 8.591970

C 0 0.871038 -0.657157 0.897520

C 0 -0.503209 -0.604312 0.659451

O 0 -5.481066 -0.102922 3.624103

O 0 2.989341 0.438291 1.001773

O 0 1.821671 2.823998 0.236893

H 0 -4.634693 3.333989 8.584200

H 0 -3.547637 2.077079 6.761413

H 0 3.298858 1.355898 1.072135

H 0 -1.102399 -1.506922 0.747938

H 0 -3.174386 -0.057704 1.993261

H 0 -2.852824 1.475092 -0.636085

H 0 -4.443891 1.441265 4.420941

H 0 -6.413103 -1.096591 7.233036

H 0 -0.777708 2.693168 -0.140551

H 0 -7.471246 0.143626 9.097195

H 0 1.357111 -1.578434 1.194332

H 0 -5.177383 1.241816 0.206766

H 0 -2.899567 -0.273276 -0.509146

H 0 -3.313266 1.699247 1.788453

H 0 -5.463657 0.626283 1.812474

H 0 2.582753 2.641117 -0.421411

H 0 -5.012562 -0.401683 0.579769

H 0 -6.583712 2.370003 9.775111

H 0 -4.705774 -1.371080 5.667045

C 0 -2.750478 -0.436482 5.721525

H 0 -2.411710 -0.830189 6.683075

H 0 -2.397518 -1.104447 4.930350

H 0 -2.292004 0.542865 5.559172

**TS1S** (-8045.698385)

C -1 7.280817 0.460470 1.969991

C 0 5.964668 0.643224 2.731262

C 0 6.170454 0.522075 4.244611

C 0 5.306361 1.983821 2.379467

H 0 7.117269 0.458923 0.889724

H 0 7.984985 1.270538 2.195459

H 0 5.281832 -0.160187 2.424439

H 0 6.842973 1.307850 4.611756

H 0 5.221745 0.617239 4.781434

H 0 6.611155 -0.444498 4.511623

H 0 4.314540 2.079911 2.834340

H 0 5.919199 2.820374 2.737621

H 0 5.204519 2.120494 1.297584

C -1 2.868133 -2.653711 6.140067

C 0 3.004479 -1.896400 4.813559

C 0 3.097138 -2.858663 3.623267

C 0 1.854452 -0.903399 4.617114

H 0 1.944852 -3.247098 6.155431

H 0 3.706012 -3.341903 6.296459

H 0 3.939860 -1.321610 4.849281

H 0 2.178041 -3.451559 3.535620

H 0 3.236528 -2.309227 2.687480

H 0 3.932476 -3.558386 3.737202

H 0 0.889762 -1.423443 4.568536

H 0 1.803044 -0.185484 5.441832

H 0 1.975517 -0.343589 3.686249

C -1 0.466595 0.417014 10.770880

C 0 0.764784 0.208913 9.278979

C 0 0.222123 -1.136372 8.783426

C 0 0.198621 1.357032 8.434626

H 0 -0.615863 0.437766 10.945325

H 0 0.889411 -0.390437 11.378634

H 0 1.857661 0.201507 9.155917

H 0 -0.868635 -1.173912 8.873522

H 0 0.469309 -1.289593 7.727643

H 0 0.638969 -1.975560 9.351761

H 0 -0.891684 1.403940 8.519422

H 0 0.606007 2.324183 8.749738

H 0 0.441250 1.220859 7.375834

C -1 -3.085947 -4.132902 12.675631

C 0 -3.271380 -5.445963 11.897128

O 0 -4.341992 -5.718585 11.361663

C 0 -3.086014 -2.951255 11.694354

H 0 -3.928940 -4.051331 13.367440

H 0 -2.252840 -3.025907 10.988149

H 0 -4.014020 -2.949210 11.117139

H 0 -3.000813 -1.999450 12.226837

N 0 -2.173651 -6.262036 11.822513

C -1 -2.114058 -7.480052 11.012443

C 0 -1.186771 -7.337549 9.796330

C 0 -1.592190 -6.260153 8.812611

C 0 -2.841438 -6.301063 8.178749

C 0 -0.713393 -5.221545 8.485048

C 0 -3.189267 -5.350682 7.222375

C 0 -1.055702 -4.265622 7.525010

C 0 -2.292008 -4.334423 6.882583

H 0 -3.141191 -7.679196 10.701560

H 0 -1.159250 -8.311251 9.288825

H 0 -0.163229 -7.147861 10.142866

H 0 -3.553172 -7.081297 8.431000

H 0 -1.312392 -5.927294 12.228275

H 0 0.257851 -5.170359 8.971171

H 0 -4.158005 -5.398916 6.742328

H 0 -0.355883 -3.473587 7.280320

H 0 -2.551657 -3.622935 6.105241

C -1 0.152351 -6.400878 -0.187594

C 0 1.363948 -5.572301 -0.639841

C 0 1.317938 -5.278613 -2.143977

C 0 1.467992 -4.268232 0.157784

H 0 0.201780 -6.625282 0.883788

H 0 -0.780756 -5.854403 -0.365754

H 0 2.268993 -6.164204 -0.441230

H 0 0.453667 -4.651829 -2.390494

H 0 2.210090 -4.735030 -2.470246

H 0 1.241945 -6.199650 -2.732171

H 0 0.557821 -3.670016 0.037052

H 0 1.600094 -4.461759 1.227209

H 0 2.309958 -3.655455 -0.179335

C -1 -3.596753 -6.933917 3.872070

C 0 -2.271288 -6.181804 3.780740

S 0 -2.533157 -4.473072 3.156969

C 0 -0.832437 -3.843823 3.345760

H 0 -4.271585 -6.460895 4.590228

H 0 -4.106016 -6.963840 2.903788

H 0 -1.578303 -6.694309 3.104369

H 0 -1.805046 -6.112703 4.767827

H 0 -0.126931 -4.433426 2.756021

H 0 -0.818335 -2.814626 2.983124

H 0 -0.529857 -3.854645 4.396731

C -1 -8.592990 -6.747968 0.713027

C 0 -7.679556 -5.666792 1.234140

C 0 -6.396243 -5.480371 0.712884

C 0 -8.103116 -4.786781 2.240929

C 0 -5.567401 -4.448413 1.154865

C 0 -7.295820 -3.743396 2.684183

C 0 -6.024739 -3.552173 2.127203

O 0 -5.284653 -2.495833 2.564308

H 0 -9.007880 -7.354164 1.525853

H 0 -9.439012 -6.307965 0.171955

H 0 -6.027758 -6.157086 -0.054358

H 0 -9.095200 -4.905061 2.667644

H 0 -4.561468 -4.351275 0.770644

H 0 -7.646085 -3.050115 3.441405

H 0 -4.579616 -2.305396 1.916568

C -1 -3.717699 -4.902600 -2.924002

C 0 -2.538676 -3.973702 -2.621661

C 0 -2.472233 -3.443777 -1.183903

C 0 -3.551622 -2.475642 -0.748679

O 0 -4.444878 -2.196807 -1.679254

O 0 -3.571049 -1.982080 0.379536

H 0 -4.671080 -4.386956 -2.789939

H 0 -2.553815 -3.126829 -3.313120

H 0 -1.604415 -4.512090 -2.814247

H 0 -1.526441 -2.914057 -1.017171

H 0 -2.474093 -4.259903 -0.449800

C -1 2.462884 -1.588944 -6.455049

C 0 2.766731 -1.440080 -4.978935

C 0 1.744489 -1.557669 -4.026562

C 0 4.067982 -1.199962 -4.520467

C 0 2.008060 -1.435757 -2.662010

C 0 4.345058 -1.093667 -3.154488

C 0 3.314008 -1.209764 -2.218756

H 0 3.266277 -1.175424 -7.070939

H 0 2.344933 -2.643956 -6.730071

H 0 0.728953 -1.743071 -4.362943

H 0 4.875269 -1.094956 -5.241091

H 0 1.199460 -1.507487 -1.941563

H 0 5.360650 -0.893542 -2.822340

H 0 3.516519 -1.108890 -1.156793

C -1 2.556977 4.064001 -4.609282

C 0 3.007576 3.701809 -3.189691

C 0 3.709494 2.345346 -3.127104

N 0 4.192230 2.055520 -1.763547

H 0 3.411724 4.125114 -5.292149

H 0 1.868294 3.310492 -5.004195

H 0 2.151902 3.687925 -2.504613

H 0 3.689788 4.477864 -2.812485

H 0 4.530507 2.317083 -3.859891

H 0 3.012549 1.549485 -3.406026

H 0 4.902402 2.732423 -1.485869

H 0 4.664552 1.153120 -1.753756

C -1 -2.421931 0.949308 -5.153834

C 0 -1.762067 0.989515 -3.769422

C 0 -1.757810 -0.407501 -3.142935

C 0 -0.348626 1.580793 -3.831949

H 0 -1.857932 0.296675 -5.831387

H 0 -2.370259 1.637995 -3.123547

H 0 -2.778284 -0.767961 -2.987897

H 0 -1.250768 -1.123812 -3.798554

H 0 -1.238126 -0.412826 -2.181114

H 0 0.302671 0.972872 -4.470755

H 0 -0.363513 2.599471 -4.235589

H 0 0.106948 1.615395 -2.837404

C -1 -8.784114 -2.890660 -3.161072

C 0 -8.930088 -3.849554 -1.982305

S 0 -10.036380 -3.242204 -0.651304

C 0 -8.957651 -1.987464 0.117942

H 0 -9.752899 -2.691287 -3.625602

H 0 -8.374300 -1.928247 -2.840325

H 0 -7.961398 -4.085971 -1.528551

H 0 -9.372558 -4.796166 -2.307545

H 0 -8.862822 -1.089762 -0.494734

H 0 -9.427214 -1.706753 1.062742

H 0 -7.976442 -2.410235 0.340508

C -1 -11.414034 -0.217205 -4.324855

C 0 -10.431970 0.417414 -3.380405

C 0 -9.133139 0.721977 -3.790905

C 0 -10.798589 0.736523 -2.065928

C 0 -8.227177 1.353423 -2.939869

C 0 -9.911324 1.366297 -1.199300

C 0 -8.627712 1.692013 -1.643661

O 0 -7.813697 2.383202 -0.783724

H 0 -11.944795 -1.048243 -3.848404

H 0 -12.171269 0.502570 -4.658095

H 0 -8.820635 0.472802 -4.801184

H 0 -11.794686 0.483543 -1.714331

H 0 -7.230345 1.609854 -3.279449

H 0 -10.195269 1.616815 -0.182971

H 0 -6.906213 2.344766 -1.132772

C -1 -5.819904 4.559360 -3.593999

C 0 -4.504686 4.091837 -2.941910

C 0 -4.771901 3.054649 -1.876971

O 0 -4.500370 3.383009 -0.653752

O 0 -5.279612 1.956516 -2.193043

H 0 -6.518204 4.961096 -2.855382

H 0 -3.958170 4.928526 -2.505025

H 0 -3.863828 3.621931 -3.696560

C -1 -9.495884 6.948221 -2.625185

C 0 -9.574757 5.505090 -2.141964

C 0 -9.877623 4.546667 -3.294128

O 0 -8.323220 5.223966 -1.513510

H 0 -8.711241 7.046916 -3.382420

H 0 -10.385036 5.425945 -1.397279

H 0 -10.808012 4.834318 -3.796026

H 0 -9.066810 4.574355 -4.029262

H 0 -9.989806 3.518366 -2.947176

H 0 -8.325695 4.324838 -1.145295

N 0 -7.551529 4.971587 6.194853

C 0 -6.490296 4.022380 5.809073

C 0 -5.200794 4.668818 5.306064

O 0 -4.123653 4.098594 5.465081

C 0 -7.159682 3.171702 4.711940

C 0 -8.635760 3.166175 5.126193

C 0 -8.861956 4.596606 5.635429

H 0 -6.200717 3.412314 6.670039

H 0 -6.710753 2.181309 4.627804

H 0 -7.043992 3.673697 3.744325

H 0 -8.794533 2.453884 5.942995

H 0 -9.312569 2.902280 4.309963

H 0 -9.140125 5.269295 4.812787

H 0 -9.646947 4.653897 6.393105

N 0 -5.352477 5.810641 4.593761

C -1 -4.224788 6.582937 4.088890

C 0 -4.462771 7.095889 2.668839

C 0 -4.406455 6.022756 1.570598

C 0 -4.906028 6.599267 0.240646

C 0 -2.992122 5.446187 1.425530

H 0 -3.355375 5.926283 4.139934

H 0 -3.708326 7.864281 2.451732

H 0 -5.434078 7.610498 2.635288

H 0 -5.081526 5.204661 1.860100

H 0 -5.940200 6.952727 0.314637

H 0 -4.280661 7.444203 -0.074906

H 0 -6.255531 6.260607 4.649839

H 0 -4.873552 5.839778 -0.541620

H 0 -2.629400 4.977737 2.344542

H 0 -2.973744 4.686067 0.642766

H 0 -2.281981 6.235329 1.146289

C -1 -0.160298 5.153792 5.413086

C 0 -0.978088 3.889252 5.665484

S 0 -1.394779 2.937712 4.155978

C 0 0.241033 2.260528 3.712457

H 0 0.809729 4.925804 4.959929

H 0 -0.693571 5.832998 4.741502

H 0 -1.953104 4.134129 6.088435

H 0 -0.465324 3.216563 6.360171

H 0 0.922551 3.026872 3.335972

H 0 0.686371 1.757067 4.573701

H 0 0.079863 1.525167 2.922867

C -1 -0.793856 9.087972 -2.939871

C 0 -0.390883 7.605142 -2.928272

C 0 -1.282951 6.799799 -1.965207

C 0 1.099020 7.455787 -2.593007

C 0 -1.045850 5.286306 -1.982644

H 0 -0.649838 9.535764 -1.949223

H 0 -0.550483 7.203148 -3.940553

H 0 -2.334307 7.000444 -2.210663

H 0 -1.136685 7.178767 -0.944199

H 0 1.717393 8.037786 -3.285042

H 0 1.303590 7.817951 -1.577878

H 0 1.429354 6.414643 -2.646374

H 0 -0.039483 5.026683 -1.639988

H 0 -1.756766 4.777401 -1.325701

H 0 -1.167622 4.876341 -2.992176

C -1 -8.524985 6.609925 7.776954

C 0 -7.337664 5.798392 7.272354

O 0 -6.229301 5.877211 7.791134

H 0 -9.225171 5.965513 8.320076

H 0 -8.144993 7.368059 8.460501

H 0 -1.847685 9.212182 -3.210598

H 0 -0.191059 9.658686 -3.654235

H 0 2.042603 5.029361 -4.627163

H 0 2.833144 -1.965177 6.991453

H 0 0.881395 1.364711 11.131010

H 0 -1.781125 -8.316260 11.638109

H 0 -2.163996 -4.145988 13.268286

H 0 -3.420473 -7.963187 4.199974

H 0 -8.065174 -7.419192 0.029647

H 0 -8.109441 -3.305878 -3.918791

H 0 -3.708822 -5.776549 -2.261524

H 0 -3.667396 -5.268034 -3.954341

H 0 0.091766 -7.350889 -0.729083

H 0 1.531929 -1.078686 -6.721933

H 0 -2.461581 1.945355 -5.609616

H 0 -3.445844 0.566098 -5.091056

H 0 -5.610307 5.336835 -4.334053

H 0 -9.254206 7.617264 -1.794874

H 0 -10.446382 7.263219 -3.066559

H 0 -6.308133 3.726177 -4.106477

H 0 -10.914592 -0.605148 -5.216866

H 0 -9.076918 7.086955 6.961650

H 0 -4.033416 7.427077 4.765402

H 0 0.027173 5.679789 6.356658

O 0 7.145008 0.842863 -1.728278

O 0 -6.166032 -0.348259 -1.110927

H 0 7.913508 0.424222 -1.300989

H 0 7.022244 1.652898 -1.206580

H 0 -5.746529 0.475386 -1.455801

H 0 -6.948975 -0.459649 -1.671212

C -1 5.111048 1.406800 8.853850

C 0 3.961367 2.296552 8.363858

C 0 4.386054 3.768230 8.307101

C 0 3.449060 1.822735 6.999865

H 0 4.798151 0.359521 8.926781

H 0 5.958302 1.452175 8.158850

H 0 3.135294 2.210918 9.083355

H 0 5.209161 3.906039 7.595203

H 0 3.557578 4.408248 7.985309

H 0 4.727946 4.124319 9.284895

H 0 2.608677 2.431586 6.651474

H 0 4.244093 1.886030 6.248882

H 0 3.112784 0.781789 7.042868

C -1 -7.140077 -6.831028 9.563872

C 0 -6.857166 -5.899384 8.404294

C 0 -6.418923 -4.586767 8.637981

C 0 -7.045858 -6.304210 7.076207

C 0 -6.211485 -3.701020 7.580526

C 0 -6.824239 -5.426052 6.011085

C 0 -6.412493 -4.116629 6.261657

H 0 -6.340422 -6.771353 10.308311

H 0 -8.077873 -6.558203 10.062416

H 0 -6.224541 -4.272734 9.659501

H 0 -7.375169 -7.320386 6.873465

H 0 -5.874395 -2.688593 7.779837

H 0 -6.977442 -5.755002 4.987472

H 0 -6.228216 -3.440733 5.432563

H 0 -7.234726 -7.867308 9.226424

H 0 5.469026 1.721957 9.839803

H 0 7.769836 -0.483721 2.230630

H 0 -5.118626 -1.516162 -1.355936

C -1 6.647535 5.725784 0.043049

C 0 7.108294 4.565281 -0.806570

O 0 6.849692 3.396430 -0.484106

H 0 7.064653 5.609013 1.046481

N 0 7.816899 4.862529 -1.919804

C 0 8.341471 3.851617 -2.826369

C 0 9.726434 3.302349 -2.470120

O 0 10.550433 3.070863 -3.347918

H 0 7.660660 2.993271 -2.833178

H 0 8.072192 5.824410 -2.084856

N 0 9.944270 3.067170 -1.141542

C -1 11.056566 2.194051 -0.766065

C 0 10.607392 0.760950 -0.970304

O 0 9.609153 0.349337 -0.359042

H 0 11.930499 2.476926 -1.347719

H 0 9.115027 2.967883 -0.564230

N 0 11.272633 0.001374 -1.849378

C -1 10.757114 -1.336918 -2.119972

C 0 11.770039 -1.918747 -3.113205

C 0 12.263263 -0.676846 -3.871872

C 0 12.347513 0.398311 -2.779792

H 0 11.326212 -2.674966 -3.764433

H 0 12.601226 -2.385647 -2.573086

H 0 11.524250 -0.374980 -4.621532

H 0 13.219061 -0.826052 -4.379471

H 0 13.319191 0.369705 -2.270458

H 0 12.164293 1.401776 -3.165394

H 0 9.753038 -1.262627 -2.555861

H 0 5.559657 5.675340 0.137757

H 0 6.930421 6.701918 -0.356879

H 0 8.404501 4.262377 -3.833933

H 0 11.270455 2.340940 0.295407

H 0 10.666456 -1.906345 -1.192288

N 0 -4.583501 1.465963 1.017229

C 0 -3.142639 1.236753 1.299023

C 0 -2.351669 1.098357 -0.003314

C 0 -0.904650 0.753575 0.247476

C 0 0.092885 1.714904 0.040479

C 0 1.433013 1.434757 0.300896

C 0 1.780490 0.163167 0.792490

C 0 0.802062 -0.804988 1.001363

C 0 -0.535183 -0.511605 0.725173

O 0 3.091993 -0.109970 1.078883

O 0 2.407779 2.383735 0.175885

H 0 3.554563 0.744995 1.042702

H 0 -1.301790 -1.262416 0.892332

H 0 -3.012745 0.344185 1.912236

H 0 -2.406828 2.034105 -0.564692

H 0 -0.165196 2.708995 -0.311569

H 0 1.095071 -1.771025 1.394229

H 0 -4.641560 2.541000 0.073909

H 0 -2.827940 0.326534 -0.615144

H 0 -2.778004 2.090408 1.875448

H 0 -5.058389 1.615118 1.907324

H 0 3.039185 2.185917 -0.603628

H 0 -4.991897 0.607491 0.642647

C 0 -3.641221 1.183719 9.078614

C 0 -3.603654 -0.211477 9.078485

C 0 -3.687486 -0.918827 7.876573

C 0 -3.804251 -0.234561 6.660163

C 0 -3.893310 -0.985058 5.329663

C 0 -5.145634 -0.474475 4.644279

C 0 -3.828888 1.167047 6.668112

C 0 -3.750380 1.872243 7.868203

O 0 -5.175485 0.406282 3.805260

H 0 -3.513077 -0.755186 10.013250

H 0 -3.645452 -2.003522 7.892348

H 0 -6.090682 -0.913053 5.027135

H 0 -3.911175 1.724748 5.742834

H 0 -3.782690 2.956447 7.838162

H 0 -3.583329 1.730348 10.015307

H 0 -4.060280 -2.046333 5.547160

C 0 -2.632962 -0.810580 4.478375

H 0 -1.767158 -1.216749 5.008944

H 0 -2.742480 -1.344306 3.531078

H 0 -2.441393 0.245050 4.277458

**Int2S** (-8045.701456)

C -1 7.280907 0.460467 1.969980

C 0 5.931419 0.622317 2.677781

C 0 6.079711 0.508057 4.199093

C 0 5.263055 1.950380 2.298969

H 0 7.161031 0.459673 0.884323

H 0 7.962731 1.280230 2.226976

H 0 5.273889 -0.192859 2.345489

H 0 6.720720 1.308884 4.589275

H 0 5.108628 0.583918 4.697427

H 0 6.530468 -0.448035 4.486278

H 0 4.260876 2.036916 2.733162

H 0 5.855302 2.799003 2.663232

H 0 5.183354 2.075664 1.214095

C -1 2.868127 -2.653716 6.140069

C 0 3.062579 -1.957632 4.787052

C 0 3.004419 -2.953550 3.623057

C 0 2.040242 -0.834482 4.584366

H 0 1.881192 -3.130411 6.192837

H 0 3.620976 -3.432853 6.302557

H 0 4.062337 -1.503153 4.786443

H 0 2.024505 -3.445092 3.586121

H 0 3.160945 -2.444806 2.666669

H 0 3.764052 -3.736399 3.725056

H 0 1.017252 -1.230938 4.569692

H 0 2.098049 -0.096914 5.390517

H 0 2.209631 -0.318614 3.635415

C -1 0.466585 0.417010 10.770894

C 0 0.847813 0.460575 9.286606

C 0 0.397889 -0.811439 8.560705

C 0 0.268829 1.704191 8.602491

H 0 -0.622589 0.356833 10.884677

H 0 0.903667 -0.455120 11.269614

H 0 1.943740 0.515946 9.225272

H 0 -0.692577 -0.908262 8.584095

H 0 0.704779 -0.794598 7.509708

H 0 0.827772 -1.707360 9.022804

H 0 -0.825442 1.691747 8.636326

H 0 0.614904 2.625634 9.083866

H 0 0.565244 1.744623 7.550055

C -1 -3.085947 -4.132907 12.675630

C 0 -3.271813 -5.447164 11.898990

O 0 -4.339948 -5.715360 11.356167

C 0 -3.088540 -2.953932 11.690719

H 0 -3.927655 -4.050100 13.368911

H 0 -2.255905 -3.031214 10.984107

H 0 -4.016603 -2.955361 11.113658

H 0 -3.004587 -2.000007 12.219582

N 0 -2.177538 -6.268558 11.832250

C -1 -2.114060 -7.480049 11.012453

C 0 -1.157505 -7.333242 9.819130

C 0 -1.533490 -6.233318 8.849771

C 0 -2.751346 -6.278006 8.157310

C 0 -0.667394 -5.161450 8.606506

C 0 -3.078534 -5.298301 7.223810

C 0 -0.990452 -4.174593 7.670914

C 0 -2.191433 -4.249962 6.967442

H 0 -3.136023 -7.663213 10.675710

H 0 -1.130864 -8.298702 9.296728

H 0 -0.140008 -7.160421 10.191305

H 0 -3.453890 -7.084019 8.347435

H 0 -1.317884 -5.937780 12.244695

H 0 0.277745 -5.106453 9.141312

H 0 -4.023785 -5.347506 6.699596

H 0 -0.308919 -3.348828 7.496396

H 0 -2.436013 -3.509799 6.214022

C -1 0.152350 -6.400879 -0.187590

C 0 1.336986 -5.531631 -0.634627

C 0 1.339456 -5.324646 -2.153944

C 0 1.340683 -4.182256 0.091934

H 0 0.164753 -6.564469 0.895865

H 0 -0.799525 -5.916939 -0.437179

H 0 2.262429 -6.061342 -0.366386

H 0 0.446234 -4.774016 -2.470807

H 0 2.207313 -4.739908 -2.474379

H 0 1.350538 -6.279865 -2.690010

H 0 0.409575 -3.636877 -0.100414

H 0 1.434197 -4.309031 1.175195

H 0 2.167452 -3.548600 -0.243515

C -1 -3.596750 -6.933916 3.872073

C 0 -2.270319 -6.182318 3.812712

S 0 -2.526827 -4.447333 3.263701

C 0 -0.809803 -3.859089 3.433598

H 0 -4.280144 -6.466469 4.586306

H 0 -4.093308 -6.948034 2.897129

H 0 -1.576986 -6.666654 3.116336

H 0 -1.805854 -6.155534 4.802985

H 0 -0.135212 -4.448170 2.808251

H 0 -0.779500 -2.820163 3.102187

H 0 -0.482724 -3.910512 4.476069

C -1 -8.592991 -6.747969 0.713026

C 0 -7.665683 -5.682173 1.240870

C 0 -6.393704 -5.487608 0.695338

C 0 -8.064566 -4.824044 2.276271

C 0 -5.555410 -4.464899 1.139965

C 0 -7.247268 -3.789501 2.722439

C 0 -5.991873 -3.585950 2.136647

O 0 -5.241051 -2.533440 2.565102

H 0 -9.026864 -7.344272 1.523019

H 0 -9.425326 -6.293371 0.162749

H 0 -6.042927 -6.149361 -0.092899

H 0 -9.045642 -4.952785 2.724921

H 0 -4.559234 -4.357519 0.733464

H 0 -7.577934 -3.114188 3.504423

H 0 -4.560722 -2.336466 1.893722

C -1 -3.717711 -4.902584 -2.923995

C 0 -2.570390 -3.949741 -2.578163

C 0 -2.609503 -3.364717 -1.161537

C 0 -3.737949 -2.404247 -0.849440

O 0 -4.587347 -2.220198 -1.839358

O 0 -3.831590 -1.830721 0.238244

H 0 -4.681675 -4.392796 -2.880785

H 0 -2.548864 -3.127784 -3.298804

H 0 -1.618572 -4.481400 -2.686508

H 0 -1.689642 -2.805357 -0.954393

H 0 -2.641035 -4.152851 -0.397360

C -1 2.462886 -1.588938 -6.455080

C 0 2.758194 -1.448792 -4.976356

C 0 1.734702 -1.597809 -4.029790

C 0 4.051049 -1.180272 -4.509956

C 0 1.988324 -1.473808 -2.663960

C 0 4.319197 -1.073961 -3.142049

C 0 3.285782 -1.217350 -2.212825

H 0 3.276723 -1.185343 -7.063723

H 0 2.330166 -2.640977 -6.734485

H 0 0.724901 -1.805486 -4.369897

H 0 4.859037 -1.051912 -5.225996

H 0 1.177229 -1.566592 -1.949048

H 0 5.328097 -0.851267 -2.803812

H 0 3.477800 -1.114035 -1.149159

C -1 2.556977 4.064056 -4.609216

C 0 2.977944 3.707696 -3.179244

C 0 3.666544 2.345794 -3.096270

N 0 4.124858 2.060577 -1.722556

H 0 3.425600 4.120696 -5.274767

H 0 1.875375 3.310192 -5.014925

H 0 2.109727 3.704918 -2.510102

H 0 3.659418 4.480414 -2.793988

H 0 4.499210 2.304219 -3.814958

H 0 2.966661 1.554801 -3.381081

H 0 4.848499 2.724025 -1.446501

H 0 4.578472 1.148864 -1.702004

C -1 -2.421908 0.949302 -5.153818

C 0 -1.703096 0.974576 -3.799140

C 0 -1.729948 -0.417827 -3.164141

C 0 -0.272646 1.513064 -3.916958

H 0 -1.932030 0.248940 -5.841737

H 0 -2.262529 1.648394 -3.139051

H 0 -2.759610 -0.742201 -2.990698

H 0 -1.261257 -1.152509 -3.828683

H 0 -1.193201 -0.435865 -2.211457

H 0 0.334774 0.886792 -4.580636

H 0 -0.269567 2.532796 -4.318753

H 0 0.220618 1.531650 -2.939634

C -1 -8.784080 -2.890608 -3.161058

C 0 -9.007648 -3.879297 -2.019935

S 0 -9.982174 -3.219339 -0.613893

C 0 -8.770429 -2.049924 0.090170

H 0 -9.734087 -2.520039 -3.552244

H 0 -8.200578 -2.026513 -2.831542

H 0 -8.058201 -4.252702 -1.620621

H 0 -9.576270 -4.746679 -2.369526

H 0 -8.731882 -1.120233 -0.478421

H 0 -9.094681 -1.823014 1.106924

H 0 -7.783764 -2.513446 0.146235

C -1 -11.413971 -0.217158 -4.324807

C 0 -10.469276 0.429280 -3.334099

C 0 -9.215899 0.914959 -3.718928

C 0 -10.834200 0.585003 -1.988466

C 0 -8.367656 1.570361 -2.819307

C 0 -10.005305 1.233621 -1.077951

C 0 -8.772686 1.751288 -1.493014

O 0 -8.050717 2.453274 -0.564728

H 0 -11.905364 -1.092330 -3.888584

H 0 -12.201118 0.480995 -4.633098

H 0 -8.889267 0.794798 -4.748264

H 0 -11.784168 0.186586 -1.644493

H 0 -7.409918 1.951586 -3.156965

H 0 -10.298166 1.354387 -0.040629

H 0 -7.137990 2.638442 -0.861279

C -1 -5.820178 4.559262 -3.594201

C 0 -4.547595 4.000476 -2.933240

C 0 -4.882349 2.766473 -2.132398

O -1 -5.248249 2.993762 -0.889906

O 0 -4.871349 1.640837 -2.650441

H 0 -6.590479 4.802833 -2.856430

H 0 -4.108381 4.747469 -2.268784

H 0 -3.814176 3.721019 -3.694074

C -1 -9.495881 6.948242 -2.625198

C 0 -9.722541 5.624019 -1.908368

C 0 -10.363442 4.584159 -2.827185

O 0 -8.446798 5.199203 -1.426149

H 0 -8.850796 6.797210 -3.497158

H 0 -10.396638 5.800394 -1.052474

H 0 -11.312980 4.956165 -3.227187

H 0 -9.696674 4.361082 -3.665940

H 0 -10.565075 3.648353 -2.299751

H 0 -8.543144 4.347127 -0.970297

N 0 -7.558385 4.986065 6.178956

C 0 -6.514263 4.026173 5.778067

C 0 -5.227168 4.647413 5.245377

O 0 -4.198137 3.973057 5.206142

C 0 -7.196610 3.190146 4.674222

C 0 -8.678975 3.236991 5.060838

C 0 -8.860284 4.675520 5.563264

H 0 -6.224841 3.407535 6.632819

H 0 -6.775329 2.187230 4.601370

H 0 -7.049582 3.679482 3.704357

H 0 -8.880027 2.533781 5.876269

H 0 -9.348624 2.996944 4.231382

H 0 -9.069488 5.359782 4.729731

H 0 -9.672126 4.771382 6.287350

N 0 -5.328889 5.894063 4.737991

C -1 -4.224762 6.582907 4.088989

C 0 -4.576101 7.012360 2.659264

C 0 -4.866694 5.857507 1.687160

C 0 -5.384532 6.390233 0.345786

C 0 -3.635723 4.967291 1.481294

H 0 -3.380610 5.892467 4.101220

H 0 -3.742607 7.612051 2.268451

H 0 -5.444500 7.686043 2.695035

H 0 -5.661344 5.240630 2.131142

H 0 -6.265356 7.028660 0.472733

H 0 -4.609894 6.982040 -0.159313

H 0 -6.203910 6.378231 4.875713

H 0 -5.667937 5.567480 -0.314525

H 0 -3.327490 4.459623 2.398006

H 0 -3.845565 4.204919 0.727662

H 0 -2.783438 5.562119 1.128787

C -1 -0.160299 5.153803 5.413078

C 0 -1.047351 3.914081 5.513916

S 0 -1.264167 2.990945 3.945253

C 0 0.400635 2.277686 3.727093

H 0 0.861932 4.897767 5.115936

H 0 -0.557559 5.857043 4.674965

H 0 -2.068056 4.184226 5.788806

H 0 -0.671423 3.217737 6.270035

H 0 1.139280 3.026152 3.430313

H 0 0.723072 1.781483 4.645835

H 0 0.324127 1.532843 2.934354

C -1 -0.793784 9.087919 -2.939864

C 0 -0.435435 7.594567 -2.870105

C 0 -1.343601 6.857035 -1.867513

C 0 1.052549 7.413303 -2.542483

C 0 -1.129610 5.341310 -1.796950

H 0 -0.622090 9.572424 -1.971134

H 0 -0.616883 7.156256 -3.863371

H 0 -2.390148 7.059227 -2.132403

H 0 -1.195113 7.291898 -0.868892

H 0 1.680100 7.956600 -3.257366

H 0 1.277272 7.800061 -1.540733

H 0 1.353471 6.362229 -2.567742

H 0 -0.129540 5.086499 -1.433500

H 0 -1.849109 4.880102 -1.113314

H 0 -1.254097 4.873810 -2.780747

C -1 -8.524986 6.609922 7.776932

C 0 -7.355568 5.757612 7.299259

O 0 -6.269936 5.764258 7.868873

H 0 -9.276799 5.986190 8.273221

H 0 -8.140041 7.330654 8.497316

H 0 -1.847043 9.232952 -3.202335

H 0 -0.184052 9.608564 -3.685655

H 0 2.045209 5.030576 -4.641441

H 0 2.935734 -1.938304 6.968044

H 0 0.808925 1.314544 11.296738

H 0 -1.805093 -8.326521 11.636698

H 0 -2.162718 -4.143924 13.266317

H 0 -3.427016 -7.967620 4.188742

H 0 -8.070917 -7.429852 0.035795

H 0 -8.229342 -3.367564 -3.977316

H 0 -3.748812 -5.749064 -2.226924

H 0 -3.589830 -5.310396 -3.931564

H 0 0.167134 -7.380402 -0.677184

H 0 1.541697 -1.063337 -6.726514

H 0 -2.417221 1.937842 -5.627793

H 0 -3.464479 0.641355 -5.031017

H 0 -5.582857 5.470403 -4.150810

H 0 -9.005726 7.663212 -1.958669

H 0 -10.444452 7.375502 -2.963934

H 0 -6.232842 3.834073 -4.302935

H 0 -10.885439 -0.538057 -5.226601

H 0 -9.019020 7.134121 6.953615

H 0 -3.943308 7.460503 4.684402

H 0 -0.108468 5.663113 6.382539

O 0 7.127276 0.846794 -1.739635

O 0 -6.215376 -0.283053 -1.429890

H 0 7.902356 0.437981 -1.315226

H 0 6.997663 1.658192 -1.221960

H 0 -5.714549 0.431607 -1.911364

H 0 -7.107922 -0.275484 -1.801320

C -1 5.111043 1.406803 8.853846

C 0 4.107094 2.448207 8.341471

C 0 4.768657 3.824152 8.200049

C 0 3.480434 2.006005 7.014632

H 0 4.634483 0.430494 9.001912

H 0 5.927923 1.271465 8.134505

H 0 3.300679 2.535892 9.082473

H 0 5.583018 3.787394 7.466064

H 0 4.049521 4.577582 7.861513

H 0 5.192193 4.164381 9.151153

H 0 2.740843 2.731443 6.659628

H 0 4.245781 1.904443 6.237860

H 0 2.975654 1.038930 7.116555

C -1 -7.140077 -6.831028 9.563873

C 0 -6.821439 -5.872716 8.437149

C 0 -6.374687 -4.573399 8.720714

C 0 -6.984798 -6.235496 7.094172

C 0 -6.136724 -3.657112 7.696964

C 0 -6.731517 -5.326889 6.061930

C 0 -6.313982 -4.029604 6.361787

H 0 -6.341576 -6.820482 10.312018

H 0 -8.069425 -6.542533 10.069166

H 0 -6.198659 -4.293724 9.755310

H 0 -7.320883 -7.240936 6.852695

H 0 -5.799748 -2.654811 7.941271

H 0 -6.867745 -5.622726 5.025944

H 0 -6.111871 -3.328564 5.557878

H 0 -7.267747 -7.852572 9.193920

H 0 5.552648 1.714771 9.807645

H 0 7.771419 -0.477727 2.248783

H 0 -5.288196 -1.526969 -1.604689

C -1 6.647540 5.725775 0.043040

C 0 7.097949 4.566012 -0.813056

O 0 6.819701 3.398896 -0.502571

H 0 7.057488 5.595627 1.047819

N 0 7.821377 4.863164 -1.917007

C 0 8.343705 3.853721 -2.826099

C 0 9.727759 3.302731 -2.469933

O 0 10.550818 3.066587 -3.347270

H 0 7.661522 2.996448 -2.833821

H 0 8.089632 5.823236 -2.071716

N 0 9.946549 3.070718 -1.140586

C -1 11.056567 2.194056 -0.766057

C 0 10.603825 0.762826 -0.974409

O 0 9.599552 0.355121 -0.370560

H 0 11.931827 2.476387 -1.345931

H 0 9.117405 2.970799 -0.563457

N 0 11.273376 0.000738 -1.847846

C -1 10.757107 -1.336920 -2.119969

C 0 11.774183 -1.921097 -3.107648

C 0 12.273377 -0.680533 -3.864603

C 0 12.353692 0.395297 -2.772861

H 0 11.332357 -2.676886 -3.760719

H 0 12.601789 -2.389143 -2.563037

H 0 11.538696 -0.378002 -4.618224

H 0 13.231533 -0.831655 -4.367165

H 0 13.322731 0.365760 -2.258584

H 0 12.173736 1.398719 -3.160166

H 0 9.755276 -1.261574 -2.560704

H 0 5.558587 5.689290 0.131346

H 0 6.945719 6.701229 -0.347351

H 0 8.407145 4.265682 -3.833226

H 0 11.269462 2.337881 0.296041

H 0 10.661104 -1.905516 -1.192292

N 0 -4.832040 1.194458 0.784017

C 0 -3.391697 1.196808 1.155793

C 0 -2.506020 1.231288 -0.091874

C 0 -1.064458 0.887711 0.206110

C 0 -0.045392 1.827285 0.009433

C 0 1.289080 1.517363 0.274336

C 0 1.610102 0.237860 0.763285

C 0 0.609463 -0.709571 0.964438

C 0 -0.719222 -0.386657 0.680855

O 0 2.913408 -0.063842 1.064646

O 0 2.281059 2.450268 0.158627

H 0 3.399535 0.777269 1.024624

H 0 -1.499066 -1.125783 0.837629

H 0 -3.167524 0.311065 1.747328

H 0 -2.557553 2.221481 -0.555321

H 0 -0.277048 2.828171 -0.340179

H 0 0.879867 -1.683856 1.354944

H -1 -5.128337 2.155721 -0.172176

H 0 -2.911915 0.523292 -0.820801

H 0 -3.198993 2.063655 1.790366

H 0 -5.376285 1.270197 1.644082

H 0 2.935821 2.226790 -0.592448

H 0 -5.068189 0.279015 0.394284

C 0 -3.552660 1.105962 8.936028

C 0 -3.511259 -0.288856 8.895384

C 0 -3.614092 -0.959822 7.674425

C 0 -3.751376 -0.241289 6.481165

C 0 -3.856368 -0.951913 5.131676

C 0 -5.129448 -0.439448 4.488550

C 0 -3.781191 1.159157 6.528058

C 0 -3.685223 1.828261 7.747613

O 0 -5.184493 0.435025 3.644576

H 0 -3.402628 -0.860034 9.811947

H 0 -3.574280 -2.043522 7.653365

H 0 -6.061129 -0.869492 4.911567

H 0 -3.885086 1.743987 5.621456

H 0 -3.721450 2.913053 7.754021

H 0 -3.481185 1.625989 9.886831

H 0 -4.005221 -2.023304 5.308706

C 0 -2.611261 -0.734504 4.268526

H 0 -1.732756 -1.131161 4.785220

H 0 -2.714051 -1.254829 3.312866

H 0 -2.441123 0.327009 4.079114

**TS2S** (-8045.686652)

C -1 7.281889 0.461245 1.969176

C 0 6.016743 0.896560 2.716903

C 0 6.180320 0.738511 4.231381

C 0 5.633666 2.340148 2.364023

H 0 7.146467 0.524629 0.885069

H 0 8.138503 1.093993 2.228774

H 0 5.191695 0.250038 2.395279

H 0 6.966413 1.404213 4.610709

H 0 5.251717 0.983606 4.756081

H 0 6.456139 -0.287167 4.498697

H 0 4.693577 2.635042 2.845435

H 0 6.407044 3.040305 2.708229

H 0 5.534626 2.479083 1.282673

C -1 2.868031 -2.655295 6.140050

C 0 2.936278 -3.105858 4.673656

C 0 1.535639 -3.383562 4.109693

C 0 3.683849 -2.080360 3.810989

H 0 2.343015 -1.696431 6.224629

H 0 2.330618 -3.381328 6.759001

H 0 3.503677 -4.047360 4.640853

H 0 0.935219 -2.468629 4.089075

H 0 1.576087 -3.763170 3.083056

H 0 1.003277 -4.124168 4.716856

H 0 3.150709 -1.123168 3.778434

H 0 4.688297 -1.887004 4.203046

H 0 3.787827 -2.427128 2.777208

C -1 0.465961 0.418174 10.771475

C 0 1.256932 0.976367 9.581427

C 0 1.385978 -0.066392 8.467710

C 0 0.617134 2.262082 9.043948

H 0 -0.554658 0.153787 10.469391

H 0 0.936955 -0.485463 11.173818

H 0 2.268744 1.215302 9.936350

H 0 0.399402 -0.338418 8.078808

H 0 1.966684 0.319378 7.624516

H 0 1.878269 -0.978035 8.822539

H 0 -0.398272 2.065200 8.680530

H 0 0.551205 3.031783 9.820372

H 0 1.191644 2.672930 8.207357

C -1 -3.085941 -4.133040 12.675992

C 0 -3.243028 -5.429316 11.863229

O 0 -4.234052 -5.610906 11.161261

C 0 -2.897080 -2.944992 11.722231

H 0 -4.003809 -4.011246 13.258172

H 0 -1.979930 -3.052609 11.136079

H 0 -3.736971 -2.890930 11.025802

H 0 -2.843558 -2.004885 12.278721

N 0 -2.227775 -6.349651 11.943825

C -1 -2.114067 -7.479945 11.011975

C 0 -0.961536 -7.284646 10.004740

C 0 -0.921958 -5.896255 9.401915

C 0 -1.949353 -5.428721 8.570600

C 0 0.135107 -5.030496 9.706184

C 0 -1.910559 -4.132625 8.058661

C 0 0.174820 -3.730469 9.198503

C 0 -0.852285 -3.276941 8.371816

H 0 -3.075961 -7.526228 10.498923

H 0 -1.064852 -8.046237 9.221970

H 0 -0.005090 -7.482246 10.503152

H 0 -2.797217 -6.066169 8.338032

H 0 -1.395599 -6.073639 12.444835

H 0 0.939451 -5.380646 10.348620

H 0 -2.714079 -3.788831 7.418044

H 0 1.000435 -3.072800 9.450994

H 0 -0.833107 -2.262618 7.988417

C -1 0.152881 -6.400872 -0.187984

C 0 0.964991 -5.324009 -0.921119

C 0 0.190550 -4.764402 -2.121018

C 0 1.362034 -4.191391 0.032532

H 0 0.721815 -6.830047 0.643599

H 0 -0.766458 -5.970485 0.226612

H 0 1.885239 -5.791052 -1.300468

H 0 -0.726088 -4.263870 -1.785457

H 0 0.788199 -4.031353 -2.671512

H 0 -0.097151 -5.559920 -2.817466

H 0 0.468747 -3.668879 0.390910

H 0 1.900500 -4.572797 0.907784

H 0 1.997948 -3.455974 -0.471827

C -1 -3.596867 -6.933977 3.872024

C 0 -2.695024 -6.281327 2.825637

S 0 -2.863949 -4.456240 2.680475

C 0 -2.475683 -3.960398 4.392212

H 0 -3.341590 -6.607675 4.883990

H 0 -4.647154 -6.684026 3.699012

H 0 -2.930169 -6.653043 1.823613

H 0 -1.641048 -6.510641 3.016357

H 0 -1.537710 -4.407436 4.731720

H 0 -2.355352 -2.876988 4.386667

H 0 -3.284782 -4.228738 5.075162

C -1 -8.592930 -6.747974 0.713125

C 0 -8.321969 -5.488272 1.504908

C 0 -7.079864 -4.844552 1.483815

C 0 -9.325902 -4.921512 2.305008

C 0 -6.841908 -3.672447 2.208558

C 0 -9.106343 -3.762948 3.040422

C 0 -7.864049 -3.111931 2.985373

O 0 -7.714989 -1.973829 3.703166

H 0 -9.574055 -6.698997 0.230393

H 0 -7.841411 -6.893669 -0.068978

H 0 -6.265923 -5.266843 0.898704

H 0 -10.305916 -5.390123 2.336026

H 0 -5.869906 -3.195641 2.159371

H 0 -9.892099 -3.324840 3.646760

H 0 -6.932848 -1.449315 3.400796

C -1 -3.717879 -4.902605 -2.923837

C 0 -3.892766 -3.693842 -2.003769

C 0 -5.360261 -3.389576 -1.678657

C 0 -5.498486 -2.282153 -0.657287

O 0 -4.706846 -2.122002 0.259516

O 0 -6.565524 -1.508988 -0.846863

H 0 -4.239099 -4.757137 -3.876751

H 0 -3.459546 -2.808151 -2.480623

H 0 -3.349910 -3.840959 -1.064906

H 0 -5.845811 -4.273448 -1.243830

H 0 -5.928453 -3.129213 -2.575044

C -1 2.463035 -1.588842 -6.455116

C 0 2.760060 -1.355139 -4.983352

C 0 1.768966 -1.540320 -4.010544

C 0 4.036708 -0.963930 -4.551185

C 0 2.036544 -1.345710 -2.651632

C 0 4.322016 -0.784085 -3.195315

C 0 3.318327 -0.979819 -2.240358

H 0 2.874379 -0.786901 -7.075568

H 0 2.907199 -2.528893 -6.802086

H 0 0.773255 -1.840219 -4.320911

H 0 4.820325 -0.799565 -5.287035

H 0 1.247607 -1.476108 -1.916492

H 0 5.314710 -0.466904 -2.883525

H 0 3.527895 -0.846487 -1.182545

C -1 2.557156 4.063985 -4.609080

C 0 2.828117 3.868440 -3.115526

C 0 3.408683 2.489950 -2.809421

N 0 3.707091 2.348980 -1.371306

H 0 3.479878 3.979478 -5.193701

H 0 1.858746 3.307407 -4.981283

H 0 1.902551 3.993415 -2.540313

H 0 3.522493 4.642737 -2.759437

H 0 4.303095 2.309151 -3.422402

H 0 2.684890 1.715551 -3.077328

H 0 4.525921 2.907830 -1.128551

H 0 3.958467 1.383247 -1.169153

C -1 -2.421973 0.948817 -5.153644

C 0 -1.758724 0.912002 -3.769105

C 0 -1.707145 -0.525847 -3.241372

C 0 -0.363476 1.548678 -3.793768

H 0 -1.824559 0.384592 -5.879940

H 0 -2.379762 1.500278 -3.077028

H 0 -2.714758 -0.908048 -3.055856

H 0 -1.234261 -1.188292 -3.974272

H 0 -1.141993 -0.593622 -2.308803

H 0 0.309942 0.992057 -4.455429

H 0 -0.407155 2.584898 -4.148383

H 0 0.082750 1.549972 -2.794426

C -1 -8.784154 -2.890548 -3.160900

C 0 -9.166585 -3.942527 -2.121167

S 0 -10.594750 -3.475168 -1.071518

C 0 -9.827466 -2.151351 -0.075216

H 0 -9.649135 -2.641196 -3.780625

H 0 -8.423214 -1.980498 -2.681185

H 0 -8.327829 -4.171816 -1.456084

H 0 -9.467195 -4.877809 -2.603769

H 0 -8.924785 -2.523467 0.410685

H 0 -9.601700 -1.276999 -0.685579

H 0 -10.550148 -1.873862 0.693823

C -1 -11.413519 -0.217402 -4.324783

C 0 -10.734867 0.414899 -3.135091

C 0 -9.345225 0.577854 -3.108205

C 0 -11.461955 0.825181 -2.011350

C 0 -8.698087 1.128191 -2.005221

C 0 -10.831972 1.401007 -0.907982

C 0 -9.448591 1.563849 -0.910306

O 0 -8.843519 2.136599 0.197560

H 0 -11.397713 -1.310618 -4.248038

H 0 -12.460611 0.089705 -4.396362

H 0 -8.754287 0.254293 -3.960283

H 0 -12.541181 0.701353 -2.001640

H 0 -7.618415 1.202806 -1.990724

H 0 -11.398338 1.742416 -0.048665

H 0 -8.226647 2.825885 -0.128872

C -1 -5.818521 4.554974 -3.596533

C 0 -4.987270 3.708874 -2.627399

C 0 -5.793869 3.119300 -1.491272

O 0 -6.846000 3.609866 -1.082930

O 0 -5.251225 2.030511 -0.974200

H 0 -6.296701 5.388316 -3.076144

H 0 -4.197250 4.315922 -2.164882

H 0 -4.475301 2.888537 -3.138963

C -1 -9.497188 6.950396 -2.623715

C 0 -9.946579 5.611289 -2.031056

C 0 -9.987264 4.499024 -3.079299

O 0 -9.139683 5.250269 -0.912395

H 0 -8.497360 6.858022 -3.066515

H 0 -10.954742 5.738345 -1.616322

H 0 -10.640242 4.770095 -3.915853

H 0 -8.984274 4.319029 -3.483973

H 0 -10.347986 3.565963 -2.642825

H 0 -8.273126 4.962392 -1.239067

N 0 -7.671403 5.075353 6.020664

C 0 -6.702475 4.070090 5.543856

C 0 -5.373402 4.628464 5.043348

O 0 -4.405636 3.880524 4.896774

C 0 -7.447294 3.363269 4.388648

C 0 -8.924105 3.510219 4.771072

C 0 -8.986728 4.920556 5.372661

H 0 -6.459134 3.376871 6.353709

H 0 -7.118310 2.330863 4.257932

H 0 -7.252650 3.893579 3.449167

H 0 -9.192205 2.770345 5.532635

H 0 -9.599040 3.386143 3.921132

H 0 -9.122846 5.675773 4.586987

H 0 -9.797227 5.037410 6.094765

N 0 -5.377985 5.931283 4.681649

C -1 -4.224920 6.582791 4.089352

C 0 -4.445714 6.991877 2.625016

C 0 -4.881165 5.866197 1.670203

C 0 -5.151894 6.441064 0.273942

C 0 -3.864199 4.718398 1.604656

H 0 -3.399740 5.874248 4.175325

H 0 -3.511261 7.439254 2.257606

H 0 -5.199630 7.790266 2.588180

H 0 -5.826522 5.456757 2.051593

H 0 -5.862238 7.273728 0.313773

H 0 -4.225027 6.812573 -0.181159

H 0 -6.209846 6.464096 4.888268

H 0 -5.578558 5.679922 -0.383402

H 0 -3.727803 4.246449 2.581229

H 0 -4.195789 3.942868 0.905468

H 0 -2.890144 5.083358 1.256067

C -1 -0.159843 5.153580 5.412976

C 0 1.051690 5.635860 4.618681

S 0 1.476807 4.636146 3.135713

C 0 -0.024714 4.879339 2.129949

H 0 -1.080460 5.198208 4.824301

H 0 -0.030996 4.118117 5.740512

H 0 1.954575 5.595479 5.236732

H 0 0.922938 6.677972 4.304836

H 0 -0.902614 4.438115 2.606216

H 0 -0.202579 5.943651 1.948067

H 0 0.163473 4.372156 1.183827

C -1 -0.794067 9.087981 -2.940076

C 0 -0.393129 7.611521 -2.776817

C 0 -1.333856 6.885074 -1.795719

C 0 1.077025 7.494932 -2.352851

C 0 -1.100497 5.373755 -1.681657

H 0 -0.699402 9.622386 -1.987013

H 0 -0.501216 7.122952 -3.757245

H 0 -2.370869 7.064301 -2.110170

H 0 -1.235164 7.346239 -0.802417

H 0 1.730522 8.021164 -3.056959

H 0 1.228516 7.939708 -1.361526

H 0 1.410221 6.454528 -2.304068

H 0 -0.123085 5.133634 -1.252890

H 0 -1.855344 4.909975 -1.037200

H 0 -1.158432 4.889378 -2.663820

C -1 -8.525009 6.609716 7.776797

C 0 -7.423154 5.717842 7.214050

O 0 -6.344946 5.585685 7.781557

H 0 -9.327742 6.001631 8.208396

H 0 -8.087014 7.220300 8.565340

H 0 -1.832162 9.184694 -3.274664

H 0 -0.153850 9.593376 -3.670203

H 0 2.122261 5.048345 -4.807694

H 0 3.868377 -2.519717 6.564196

H 0 0.394893 1.151123 11.582029

H 0 -1.977661 -8.412372 11.569682

H 0 -2.248492 -4.204245 13.380305

H 0 -3.484556 -8.023061 3.835571

H 0 -8.581030 -7.641162 1.350227

H 0 -7.988515 -3.268334 -3.815243

H 0 -4.121349 -5.811616 -2.462853

H 0 -2.661509 -5.079311 -3.141869

H 0 -0.130191 -7.216773 -0.862063

H 0 1.386562 -1.642691 -6.636122

H 0 -2.516567 1.974385 -5.527560

H 0 -3.422498 0.503530 -5.128206

H 0 -5.180474 4.961493 -4.384874

H 0 -9.453041 7.714515 -1.842610

H 0 -10.181229 7.289701 -3.409061

H 0 -6.604103 3.955936 -4.065256

H 0 -10.914657 0.054212 -5.259879

H 0 -8.970008 7.251866 7.011079

H 0 -3.962683 7.466718 4.682496

H 0 -0.306563 5.780515 6.299263

O 0 7.202893 0.964375 -1.974467

O 0 -6.669235 0.664684 0.788591

H 0 7.919911 0.597687 -1.427278

H 0 6.878645 1.727545 -1.470803

H 0 -7.569583 1.063267 0.813543

H 0 -6.353863 0.475341 1.712490

C -1 5.110501 1.406094 8.853750

C 0 4.470870 2.750663 8.486663

C 0 5.429620 3.910154 8.780807

C 0 4.019922 2.773293 7.021183

H 0 4.418835 0.575103 8.677891

H 0 6.006658 1.225430 8.247787

H 0 3.577197 2.883334 9.111041

H 0 6.340801 3.821120 8.176653

H 0 4.969761 4.876776 8.548538

H 0 5.728764 3.923972 9.834342

H 0 3.515089 3.714019 6.774301

H 0 4.879861 2.667789 6.349137

H 0 3.323297 1.957745 6.801350

C -1 -7.139897 -6.830974 9.563999

C 0 -6.811583 -6.063659 8.297135

C 0 -5.804525 -5.089178 8.284966

C 0 -7.521323 -6.296070 7.108680

C 0 -5.535825 -4.358696 7.122829

C 0 -7.244664 -5.578049 5.944785

C 0 -6.248907 -4.597855 5.948031

H 0 -6.500787 -6.511516 10.389470

H 0 -8.186983 -6.682430 9.852122

H 0 -5.241008 -4.900235 9.192936

H 0 -8.308880 -7.046527 7.099793

H 0 -4.780899 -3.576640 7.148475

H 0 -7.814109 -5.759542 5.037998

H 0 -6.056170 -4.021511 5.049720

H 0 -6.996940 -7.908556 9.421105

H 0 5.407267 1.380535 9.907663

H 0 7.551699 -0.572591 2.206991

C -1 6.647827 5.725782 0.043023

C 0 7.007559 4.625030 -0.926553

O 0 6.463675 3.514541 -0.873220

H 0 6.541253 5.290063 1.038106

N 0 7.945018 4.925890 -1.857158

C 0 8.440363 3.978381 -2.841268

C 0 9.786978 3.349164 -2.483638

O 0 10.609213 3.080845 -3.351041

H 0 7.723516 3.155264 -2.923811

H 0 8.405328 5.821715 -1.794654

N 0 9.984279 3.107470 -1.150775

C -1 11.056988 2.194006 -0.766054

C 0 10.580344 0.774556 -0.994848

O 0 9.549661 0.387075 -0.422523

H 0 11.951040 2.459923 -1.324949

H 0 9.148289 3.030325 -0.583886

N 0 11.272194 -0.000652 -1.838366

C -1 10.756977 -1.336994 -2.119989

C 0 11.798253 -1.930831 -3.075576

C 0 12.322583 -0.696491 -3.825334

C 0 12.378083 0.384736 -2.737439

H 0 11.371155 -2.688399 -3.736299

H 0 12.608872 -2.399080 -2.506096

H 0 11.609518 -0.395175 -4.599829

H 0 13.293352 -0.853929 -4.301047

H 0 13.333348 0.354878 -2.198038

H 0 12.211654 1.386645 -3.134629

H 0 9.768135 -1.257730 -2.588102

H 0 5.675197 6.142852 -0.236480

H 0 7.381760 6.534829 0.070358

H 0 8.547605 4.459381 -3.814703

H 0 11.254798 2.321247 0.301153

H 0 10.632106 -1.900636 -1.192501

N 0 -3.274383 -0.664845 2.282105

C 0 -3.369198 0.396106 1.274956

C 0 -2.487276 0.170420 0.039540

C 0 -1.012400 0.293022 0.321417

C 0 -0.356644 1.512681 0.105510

C 0 0.996767 1.668265 0.391600

C 0 1.707287 0.585102 0.946498

C 0 1.075593 -0.636409 1.153981

C 0 -0.277184 -0.781880 0.834392

O 0 3.032994 0.732686 1.267081

O 0 1.634604 2.859096 0.189920

H 0 3.205355 1.687450 1.315704

H 0 -0.760683 -1.741887 0.994264

H 0 -3.124948 1.348425 1.752329

H 0 -2.788712 0.907083 -0.711851

H 0 -0.893183 2.359864 -0.311256

H 0 1.643486 -1.458423 1.574235

H 0 -3.550720 -1.547578 1.851635

H 0 -2.724864 -0.815961 -0.367105

H 0 -4.407145 0.414583 0.962422

H 0 -2.334560 -0.738933 2.664705

H 0 2.445954 2.709874 -0.426730

H 0 -5.806397 1.628061 -0.232032

H 0 -6.607891 -0.802268 -0.145521

C 0 0.229293 0.292567 4.806678

C 0 -0.315821 1.414890 4.182108

C 0 -1.700947 1.584079 4.142704

C 0 -2.563238 0.639496 4.714147

C 0 -4.059091 0.753917 4.522253

C 0 -4.654191 -0.401954 3.709114

C 0 -1.998297 -0.456150 5.383231

C 0 -0.615091 -0.637307 5.417603

O 0 -5.766669 -0.239873 3.122377

H 0 0.333435 2.151012 3.719543

H 0 -2.128017 2.456656 3.657923

H 0 -4.281993 1.680602 3.989147

H 0 -4.436267 -1.399413 4.129369

H 0 -2.643860 -1.182461 5.868392

H 0 -0.200816 -1.514545 5.903673

H 0 1.303885 0.142038 4.813620

C 0 -4.816031 0.804924 5.865327

H 0 -4.485599 1.679492 6.427941

H 0 -5.893503 0.879755 5.697603

H 0 -4.633725 -0.093298 6.464709

**Int3S** (-8045.710373)

C -1 7.282120 0.461205 1.969177

C 0 6.051043 0.889310 2.778138

C 0 6.353527 0.926315 4.281286

C 0 5.511716 2.246615 2.308390

H 0 7.045996 0.376622 0.904679

H 0 8.096637 1.188785 2.065823

H 0 5.262441 0.144675 2.610503

H 0 7.163074 1.635124 4.496356

H 0 5.475083 1.241886 4.854941

H 0 6.664590 -0.056041 4.652668

H 0 4.574235 2.504016 2.815835

H 0 6.231696 3.047263 2.520786

H 0 5.349894 2.266967 1.226006

C -1 2.868013 -2.655364 6.140060

C 0 2.780139 -2.463230 4.620523

C 0 1.445534 -2.985633 4.075888

C 0 2.990414 -0.995850 4.229802

H 0 2.068719 -2.101631 6.648385

H 0 2.760010 -3.708753 6.419387

H 0 3.587360 -3.053222 4.162718

H 0 0.612367 -2.398025 4.475528

H 0 1.408149 -2.907984 2.984955

H 0 1.277860 -4.033407 4.347478

H 0 2.186072 -0.366733 4.627216

H 0 3.936501 -0.616103 4.627037

H 0 3.004269 -0.859581 3.144869

C -1 0.465920 0.417433 10.771286

C 0 -0.273207 0.689036 9.454093

C 0 -1.723978 0.196657 9.512447

C 0 -0.222544 2.175435 9.082044

H 0 -0.003735 0.960227 11.600455

H 0 0.448797 -0.649655 11.019641

H 0 0.240242 0.127981 8.661013

H 0 -2.289599 0.751419 10.270840

H 0 -2.225083 0.336382 8.549482

H 0 -1.776423 -0.865980 9.767725

H 0 -0.741436 2.779595 9.835960

H 0 0.808105 2.539489 9.017190

H 0 -0.710327 2.354553 8.118836

C -1 -3.085942 -4.133018 12.675992

C 0 -3.246743 -5.435222 11.872381

O 0 -4.234582 -5.614795 11.164692

C 0 -2.753138 -2.972407 11.727374

H 0 -4.040738 -3.954590 13.177748

H 0 -1.796381 -3.137203 11.224734

H 0 -3.523851 -2.882742 10.958264

H 0 -2.697512 -2.026986 12.274323

N 0 -2.238571 -6.363138 11.961330

C -1 -2.114064 -7.479946 11.012003

C 0 -0.920414 -7.289782 10.054658

C 0 -0.812764 -5.883404 9.504742

C 0 -1.812709 -5.335847 8.688495

C 0 0.285979 -5.084967 9.843535

C 0 -1.708709 -4.026325 8.221732

C 0 0.393113 -3.772567 9.381094

C 0 -0.606991 -3.240016 8.569296

H 0 -3.057787 -7.496112 10.464415

H 0 -1.017979 -8.020523 9.242214

H 0 0.010049 -7.536140 10.579886

H 0 -2.688257 -5.923354 8.428878

H 0 -1.406468 -6.088330 12.463599

H 0 1.068895 -5.499056 10.474399

H 0 -2.490016 -3.624960 7.584731

H 0 1.253126 -3.167748 9.653263

H 0 -0.532861 -2.217790 8.218889

C -1 0.152852 -6.400922 -0.188125

C 0 0.816227 -5.178369 -0.830946

C 0 0.392908 -5.010714 -2.295172

C 0 0.511989 -3.904961 -0.036609

H 0 0.489565 -6.540319 0.845096

H 0 -0.938038 -6.282255 -0.169357

H 0 1.903999 -5.336216 -0.811126

H 0 -0.687343 -4.835998 -2.364186

H 0 0.897403 -4.156499 -2.758145

H 0 0.626314 -5.904120 -2.884606

H 0 -0.559908 -3.672987 -0.061765

H 0 0.805172 -4.011068 1.013462

H 0 1.047699 -3.049912 -0.454682

C -1 -3.596932 -6.934023 3.872160

C 0 -4.266426 -5.609930 3.525490

S 0 -3.381135 -4.126615 4.141178

C 0 -2.055406 -3.978630 2.892284

H 0 -2.591583 -7.003858 3.444548

H 0 -3.513952 -7.052956 4.955600

H 0 -5.259464 -5.546945 3.974164

H 0 -4.393526 -5.496667 2.445908

H 0 -2.471729 -3.768011 1.904188

H 0 -1.422859 -3.142314 3.197947

H 0 -1.435204 -4.877410 2.858280

C -1 -8.593029 -6.747970 0.713177

C 0 -8.226375 -5.523089 1.513670

C 0 -6.979208 -4.904431 1.387963

C 0 -9.150985 -4.941346 2.393440

C 0 -6.666064 -3.734283 2.083731

C 0 -8.861545 -3.769830 3.083134

C 0 -7.624331 -3.134195 2.908088

O 0 -7.407645 -1.961245 3.553964

H 0 -9.033174 -7.527116 1.344782

H 0 -9.332336 -6.496927 -0.056822

H 0 -6.228239 -5.350477 0.739174

H 0 -10.126918 -5.402583 2.517932

H 0 -5.680890 -3.291985 2.000944

H 0 -9.589897 -3.309722 3.742490

H 0 -6.761664 -1.399628 3.048321

C -1 -3.717987 -4.902577 -2.924033

C 0 -3.546039 -3.963807 -1.727494

C 0 -4.787820 -3.113102 -1.451795

C 0 -4.641613 -2.202283 -0.253664

O 0 -3.605968 -2.192614 0.430823

O 0 -5.686760 -1.457140 -0.003556

H 0 -3.913797 -4.341099 -3.844313

H 0 -2.688925 -3.301214 -1.888814

H 0 -3.308599 -4.544037 -0.828468

H 0 -5.670871 -3.738654 -1.267288

H 0 -5.049995 -2.487980 -2.314780

C -1 2.463013 -1.588922 -6.455113

C 0 2.700797 -1.366347 -4.977000

C 0 1.635851 -1.429614 -4.068414

C 0 3.982231 -1.111168 -4.471514

C 0 1.840867 -1.249966 -2.700104

C 0 4.201391 -0.945959 -3.100785

C 0 3.128499 -1.020608 -2.208785

H 0 3.253359 -1.134492 -7.059268

H 0 2.440331 -2.659021 -6.694112

H 0 0.631951 -1.614812 -4.439780

H 0 4.821159 -1.041374 -5.159505

H 0 0.996705 -1.272613 -2.019629

H 0 5.201676 -0.730238 -2.733162

H 0 3.287741 -0.884951 -1.143145

C -1 2.557106 4.063971 -4.609102

C 0 3.034115 3.854975 -3.168740

C 0 3.501739 2.423785 -2.914573

N 0 3.955895 2.252625 -1.520146

H 0 3.360379 3.863311 -5.326473

H 0 1.725788 3.391303 -4.844410

H 0 2.230155 4.089640 -2.461377

H 0 3.856426 4.550697 -2.946208

H 0 4.288984 2.147720 -3.630203

H 0 2.673757 1.727628 -3.077534

H 0 4.804931 2.791284 -1.347409

H 0 4.209140 1.278687 -1.365801

C -1 -2.422008 0.948919 -5.153625

C 0 -1.953971 0.942343 -3.690835

C 0 -2.011263 -0.474592 -3.103273

C 0 -0.550529 1.548868 -3.559718

H 0 -1.753319 0.339425 -5.773556

H 0 -2.645474 1.569879 -3.109729

H 0 -3.038116 -0.858178 -3.092947

H 0 -1.409797 -1.169555 -3.700246

H 0 -1.628267 -0.498995 -2.079126

H 0 0.180191 0.970570 -4.135536

H 0 -0.533230 2.579488 -3.932220

H 0 -0.217213 1.560916 -2.518134

C -1 -8.783948 -2.890677 -3.160860

C 0 -9.193742 -3.982043 -2.176358

S 0 -10.363223 -3.426456 -0.880352

C 0 -9.276068 -2.261086 0.007512

H 0 -9.656912 -2.475199 -3.669071

H 0 -8.270798 -2.069547 -2.652972

H 0 -8.318965 -4.411103 -1.673699

H 0 -9.706246 -4.799455 -2.693039

H 0 -8.326520 -2.740855 0.242814

H 0 -9.110449 -1.351506 -0.569322

H 0 -9.773600 -2.006999 0.944015

C -1 -11.414108 -0.217160 -4.324858

C 0 -10.789153 0.548858 -3.182302

C 0 -9.939155 1.635233 -3.420653

C 0 -11.051190 0.211007 -1.849386

C 0 -9.359578 2.355102 -2.377922

C 0 -10.478568 0.918463 -0.792871

C 0 -9.617370 1.985826 -1.051682

O 0 -9.054935 2.641289 0.016113

H 0 -10.692879 -0.394436 -5.129497

H 0 -11.790328 -1.187089 -3.988136

H 0 -9.720238 1.927271 -4.445040

H 0 -11.688579 -0.640195 -1.630318

H 0 -8.702786 3.192063 -2.585238

H 0 -10.676592 0.638470 0.236384

H 0 -8.331776 3.225723 -0.310800

C -1 -5.818503 4.555090 -3.596513

C 0 -5.162701 3.551609 -2.653491

C 0 -5.966747 3.272324 -1.404211

O 0 -6.958038 3.943057 -1.081670

O 0 -5.525385 2.259227 -0.704857

H 0 -5.996374 5.515390 -3.108656

H 0 -4.173850 3.900718 -2.331002

H 0 -4.986362 2.586805 -3.142071

C -1 -9.497210 6.950521 -2.623697

C 0 -8.721921 7.153896 -1.316747

C 0 -9.394560 6.462635 -0.129160

O 0 -7.362379 6.741068 -1.446480

H 0 -9.592162 5.881706 -2.850886

H 0 -8.669678 8.231148 -1.112089

H 0 -10.405096 6.851065 0.035802

H 0 -9.477262 5.382688 -0.296404

H 0 -8.807850 6.620323 0.781040

H 0 -7.328390 5.769155 -1.400294

N 0 -7.724867 5.139755 5.940732

C 0 -6.812472 4.100623 5.422133

C 0 -5.468299 4.607865 4.900784

O 0 -4.543053 3.818337 4.702209

C 0 -7.612342 3.443572 4.275104

C 0 -9.075203 3.692642 4.657597

C 0 -9.037882 5.095924 5.273817

H 0 -6.578721 3.388760 6.219123

H 0 -7.359567 2.390895 4.160142

H 0 -7.377356 3.931335 3.324418

H 0 -9.399231 2.968649 5.413026

H 0 -9.755490 3.625621 3.805152

H 0 -9.101437 5.867266 4.494103

H 0 -9.846832 5.270571 5.985255

N 0 -5.416825 5.922334 4.598794

C -1 -4.225189 6.582967 4.088996

C 0 -4.252570 6.803698 2.573021

C 0 -4.114535 5.536825 1.712496

C 0 -4.347609 5.890512 0.239708

C 0 -2.743754 4.874665 1.899488

H 0 -3.369122 5.971101 4.381631

H 0 -3.436221 7.492817 2.313068

H 0 -5.185218 7.320375 2.311101

H 0 -4.883665 4.816662 2.022222

H 0 -5.357905 6.264716 0.050730

H 0 -3.637562 6.658185 -0.089649

H 0 -6.219377 6.477245 4.856003

H 0 -4.187894 5.011888 -0.391137

H 0 -2.600018 4.511721 2.919880

H 0 -2.631954 4.019432 1.226702

H 0 -1.936176 5.578354 1.661098

C -1 -0.160161 5.153834 5.413221

C 0 1.169964 4.439605 5.181643

S 0 1.329034 3.606318 3.553416

C 0 1.275393 5.054952 2.445647

H 0 -0.308321 5.965194 4.694826

H 0 -1.003505 4.463821 5.317755

H 0 1.309540 3.642013 5.917504

H 0 2.011321 5.130850 5.302615

H 0 0.286389 5.517859 2.441615

H 0 2.027767 5.792492 2.741161

H 0 1.506614 4.676654 1.448985

C -1 -0.794132 9.088016 -2.940147

C 0 -0.372695 7.612161 -2.863750

C 0 -1.277734 6.835643 -1.889822

C 0 1.111093 7.489352 -2.493768

C 0 -1.016780 5.327220 -1.829982

H 0 -0.672715 9.576173 -1.965623

H 0 -0.508050 7.169632 -3.862437

H 0 -2.323199 7.005370 -2.176489

H 0 -1.167010 7.265222 -0.883890

H 0 1.737690 8.058156 -3.189290

H 0 1.291313 7.882558 -1.485448

H 0 1.452672 6.450561 -2.511364

H 0 -0.034061 5.091338 -1.410234

H 0 -1.764190 4.832123 -1.202229

H 0 -1.067818 4.876759 -2.828225

C -1 -8.525046 6.609702 7.776827

C 0 -7.455774 5.707548 7.167112

O 0 -6.387637 5.499623 7.730625

H 0 -9.362014 6.011965 8.154252

H 0 -8.071336 7.142598 8.611380

H 0 -1.844449 9.187221 -3.233159

H 0 -0.186154 9.638048 -3.666004

H 0 2.212564 5.090399 -4.767040

H 0 3.823979 -2.292711 6.532545

H 0 1.512851 0.735226 10.714369

H 0 -2.018867 -8.425751 11.555806

H 0 -2.313093 -4.237021 13.446846

H 0 -4.187976 -7.768593 3.477926

H 0 -7.718356 -7.175526 0.213986

H 0 -8.100436 -3.295090 -3.917253

H 0 -4.556957 -5.590853 -2.771715

H 0 -2.818306 -5.503257 -3.084736

H 0 0.378127 -7.318070 -0.742865

H 0 1.505090 -1.164911 -6.771497

H 0 -2.425384 1.963412 -5.566620

H 0 -3.433449 0.539781 -5.253402

H 0 -5.179476 4.722266 -4.468043

H 0 -8.972146 7.432579 -3.452761

H 0 -10.506291 7.370814 -2.554706

H 0 -6.782550 4.181652 -3.954422

H 0 -12.258654 0.329665 -4.762301

H 0 -8.926698 7.324850 7.052959

H 0 -4.131011 7.546144 4.601910

H 0 -0.187776 5.588923 6.418249

O 0 7.143588 0.840311 -1.788548

O 0 -6.763865 1.888767 1.462666

H 0 7.909001 0.462571 -1.319999

H 0 6.948854 1.646067 -1.284529

H 0 -7.711311 1.824606 1.246251

H 0 -6.446034 1.027237 1.866676

C -1 5.110982 1.406465 8.853561

C 0 3.736480 1.969010 8.470160

C 0 3.858049 3.392266 7.914413

C 0 3.023825 1.052056 7.469092

H 0 5.024573 0.399669 9.276211

H 0 5.761454 1.342211 7.972617

H 0 3.125139 2.013586 9.382784

H 0 4.425698 3.393888 6.975862

H 0 2.873558 3.823801 7.706613

H 0 4.372727 4.054523 8.618832

H 0 2.034631 1.441135 7.207720

H 0 3.599632 0.962786 6.541732

H 0 2.889167 0.044143 7.874920

C -1 -7.139961 -6.830984 9.563996

C 0 -6.839700 -5.939733 8.375149

C 0 -5.874948 -4.924565 8.463065

C 0 -7.542096 -6.083185 7.170728

C 0 -5.644613 -4.065923 7.385277

C 0 -7.314554 -5.225261 6.093314

C 0 -6.368857 -4.202389 6.199369

H 0 -6.297687 -6.848089 10.259877

H 0 -8.016173 -6.464597 10.113212

H 0 -5.311925 -4.812586 9.383912

H 0 -8.285872 -6.871849 7.079994

H 0 -4.904777 -3.274869 7.478239

H 0 -7.879293 -5.341347 5.173279

H 0 -6.216882 -3.514202 5.374241

H 0 -7.361932 -7.855558 9.247603

H 0 5.610388 2.041841 9.592598

H 0 7.664799 -0.508116 2.304351

C -1 6.647996 5.725950 0.042983

C 0 7.072376 4.587026 -0.854289

O 0 6.716730 3.423614 -0.618798

H 0 7.018490 5.528496 1.052160

N 0 7.861795 4.894491 -1.908830

C 0 8.377058 3.896800 -2.834197

C 0 9.752289 3.328281 -2.473940

O 0 10.586123 3.105241 -3.343938

H 0 7.684935 3.048075 -2.854486

H 0 8.191413 5.843427 -2.003136

N 0 9.952551 3.074729 -1.145230

C -1 11.057030 2.194000 -0.766003

C 0 10.602004 0.763930 -0.973958

O 0 9.597925 0.359013 -0.367582

H 0 11.935096 2.474671 -1.342384

H 0 9.116127 2.964084 -0.581764

N 0 11.270959 0.001553 -1.847356

C -1 10.756966 -1.337000 -2.120001

C 0 11.777836 -1.920986 -3.103565

C 0 12.275964 -0.680877 -3.861900

C 0 12.351639 0.397361 -2.772190

H 0 11.339527 -2.679288 -3.756057

H 0 12.605212 -2.385707 -2.555759

H 0 11.542534 -0.381779 -4.618101

H 0 13.235685 -0.830856 -4.361778

H 0 13.319888 0.371278 -2.256324

H 0 12.170359 1.399862 -3.161370

H 0 9.756557 -1.262631 -2.564179

H 0 5.556314 5.736928 0.096626

H 0 7.003653 6.703179 -0.290510

H 0 8.450886 4.322292 -3.835202

H 0 11.266611 2.337023 0.296999

H 0 10.658276 -1.904365 -1.191870

N 0 -3.334422 -0.144134 2.199682

C 0 -0.553612 0.008310 5.871018

C 0 -0.998458 1.267662 5.469443

C 0 -3.307002 1.202741 1.547660

C 0 -2.565086 1.165978 0.208336

C 0 -2.307900 1.445673 5.016200

C 0 -3.194543 0.361749 4.964815

C 0 -1.075010 0.952143 0.341946

C 0 -4.557175 0.491515 4.305049

C 0 -0.200371 2.034512 0.183217

C 0 1.177765 1.883694 0.329914

C 0 -4.582486 -0.440416 3.072673

C 0 1.687106 0.631322 0.723056

C 0 -2.749769 -0.891756 5.415032

C 0 -1.437578 -1.072286 5.847867

C 0 0.834124 -0.457285 0.858952

C 0 -0.538382 -0.305899 0.648433

O 0 -5.691647 -0.360996 2.280201

O 0 3.030672 0.468120 0.948163

O 0 2.022733 2.936792 0.152157

H 0 -0.319445 2.110709 5.478235

H 0 -2.656751 2.425424 4.706104

H 0 3.403478 1.345568 1.130460

H 0 -4.686759 1.524923 3.968831

H 0 -1.181976 -1.178271 0.708506

H 0 -2.841707 1.898814 2.245181

H 0 -2.764161 2.117882 -0.290600

H 0 -4.391723 -1.474617 3.397855

H 0 -3.420799 -1.742981 5.399937

H 0 -0.578224 3.015524 -0.083962

H 0 -1.109008 -2.060310 6.148136

H 0 1.252668 -1.416663 1.135265

H 0 -3.355490 -0.902844 1.478359

H 0 -3.022742 0.396251 -0.421493

H 0 -4.340485 1.499338 1.397151

H 0 -2.492996 -0.274270 2.766639

H 0 2.791585 2.673152 -0.481164

H 0 0.471991 -0.130505 6.194643

C 0 -5.712052 0.157161 5.257951

H 0 -5.693510 0.847227 6.105468

H 0 -6.675789 0.243884 4.752155

H 0 -5.641329 -0.863708 5.644068

H 0 -6.075806 2.117743 0.169337

H 0 -5.631929 -0.997203 0.939438

**TS3S** (-8045.70503)

C -1 7.282033 0.461305 1.969303

C 0 6.107085 0.792497 2.884755

C 0 6.505959 0.628191 4.355805

C 0 5.565739 2.204216 2.618619

H 0 6.987700 0.473711 0.915697

H 0 8.100226 1.182457 2.087556

H 0 5.302463 0.081302 2.667913

H 0 7.338051 1.298480 4.605685

H 0 5.681483 0.865920 5.033497

H 0 6.828370 -0.397122 4.566857

H 0 4.633434 2.390894 3.163307

H 0 6.294281 2.962311 2.933691

H 0 5.390996 2.381801 1.550310

C -1 2.868012 -2.655418 6.139963

C 0 3.000521 -2.366218 4.642759

C 0 1.931304 -3.124350 3.850654

C 0 2.935786 -0.862866 4.342199

H 0 1.881504 -2.344677 6.514378

H 0 2.970698 -3.724163 6.356741

H 0 3.985930 -2.731393 4.319321

H 0 0.931895 -2.769475 4.125829

H 0 2.055430 -2.971656 2.776041

H 0 1.968372 -4.201811 4.045950

H 0 1.948478 -0.456377 4.569713

H 0 3.669761 -0.309068 4.936557

H 0 3.129532 -0.652725 3.288803

C -1 0.465876 0.417453 10.771274

C 0 -0.227885 0.705915 9.434752

C 0 -1.686797 0.234739 9.447415

C 0 -0.136767 2.194784 9.079653

H 0 -0.027611 0.954229 11.589843

H 0 0.436529 -0.652150 11.008873

H 0 0.301130 0.139271 8.654213

H 0 -2.272326 0.808471 10.175894

H 0 -2.149086 0.366519 8.464353

H 0 -1.762472 -0.823087 9.717524

H 0 -0.623352 2.805018 9.849974

H 0 0.904699 2.525560 9.002985

H 0 -0.635268 2.405841 8.129067

C -1 -3.085938 -4.133025 12.675982

C 0 -3.232042 -5.410762 11.831635

O 0 -4.177819 -5.544227 11.059098

C 0 -2.758094 -2.944787 11.760548

H 0 -4.045397 -3.974582 13.176535

H 0 -1.795956 -3.088250 11.261054

H 0 -3.524976 -2.847102 10.988837

H 0 -2.715693 -2.012835 12.331216

N 0 -2.257839 -6.369061 11.963737

C -1 -2.114063 -7.479944 11.011995

C 0 -0.906209 -7.282153 10.072092

C 0 -0.792983 -5.875033 9.523244

C 0 -1.775210 -5.333369 8.681708

C 0 0.294286 -5.070280 9.883877

C 0 -1.664490 -4.023872 8.215894

C 0 0.409345 -3.759509 9.417834

C 0 -0.573349 -3.232165 8.581450

H 0 -3.048602 -7.497662 10.449075

H 0 -0.988244 -8.012341 9.257376

H 0 0.016873 -7.525385 10.611444

H 0 -2.640343 -5.926869 8.401975

H 0 -1.453433 -6.132184 12.526485

H 0 1.063514 -5.478636 10.534850

H 0 -2.435272 -3.614745 7.570753

H 0 1.262553 -3.152562 9.705896

H 0 -0.496634 -2.213063 8.222495

C -1 0.152991 -6.401030 -0.188075

C 0 0.822106 -5.190098 -0.853973

C 0 0.446743 -5.086053 -2.336433

C 0 0.474685 -3.887640 -0.122450

H 0 0.449121 -6.486689 0.864183

H 0 -0.940229 -6.294693 -0.218604

H 0 1.910545 -5.331720 -0.791393

H 0 -0.632184 -4.932862 -2.448100

H 0 0.951894 -4.239754 -2.813671

H 0 0.717059 -5.996001 -2.883248

H 0 -0.601914 -3.681521 -0.163969

H 0 0.766796 -3.933117 0.932431

H 0 0.993912 -3.041596 -0.581513

C -1 -3.596926 -6.933979 3.872113

C 0 -3.887964 -5.994356 2.703469

S 0 -3.477314 -4.228482 2.997295

C 0 -1.659433 -4.310402 3.132401

H 0 -2.529128 -6.959366 4.109790

H 0 -4.143528 -6.621349 4.765007

H 0 -4.956885 -5.979241 2.474061

H 0 -3.356794 -6.316005 1.800036

H 0 -1.230220 -4.819344 2.265972

H 0 -1.296251 -3.281090 3.149878

H 0 -1.341872 -4.807810 4.051567

C -1 -8.592985 -6.747955 0.713185

C 0 -8.232548 -5.486875 1.455882

C 0 -7.058197 -4.785946 1.167907

C 0 -9.101353 -4.932763 2.405625

C 0 -6.783332 -3.550472 1.753673

C 0 -8.849063 -3.697587 2.991724

C 0 -7.705487 -2.974305 2.633979

O 0 -7.549143 -1.726292 3.148678

H 0 -9.112309 -7.464792 1.357306

H 0 -9.259773 -6.527965 -0.129962

H 0 -6.345638 -5.200871 0.458345

H 0 -10.014495 -5.463018 2.663726

H 0 -5.876030 -3.019968 1.501272

H 0 -9.543210 -3.258995 3.700236

H 0 -6.793946 -1.264116 2.720287

C -1 -3.717975 -4.902510 -2.923973

C 0 -3.387081 -3.887805 -1.829817

C 0 -4.555707 -2.949633 -1.540358

C 0 -4.305208 -1.960752 -0.419595

O 0 -3.186939 -1.903279 0.138383

O 0 -5.320016 -1.229287 -0.101943

H 0 -3.964698 -4.402444 -3.868083

H 0 -2.509852 -3.296554 -2.111077

H 0 -3.112858 -4.406534 -0.905660

H 0 -5.456043 -3.513477 -1.265247

H 0 -4.838460 -2.375167 -2.432023

C -1 2.463001 -1.588778 -6.455246

C 0 2.755556 -1.392006 -4.985438

C 0 1.709240 -1.395611 -4.050696

C 0 4.057045 -1.181828 -4.517646

C 0 1.953915 -1.194541 -2.693268

C 0 4.315061 -0.996518 -3.156062

C 0 3.263162 -1.007891 -2.239088

H 0 3.380103 -1.590480 -7.050014

H 0 1.940981 -2.535099 -6.633089

H 0 0.691051 -1.556877 -4.394300

H 0 4.880912 -1.168518 -5.226764

H 0 1.127249 -1.174005 -1.990233

H 0 5.331317 -0.821150 -2.812861

H 0 3.457028 -0.863277 -1.182667

C -1 2.557264 4.064039 -4.609070

C 0 3.122740 3.839467 -3.204271

C 0 3.625002 2.411377 -3.002165

N 0 4.173033 2.229820 -1.643991

H 0 3.317754 3.883326 -5.376807

H 0 1.719858 3.386047 -4.804413

H 0 2.361470 4.053104 -2.444398

H 0 3.948301 4.543238 -3.021380

H 0 4.366752 2.157000 -3.772594

H 0 2.798264 1.704678 -3.118794

H 0 5.006158 2.801523 -1.509083

H 0 4.485813 1.267016 -1.527148

C -1 -2.421987 0.948942 -5.153625

C 0 -1.958949 0.937190 -3.689133

C 0 -1.993339 -0.486981 -3.120331

C 0 -0.568264 1.569524 -3.542289

H 0 -1.740567 0.356209 -5.776183

H 0 -2.664506 1.545481 -3.103948

H 0 -3.008605 -0.897163 -3.150674

H 0 -1.353531 -1.155172 -3.709048

H 0 -1.645853 -0.523186 -2.084311

H 0 0.178324 1.009195 -4.116195

H 0 -0.567037 2.603539 -3.906427

H 0 -0.242307 1.578893 -2.498132

C -1 -8.784212 -2.890408 -3.161028

C 0 -8.965557 -3.626309 -1.836816

S 0 -9.838810 -2.666205 -0.546414

C 0 -8.603097 -1.354025 -0.260612

H 0 -9.750574 -2.617061 -3.591453

H 0 -8.208582 -1.970244 -3.026301

H 0 -8.002592 -3.942670 -1.421774

H 0 -9.568786 -4.529396 -1.975144

H 0 -7.603502 -1.783423 -0.201749

H 0 -8.639941 -0.591255 -1.038334

H 0 -8.838812 -0.897024 0.702597

C -1 -11.413796 -0.217465 -4.324651

C 0 -10.787732 0.569856 -3.196190

C 0 -9.731011 1.454699 -3.433739

C 0 -11.252106 0.456770 -1.879099

C 0 -9.145760 2.192803 -2.405318

C 0 -10.680292 1.184893 -0.838726

C 0 -9.609699 2.044786 -1.093805

O 0 -9.037515 2.701724 -0.030070

H 0 -10.670826 -0.483451 -5.082252

H 0 -11.864625 -1.143374 -3.955440

H 0 -9.349058 1.570712 -4.445191

H 0 -12.057288 -0.235606 -1.655067

H 0 -8.330945 2.875001 -2.614443

H 0 -11.030872 1.067894 0.181186

H 0 -8.305207 3.271723 -0.355534

C -1 -5.818452 4.555079 -3.596507

C 0 -5.061108 3.714096 -2.573643

C 0 -5.845774 3.464015 -1.307630

O 0 -6.908756 4.047403 -1.054353

O 0 -5.314384 2.568433 -0.513556

H 0 -6.100501 5.528393 -3.188825

H 0 -4.117462 4.193252 -2.285217

H 0 -4.780843 2.734141 -2.975927

C -1 -9.497194 6.950520 -2.623693

C 0 -8.719058 7.217622 -1.332960

C 0 -9.353071 6.534892 -0.119925

O 0 -7.347811 6.847920 -1.472423

H 0 -9.544315 5.873497 -2.825210

H 0 -8.700774 8.300916 -1.156662

H 0 -10.372533 6.897088 0.049640

H 0 -9.405520 5.449431 -0.260972

H 0 -8.759788 6.732642 0.778000

H 0 -7.282041 5.879445 -1.397506

N 0 -7.584701 5.049592 6.079344

C 0 -6.592138 4.030226 5.678341

C 0 -5.308255 4.575236 5.048928

O 0 -4.328151 3.839787 4.909694

C 0 -7.351949 3.135745 4.670695

C 0 -8.830027 3.352498 5.017745

C 0 -8.881076 4.831564 5.415390

H 0 -6.273969 3.472847 6.563846

H 0 -7.040261 2.094900 4.753690

H 0 -7.146285 3.449603 3.643773

H 0 -9.116356 2.733217 5.874678

H 0 -9.500141 3.117491 4.187317

H 0 -8.970758 5.471654 4.527251

H 0 -9.710227 5.066426 6.084325

N 0 -5.369463 5.853432 4.614682

C -1 -4.225183 6.582956 4.088983

C 0 -4.344063 6.946336 2.608120

C 0 -4.342853 5.770874 1.618365

C 0 -4.467633 6.319882 0.192573

C 0 -3.092312 4.894525 1.761543

H 0 -3.348272 5.958743 4.270632

H 0 -3.499979 7.607373 2.364657

H 0 -5.252129 7.545023 2.454881

H 0 -5.218320 5.139575 1.825222

H 0 -5.431790 6.801455 0.009130

H 0 -3.674295 7.049514 -0.008045

H 0 -6.204768 6.365375 4.859179

H 0 -4.356358 5.519853 -0.542996

H 0 -3.049976 4.403762 2.737214

H 0 -3.096317 4.116048 0.993469

H 0 -2.177188 5.486027 1.626525

C -1 -0.160233 5.153927 5.413309

C 0 1.106947 4.318750 5.284983

S 0 1.267249 3.395385 3.706172

C 0 1.331751 4.801556 2.542059

H 0 -0.201742 5.935644 4.649318

H 0 -1.058280 4.535760 5.307601

H 0 1.145275 3.555223 6.069832

H 0 1.996679 4.947385 5.408577

H 0 0.342805 5.239649 2.387336

H 0 2.017874 5.567123 2.917144

H 0 1.716777 4.410785 1.599165

C -1 -0.794036 9.087917 -2.939975

C 0 -0.418126 7.597971 -2.890653

C 0 -1.318828 6.842964 -1.896139

C 0 1.071557 7.425280 -2.566261

C 0 -1.108263 5.326216 -1.854808

H 0 -0.628665 9.560748 -1.964457

H 0 -0.597287 7.170415 -3.888932

H 0 -2.366292 7.050717 -2.147340

H 0 -1.161946 7.259112 -0.890783

H 0 1.694479 7.980596 -3.275856

H 0 1.294346 7.803819 -1.560873

H 0 1.381242 6.376804 -2.602385

H 0 -0.111087 5.058475 -1.491622

H 0 -1.834871 4.852105 -1.187288

H 0 -1.227574 4.879363 -2.848649

C -1 -8.525036 6.609690 7.776824

C 0 -7.377060 5.752065 7.247809

O 0 -6.299111 5.691505 7.827763

H 0 -9.314316 5.977470 8.198146

H 0 -8.124859 7.244338 8.566076

H 0 -1.848548 9.223432 -3.200893

H 0 -0.189954 9.626078 -3.677772

H 0 2.193078 5.088941 -4.730618

H 0 3.628134 -2.118386 6.720270

H 0 1.515716 0.731505 10.751510

H 0 -2.022634 -8.426713 11.554426

H 0 -2.318244 -4.253207 13.449420

H 0 -3.904754 -7.953221 3.615057

H 0 -7.705343 -7.242565 0.306636

H 0 -8.247462 -3.519786 -3.881572

H 0 -4.579118 -5.520299 -2.644836

H 0 -2.874684 -5.573534 -3.114351

H 0 0.411744 -7.335845 -0.692421

H 0 1.816466 -0.789967 -6.839364

H 0 -2.441531 1.966665 -5.558597

H 0 -3.425414 0.522776 -5.259784

H 0 -5.197041 4.717996 -4.481066

H 0 -9.003455 7.436488 -3.469722

H 0 -10.523769 7.325849 -2.553567

H 0 -6.734148 4.051193 -3.918806

H 0 -12.203720 0.354796 -4.826864

H 0 -8.975434 7.228158 6.994974

H 0 -4.102104 7.497792 4.680748

H 0 -0.197631 5.643945 6.392761

O 0 7.151993 0.835473 -1.717689

O 0 -6.730269 2.067667 1.539037

H 0 7.915940 0.411766 -1.287513

H 0 7.027735 1.638469 -1.187035

H 0 -7.648833 1.945528 1.234928

H 0 -6.378500 1.202246 1.866117

C -1 5.111018 1.406462 8.853573

C 0 3.993375 2.323193 8.337360

C 0 3.788864 3.520775 9.272020

C 0 4.282648 2.783698 6.903405

H 0 5.249043 0.540481 8.196822

H 0 6.063837 1.947734 8.895213

H 0 3.054831 1.748890 8.322806

H 0 4.694337 4.138256 9.315887

H 0 2.967681 4.158501 8.927417

H 0 3.556442 3.196723 10.292210

H 0 3.529801 3.489365 6.544588

H 0 5.255680 3.284499 6.841430

H 0 4.300267 1.936727 6.210305

C -1 -7.139957 -6.830980 9.564000

C 0 -6.811219 -6.163717 8.243822

C 0 -5.694600 -5.325970 8.117653

C 0 -7.627211 -6.354865 7.118657

C 0 -5.409811 -4.699083 6.901147

C 0 -7.347075 -5.725614 5.905434

C 0 -6.232878 -4.890499 5.790804

H 0 -6.335071 -6.682401 10.287155

H 0 -8.062071 -6.419371 9.992568

H 0 -5.056935 -5.161825 8.980938

H 0 -8.497459 -7.002852 7.198386

H 0 -4.542866 -4.049454 6.822669

H 0 -7.995273 -5.873589 5.046857

H 0 -6.015399 -4.393929 4.850687

H 0 -7.300407 -7.907431 9.434393

H 0 4.891718 1.035943 9.860166

H 0 7.691647 -0.532867 2.182191

C -1 6.648095 5.725859 0.042876

C 0 7.117896 4.565263 -0.801521

O 0 6.871760 3.394172 -0.473155

H 0 7.015806 5.584071 1.062188

N 0 7.813283 4.861834 -1.922522

C 0 8.340743 3.848711 -2.825949

C 0 9.728756 3.307168 -2.469263

O 0 10.557795 3.089769 -3.345844

H 0 7.664352 2.986804 -2.827346

H 0 8.062182 5.824387 -2.093795

N 0 9.941594 3.062640 -1.141896

C -1 11.056825 2.193892 -0.765948

C 0 10.611258 0.759118 -0.965909

O 0 9.619879 0.344476 -0.346043

H 0 11.928890 2.478274 -1.349806

H 0 9.110030 2.954332 -0.569371

N 0 11.270458 0.002650 -1.852230

C -1 10.756982 -1.336960 -2.119984

C 0 11.766370 -1.916548 -3.118269

C 0 12.253816 -0.673542 -3.878873

C 0 12.341066 0.401394 -2.786874

H 0 11.320931 -2.673431 -3.767618

H 0 12.600958 -2.382039 -2.582209

H 0 11.510942 -0.372897 -4.625215

H 0 13.207606 -0.820834 -4.390776

H 0 13.314809 0.373594 -2.281406

H 0 12.155634 1.404885 -3.171355

H 0 9.750546 -1.265204 -2.550790

H 0 5.555816 5.701690 0.086577

H 0 6.972791 6.698737 -0.330982

H 0 8.400417 4.256270 -3.834897

H 0 11.272081 2.343726 0.294882

H 0 10.672525 -1.906438 -1.191821

N 0 -3.110668 -0.116850 2.088408

C 0 -0.431472 -0.220717 5.934065

C 0 -0.936375 1.053013 5.675787

C 0 -3.032991 1.286817 1.569553

C 0 -2.272874 1.340347 0.238416

C 0 -2.223925 1.211300 5.161094

C 0 -3.022439 0.091345 4.899447

C 0 -0.792152 1.053777 0.358571

C 0 -4.379324 0.257710 4.239236

C 0 0.129538 2.100832 0.221593

C 0 1.500806 1.884408 0.349386

C 0 -4.367137 -0.483349 2.890823

C 0 1.958738 0.602774 0.704916

C 0 -2.512624 -1.188881 5.176611

C 0 -1.221922 -1.343102 5.681387

C 0 1.060139 -0.451951 0.815737

C 0 -0.305650 -0.235657 0.620034

O 0 -5.473687 -0.248539 2.101107

O 0 3.296104 0.382582 0.913976

O 0 2.399257 2.898786 0.190046

H 0 -0.324518 1.927421 5.858876

H 0 -2.624633 2.201702 4.966925

H 0 3.708892 1.250917 1.057006

H 0 -4.540962 1.323974 4.044382

H 0 -0.979834 -1.084713 0.644307

H 0 -2.555581 1.902935 2.332754

H 0 -2.421566 2.341916 -0.172284

H 0 -4.231807 -1.559405 3.066157

H 0 -3.118409 -2.071435 4.989853

H 0 -0.208593 3.105028 -0.011612

H 0 -0.836570 -2.337300 5.878351

H 0 1.439898 -1.436281 1.057412

H 0 -3.098339 -0.812029 1.274054

H 0 -2.756025 0.650845 -0.460086

H 0 -4.055301 1.627340 1.429334

H 0 -2.289393 -0.314673 2.665008

H 0 3.099908 2.633326 -0.518974

H 0 0.572427 -0.337636 6.323847

C 0 -5.518324 -0.253595 5.133837

H 0 -5.500311 0.284246 6.085328

H 0 -6.493890 -0.112509 4.666902

H 0 -5.414798 -1.322049 5.346612

H 0 -5.910368 2.408530 0.315658

H 0 -5.340730 -0.723352 0.988403

**Int4S** (-8045.70752)

C -1 7.282089 0.461309 1.969257

C 0 6.062515 0.784882 2.836281

C 0 6.395564 0.645251 4.325737

C 0 5.510958 2.184657 2.531340

H 0 7.027810 0.463789 0.905957

H 0 8.083732 1.195029 2.116242

H 0 5.276765 0.058062 2.594752

H 0 7.199382 1.336451 4.609654

H 0 5.529278 0.868023 4.954653

H 0 6.731525 -0.369803 4.563953

H 0 4.555160 2.361955 3.038010

H 0 6.213317 2.959036 2.865925

H 0 5.378019 2.344838 1.455050

C -1 2.868021 -2.655414 6.140001

C 0 2.940710 -2.546146 4.610446

C 0 1.807928 -3.337114 3.944026

C 0 2.924699 -1.084467 4.147128

H 0 1.921619 -2.249842 6.518249

H 0 2.928288 -3.697002 6.473001

H 0 3.894856 -2.990351 4.292148

H 0 0.834995 -2.900432 4.195146

H 0 1.905395 -3.320807 2.853203

H 0 1.799803 -4.384258 4.266022

H 0 1.971230 -0.604406 4.392372

H 0 3.721640 -0.508952 4.626706

H 0 3.060527 -0.999477 3.065616

C -1 0.465904 0.417379 10.771319

C 0 -0.224895 0.722988 9.436115

C 0 -1.673299 0.220487 9.428831

C 0 -0.168265 2.221009 9.113096

H 0 -0.044969 0.925578 11.598029

H 0 0.455058 -0.657481 10.984003

H 0 0.322074 0.188990 8.646137

H 0 -2.269581 0.754969 10.178270

H 0 -2.139639 0.379602 8.451522

H 0 -1.728498 -0.847684 9.659646

H 0 -0.687183 2.802155 9.884775

H 0 0.864010 2.582420 9.062420

H 0 -0.653795 2.435938 8.156316

C -1 -3.085929 -4.133011 12.675994

C 0 -3.228056 -5.403662 11.820792

O 0 -4.161267 -5.523072 11.031278

C 0 -2.728394 -2.944327 11.771645

H 0 -4.053232 -3.967077 13.158426

H 0 -1.758054 -3.095565 11.290225

H 0 -3.478904 -2.837369 10.985251

H 0 -2.688124 -2.014585 12.346038

N 0 -2.262751 -6.369533 11.962837

C -1 -2.114068 -7.479958 11.012000

C 0 -0.894450 -7.290397 10.086174

C 0 -0.773861 -5.888289 9.526264

C 0 -1.748599 -5.352708 8.672314

C 0 0.311797 -5.082071 9.888067

C 0 -1.634718 -4.046622 8.197528

C 0 0.432580 -3.776055 9.410323

C 0 -0.543785 -3.254176 8.563122

H 0 -3.042062 -7.492033 10.437934

H 0 -0.966552 -8.027829 9.276960

H 0 0.021582 -7.529376 10.639315

H 0 -2.609918 -5.949952 8.389255

H 0 -1.466666 -6.142942 12.541418

H 0 1.075266 -5.485597 10.548747

H 0 -2.401694 -3.639934 7.546193

H 0 1.285210 -3.168176 9.698166

H 0 -0.462012 -2.238165 8.195671

C -1 0.152944 -6.401059 -0.188081

C 0 0.768198 -5.153253 -0.834717

C 0 0.432720 -5.073867 -2.328894

C 0 0.324722 -3.875983 -0.112831

H 0 0.420613 -6.473184 0.871981

H 0 -0.942434 -6.366525 -0.253947

H 0 1.860935 -5.229274 -0.740123

H 0 -0.649664 -4.986442 -2.477236

H 0 0.904564 -4.201394 -2.792922

H 0 0.772235 -5.967815 -2.863473

H 0 -0.760995 -3.737243 -0.168751

H 0 0.604172 -3.900713 0.946118

H 0 0.791125 -2.996279 -0.563235

C -1 -3.596926 -6.934041 3.872157

C 0 -4.102177 -5.918636 2.852211

S 0 -3.597268 -4.184468 3.171216

C 0 -1.785870 -4.301369 2.992355

H 0 -2.503763 -6.976445 3.891517

H 0 -3.953358 -6.680609 4.874025

H 0 -5.194211 -5.877966 2.847908

H 0 -3.780507 -6.177088 1.837140

H 0 -1.520193 -4.848809 2.085236

H 0 -1.405430 -3.282378 2.903415

H 0 -1.320702 -4.776625 3.858754

C -1 -8.593002 -6.747945 0.713169

C 0 -8.283177 -5.483119 1.471935

C 0 -7.124027 -4.749185 1.207088

C 0 -9.180891 -4.960883 2.412487

C 0 -6.889379 -3.513621 1.807890

C 0 -8.966963 -3.727078 3.017902

C 0 -7.834707 -2.975572 2.686484

O 0 -7.707774 -1.730922 3.225797

H 0 -9.136723 -7.469470 1.331428

H 0 -9.218624 -6.535867 -0.163075

H 0 -6.390724 -5.139068 0.505055

H 0 -10.083820 -5.516997 2.651493

H 0 -5.997036 -2.954854 1.562834

H 0 -9.681050 -3.314523 3.722267

H 0 -6.939844 -1.272159 2.829289

C -1 -3.717959 -4.902525 -2.923980

C 0 -3.395050 -3.923498 -1.792120

C 0 -4.557603 -2.980846 -1.493721

C 0 -4.324481 -1.992131 -0.362267

O 0 -3.199798 -1.957687 0.214023

O 0 -5.313278 -1.249394 -0.058094

H 0 -3.942111 -4.370131 -3.855270

H 0 -2.507796 -3.333172 -2.043159

H 0 -3.139470 -4.473276 -0.878929

H 0 -5.462659 -3.544583 -1.233570

H 0 -4.830992 -2.397258 -2.382419

C -1 2.463009 -1.588776 -6.455269

C 0 2.734070 -1.363628 -4.984015

C 0 1.683605 -1.381473 -4.056623

C 0 4.033015 -1.152176 -4.504365

C 0 1.919572 -1.201944 -2.693066

C 0 4.281933 -0.987453 -3.139176

C 0 3.223949 -1.018646 -2.227240

H 0 3.252251 -1.157373 -7.077602

H 0 2.410004 -2.659420 -6.687128

H 0 0.667345 -1.530744 -4.409578

H 0 4.860738 -1.117366 -5.208501

H 0 1.088027 -1.188059 -1.996263

H 0 5.294633 -0.805401 -2.788548

H 0 3.409354 -0.883426 -1.166551

C -1 2.557264 4.064041 -4.609068

C 0 3.096190 3.839324 -3.193292

C 0 3.597837 2.412279 -2.981772

N 0 4.111963 2.229674 -1.610130

H 0 3.332863 3.885994 -5.362033

H 0 1.725939 3.383604 -4.820721

H 0 2.319423 4.049078 -2.448485

H 0 3.916028 4.545147 -2.993172

H 0 4.359116 2.161072 -3.733992

H 0 2.776025 1.703731 -3.120177

H 0 4.947848 2.792401 -1.457812

H 0 4.407868 1.263502 -1.483570

C -1 -2.421978 0.948946 -5.153626

C 0 -1.954744 0.948808 -3.690283

C 0 -2.003708 -0.467317 -3.101753

C 0 -0.556937 1.568008 -3.557533

H 0 -1.748655 0.342368 -5.771450

H 0 -2.651547 1.572221 -3.110636

H 0 -3.025951 -0.860204 -3.106239

H 0 -1.387374 -1.156458 -3.690658

H 0 -1.638125 -0.493948 -2.071864

H 0 0.180806 0.993346 -4.128174

H 0 -0.546120 2.597123 -3.934996

H 0 -0.226393 1.587073 -2.515024

C -1 -8.783930 -2.890690 -3.160838

C 0 -8.932173 -3.602383 -1.819329

S 0 -9.793584 -2.630498 -0.530075

C 0 -8.569616 -1.300082 -0.279946

H 0 -9.760947 -2.633013 -3.577428

H 0 -8.213140 -1.963727 -3.054355

H 0 -7.957597 -3.898932 -1.417420

H 0 -9.526731 -4.515201 -1.928746

H 0 -7.560432 -1.710260 -0.239699

H 0 -8.635098 -0.541938 -1.060538

H 0 -8.795407 -0.840992 0.685150

C -1 -11.414114 -0.217163 -4.324867

C 0 -10.813511 0.588958 -3.196846

C 0 -9.796751 1.518319 -3.437804

C 0 -11.261337 0.446677 -1.876917

C 0 -9.230093 2.268929 -2.408540

C 0 -10.706434 1.185827 -0.835047

C 0 -9.671066 2.086133 -1.093455

O 0 -9.106280 2.751788 -0.029717

H 0 -10.667799 -0.447523 -5.091067

H 0 -11.823144 -1.162823 -3.957709

H 0 -9.430996 1.658797 -4.452107

H 0 -12.035710 -0.279377 -1.651359

H 0 -8.444175 2.983804 -2.619044

H 0 -11.039983 1.043669 0.187425

H 0 -8.363812 3.307914 -0.357621

C -1 -5.818467 4.555071 -3.596489

C 0 -5.124595 3.615911 -2.616397

C 0 -5.911816 3.389398 -1.349030

O 0 -6.946879 4.013804 -1.081466

O 0 -5.414020 2.463235 -0.567096

H 0 -6.010220 5.534288 -3.153350

H 0 -4.137394 3.997112 -2.328968

H 0 -4.938869 2.629080 -3.055924

C -1 -9.497207 6.950520 -2.623696

C 0 -8.699482 7.215878 -1.342581

C 0 -9.345128 6.571903 -0.114266

O 0 -7.340328 6.803958 -1.481977

H 0 -9.580815 5.872123 -2.805568

H 0 -8.648471 8.300904 -1.184683

H 0 -10.351276 6.968515 0.058224

H 0 -9.433434 5.486610 -0.238732

H 0 -8.737791 6.763832 0.775483

H 0 -7.308387 5.832770 -1.425507

N 0 -7.583093 5.051968 6.078865

C 0 -6.596524 4.027462 5.676005

C 0 -5.325317 4.562696 5.011183

O 0 -4.376756 3.803945 4.794538

C 0 -7.372154 3.111163 4.699035

C 0 -8.847329 3.356716 5.040317

C 0 -8.875558 4.844619 5.404843

H 0 -6.261001 3.489132 6.566908

H 0 -7.074300 2.069334 4.815812

H 0 -7.165098 3.389284 3.662188

H 0 -9.143984 2.761671 5.910777

H 0 -9.520152 3.113403 4.214427

H 0 -8.941027 5.465991 4.501473

H 0 -9.707317 5.111004 6.058095

N 0 -5.360846 5.860728 4.644713

C -1 -4.225182 6.582951 4.088985

C 0 -4.314868 6.819435 2.579722

C 0 -4.145875 5.575966 1.692377

C 0 -4.336561 5.976462 0.225439

C 0 -2.779776 4.909739 1.901733

H 0 -3.328828 6.013152 4.342689

H 0 -3.535061 7.545742 2.309137

H 0 -5.274495 7.301543 2.351138

H 0 -4.923657 4.846717 1.958264

H 0 -5.342796 6.351630 0.017071

H 0 -3.621419 6.758572 -0.055087

H 0 -6.179550 6.379418 4.926604

H 0 -4.148305 5.121662 -0.428699

H 0 -2.681467 4.490122 2.905393

H 0 -2.636149 4.094673 1.186836

H 0 -1.965635 5.627853 1.739437

C -1 -0.160125 5.153945 5.413304

C 0 1.050762 4.252639 5.209341

S 0 1.121294 3.409210 3.580719

C 0 1.317795 4.866627 2.498365

H 0 -0.173748 5.975739 4.691427

H 0 -1.093418 4.593680 5.300939

H 0 1.044027 3.444032 5.945993

H 0 1.984850 4.805373 5.353722

H 0 0.386142 5.430480 2.413507

H 0 2.109056 5.517443 2.882821

H 0 1.613883 4.486289 1.519915

C -1 -0.794040 9.087957 -2.939984

C 0 -0.386252 7.607133 -2.890987

C 0 -1.274054 6.830567 -1.901516

C 0 1.105418 7.465999 -2.561257

C 0 -1.030133 5.318929 -1.863317

H 0 -0.642989 9.563410 -1.963314

H 0 -0.552201 7.177157 -3.890498

H 0 -2.324756 7.015669 -2.157594

H 0 -1.130925 7.247870 -0.894487

H 0 1.719191 8.035618 -3.267484

H 0 1.315890 7.847463 -1.554291

H 0 1.436556 6.424283 -2.597859

H 0 -0.032327 5.069837 -1.489427

H 0 -1.754227 4.828132 -1.205597

H 0 -1.129668 4.874169 -2.860352

C -1 -8.525033 6.609685 7.776820

C 0 -7.381121 5.742281 7.255920

O 0 -6.310507 5.666523 7.847397

H 0 -9.328579 5.985105 8.182098

H 0 -8.127109 7.232111 8.577079

H 0 -1.850346 9.201149 -3.205083

H 0 -0.198953 9.640313 -3.674751

H 0 2.192755 5.087820 -4.736389

H 0 3.682552 -2.098484 6.616262

H 0 1.509269 0.752131 10.768648

H 0 -2.035150 -8.427595 11.554753

H 0 -2.334478 -4.264545 13.463455

H 0 -3.964875 -7.934077 3.617135

H 0 -7.681146 -7.233795 0.352014

H 0 -8.255994 -3.529053 -3.879542

H 0 -4.591215 -5.517801 -2.678702

H 0 -2.879309 -5.577832 -3.120181

H 0 0.486901 -7.318414 -0.684211

H 0 1.509598 -1.143945 -6.756360

H 0 -2.432519 1.962209 -5.569916

H 0 -3.430083 0.531545 -5.252254

H 0 -5.196842 4.693397 -4.485503

H 0 -8.995695 7.405504 -3.482179

H 0 -10.510645 7.359579 -2.551449

H 0 -6.778907 4.144478 -3.921013

H 0 -12.231506 0.324165 -4.817693

H 0 -8.956059 7.241292 6.994582

H 0 -4.158386 7.545176 4.608532

H 0 -0.143731 5.593468 6.417157

O 0 7.152588 0.836540 -1.733051

O 0 -6.842166 2.085782 1.524678

H 0 7.918590 0.426336 -1.293482

H 0 7.005448 1.638234 -1.206380

H 0 -7.765265 1.989738 1.222158

H 0 -6.532731 1.233463 1.900644

C -1 5.110906 1.406557 8.853556

C 0 3.798558 2.061015 8.410063

C 0 3.771863 3.547690 8.785534

C 0 3.572273 1.876697 6.905986

H 0 5.129347 0.341011 8.599965

H 0 5.965617 1.880029 8.355835

H 0 2.972759 1.564774 8.938742

H 0 4.571912 4.091163 8.268079

H 0 2.820320 4.012359 8.505185

H 0 3.912779 3.691880 9.862009

H 0 2.689075 2.424229 6.571268

H 0 4.422754 2.270549 6.336983

H 0 3.447808 0.823823 6.635811

C -1 -7.139967 -6.830993 9.563989

C 0 -6.841454 -6.165341 8.235784

C 0 -5.662164 -5.431819 8.042986

C 0 -7.744299 -6.264006 7.166723

C 0 -5.398807 -4.817953 6.814096

C 0 -7.488056 -5.644744 5.943731

C 0 -6.310545 -4.915864 5.762407

H 0 -6.354712 -6.617109 10.292574

H 0 -8.094557 -6.481538 9.973685

H 0 -4.964765 -5.330952 8.868995

H 0 -8.664591 -6.828689 7.299963

H 0 -4.481044 -4.253549 6.674027

H 0 -8.202411 -5.717799 5.129437

H 0 -6.116483 -4.421301 4.816376

H 0 -7.219167 -7.918932 9.450990

H 0 5.259931 1.498714 9.934729

H 0 7.693407 -0.525111 2.206971

C -1 6.648101 5.725871 0.042885

C 0 7.104422 4.567962 -0.811638

O 0 6.830795 3.399662 -0.498705

H 0 7.031457 5.581400 1.055963

N 0 7.820387 4.864306 -1.919578

C 0 8.343394 3.852602 -2.826762

C 0 9.729857 3.307490 -2.470388

O 0 10.559139 3.088330 -3.346161

H 0 7.664233 2.992966 -2.829010

H 0 8.087540 5.824344 -2.076929

N 0 9.942139 3.063483 -1.142466

C -1 11.056829 2.193896 -0.765948

C 0 10.610156 0.759614 -0.966208

O 0 9.617277 0.346286 -0.347400

H 0 11.929058 2.478005 -1.349517

H 0 9.110447 2.954370 -0.570564

N 0 11.270259 0.002599 -1.851329

C -1 10.756983 -1.336965 -2.119984

C 0 11.767367 -1.916471 -3.117151

C 0 12.255341 -0.673428 -3.877319

C 0 12.341423 0.401591 -2.785298

H 0 11.322629 -2.673389 -3.766927

H 0 12.601497 -2.381849 -2.580264

H 0 11.513113 -0.372950 -4.624350

H 0 13.209641 -0.820574 -4.388318

H 0 13.314797 0.374219 -2.279097

H 0 12.155945 1.405017 -3.169948

H 0 9.750982 -1.264951 -2.551798

H 0 5.556696 5.701403 0.104207

H 0 6.966380 6.700945 -0.332253

H 0 8.403719 4.261996 -3.835027

H 0 11.271705 2.343519 0.294960

H 0 10.671368 -1.906531 -1.191909

N 0 -3.201007 -0.152728 2.091827

C 0 -0.413262 -0.221833 5.895186

C 0 -0.933845 1.047345 5.647422

C 0 -3.141350 1.247392 1.560080

C 0 -2.381522 1.295115 0.229305

C 0 -2.231887 1.198445 5.157008

C 0 -3.028321 0.072972 4.913489

C 0 -0.897067 1.029662 0.353381

C 0 -4.402102 0.229446 4.284101

C 0 0.010884 2.086055 0.202599

C 0 1.384952 1.887133 0.326694

C 0 -4.411599 -0.507568 2.937919

C 0 1.859728 0.614926 0.696338

C 0 -2.506005 -1.202783 5.185750

C 0 -1.204236 -1.349042 5.663726

C 0 0.974439 -0.448462 0.822837

C 0 -0.394497 -0.251056 0.627489

O 0 -5.564348 -0.252290 2.191377

O 0 3.200139 0.410353 0.902265

O 0 2.265899 2.913063 0.152123

H 0 -0.325666 1.924713 5.821146

H 0 -2.639010 2.187336 4.968152

H 0 3.601853 1.279795 1.061397

H 0 -4.575998 1.295016 4.096363

H 0 -1.059307 -1.107019 0.658206

H 0 -2.674007 1.879178 2.316918

H 0 -2.545160 2.290386 -0.191964

H 0 -4.299035 -1.585942 3.100104

H 0 -3.110537 -2.089540 5.017218

H 0 -0.339659 3.083420 -0.040793

H 0 -0.811945 -2.341560 5.856365

H 0 1.364974 -1.426800 1.075790

H 0 -3.188899 -0.882311 1.272184

H 0 -2.856754 0.591410 -0.459758

H 0 -4.166555 1.575936 1.413933

H 0 -2.363756 -0.335695 2.650709

H 0 2.996208 2.639918 -0.521530

H 0 0.601494 -0.330945 6.261284

C 0 -5.517634 -0.298582 5.199794

H 0 -5.483160 0.231426 6.155005

H 0 -6.505733 -0.159890 4.758329

H 0 -5.400403 -1.367666 5.402309

H 0 -6.002525 2.337777 0.264064

H 0 -5.442560 -0.671543 1.219031

**Int5S** (-8045.704401)

C -1 7.281855 0.461279 1.969265

C 0 5.951851 0.677818 2.698418

C 0 6.077433 0.383070 4.197507

C 0 5.417413 2.098754 2.468852

H 0 7.164983 0.576452 0.888789

H 0 8.038642 1.183414 2.299511

H 0 5.221061 -0.024898 2.278070

H 0 6.806017 1.056615 4.666463

H 0 5.119922 0.515919 4.708753

H 0 6.412962 -0.644388 4.375898

H 0 4.425051 2.237275 2.914046

H 0 6.084535 2.840378 2.927158

H 0 5.370508 2.347406 1.402139

C -1 2.868036 -2.655453 6.139948

C 0 3.182016 -2.443410 4.651369

C 0 2.679407 -3.625118 3.811829

C 0 2.601278 -1.126853 4.120964

H 0 1.785834 -2.694856 6.314537

H 0 3.291272 -3.596726 6.507274

H 0 4.275508 -2.395442 4.547184

H 0 1.589719 -3.721067 3.898612

H 0 2.917450 -3.487965 2.751393

H 0 3.122910 -4.572086 4.138355

H 0 1.507651 -1.165829 4.081307

H 0 2.875913 -0.278034 4.755030

H 0 2.957987 -0.909867 3.110751

C -1 0.465962 0.417472 10.771284

C 0 -0.201745 0.743205 9.428887

C 0 -1.639247 0.212525 9.376253

C 0 -0.173208 2.249709 9.146511

H 0 -0.075090 0.892472 11.598908

H 0 0.473599 -0.662583 10.955489

H 0 0.373088 0.241579 8.637712

H 0 -2.264913 0.722374 10.119010

H 0 -2.082845 0.379287 8.389922

H 0 -1.678920 -0.860142 9.588371

H 0 -0.739896 2.796006 9.910060

H 0 0.849365 2.640144 9.149689

H 0 -0.624015 2.475911 8.175434

C -1 -3.085943 -4.133017 12.675972

C 0 -3.251358 -5.436855 11.875587

O 0 -4.243961 -5.620784 11.175732

C 0 -2.703003 -2.987212 11.726632

H 0 -4.049448 -3.931495 13.151024

H 0 -1.739441 -3.178577 11.246227

H 0 -3.453145 -2.884572 10.938990

H 0 -2.636419 -2.038963 12.267450

N 0 -2.236202 -6.358463 11.955654

C -1 -2.114097 -7.479961 11.011984

C 0 -0.926995 -7.293994 10.045588

C 0 -0.820335 -5.894883 9.475203

C 0 -1.828580 -5.348779 8.667315

C 0 0.294380 -5.103867 9.778400

C 0 -1.712316 -4.050328 8.171642

C 0 0.411662 -3.802354 9.287991

C 0 -0.593957 -3.272023 8.481229

H 0 -3.061403 -7.502151 10.470573

H 0 -1.027435 -8.034598 9.242301

H 0 0.007112 -7.532980 10.567722

H 0 -2.719527 -5.926107 8.438665

H 0 -1.403531 -6.081260 12.455616

H 0 1.083101 -5.516177 10.403128

H 0 -2.501011 -3.643931 7.547096

H 0 1.284897 -3.204950 9.532919

H 0 -0.513926 -2.259430 8.104700

C -1 0.152881 -6.400938 -0.188037

C 0 0.741791 -5.130744 -0.812944

C 0 0.335824 -4.988272 -2.284428

C 0 0.338776 -3.883564 -0.019353

H 0 0.470187 -6.514971 0.854601

H 0 -0.944569 -6.364690 -0.201763

H 0 1.837332 -5.213016 -0.774326

H 0 -0.749602 -4.866990 -2.374897

H 0 0.807806 -4.112095 -2.740760

H 0 0.623429 -5.870387 -2.866970

H 0 -0.751953 -3.766735 0.011804

H 0 0.701328 -3.932710 1.013810

H 0 0.754011 -2.983517 -0.479538

C -1 -3.596866 -6.933982 3.872085

C 0 -3.684092 -5.939286 2.717252

S 0 -3.232276 -4.207352 3.131475

C 0 -1.441765 -4.374968 3.442112

H 0 -2.574939 -7.021450 4.253363

H 0 -4.246437 -6.627397 4.696176

H 0 -4.714269 -5.862715 2.362338

H 0 -3.061227 -6.253193 1.871790

H 0 -0.948367 -4.845513 2.589073

H 0 -1.046808 -3.364315 3.563332

H 0 -1.241894 -4.942568 4.353656

C -1 -8.592981 -6.747971 0.713181

C 0 -8.211921 -5.581887 1.588535

C 0 -7.005059 -4.898263 1.416101

C 0 -9.090918 -5.106746 2.572519

C 0 -6.690198 -3.766930 2.171940

C 0 -8.800535 -3.973059 3.323149

C 0 -7.606697 -3.275082 3.105909

O 0 -7.387234 -2.140279 3.825592

H 0 -9.318429 -6.431673 -0.045601

H 0 -7.722126 -7.161902 0.195881

H 0 -6.296525 -5.245768 0.667433

H 0 -10.033638 -5.622225 2.734498

H 0 -5.749907 -3.254388 2.014859

H 0 -9.492210 -3.598560 4.069900

H 0 -6.785880 -1.544225 3.334018

C -1 -3.717957 -4.902509 -2.924011

C 0 -3.585901 -3.980323 -1.710293

C 0 -4.784455 -3.039127 -1.555883

C 0 -4.699053 -2.086828 -0.382467

O 0 -3.493562 -1.894910 0.087798

O 0 -5.712729 -1.539681 0.074156

H 0 -3.777235 -4.325359 -3.853443

H 0 -2.673312 -3.382644 -1.789670

H 0 -3.477805 -4.574456 -0.794703

H 0 -5.724479 -3.591239 -1.449050

H 0 -4.902754 -2.420615 -2.456395

C -1 2.463025 -1.588781 -6.455163

C 0 2.719801 -1.364240 -4.980735

C 0 1.662511 -1.390579 -4.060910

C 0 4.013639 -1.145291 -4.490612

C 0 1.887506 -1.215000 -2.694616

C 0 4.251768 -0.983668 -3.123187

C 0 3.187498 -1.026140 -2.218735

H 0 3.243908 -1.132797 -7.070483

H 0 2.441017 -2.659255 -6.692899

H 0 0.649750 -1.543974 -4.422469

H 0 4.846325 -1.101679 -5.188333

H 0 1.050491 -1.206922 -2.003631

H 0 5.260993 -0.794055 -2.766127

H 0 3.364557 -0.895975 -1.155914

C -1 2.557186 4.064006 -4.609087

C 0 3.052675 3.837043 -3.177907

C 0 3.561241 2.413696 -2.958199

N 0 4.029833 2.226165 -1.570916

H 0 3.357648 3.897163 -5.338265

H 0 1.739266 3.377009 -4.850140

H 0 2.249271 4.034096 -2.458739

H 0 3.858113 4.549942 -2.947424

H 0 4.350256 2.174745 -3.685577

H 0 2.751150 1.698757 -3.129468

H 0 4.863711 2.785168 -1.391751

H 0 4.313415 1.257196 -1.439067

C -1 -2.422028 0.948893 -5.153634

C 0 -1.969380 0.959226 -3.685194

C 0 -2.017846 -0.452097 -3.084989

C 0 -0.573930 1.581177 -3.541787

H 0 -1.742142 0.338882 -5.760814

H 0 -2.673091 1.584339 -3.116273

H 0 -3.036049 -0.856130 -3.106039

H 0 -1.381063 -1.139790 -3.653382

H 0 -1.673089 -0.461308 -2.047525

H 0 0.170552 1.004075 -4.101344

H 0 -0.560683 2.608005 -3.924729

H 0 -0.255545 1.607085 -2.495616

C -1 -8.783931 -2.890691 -3.160798

C 0 -9.119181 -4.007581 -2.176750

S 0 -10.287222 -3.528478 -0.848832

C 0 -9.235078 -2.353016 0.069819

H 0 -9.684916 -2.523167 -3.657410

H 0 -8.312306 -2.044251 -2.654010

H 0 -8.213413 -4.397316 -1.697473

H 0 -9.600831 -4.845805 -2.689939

H 0 -8.248791 -2.782734 0.242007

H 0 -9.142494 -1.403756 -0.457367

H 0 -9.711502 -2.188523 1.037569

C -1 -11.414085 -0.217202 -4.324906

C 0 -10.787679 0.544026 -3.179932

C 0 -9.982179 1.664604 -3.416116

C 0 -11.000795 0.166608 -1.849004

C 0 -9.400897 2.381778 -2.373031

C 0 -10.427839 0.873438 -0.791373

C 0 -9.614507 1.977053 -1.049567

O 0 -9.054363 2.640638 0.016335

H 0 -10.676768 -0.455642 -5.099041

H 0 -11.852943 -1.156710 -3.978467

H 0 -9.801603 1.986718 -4.438898

H 0 -11.602292 -0.711181 -1.632408

H 0 -8.777562 3.244754 -2.578045

H 0 -10.593452 0.568106 0.236370

H 0 -8.345504 3.237319 -0.318954

C -1 -5.818479 4.555069 -3.596519

C 0 -5.166957 3.541003 -2.663290

C 0 -5.968996 3.265079 -1.415134

O 0 -6.963350 3.929218 -1.093793

O 0 -5.517430 2.259098 -0.706011

H 0 -5.985863 5.514135 -3.102713

H 0 -4.175932 3.881074 -2.339491

H 0 -4.998810 2.577537 -3.157396

C -1 -9.497205 6.950490 -2.623703

C 0 -8.710781 7.155129 -1.322977

C 0 -9.386338 6.483237 -0.125868

O 0 -7.357607 6.723235 -1.453995

H 0 -9.608581 5.881042 -2.840655

H 0 -8.643669 8.233334 -1.128183

H 0 -10.390043 6.887373 0.043008

H 0 -9.486231 5.403235 -0.284985

H 0 -8.790828 6.638612 0.778819

H 0 -7.340025 5.751182 -1.412496

N 0 -7.596890 5.051075 6.076045

C 0 -6.610621 4.032411 5.661162

C 0 -5.320055 4.578891 5.047386

O 0 -4.355295 3.831800 4.873652

C 0 -7.370426 3.169045 4.626855

C 0 -8.848977 3.389119 4.969262

C 0 -8.890710 4.859415 5.399907

H 0 -6.304046 3.451162 6.535370

H 0 -7.064928 2.124789 4.685196

H 0 -7.155860 3.509295 3.609921

H 0 -9.145114 2.753006 5.810358

H 0 -9.516307 3.178797 4.129844

H 0 -8.970726 5.519928 4.525774

H 0 -9.722192 5.085214 6.069192

N 0 -5.360960 5.870160 4.656974

C -1 -4.225204 6.582971 4.088977

C 0 -4.264425 6.684700 2.561372

C 0 -3.999124 5.373555 1.804100

C 0 -4.209394 5.596310 0.302913

C 0 -2.588188 4.839876 2.081914

H 0 -3.321406 6.069320 4.423893

H 0 -3.512800 7.424495 2.250478

H 0 -5.238837 7.090772 2.259878

H 0 -4.721831 4.620465 2.145636

H 0 -5.226392 5.924627 0.067445

H 0 -3.517439 6.357388 -0.076370

H 0 -6.193073 6.386082 4.901177

H 0 -4.008579 4.670883 -0.244010

H 0 -2.467567 4.536035 3.123547

H 0 -2.368613 3.967090 1.462083

H 0 -1.833454 5.601466 1.846623

C -1 -0.159933 5.153850 5.413260

C 0 1.176423 4.428937 5.284996

S 0 1.399812 3.513055 3.709014

C 0 1.435126 4.912558 2.538632

H 0 -0.277717 5.914111 4.635815

H 0 -0.999751 4.456774 5.326992

H 0 1.283478 3.676018 6.067292

H 0 2.014201 5.125447 5.406702

H 0 0.455539 5.388276 2.457452

H 0 2.180712 5.651451 2.848235

H 0 1.716415 4.492582 1.571742

C -1 -0.794041 9.087967 -2.940022

C 0 -0.337008 7.622413 -2.872948

C 0 -1.220309 6.819730 -1.900435

C 0 1.150541 7.533165 -2.508259

C 0 -0.917312 5.319502 -1.841686

H 0 -0.680426 9.573709 -1.963355

H 0 -0.464790 7.181997 -3.873571

H 0 -2.270064 6.961531 -2.187716

H 0 -1.121952 7.251197 -0.893955

H 0 1.760762 8.121866 -3.201804

H 0 1.324246 7.923780 -1.497800

H 0 1.517117 6.503001 -2.534000

H 0 0.069449 5.110667 -1.417381

H 0 -1.652394 4.802264 -1.218077

H 0 -0.951413 4.867377 -2.839665

C -1 -8.525030 6.609677 7.776808

C 0 -7.388107 5.735190 7.254754

O 0 -6.318389 5.647728 7.845915

H 0 -9.329661 5.990030 8.187698

H 0 -8.121835 7.233326 8.573522

H 0 -1.847629 9.163414 -3.228344

H 0 -0.202381 9.656433 -3.665401

H 0 2.186965 5.084954 -4.742723

H 0 3.275216 -1.843428 6.750992

H 0 1.501025 0.774163 10.802796

H 0 -2.012387 -8.422484 11.560124

H 0 -2.334941 -4.247489 13.466745

H 0 -3.911587 -7.926944 3.532151

H 0 -9.055238 -7.553825 1.292459

H 0 -8.087809 -3.252982 -3.926895

H 0 -4.621452 -5.519605 -2.859636

H 0 -2.859724 -5.575767 -3.004102

H 0 0.462480 -7.298371 -0.734375

H 0 1.499737 -1.168338 -6.759471

H 0 -2.428581 1.959589 -5.576113

H 0 -3.429056 0.530781 -5.259923

H 0 -5.181920 4.721833 -4.469890

H 0 -8.970868 7.417553 -3.460461

H 0 -10.499964 7.385175 -2.552216

H 0 -6.787267 4.192215 -3.952782

H 0 -12.210589 0.363401 -4.805642

H 0 -8.956852 7.240050 6.994001

H 0 -4.213164 7.586765 4.526972

H 0 -0.231249 5.655144 6.384602

O 0 7.168077 0.855831 -1.804461

O 0 -6.802994 1.992311 1.502615

H 0 7.925168 0.462131 -1.335575

H 0 6.980384 1.655953 -1.288699

H 0 -7.742719 1.923599 1.246538

H 0 -6.532668 1.166717 1.959493

C -1 5.110884 1.406509 8.853606

C 0 3.715655 1.966724 8.549374

C 0 3.723852 3.499933 8.542970

C 0 3.172872 1.423209 7.222720

H 0 5.097377 0.312182 8.901761

H 0 5.824304 1.695416 8.072355

H 0 3.040232 1.636063 9.349941

H 0 4.390648 3.877739 7.758473

H 0 2.724335 3.903960 8.349711

H 0 4.070388 3.903152 9.500440

H 0 2.135165 1.729181 7.061231

H 0 3.761116 1.797902 6.377673

H 0 3.203921 0.329701 7.193907

C -1 -7.139977 -6.831009 9.564024

C 0 -6.706225 -6.077986 8.322821

C 0 -5.798342 -5.011879 8.414553

C 0 -7.220762 -6.402480 7.060290

C 0 -5.435057 -4.287391 7.277316

C 0 -6.856502 -5.680537 5.921516

C 0 -5.961704 -4.613901 6.026414

H 0 -6.350821 -6.817231 10.320093

H 0 -8.031481 -6.370915 10.008210

H 0 -5.374352 -4.761923 9.381628

H 0 -7.921209 -7.229689 6.969189

H 0 -4.743939 -3.453798 7.371573

H 0 -7.277044 -5.937415 4.954046

H 0 -5.684648 -4.045297 5.146242

H 0 -7.392544 -7.870269 9.331172

H 0 5.491068 1.782494 9.809993

H 0 7.682032 -0.541437 2.150232

C -1 6.648093 5.725945 0.042912

C 0 7.089039 4.578976 -0.835148

O 0 6.766504 3.411536 -0.568872

H 0 6.983054 5.528753 1.064168

N 0 7.848456 4.886089 -1.911062

C 0 8.370745 3.888634 -2.833454

C 0 9.748329 3.325927 -2.471618

O 0 10.583245 3.106993 -3.341878

H 0 7.683854 3.035881 -2.858552

H 0 8.151779 5.841297 -2.027097

N 0 9.948707 3.070956 -1.143489

C -1 11.056874 2.193901 -0.765950

C 0 10.604701 0.762632 -0.973360

O 0 9.602889 0.354083 -0.365949

H 0 11.932590 2.476401 -1.345037

H 0 9.112275 2.956545 -0.580117

N 0 11.272103 0.001653 -1.849295

C -1 10.756977 -1.336953 -2.119980

C 0 11.771903 -1.919321 -3.110869

C 0 12.266792 -0.677939 -3.869335

C 0 12.349043 0.397869 -2.777678

H 0 11.329275 -2.675769 -3.762634

H 0 12.602041 -2.386056 -2.568981

H 0 11.529360 -0.376603 -4.620742

H 0 13.223638 -0.827541 -4.374830

H 0 13.319705 0.369319 -2.266391

H 0 12.167593 1.401444 -3.164107

H 0 9.753639 -1.262684 -2.557279

H 0 5.555029 5.749021 0.060737

H 0 7.023303 6.698204 -0.283575

H 0 8.445504 4.315916 -3.833477

H 0 11.268575 2.338109 0.296416

H 0 10.664973 -1.905591 -1.191925

N 0 -3.381760 -0.237196 2.077857

C 0 -0.333213 -0.093913 5.861360

C 0 -0.880802 1.165344 5.618112

C 0 -3.282641 1.173093 1.627949

C 0 -2.520099 1.282051 0.298747

C 0 -2.191320 1.289467 5.153143

C 0 -2.974843 0.150647 4.930611

C 0 -1.034011 1.015913 0.405989

C 0 -4.375684 0.282492 4.355139

C 0 -0.128625 2.069620 0.224110

C 0 1.248364 1.877710 0.327476

C 0 -4.445989 -0.539881 3.056176

C 0 1.732710 0.611737 0.703813

C 0 -2.425170 -1.113214 5.200704

C 0 -1.110151 -1.234664 5.649013

C 0 0.851790 -0.451040 0.863687

C 0 -0.520526 -0.258448 0.690562

O 0 -5.719327 -0.372438 2.434557

O 0 3.077776 0.410120 0.891632

O 0 2.117800 2.909518 0.129131

H 0 -0.280721 2.055369 5.765897

H 0 -2.620409 2.269043 4.966671

H 0 3.481795 1.278575 1.046835

H 0 -4.544369 1.336225 4.108511

H 0 -1.180078 -1.115320 0.764882

H 0 -2.801770 1.788810 2.394576

H 0 -2.676287 2.294384 -0.084324

H 0 -4.316595 -1.601458 3.297481

H 0 -3.018479 -2.011478 5.053274

H 0 -0.484288 3.063456 -0.024602

H 0 -0.694509 -2.220873 5.827686

H 0 1.249430 -1.421090 1.137009

H 0 -3.494203 -1.208670 0.898596

H 0 -2.988325 0.610597 -0.427118

H 0 -4.295375 1.547340 1.487837

H 0 -2.496497 -0.499457 2.511944

H 0 2.867719 2.630633 -0.516796

H 0 0.690515 -0.186298 6.204666

C 0 -5.444785 -0.152688 5.369048

H 0 -5.336368 0.434073 6.285024

H 0 -6.454644 -0.002834 4.981030

H 0 -5.351814 -1.211276 5.629400

H 0 -6.052178 2.145506 0.160553

H 0 -5.693793 -0.808304 1.528591

**TS4S** (-8045.68022)

C -1 7.281928 0.461241 1.969279

C 0 5.968781 0.633082 2.741781

C 0 6.174531 0.439999 4.248658

C 0 5.333344 1.999772 2.452889

H 0 7.114127 0.504430 0.890723

H 0 7.998756 1.250574 2.225505

H 0 5.270973 -0.142850 2.398363

H 0 6.861235 1.197446 4.647882

H 0 5.229564 0.523803 4.794058

H 0 6.602360 -0.543932 4.469155

H 0 4.347998 2.096458 2.921892

H 0 5.965149 2.807974 2.842358

H 0 5.225154 2.184559 1.378944

C -1 2.867849 -2.655514 6.139768

C 0 3.157870 -2.055061 4.756683

C 0 2.731280 -3.006426 3.631822

C 0 2.494039 -0.685622 4.570336

H 0 1.815613 -2.934717 6.237535

H 0 3.460263 -3.562022 6.307358

H 0 4.244528 -1.908677 4.681517

H 0 1.647438 -3.172328 3.662415

H 0 2.977477 -2.586940 2.651284

H 0 3.220300 -3.982844 3.720035

H 0 1.401258 -0.771565 4.573511

H 0 2.779793 0.008617 5.367021

H 0 2.782650 -0.246015 3.611903

C -1 0.465913 0.417582 10.771232

C 0 0.997412 0.986112 9.447350

C 0 0.588181 0.109912 8.258753

C 0 0.528676 2.431025 9.240043

H 0 -0.629964 0.377597 10.764531

H 0 0.833390 -0.599132 10.946408

H 0 2.094766 0.992848 9.502302

H 0 -0.501108 0.078073 8.147490

H 0 0.996707 0.495151 7.318044

H 0 0.936873 -0.921239 8.372420

H 0 -0.565268 2.477849 9.177176

H 0 0.842441 3.078216 10.066135

H 0 0.933793 2.849083 8.312684

C -1 -3.085913 -4.133135 12.675958

C 0 -3.292303 -5.507630 11.986354

O 0 -4.368241 -5.823466 11.490399

C 0 -4.258970 -3.194495 12.422861

H 0 -2.942882 -4.308560 13.750314

H 0 -4.340409 -2.967084 11.357307

H 0 -5.198139 -3.654102 12.737310

H 0 -4.123178 -2.255407 12.967126

N 0 -2.209210 -6.366467 11.979485

C -1 -2.114078 -7.479858 11.011985

C 0 -1.284941 -7.090729 9.748858

C 0 -1.360676 -5.606279 9.466452

C 0 -2.557375 -5.006679 9.049763

C 0 -0.270008 -4.780648 9.766176

C 0 -2.668178 -3.620128 8.984474

C 0 -0.375745 -3.389733 9.687709

C 0 -1.583596 -2.805130 9.312054

H 0 -3.143775 -7.709639 10.739075

H 0 -1.652190 -7.678197 8.899762

H 0 -0.236657 -7.371919 9.897190

H 0 -3.423930 -5.618825 8.822013

H 0 -1.329282 -5.947977 12.250844

H 0 0.667778 -5.232486 10.081013

H 0 -3.601671 -3.176902 8.660871

H 0 0.481361 -2.767750 9.925814

H 0 -1.673898 -1.726429 9.249360

C -1 0.153006 -6.401042 -0.188100

C 0 0.913306 -5.258626 -0.874264

C 0 0.229059 -4.842473 -2.181621

C 0 1.055166 -4.052766 0.060816

H 0 0.671525 -6.734105 0.717609

H 0 -0.853446 -6.072063 0.098484

H 0 1.922269 -5.620175 -1.119669

H 0 -0.775561 -4.455391 -1.978443

H 0 0.792638 -4.054839 -2.691760

H 0 0.129596 -5.689188 -2.870034

H 0 0.066445 -3.652975 0.316293

H 0 1.560020 -4.322705 0.994814

H 0 1.628345 -3.251086 -0.414122

C -1 -3.596647 -6.933961 3.872232

C 0 -3.359184 -6.420273 2.453034

S 0 -2.721851 -4.701029 2.321572

C 0 -1.070462 -4.875512 3.070703

H 0 -2.673772 -6.942929 4.459987

H 0 -4.330052 -6.314068 4.395941

H 0 -4.302695 -6.390344 1.901692

H 0 -2.674079 -7.075565 1.904219

H 0 -0.456941 -5.583344 2.510242

H 0 -0.605750 -3.888851 3.028141

H 0 -1.136705 -5.171284 4.118182

C -1 -8.593123 -6.747965 0.713290

C 0 -7.929615 -5.548226 1.349796

C 0 -6.659783 -5.105860 0.963914

C 0 -8.592628 -4.809330 2.341035

C 0 -6.067395 -3.974284 1.533259

C 0 -8.024142 -3.675323 2.909690

C 0 -6.756836 -3.235790 2.500719

O 0 -6.266864 -2.115347 3.090686

H 0 -8.995503 -7.432163 1.467904

H 0 -9.428638 -6.430594 0.078508

H 0 -6.111478 -5.654943 0.201163

H 0 -9.582912 -5.121137 2.661661

H 0 -5.074620 -3.666506 1.230097

H 0 -8.549202 -3.106212 3.669311

H 0 -5.427024 -1.778905 2.685249

C -1 -3.717956 -4.902506 -2.923940

C 0 -3.677342 -3.853188 -1.813768

C 0 -4.907809 -2.943573 -1.828353

C 0 -4.970325 -1.939954 -0.695826

O 0 -3.829351 -1.769908 -0.066645

O 0 -6.022970 -1.367574 -0.405972

H 0 -3.760250 -4.433807 -3.913894

H 0 -2.777117 -3.237628 -1.908380

H 0 -3.597713 -4.341888 -0.836273

H 0 -5.835363 -3.523630 -1.769003

H 0 -4.967733 -2.383070 -2.771018

C -1 2.463009 -1.588733 -6.455196

C 0 2.688201 -1.419438 -4.967407

C 0 1.610067 -1.419529 -4.071785

C 0 3.975963 -1.232267 -4.447031

C 0 1.807186 -1.240277 -2.700549

C 0 4.184644 -1.065065 -3.076444

C 0 3.098876 -1.073131 -2.196025

H 0 1.582732 -2.205636 -6.655723

H 0 2.300944 -0.619535 -6.943127

H 0 0.603344 -1.565152 -4.453890

H 0 4.827419 -1.225576 -5.123228

H 0 0.956978 -1.233323 -2.025799

H 0 5.191720 -0.911293 -2.697111

H 0 3.253166 -0.945164 -1.129255

C -1 2.557126 4.063986 -4.609194

C 0 3.009795 3.777953 -3.171001

C 0 3.572115 2.367360 -3.003057

N 0 4.044267 2.138360 -1.622095

H 0 3.389928 3.962016 -5.313223

H 0 1.773110 3.363472 -4.915914

H 0 2.175610 3.906424 -2.470778

H 0 3.775658 4.510196 -2.875628

H 0 4.375519 2.194993 -3.734521

H 0 2.794160 1.627242 -3.211967

H 0 4.820762 2.763561 -1.406163

H 0 4.435959 1.200061 -1.558100

C -1 -2.422081 0.948985 -5.153472

C 0 -2.029159 0.952860 -3.668915

C 0 -2.082529 -0.462567 -3.080221

C 0 -0.650718 1.595381 -3.463491

H 0 -1.710541 0.352948 -5.738190

H 0 -2.769891 1.556737 -3.124960

H 0 -3.090240 -0.883499 -3.161958

H 0 -1.399974 -1.133792 -3.614678

H 0 -1.803347 -0.471970 -2.023437

H 0 0.121870 1.039597 -4.006917

H 0 -0.639182 2.629712 -3.826394

H 0 -0.366497 1.603075 -2.407183

C -1 -8.784546 -2.890274 -3.161119

C 0 -9.098259 -3.951136 -2.108643

S 0 -10.310484 -3.422383 -0.840398

C 0 -9.348758 -2.097047 -0.029991

H 0 -9.695558 -2.558362 -3.663709

H 0 -8.308850 -2.018087 -2.706214

H 0 -8.189836 -4.274125 -1.587290

H 0 -9.543744 -4.838538 -2.569544

H 0 -8.345520 -2.449525 0.212324

H 0 -9.291778 -1.209209 -0.659519

H 0 -9.868850 -1.848226 0.896588

C -1 -11.413299 -0.217540 -4.324435

C 0 -10.679865 0.398909 -3.155652

C 0 -9.301789 0.628113 -3.199121

C 0 -11.359751 0.751251 -1.981251

C 0 -8.617429 1.192529 -2.121927

C 0 -10.697779 1.335284 -0.904553

C 0 -9.322098 1.566155 -0.974547

O 0 -8.696668 2.138706 0.113358

H 0 -11.885207 -1.164385 -4.039319

H 0 -12.206570 0.442745 -4.693197

H 0 -8.742870 0.345464 -4.086927

H 0 -12.429136 0.571234 -1.910498

H 0 -7.541381 1.308454 -2.162919

H 0 -11.230553 1.620688 -0.003830

H 0 -7.973277 2.725859 -0.216175

C -1 -5.817640 4.555212 -3.595789

C 0 -4.933659 3.891294 -2.533238

C 0 -5.725991 3.165993 -1.472036

O 0 -6.717852 3.672420 -0.918782

O 0 -5.281271 1.974123 -1.178876

H 0 -6.475122 5.302179 -3.147682

H 0 -4.339407 4.653943 -2.013777

H 0 -4.230859 3.182464 -2.977310

C -1 -9.497206 6.950486 -2.623754

C 0 -9.013112 6.441502 -1.269135

C 0 -9.696724 5.133451 -0.882689

O 0 -7.590201 6.314180 -1.311862

H 0 -9.315954 6.195637 -3.396804

H 0 -9.250951 7.200290 -0.506836

H 0 -10.780721 5.269307 -0.816196

H 0 -9.502969 4.360397 -1.633145

H 0 -9.341677 4.762622 0.082938

H 0 -7.332237 5.394069 -1.121474

N 0 -7.886824 5.319476 5.749494

C 0 -7.099024 4.265362 5.080910

C 0 -5.710600 4.681424 4.602166

O 0 -4.932220 3.825539 4.167095

C 0 -7.964701 3.864157 3.862556

C 0 -9.388797 4.238431 4.287692

C 0 -9.181453 5.534639 5.080799

H 0 -6.950548 3.429408 5.770743

H 0 -7.838397 2.812029 3.605055

H 0 -7.667040 4.452869 2.987590

H 0 -9.804274 3.467722 4.945610

H 0 -10.066977 4.366421 3.440744

H 0 -9.128850 6.400929 4.407409

H 0 -9.973299 5.721219 5.807998

N 0 -5.454024 6.003613 4.602783

C -1 -4.224800 6.582975 4.089082

C 0 -4.463533 7.554495 2.927275

C 0 -5.246240 6.979264 1.735686

C 0 -5.418508 8.041271 0.643499

C 0 -4.596386 5.713094 1.165579

H 0 -3.600993 5.743667 3.777559

H 0 -3.481488 7.906439 2.582393

H 0 -4.989752 8.443980 3.301426

H 0 -6.247177 6.702806 2.097180

H 0 -5.868810 8.956449 1.045711

H 0 -4.444792 8.313134 0.215768

H 0 -6.154411 6.603215 5.013782

H 0 -6.059417 7.667403 -0.159805

H 0 -4.536390 4.913259 1.907347

H 0 -5.182591 5.342811 0.320988

H 0 -3.579504 5.925639 0.811290

C -1 -0.160139 5.153586 5.413531

C 0 -0.928212 3.833715 5.349404

S 0 -0.636570 2.857454 3.823926

C 0 1.151239 2.525867 3.958488

H 0 0.920864 4.986048 5.422041

H 0 -0.397981 5.784632 4.552111

H 0 -2.009194 4.006848 5.347267

H 0 -0.697308 3.208277 6.219642

H 0 1.746535 3.420887 3.767399

H 0 1.396009 2.117010 4.941207

H 0 1.384226 1.781709 3.197665

C -1 -0.794916 9.087910 -2.940807

C 0 -0.570365 7.570874 -2.850049

C 0 -1.446707 6.948650 -1.746342

C 0 0.918228 7.259944 -2.647624

C 0 -1.381251 5.419757 -1.662179

H 0 -0.493244 9.580658 -2.008502

H 0 -0.880582 7.125303 -3.807386

H 0 -2.488236 7.254001 -1.912013

H 0 -1.155202 7.379899 -0.778066

H 0 1.525001 7.718462 -3.436082

H 0 1.268236 7.655925 -1.686209

H 0 1.119746 6.185626 -2.657008

H 0 -0.381447 5.065785 -1.392967

H 0 -2.073561 5.039252 -0.903900

H 0 -1.648177 4.957135 -2.619308

C -1 -8.524729 6.609098 7.776298

C 0 -7.574862 5.662604 7.048141

O 0 -6.555909 5.235152 7.577945

H 0 -9.444803 6.087349 8.062164

H 0 -8.021739 6.950905 8.679804

H 0 -1.849467 9.325321 -3.116176

H 0 -0.208923 9.527993 -3.754575

H 0 2.156803 5.077283 -4.703294

H 0 3.103820 -1.946584 6.941104

H 0 0.771485 1.036087 11.621266

H 0 -1.682847 -8.355849 11.504543

H 0 -2.151527 -3.701698 12.297995

H 0 -3.978323 -7.960480 3.838056

H 0 -7.889428 -7.308987 0.091228

H 0 -8.095788 -3.292961 -3.914044

H 0 -4.598134 -5.548521 -2.828167

H 0 -2.829678 -5.540299 -2.895496

H 0 0.048342 -7.266855 -0.851154

H 0 3.326096 -2.056107 -6.938494

H 0 -2.425288 1.962925 -5.568989

H 0 -3.418521 0.519253 -5.301991

H 0 -5.191636 5.046771 -4.344921

H 0 -8.957227 7.859792 -2.901651

H 0 -10.569501 7.175045 -2.602670

H 0 -6.435589 3.810656 -4.107562

H 0 -10.732875 -0.419623 -5.155926

H 0 -8.806911 7.467388 7.159519

H 0 -3.699084 7.097268 4.903232

H 0 -0.419230 5.699587 6.327201

O 0 7.090317 0.836600 -1.722801

O 0 -6.331413 0.934565 0.855668

H 0 7.874957 0.436409 -1.307996

H 0 6.961058 1.648737 -1.206117

H 0 -7.292510 1.108759 0.818703

H 0 -6.213456 -0.019513 0.615103

C -1 5.110891 1.406499 8.853575

C 0 4.374869 2.669682 8.390264

C 0 5.328365 3.868119 8.322868

C 0 3.686322 2.442629 7.039565

H 0 4.427641 0.553811 8.931923

H 0 5.898545 1.134429 8.140406

H 0 3.595697 2.898403 9.130595

H 0 6.126058 3.686089 7.592390

H 0 4.801809 4.779688 8.020490

H 0 5.801317 4.057680 9.292332

H 0 3.117750 3.325227 6.726457

H 0 4.422884 2.227166 6.256885

H 0 2.994325 1.596547 7.085940

C -1 -7.139811 -6.830937 9.564107

C 0 -6.845610 -5.990069 8.342422

C 0 -6.204862 -4.748521 8.472883

C 0 -7.234699 -6.402035 7.060983

C 0 -5.990432 -3.936386 7.359106

C 0 -7.013011 -5.594867 5.942266

C 0 -6.395173 -4.352456 6.089007

H 0 -8.049011 -6.478834 10.066826

H 0 -7.300877 -7.879227 9.295111

H 0 -5.872790 -4.428067 9.456444

H 0 -7.725265 -7.364987 6.940792

H 0 -5.507810 -2.970360 7.474465

H 0 -7.333145 -5.920602 4.956918

H 0 -6.244680 -3.712917 5.226456

H 0 -6.322175 -6.772416 10.287737

H 0 5.580522 1.553962 9.831852

H 0 7.756337 -0.500370 2.190415

C -1 6.647949 5.725774 0.042910

C 0 7.085634 4.557952 -0.809171

O 0 6.797097 3.394729 -0.492890

H 0 7.058940 5.595820 1.047268

N 0 7.808600 4.845215 -1.915846

C 0 8.327898 3.829603 -2.819629

C 0 9.717410 3.290335 -2.467505

O 0 10.536799 3.051185 -3.347161

H 0 7.650349 2.968532 -2.812577

H 0 8.085943 5.802521 -2.072147

N 0 9.945546 3.071300 -1.136770

C -1 11.056736 2.193862 -0.765948

C 0 10.600855 0.763700 -0.975236

O 0 9.591233 0.360883 -0.376918

H 0 11.930162 2.475900 -1.348869

H 0 9.118968 2.968450 -0.556481

N 0 11.274485 -0.000730 -1.843687

C -1 10.756984 -1.336953 -2.119978

C 0 11.778579 -1.922947 -3.101504

C 0 12.282971 -0.683364 -3.856429

C 0 12.358353 0.393281 -2.765069

H 0 11.339535 -2.678661 -3.756515

H 0 12.602677 -2.391510 -2.552054

H 0 11.552665 -0.380722 -4.614229

H 0 13.243694 -0.835600 -4.353695

H 0 13.325335 0.365155 -2.246976

H 0 12.179227 1.396030 -3.154504

H 0 9.757919 -1.259071 -2.566683

H 0 5.558900 5.699896 0.133282

H 0 6.954732 6.696645 -0.352109

H 0 8.380799 4.231341 -3.831541

H 0 11.273829 2.337325 0.295330

H 0 10.653919 -1.905801 -1.193103

H 0 -5.760605 1.562321 -0.334566

O 0 -3.906580 -1.013373 2.295218

N 0 -3.722144 1.603255 2.382204

C 0 -1.587349 -3.163749 6.014130

C 0 -2.937417 -3.222948 5.673950

C 0 -3.145424 2.303046 1.232700

C 0 -2.483238 1.424373 0.156786

C 0 -3.592582 -2.085849 5.198593

C 0 -2.902929 -0.877438 5.043523

C 0 -1.043615 1.032900 0.417070

C 0 -3.560167 0.374424 4.489407

C 0 -0.030045 1.994557 0.275634

C 0 1.309929 1.671044 0.469061

C 0 -3.220640 0.543651 3.012654

C 0 1.647731 0.359284 0.855961

C 0 -1.543084 -0.831950 5.377805

C 0 -0.893533 -1.963734 5.865059

C 0 0.658942 -0.606177 1.003949

C 0 -0.678482 -0.272960 0.764154

O 0 2.963590 0.048372 1.092518

O 0 2.305990 2.602886 0.362709

H 0 -3.493371 -4.145851 5.792330

H 0 -4.640589 -2.145702 4.929920

H 0 3.442902 0.895044 1.085408

H 0 -1.442966 -1.038070 0.850190

H 0 -2.424958 3.050206 1.586347

H 0 -2.518597 2.008861 -0.768628

H 0 -2.193308 0.312541 2.756175

H 0 -0.997579 0.101093 5.256865

H 0 -0.272535 3.016812 0.001726

H 0 0.148171 -1.897490 6.148038

H 0 0.940530 -1.607429 1.306214

H 0 -4.560325 2.022937 2.761217

H 0 -3.095831 0.541699 -0.026472

H 0 -3.974782 2.839568 0.774015

H 0 2.914851 2.397846 -0.430474

H 0 -3.356439 -1.710735 2.690327

H 0 -3.914498 -1.318359 0.880957

H 0 -1.087471 -4.035481 6.423268

C 0 -5.054763 0.514155 4.809900

H 0 -5.670136 -0.173338 4.229370

H 0 -5.215921 0.305950 5.871547

H 0 -5.387561 1.537408 4.623740

H 0 -3.043020 1.226842 4.950576

**Int6S** (-8045.70295)

C -1 7.281669 0.460895 1.969230

C 0 6.042366 0.581943 2.862012

C 0 6.368350 0.256841 4.324116

C 0 5.412759 1.977287 2.746181

H 0 7.023439 0.580960 0.914388

H 0 8.027636 1.225525 2.217608

H 0 5.305544 -0.153805 2.514581

H 0 7.088410 0.976865 4.733421

H 0 5.469225 0.287995 4.947395

H 0 6.807465 -0.742023 4.421097

H 0 4.458691 2.036794 3.281119

H 0 6.081327 2.734414 3.175416

H 0 5.242357 2.270647 1.703826

C -1 2.866858 -2.655383 6.140548

C 0 3.204381 -2.031399 4.779519

C 0 2.711255 -2.906519 3.621133

C 0 2.628793 -0.616939 4.655461

H 0 1.782427 -2.772295 6.253764

H 0 3.317046 -3.647205 6.250687

H 0 4.298040 -1.956692 4.703870

H 0 1.615924 -2.967494 3.630829

H 0 3.013994 -2.484129 2.658333

H 0 3.103392 -3.927059 3.687728

H 0 1.533982 -0.638117 4.717276

H 0 2.993233 0.035960 5.454988

H 0 2.896772 -0.167439 3.695304

C -1 0.466005 0.418417 10.770847

C 0 0.925034 0.714118 9.338682

C 0 0.025869 0.014851 8.312853

C 0 0.973124 2.224252 9.080857

H 0 -0.552071 0.794497 10.935948

H 0 0.456750 -0.658496 10.972787

H 0 1.943606 0.316369 9.221849

H 0 -0.982536 0.443477 8.330835

H 0 0.415375 0.130245 7.295597

H 0 -0.061415 -1.056011 8.523814

H 0 -0.018429 2.672463 9.216303

H 0 1.662925 2.719890 9.770897

H 0 1.299924 2.445441 8.060082

C -1 -3.085955 -4.133162 12.676065

C 0 -3.257274 -5.442190 11.888670

O 0 -4.291159 -5.670283 11.266066

C 0 -2.821878 -2.980675 11.692685

H 0 -4.017744 -3.965400 13.221750

H 0 -1.900004 -3.151166 11.127093

H 0 -3.642875 -2.904128 10.975125

H 0 -2.730758 -2.027540 12.221635

N 0 -2.194176 -6.306605 11.887994

C -1 -2.114193 -7.479768 11.011858

C 0 -0.949589 -7.386943 10.015216

C 0 -0.938129 -6.118739 9.190232

C 0 -1.969183 -5.825556 8.286350

C 0 0.119822 -5.211336 9.314932

C 0 -1.924536 -4.669769 7.507236

C 0 0.167430 -4.050497 8.541943

C 0 -0.852475 -3.783025 7.630091

H 0 -3.074230 -7.519973 10.493348

H 0 -1.002975 -8.266157 9.360704

H 0 -0.000080 -7.469127 10.558427

H 0 -2.818528 -6.495779 8.190892

H 0 -1.351782 -6.014679 12.361503

H 0 0.924734 -5.425929 10.013689

H 0 -2.730732 -4.462243 6.812368

H 0 1.003652 -3.365112 8.641574

H 0 -0.814035 -2.889308 7.019507

C -1 0.152974 -6.400700 -0.188080

C 0 1.009211 -5.329538 -0.871610

C 0 0.372592 -4.865993 -2.187155

C 0 1.234238 -4.139616 0.066520

H 0 0.641738 -6.779943 0.716374

H 0 -0.818122 -5.985590 0.105665

H 0 1.989048 -5.770319 -1.105671

H 0 -0.605443 -4.410330 -1.998218

H 0 0.996503 -4.120445 -2.690205

H 0 0.222924 -5.705314 -2.875462

H 0 0.273519 -3.675726 0.320798

H 0 1.715568 -4.448901 1.001135

H 0 1.864311 -3.379278 -0.404302

C -1 -3.593644 -6.933617 3.872238

C 0 -3.176199 -6.439878 2.492086

S 0 -2.330808 -4.811546 2.479984

C 0 -0.783333 -5.259149 3.340885

H 0 -2.731605 -7.068387 4.532866

H 0 -4.280003 -6.230159 4.350924

H 0 -4.056252 -6.295255 1.862051

H 0 -2.521532 -7.160039 1.988664

H 0 -0.346521 -6.155310 2.893674

H 0 -0.084369 -4.433715 3.200046

H 0 -0.939021 -5.414445 4.411293

C -1 -8.593101 -6.748065 0.712696

C 0 -7.788078 -5.627029 1.309523

C 0 -6.487021 -5.360018 0.873441

C 0 -8.352474 -4.769464 2.263390

C 0 -5.791406 -4.238939 1.322648

C 0 -7.678344 -3.632698 2.702171

C 0 -6.414317 -3.340547 2.188796

O 0 -5.812436 -2.142611 2.506303

H 0 -9.126007 -7.321214 1.478870

H 0 -9.347847 -6.342339 0.028705

H 0 -6.014846 -6.027750 0.156753

H 0 -9.357400 -4.965496 2.625555

H 0 -4.782384 -4.034360 0.987724

H 0 -8.139620 -2.937602 3.395516

H 0 -5.634800 -1.702988 1.646663

C -1 -3.718552 -4.901547 -2.924611

C 0 -3.441483 -3.895063 -1.797907

C 0 -4.621974 -2.950975 -1.589029

C 0 -4.527738 -1.974669 -0.424129

O 0 -3.466137 -1.845054 0.234619

O 0 -5.616928 -1.342490 -0.159933

H 0 -3.922218 -4.387867 -3.870712

H 0 -2.547413 -3.305342 -2.026081

H 0 -3.217857 -4.425357 -0.864640

H 0 -5.546953 -3.517424 -1.430414

H 0 -4.797558 -2.350774 -2.489502

C -1 2.463041 -1.588933 -6.455027

C 0 2.762082 -1.392049 -4.984420

C 0 1.736514 -1.464704 -4.031675

C 0 4.066342 -1.156208 -4.530679

C 0 2.001939 -1.314561 -2.669725

C 0 4.344996 -1.020178 -3.168157

C 0 3.311738 -1.103771 -2.231083

H 0 3.234680 -1.135493 -7.083737

H 0 2.416430 -2.655033 -6.708208

H 0 0.715661 -1.637123 -4.359416

H 0 4.875150 -1.081613 -5.253523

H 0 1.189455 -1.354105 -1.951651

H 0 5.361567 -0.822978 -2.837697

H 0 3.519882 -0.994511 -1.171117

C -1 2.556985 4.063958 -4.608937

C 0 3.068052 3.791257 -3.189603

C 0 3.595415 2.367227 -3.022182

N 0 4.142888 2.155721 -1.667551

H 0 3.354467 3.931762 -5.348309

H 0 1.744984 3.377003 -4.868942

H 0 2.271872 3.960904 -2.454445

H 0 3.869925 4.503524 -2.944837

H 0 4.347320 2.153669 -3.795822

H 0 2.785245 1.646571 -3.166781

H 0 4.948745 2.759499 -1.509587

H 0 4.505754 1.206199 -1.599149

C -1 -2.422015 0.948826 -5.153487

C 0 -1.904661 0.893234 -3.708498

C 0 -1.975039 -0.541330 -3.169353

C 0 -0.492115 1.481213 -3.595920

H 0 -1.788789 0.340686 -5.810854

H 0 -2.569473 1.510499 -3.085364

H 0 -3.012511 -0.886256 -3.142357

H 0 -1.409329 -1.225672 -3.812498

H 0 -1.560803 -0.617375 -2.160025

H 0 0.215996 0.919158 -4.215055

H 0 -0.473322 2.526200 -3.927889

H 0 -0.126482 1.447269 -2.565116

C -1 -8.784267 -2.890388 -3.160923

C 0 -8.985497 -3.905858 -2.039791

S 0 -10.066584 -3.323271 -0.679440

C 0 -9.039075 -1.951356 -0.048754

H 0 -9.742632 -2.587772 -3.589554

H 0 -8.280393 -1.990941 -2.800579

H 0 -8.028173 -4.210140 -1.601211

H 0 -9.473332 -4.810520 -2.416811

H 0 -8.002705 -2.272114 0.071188

H 0 -9.089264 -1.089456 -0.712515

H 0 -9.437685 -1.674364 0.928836

C -1 -11.413357 -0.217180 -4.324373

C 0 -10.650733 0.401789 -3.175349

C 0 -9.275880 0.640742 -3.259096

C 0 -11.297376 0.741323 -1.978563

C 0 -8.562836 1.201268 -2.198835

C 0 -10.605199 1.315580 -0.914963

C 0 -9.233333 1.549240 -1.025507

O 0 -8.568960 2.093950 0.058521

H 0 -10.757971 -0.395968 -5.180650

H 0 -11.855094 -1.177010 -4.033404

H 0 -8.734047 0.365278 -4.159291

H 0 -12.363434 0.555825 -1.877125

H 0 -7.488418 1.303875 -2.283855

H 0 -11.111486 1.584649 0.005858

H 0 -7.883745 2.716469 -0.279854

C -1 -5.818626 4.554584 -3.596904

C 0 -4.861661 3.855139 -2.639044

C 0 -5.581358 3.232071 -1.471170

O 0 -6.635968 3.680315 -1.006633

O 0 -4.993535 2.162134 -0.988891

H 0 -6.373652 5.346998 -3.089440

H 0 -4.132238 4.564111 -2.227013

H 0 -4.280774 3.073770 -3.136924

C -1 -9.497422 6.950132 -2.623579

C 0 -9.139395 6.349582 -1.267883

C 0 -9.762416 4.969579 -1.081772

O 0 -7.717470 6.323375 -1.131139

H 0 -9.167032 6.286189 -3.430078

H 0 -9.523797 7.017001 -0.480788

H 0 -10.853665 5.022469 -1.145629

H 0 -9.418231 4.283207 -1.862745

H 0 -9.499543 4.537936 -0.112364

H 0 -7.413913 5.401824 -1.092827

N 0 -7.451031 4.947799 6.266577

C 0 -6.351526 4.008641 5.960018

C 0 -5.149793 4.597282 5.220897

O 0 -4.130442 3.916246 5.098570

C 0 -7.019553 2.903661 5.110388

C 0 -8.495127 2.967974 5.521896

C 0 -8.732389 4.470295 5.718757

H 0 -5.951071 3.608575 6.896175

H 0 -6.551578 1.933547 5.284337

H 0 -6.914134 3.136520 4.045193

H 0 -8.647898 2.445401 6.472701

H 0 -9.167368 2.530919 4.779640

H 0 -8.947054 4.960388 4.759813

H 0 -9.557011 4.686961 6.399434

N 0 -5.319273 5.824915 4.678601

C -1 -4.224607 6.582541 4.088830

C 0 -4.475467 7.035848 2.650424

C 0 -4.675182 5.923057 1.609255

C 0 -4.824707 6.554007 0.221251

C 0 -3.537752 4.894327 1.626850

H 0 -3.345827 5.938380 4.146440

H 0 -3.611603 7.646775 2.352168

H 0 -5.343644 7.708445 2.623849

H 0 -5.609406 5.395628 1.846566

H 0 -5.682789 7.227846 0.166093

H 0 -3.919428 7.112446 -0.046630

H 0 -6.171830 6.308211 4.921954

H 0 -4.985847 5.788983 -0.539753

H 0 -3.488346 4.349497 2.573294

H 0 -3.680336 4.162136 0.825058

H 0 -2.565450 5.376154 1.463968

C -1 -0.160191 5.152886 5.413639

C 0 -0.835283 3.787758 5.509176

S 0 -0.696345 2.776721 3.987006

C 0 1.099917 2.470749 3.901708

H 0 0.919217 5.062001 5.256709

H 0 -0.574908 5.733065 4.583964

H 0 -1.913969 3.891908 5.652243

H 0 -0.433764 3.206424 6.346216

H 0 1.646287 3.359835 3.581321

H 0 1.477420 2.125135 4.866808

H 0 1.247660 1.687399 3.158794

C -1 -0.794172 9.088043 -2.940253

C 0 -0.488757 7.582451 -2.886190

C 0 -1.376942 6.880912 -1.841447

C 0 1.004612 7.344849 -2.626542

C 0 -1.237674 5.355475 -1.793212

H 0 -0.561634 9.564562 -1.980302

H 0 -0.732579 7.152163 -3.869391

H 0 -2.424422 7.136567 -2.046118

H 0 -1.152891 7.298019 -0.849268

H 0 1.619145 7.859986 -3.372820

H 0 1.290359 7.727138 -1.638697

H 0 1.263585 6.282985 -2.659341

H 0 -0.231926 5.043645 -1.495098

H 0 -1.937179 4.925156 -1.069387

H 0 -1.445398 4.904524 -2.770725

C -1 -8.525074 6.609730 7.776867

C 0 -7.297466 5.825147 7.319237

O 0 -6.203068 5.974895 7.851060

H 0 -9.216137 5.959277 8.324142

H 0 -8.181612 7.397611 8.445764

H 0 -1.851791 9.272257 -3.155725

H 0 -0.199082 9.586297 -3.712546

H 0 2.175532 5.084922 -4.703225

H 0 3.223562 -2.026448 6.964276

H 0 1.119745 0.896210 11.507648

H 0 -2.017738 -8.390963 11.613954

H 0 -2.272572 -4.211633 13.406932

H 0 -4.101694 -7.900993 3.788727

H 0 -7.961916 -7.441522 0.149687

H 0 -8.164680 -3.320134 -3.956892

H 0 -4.591832 -5.521325 -2.690812

H 0 -2.864245 -5.567543 -3.080132

H 0 -0.027257 -7.253752 -0.852289

H 0 1.498722 -1.148726 -6.726859

H 0 -2.417801 1.973395 -5.542496

H 0 -3.444556 0.562319 -5.217660

H 0 -5.265696 4.996360 -4.430046

H 0 -8.999989 7.915554 -2.752972

H 0 -10.578652 7.099625 -2.719969

H 0 -6.542279 3.844306 -4.007697

H 0 -12.233122 0.429469 -4.656974

H 0 -9.073410 7.047271 6.937573

H 0 -4.026780 7.459878 4.717852

H 0 -0.316212 5.716063 6.340410

O 0 7.142726 0.843963 -1.698981

O 0 -6.178637 0.976994 0.854713

H 0 7.912584 0.426165 -1.273184

H 0 7.016432 1.651581 -1.174503

H 0 -7.142666 1.131123 0.772718

H 0 -5.989539 0.047444 0.537182

C -1 5.110148 1.406399 8.853699

C 0 4.674870 2.742010 8.238973

C 0 5.850727 3.722302 8.155150

C 0 4.044261 2.534090 6.857136

H 0 4.264847 0.717849 8.948288

H 0 5.866945 0.919367 8.225424

H 0 3.913472 3.182810 8.896384

H 0 6.640966 3.322191 7.508289

H 0 5.536835 4.685919 7.739926

H 0 6.288982 3.906951 9.141721

H 0 3.654776 3.472877 6.448558

H 0 4.784505 2.145603 6.148394

H 0 3.216786 1.819001 6.901082

C -1 -7.139980 -6.830969 9.564075

C 0 -6.727675 -6.069010 8.321103

C 0 -5.793258 -5.025972 8.400007

C 0 -7.290572 -6.360441 7.070387

C 0 -5.456033 -4.281902 7.266978

C 0 -6.946846 -5.626220 5.933447

C 0 -6.030982 -4.576385 6.029000

H 0 -6.347580 -6.805085 10.316071

H 0 -8.037409 -6.387086 10.013032

H 0 -5.329149 -4.807040 9.356697

H 0 -8.010200 -7.171942 6.988180

H 0 -4.751545 -3.461915 7.353980

H 0 -7.394878 -5.862116 4.972316

H 0 -5.774507 -3.993151 5.150226

H 0 -7.376694 -7.874294 9.331568

H 0 5.544594 1.547444 9.849484

H 0 7.763693 -0.515659 2.078252

C -1 6.647831 5.725883 0.042909

C 0 7.098509 4.555625 -0.797812

O 0 6.836772 3.390978 -0.461860

H 0 7.076223 5.621672 1.043036

N 0 7.796916 4.842005 -1.920435

C 0 8.322585 3.825091 -2.819963

C 0 9.715097 3.292746 -2.467837

O 0 10.539437 3.070287 -3.347342

H 0 7.649756 2.960551 -2.811519

H 0 8.060822 5.801209 -2.088234

N 0 9.938601 3.060981 -1.139072

C -1 11.057098 2.194053 -0.766048

C 0 10.611152 0.758856 -0.963990

O 0 9.619336 0.344491 -0.343845

H 0 11.926034 2.478476 -1.354262

H 0 9.111041 2.953625 -0.560749

N 0 11.270535 0.002300 -1.850100

C -1 10.756991 -1.337012 -2.120019

C 0 11.768833 -1.916969 -3.115142

C 0 12.257031 -0.674287 -3.875670

C 0 12.341406 0.401728 -2.784555

H 0 11.325324 -2.674693 -3.764795

H 0 12.602496 -2.381379 -2.576679

H 0 11.515553 -0.374888 -4.623880

H 0 13.211986 -0.821288 -4.385474

H 0 13.314630 0.376311 -2.278028

H 0 12.154496 1.404454 -3.170275

H 0 9.751975 -1.264277 -2.554075

H 0 5.561046 5.676386 0.149708

H 0 6.926343 6.696847 -0.372264

H 0 8.373957 4.225355 -3.832402

H 0 11.276618 2.345854 0.293536

H 0 10.668867 -1.906427 -1.192109

N 0 -3.116312 0.091946 2.076333

C 0 -5.078624 -0.646606 7.741780

C 0 -3.688770 -0.752442 7.761909

C 0 -3.000698 1.391258 1.397518

C 0 -2.217389 1.259444 0.084738

C 0 -2.986931 -0.989292 6.578884

C 0 -3.663780 -1.144490 5.365114

C 0 -0.762738 0.918126 0.295922

C 0 -2.925926 -1.368439 4.045530

C 0 0.212365 1.923627 0.229585

C 0 1.557380 1.638006 0.452209

C 0 -2.878674 -0.062134 3.325195

C 0 1.928116 0.325183 0.798280

C 0 -5.062181 -1.033466 5.351161

C 0 -5.761277 -0.786949 6.532223

C 0 0.973696 -0.683835 0.865049

C 0 -0.364661 -0.392746 0.591942

O 0 3.243401 0.051037 1.064809

O 0 2.530607 2.597953 0.400995

H 0 -3.144490 -0.655632 8.696142

H 0 -1.906832 -1.042501 6.611584

H 0 3.691585 0.914626 1.080332

H 0 -3.475044 -2.098780 3.443725

H 0 -1.099726 -1.192118 0.594263

H 0 -2.513718 2.089511 2.078312

H 0 -2.312267 2.214611 -0.437509

H 0 -2.568100 0.818582 3.888279

H 0 -5.594734 -1.167420 4.415755

H 0 -0.063843 2.947938 -0.001445

H 0 -6.845115 -0.726175 6.509029

H 0 1.286430 -1.687825 1.120447

H 0 -3.364649 -0.731911 1.444813

H 0 -2.705948 0.508698 -0.540595

H 0 -4.016864 1.734146 1.211846

H 0 3.115358 2.444018 -0.427094

H 0 -5.626386 -0.470078 8.662141

C 0 -1.464225 -1.839954 4.193740

H 0 -1.029117 -2.016410 3.208022

H 0 -0.844482 -1.095256 4.701314

H 0 -1.436952 -2.772366 4.760121

O 0 -5.596820 -0.039983 -2.776300

H 0 -5.040265 0.642616 -2.374672

H 0 -5.899019 -0.538676 -1.995852

H 0 -5.541360 1.730153 -0.195414

**Int7S** (-8045.710495)

C -1 7.281678 0.460918 1.968756

C 0 5.753568 0.518776 2.036789

C 0 5.211966 -0.529720 3.016208

C 0 5.271042 1.928448 2.407245

H 0 7.685031 1.195125 1.268207

H 0 7.716763 0.665789 2.955470

H 0 5.382755 0.279084 1.028145

H 0 5.608118 -0.349219 4.022632

H 0 4.121462 -0.502501 3.070953

H 0 5.505864 -1.541680 2.717789

H 0 4.178401 2.005011 2.395780

H 0 5.614594 2.195330 3.414368

H 0 5.661215 2.669512 1.703628

C -1 2.866828 -2.656142 6.141140

C 0 2.215099 -1.410133 5.523392

C 0 1.620318 -1.705906 4.143273

C 0 1.143340 -0.841976 6.459215

H 0 2.119996 -3.442528 6.305972

H 0 3.644454 -3.062110 5.486194

H 0 2.997105 -0.649181 5.397429

H 0 0.822987 -2.452564 4.228937

H 0 1.182565 -0.809944 3.693809

H 0 2.369321 -2.090318 3.445630

H 0 0.320739 -1.555022 6.581385

H 0 1.552379 -0.630502 7.451739

H 0 0.714990 0.086969 6.068184

C -1 0.459239 0.413169 10.775828

C 0 -0.381091 0.714623 9.527149

C 0 -1.515053 -0.303507 9.363853

C 0 -0.924630 2.147538 9.549998

H 0 -0.155467 0.476452 11.682119

H 0 0.883728 -0.595696 10.731815

H 0 0.276610 0.627239 8.654256

H 0 -2.221004 -0.237942 10.200870

H 0 -2.081591 -0.129955 8.443774

H 0 -1.134824 -1.328485 9.333723

H 0 -1.595161 2.299460 10.404589

H 0 -0.115637 2.882124 9.626241

H 0 -1.492536 2.369289 8.639292

C -1 -3.085939 -4.133161 12.676100

C 0 -3.258453 -5.431697 11.870531

O 0 -4.281275 -5.645106 11.225112

C 0 -2.774291 -2.974463 11.715732

H 0 -4.029914 -3.955806 13.197580

H 0 -1.838455 -3.151026 11.177357

H 0 -3.568620 -2.877279 10.971596

H 0 -2.686415 -2.028282 12.257117

N 0 -2.202480 -6.306863 11.886534

C -1 -2.114357 -7.480138 11.011919

C 0 -1.046430 -7.319906 9.916470

C 0 -1.211338 -6.069905 9.079962

C 0 -2.325810 -5.895166 8.248679

C 0 -0.251228 -5.053113 9.129577

C 0 -2.468094 -4.741620 7.478536

C 0 -0.390946 -3.892272 8.366340

C 0 -1.502058 -3.734516 7.538082

H 0 -3.107690 -7.591317 10.573276

H 0 -1.085433 -8.213469 9.280416

H 0 -0.051648 -7.315802 10.378575

H 0 -3.099629 -6.656179 8.209898

H 0 -1.366972 -6.022564 12.376462

H 0 0.620755 -5.175288 9.767373

H 0 -3.340721 -4.626258 6.846460

H 0 0.367325 -3.117387 8.416206

H 0 -1.622776 -2.829727 6.950462

C -1 0.153105 -6.400939 -0.188108

C 0 0.709062 -4.999310 -0.477852

C 0 -0.413933 -4.035499 -0.879541

C 0 1.489979 -4.452516 0.723814

H 0 0.950955 -7.102984 0.077340

H 0 -0.555087 -6.362464 0.648848

H 0 1.405950 -5.079461 -1.324108

H 0 -1.124969 -3.907715 -0.054628

H 0 -0.017777 -3.049128 -1.136036

H 0 -0.964631 -4.409604 -1.748573

H 0 0.823530 -4.313547 1.583810

H 0 2.289968 -5.136199 1.028480

H 0 1.947625 -3.484578 0.495245

C -1 -3.593507 -6.933652 3.872196

C 0 -3.096821 -6.250322 2.600175

S 0 -2.483126 -4.535937 2.835235

C 0 -1.037951 -4.865398 3.899459

H 0 -2.794182 -7.035744 4.611587

H 0 -4.406626 -6.368089 4.336610

H 0 -3.904001 -6.150212 1.869007

H 0 -2.301627 -6.834328 2.124416

H 0 -0.381784 -5.607906 3.437451

H 0 -0.490148 -3.926365 3.983486

H 0 -1.330314 -5.188188 4.899939

C -1 -8.593142 -6.748043 0.712697

C 0 -8.004391 -5.461265 1.258008

C 0 -6.773876 -5.431449 1.931441

C 0 -8.674107 -4.243275 1.091176

C 0 -6.214882 -4.234180 2.381505

C 0 -8.127079 -3.035680 1.525893

C 0 -6.883456 -3.026206 2.158320

O 0 -6.288847 -1.868716 2.599848

H 0 -9.470557 -6.540259 0.095315

H 0 -7.863697 -7.285937 0.097390

H 0 -6.233918 -6.360343 2.099153

H 0 -9.640495 -4.248661 0.599079

H 0 -5.253313 -4.213253 2.883314

H 0 -8.656642 -2.100873 1.363431

H 0 -6.497837 -1.116055 2.016088

C -1 -3.718635 -4.901626 -2.924639

C 0 -3.873950 -3.530054 -2.268040

C 0 -4.326164 -3.633001 -0.796232

C 0 -4.724758 -2.288985 -0.245567

O 0 -4.012157 -1.606667 0.488348

O 0 -5.929213 -1.904240 -0.660141

H 0 -4.673036 -5.439381 -2.942946

H 0 -4.591092 -2.928264 -2.834479

H 0 -2.924585 -2.987778 -2.303859

H 0 -3.522029 -4.033720 -0.174989

H 0 -5.196932 -4.292140 -0.717135

C -1 2.462980 -1.589028 -6.455013

C 0 2.717821 -1.392597 -4.976649

C 0 1.652938 -1.388643 -4.065744

C 0 4.015865 -1.233439 -4.474414

C 0 1.874479 -1.243104 -2.696224

C 0 4.248888 -1.102543 -3.102914

C 0 3.176602 -1.115141 -2.207346

H 0 3.270332 -1.165885 -7.059703

H 0 2.390275 -2.654704 -6.704070

H 0 0.637632 -1.496314 -4.435362

H 0 4.855010 -1.214572 -5.165590

H 0 1.033435 -1.213368 -2.011548

H 0 5.261536 -0.962973 -2.734041

H 0 3.342762 -1.016134 -1.139538

C -1 2.557036 4.064123 -4.608958

C 0 2.932829 3.739931 -3.157068

C 0 3.567565 2.357521 -3.012850

N 0 3.999635 2.097436 -1.623747

H 0 3.438972 4.045969 -5.258656

H 0 1.840741 3.333010 -4.997559

H 0 2.047021 3.791356 -2.512314

H 0 3.632591 4.500532 -2.780613

H 0 4.409259 2.261079 -3.714943

H 0 2.844110 1.582775 -3.282286

H 0 4.735787 2.750814 -1.354959

H 0 4.440131 1.178885 -1.588496

C -1 -2.421964 0.948875 -5.153516

C 0 -1.906529 0.982479 -3.709639

C 0 -1.981626 -0.419439 -3.094358

C 0 -0.497416 1.583327 -3.628074

H 0 -1.778317 0.315427 -5.776823

H 0 -2.578772 1.627408 -3.127743

H 0 -3.022048 -0.754650 -3.061090

H 0 -1.418109 -1.141806 -3.697514

H 0 -1.570878 -0.440961 -2.081358

H 0 0.212899 1.002909 -4.227109

H 0 -0.487852 2.614045 -4.002562

H 0 -0.127021 1.593327 -2.598198

C -1 -8.784099 -2.890382 -3.160715

C 0 -9.091704 -4.145366 -2.358361

S 0 -10.861926 -4.329067 -1.894307

C 0 -11.203763 -2.703305 -1.132899

H 0 -9.402783 -2.825978 -4.060550

H 0 -8.949862 -1.991501 -2.567486

H 0 -8.482197 -4.174325 -1.451047

H 0 -8.876048 -5.051022 -2.933755

H 0 -10.493107 -2.475202 -0.335145

H 0 -11.181570 -1.897937 -1.865374

H 0 -12.205780 -2.766034 -0.703481

C -1 -11.413623 -0.217114 -4.324539

C 0 -10.653019 0.383797 -3.171289

C 0 -9.254800 0.439769 -3.190967

C 0 -11.310404 0.845638 -2.024048

C 0 -8.532428 0.920103 -2.104277

C 0 -10.602036 1.350284 -0.933871

C 0 -9.208390 1.395713 -0.975834

O 0 -8.524468 1.913904 0.103296

H 0 -11.487917 -1.307920 -4.226918

H 0 -12.433966 0.173793 -4.381506

H 0 -8.705657 0.082764 -4.057338

H 0 -12.396408 0.815703 -1.984727

H 0 -7.452480 0.917467 -2.144156

H 0 -11.113945 1.731571 -0.056643

H 0 -7.778178 2.489567 -0.255041

C -1 -5.818714 4.554606 -3.596919

C 0 -4.975083 3.614977 -2.719565

C 0 -5.717148 2.818698 -1.647636

O 0 -6.654083 3.369087 -0.993716

O 0 -5.316142 1.634597 -1.409632

H 0 -6.346745 5.298007 -3.000501

H 0 -4.221893 4.203776 -2.177172

H 0 -4.420556 2.899392 -3.332307

C -1 -9.497460 6.950095 -2.623687

C 0 -9.189990 5.622947 -1.952614

C 0 -9.346181 4.458800 -2.931512

O 0 -7.870716 5.746077 -1.439386

H 0 -8.815027 7.104396 -3.465749

H 0 -9.897001 5.466708 -1.118155

H 0 -10.398182 4.332115 -3.207732

H 0 -8.772468 4.643405 -3.844570

H 0 -9.003159 3.519230 -2.497919

H 0 -7.491258 4.861458 -1.257529

N 0 -7.457637 4.958310 6.247553

C 0 -6.384459 3.996476 5.909806

C 0 -5.193051 4.567327 5.138269

O 0 -4.265392 3.816564 4.808416

C 0 -7.088788 2.914738 5.056566

C 0 -8.567652 3.048047 5.434802

C 0 -8.733813 4.558919 5.631211

H 0 -5.978905 3.575038 6.833824

H 0 -6.673692 1.926433 5.250433

H 0 -6.951624 3.133791 3.992316

H 0 -8.765079 2.530598 6.380121

H 0 -9.241864 2.645627 4.674975

H 0 -8.867633 5.064527 4.665592

H 0 -9.577578 4.820751 6.270495

N 0 -5.263361 5.872581 4.815889

C -1 -4.224605 6.582914 4.088920

C 0 -4.762641 7.373592 2.895382

C 0 -5.567502 6.565273 1.864645

C 0 -5.982792 7.472828 0.701065

C 0 -4.805164 5.333289 1.360270

H 0 -3.506843 5.825253 3.769799

H 0 -3.901398 7.839066 2.396674

H 0 -5.384189 8.203959 3.259111

H 0 -6.480592 6.205755 2.360756

H 0 -6.500777 8.367916 1.065945

H 0 -5.096899 7.810236 0.147214

H 0 -6.053738 6.387937 5.173977

H 0 -6.646142 6.957334 0.001720

H 0 -4.582547 4.634010 2.171954

H 0 -5.394105 4.803523 0.606140

H 0 -3.852308 5.629574 0.900989

C -1 -0.158630 5.154691 5.411905

C 0 -0.911539 3.825958 5.452815

S 0 -0.778106 2.825926 3.919294

C 0 0.996183 2.400159 3.901782

H 0 0.918916 5.005054 5.295940

H 0 -0.508225 5.772679 4.580071

H 0 -1.988684 3.987912 5.556548

H 0 -0.577200 3.212131 6.297133

H 0 1.617811 3.274304 3.700847

H 0 1.293151 1.937807 4.846517

H 0 1.134894 1.684107 3.091680

C -1 -0.794258 9.088148 -2.940244

C 0 -0.611499 7.565475 -2.844657

C 0 -1.507059 6.973976 -1.739812

C 0 0.867608 7.213387 -2.638280

C 0 -1.485909 5.444527 -1.646802

H 0 -0.481992 9.574959 -2.008367

H 0 -0.932652 7.125243 -3.800636

H 0 -2.539332 7.305410 -1.910562

H 0 -1.205858 7.402689 -0.773348

H 0 1.488804 7.652326 -3.426661

H 0 1.226718 7.601807 -1.677001

H 0 1.038316 6.133451 -2.645077

H 0 -0.496745 5.063443 -1.374399

H 0 -2.191902 5.092022 -0.887337

H 0 -1.765797 4.983309 -2.600888

C -1 -8.525167 6.609821 7.776912

C 0 -7.314814 5.778876 7.345828

O 0 -6.240444 5.857149 7.932518

H 0 -9.275360 5.977278 8.263797

H 0 -8.174152 7.351259 8.493296

H 0 -1.841470 9.352965 -3.119473

H 0 -0.194429 9.509812 -3.753555

H 0 2.100533 5.054950 -4.686793

H 0 3.326194 -2.424037 7.108401

H 0 1.285975 1.123341 10.885724

H 0 -1.904711 -8.372542 11.612255

H 0 -2.295741 -4.235745 13.429070

H 0 -3.961502 -7.939772 3.640932

H 0 -8.898457 -7.425874 1.518622

H 0 -7.731523 -2.881443 -3.465418

H 0 -2.997020 -5.522979 -2.382695

H 0 -3.368276 -4.802799 -3.956373

H 0 -0.379715 -6.805405 -1.055504

H 0 1.523831 -1.119090 -6.762172

H 0 -2.435695 1.949650 -5.600286

H 0 -3.436976 0.540209 -5.193247

H 0 -5.167607 5.067064 -4.311975

H 0 -9.360450 7.776590 -1.919955

H 0 -10.526205 6.979318 -2.998452

H 0 -6.559252 3.985795 -4.166381

H 0 -10.918616 -0.012632 -5.278471

H 0 -9.006579 7.108156 6.930741

H 0 -3.698138 7.258939 4.775496

H 0 -0.320046 5.706386 6.344656

O 0 7.002569 0.783031 -1.584211

O 0 -6.436282 0.292312 0.595505

H 0 7.791974 0.380059 -1.182521

H 0 6.878092 1.590966 -1.061206

H 0 -5.950712 0.846299 -0.091733

H 0 -7.326018 0.718493 0.593018

C -1 5.116008 1.409618 8.849517

C 0 4.083728 1.317842 7.717657

C 0 2.880017 2.227177 7.992609

C 0 4.716070 1.646024 6.360322

H 0 5.962842 0.738284 8.671353

H 0 5.511217 2.429471 8.932773

H 0 3.721392 0.281037 7.676755

H 0 3.189445 3.278594 8.032576

H 0 2.122248 2.133471 7.207615

H 0 2.403571 1.982856 8.947101

H 0 3.991239 1.549225 5.545644

H 0 5.094164 2.675724 6.346734

H 0 5.556218 0.980058 6.138294

C -1 -7.139764 -6.830893 9.564181

C 0 -6.782750 -5.967565 8.374626

C 0 -6.072964 -4.771347 8.555890

C 0 -7.169817 -6.319646 7.074728

C 0 -5.787147 -3.940531 7.471825

C 0 -6.871678 -5.498919 5.984642

C 0 -6.183502 -4.300786 6.181327

H 0 -6.336636 -6.815342 10.306093

H 0 -8.049003 -6.462121 10.054825

H 0 -5.727907 -4.510916 9.551775

H 0 -7.713385 -7.247912 6.915649

H 0 -5.244785 -3.013490 7.630599

H 0 -7.181359 -5.783532 4.983042

H 0 -5.955723 -3.660618 5.335540

H 0 -7.327875 -7.866210 9.263888

H 0 4.672202 1.144722 9.815159

H 0 7.635256 -0.522768 1.645975

C -1 6.647780 5.725811 0.042904

C 0 7.064556 4.531466 -0.781060

O 0 6.788364 3.379652 -0.415247

H 0 7.091299 5.632667 1.037499

N 0 7.754537 4.779476 -1.917315

C 0 8.257713 3.731114 -2.792776

C 0 9.667439 3.231242 -2.463843

O 0 10.466610 2.974752 -3.356799

H 0 7.595562 2.860625 -2.718695

H 0 8.021974 5.731396 -2.117860

N 0 9.935495 3.061073 -1.132649

C -1 11.057432 2.194068 -0.765995

C 0 10.607446 0.760087 -0.970346

O 0 9.601981 0.353962 -0.367744

H 0 11.926044 2.479684 -1.354128

H 0 9.122628 2.966324 -0.532307

N 0 11.279794 -0.003475 -1.840567

C -1 10.757127 -1.337092 -2.120029

C 0 11.767862 -1.919722 -3.114844

C 0 12.270427 -0.676717 -3.865199

C 0 12.358618 0.391868 -2.767032

H 0 11.320011 -2.668959 -3.771341

H 0 12.594934 -2.395495 -2.576158

H 0 11.534735 -0.365464 -4.614215

H 0 13.226344 -0.828884 -4.371671
[truncated: 215,758 more chars]
